# Growth-Inhibiting Activity of Resveratrol Imine Analogs on Tumor Cells *In Vitro*

**DOI:** 10.1371/journal.pone.0170502

**Published:** 2017-01-23

**Authors:** Shan Wang, Ina Willenberg, Michael Krohn, Tanja Hecker, Sven Meckelmann, Chang Li, Yuanjiang Pan, Nils Helge Schebb, Pablo Steinberg, Michael Telamon Empl

**Affiliations:** 1 Institute for Food Toxicology and Analytical Chemistry, University of Veterinary Medicine Hannover, Hannover, Germany; 2 Institute of Food Chemistry, University of Wuppertal, Wuppertal, Germany; 3 Department of Chemistry, Zhejiang University, Hangzhou, P. R. China; University of South Alabama Mitchell Cancer Institute, UNITED STATES

## Abstract

Although resveratrol exerts manifold antitumorigenic effects *in vitro*, its efficacy against malignancies *in vivo* seems limited. This has been increasingly recognized in recent years and has prompted scientists to search for structurally related compounds with more promising anticarcinogenic and/or pharmacokinetic properties. A class of structurally modified resveratrol derivatives, so-called resveratrol imine analogs (IRA’s), might meet these requirements. Therefore, the biological activity of five of these compounds was examined and compared to that of resveratrol. Firstly, the antiproliferative potency of all five IRA’s was investigated using the p53 wildtype-carrying colorectal carcinoma cell line HCT-116^wt^. Then, using the former and a panel of various other tumor cell lines (including the p53 knockout variant HCT-116^p53-/-^), the growth-inhibiting and cell cycle-disturbing effects of the most potent IRA (IRA 5, 2-[[(2-hydroxyphenyl)methylene]amino]-phenol) were studied as was its influence on cyclooxygenase-2 expression and activity. Finally, rat liver microsomes were used to determine the metabolic stability of that compound. IRA 5 was clearly the most potent compound in HCT-116^wt^ cells, with an unusually high IC_50_-value of 0.6 μM. However, in the other five cell lines used, the antiproliferative activity was mostly similar to resveratrol and the effects on the cell cycle were heterogeneous. Although all cell lines were affected by treatment with IRA 5, cells expressing functional p53 seemed to react more sensitively, suggesting that this protein plays a modulating role in the induction of IRA 5-mediated biological effects. Lastly, IRA 5 led to contradictory effects on cyclooxygenase-2 expression and activity and was less glucuronidated than resveratrol. As IRA 5 is approximately 50 times more toxic towards HCT-116^wt^ cells, exerts different effects on the cyclooxygenase-2 and is metabolized to a lesser extent, it shows certain advantages over resveratrol and could therefore serve as basis for additional chemical modifications, potentially yielding compounds with more favorable biological and pharmacokinetic features.

## Introduction

**Fig 1:**
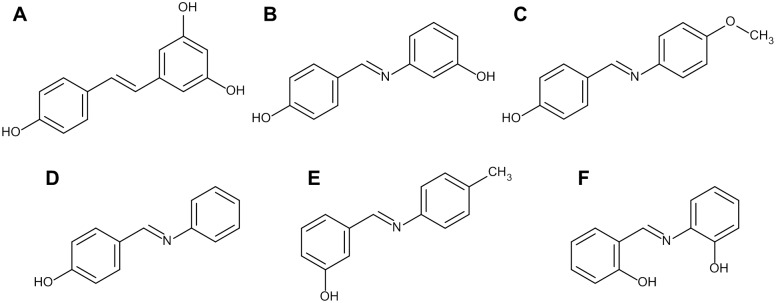
The structure of the test substances used in the present study. **A:** resveratrol, **B:** 3-[[(4-hydroxyphenyl)methylene]amino]-phenol (**IRA 1**), **C:** 4-[[(4-methoxyphenyl)imino]methyl]-phenol (**IRA 2**), **D:** 4-[(phenylimino)methyl]-phenol (**IRA 3**), **E:** 3-[[(4-methylphenyl)imino]methyl]-phenol (**IRA 4**) and **F:** 2-[[(2-hydroxyphenyl)methylene]amino]-phenol (**IRA 5**).

Since Jang et al. [1] published a study linking the natural stilbenoid resveratrol (Fig 1A) to cancer chemoprevention in the mid 1990’s, a plethora of studies have been performed to investigate this connection in more detail [2]. Up to now, a high number of published studies have reported that this polyphenol exerts manifold biological effects *in vitro*, strongly suggesting that it might inhibit or prevent the onset of cancer [2, 3]. For instance, the potential cancer-repressing effects investigated *in vitro* include anti-oxidative, anti-inflammatory, growth-inhibiting, pro-apoptotic, and anti-metastatic properties (reviewed in [4]). In addition, numerous animal studies suggest that resveratrol might indeed be able to inhibit carcinogenesis *in vivo* (reviewed in [2] and [5]). Nevertheless, not all animal studies have rendered promising results (see references [2] and [5] for a comprehensive listing of performed animal studies), and the outcomes of the few clinical trials conducted in human cancer patients are far from showing that resveratrol is notably helpful in preventing or treating cancer [6–8]. For example, in multiple myeloma patients, this compound even induced adverse effects [9]. Moreover, there is a rather vast discrepancy between resveratrol concentrations biologically active in cellular models *in vitro* (up to 500 μM but mostly in the 20–100 μM range; reviewed in [10]) and the maximum plasma concentrations (967 ng/ml = approx. 4 μM) achievable in humans after oral administration of very high doses (i.e. 5 g; [11]). The inconsistency between resveratrol concentrations that can be reached *in vivo* and those that are efficient *in vitro* as well as the absence of a clearly demonstrated *in vivo* efficacy can mostly be explained by the fast metabolization (i.e. glucuronidation and sulfonation) of this compound ([12] and reviewed in [13]). This results in a very low overall bioavailability, although the absorption of orally administered resveratrol is relatively high (reviewed in [13] and [14]). Consequently, it is not surprising that a number of studies proposing the search for molecules more suited for use in cancer therapy or chemoprevention and/or investigating the anticarcinogenic/chemopreventive efficacy as well as metabolic stability of natural or synthetic compounds related to resveratrol have been published (e.g. [15–22]).

Taking this into account, resveratrol imine analogs (IRA’s) might constitute a group of compounds that could meet these requirements. IRA’s bearing different functional groups at different positions of the aromatic rings have been synthesized by several groups (e.g. [23–26]). In spite of this and the fairly easy synthesis of most compounds [27], data on the biological activity of these substances is limited, especially regarding their anticarcinogenic properties. Up to the present time, only a limited number of reports on their anti-oxidative [23, 24, 26–29], anti-proliferative [23], cyclooxygenase-2 (COX-2)-inhibiting [30], and photoprotective effects [31] have been published.

For this reason, we examined the anticarcinogenic effects of five IRA’s (Fig 1B–1F) in a panel of six different human tumor cell lines. Moreover, their metabolic stability was investigated *in vitro* using rat liver microsomes (RLM’s).

## Methods

### Cell culture

A-431 (tissue of origin: epidermoid carcinoma) and HCT-116^wt^ cells (tissue of origin: colorectal carcinoma bearing a wildtype p53 protein) were purchased from the American Type Culture Collection (ATCC; Manassas, VA, USA), while HCT-116^p53-/-^ cells were kindly provided by Prof. Dr. R. Schneider-Stock (Institute of Pathology [Experimental Tumor Pathology], University Hospital Erlangen, Erlangen, Germany). These cells are isogenic to HCT-116^wt^ cells, with the exception of p53, which was knocked out by homologous recombination [32]. HCA-7 cells (tissue of origin: colorectal carcinoma) were obtained from the European Collection of Cell Cultures (ECACC; Salisbury, UK) and Caco-2 (tissue of origin: colorectal carcinoma) as well as LNCaP cells (tissue of origin: androgen-responsive prostate carcinoma) were acquired from the German Collection of Microorganisms and Cell Cultures (DSMZ; Braunschweig, Germany). All cell lines were cultured in a tempered (37°C) and humidified (95% rel. humidity) incubator providing an atmosphere containing 5% CO_2_. The culture medium used for the cells (DMEM in the case of A-431, Caco-2, HCA-7 and HCT-116^p53-/-^ cells and RPMI 1640 in the case of HCT-116^wt^ and LNCaP cells; both media were purchased from Biochrom, Berlin, Germany) was routinely supplemented with 10% fetal bovine serum (FBS; Biochrom), 2 mM L-glutamine (Biochrom) and 100 IU/ml penicillin/100 μg/ml streptomycin (Biochrom). In the case of the Caco-2 cells, the medium was additionally supplemented with 1% (v/v) nonessential amino acids (Biochrom), while the medium for LNCaP cells also included 10 mM HEPES buffer, 0.15% sodium bicarbonate (v/v), 1 mM sodium pyruvate (all Biochrom) as well as 4.5 g/l glucose (Carl Roth, Karlsruhe, Germany).

### Chemicals and enzymes

Resveratrol (Fig 1A; ≥ 99% purity) and nocodazole (≥ 99% purity) were acquired from Sigma-Aldrich (Schnelldorf, Germany). The IRA’s (≥ 99% purity) used in the present study were synthesized in-house as previously described [24] and comprise the following molecules: 3-[[(4-hydroxyphenyl)methylene]amino]-phenol (**IRA 1**; Fig 1B; CAS RN^®^: 27489-12-9), 4-[[(4-methoxyphenyl)imino]methyl]-phenol (**IRA 2**; Fig 1C; CAS RN^®^: 3230-50-0), 4-[(phenylimino)methyl]-phenol (**IRA 3**; Fig 1D; CAS RN^®^: 1879-73-2), 3-[[(4-methylphenyl)imino]methyl]-phenol (**IRA 4**; Fig 1E; CAS RN^®^: 17065-04-2) and 2-[[(2-hydroxyphenyl)methylene]amino]-phenol (**IRA 5**; Fig 1F; CAS RN^®^: 1761-56-4). Master stocks of all test substances were prepared in dimethyl sulfoxide (DMSO; Carl Roth) at a concentration of 100 mM. Working stocks were produced therefrom by an appropriate dilution in DMSO. Human recombinant COX-2 and arachidonic acid (AA) were purchased from Cayman Chemicals (Ann Arbor, MI, USA) and obtained through Biomol (Hamburg, Germany).

### Sulforhodamine B assay

The sulforhodamine B (SRB) assay was performed as previously described [20, 33] with additional modifications. Shortly, 1,000 (A-431, Caco-2, HCA-7, HCT-116^wt^, HCT-116^p53-/-^) or 3,000 (LNCaP) cells were seeded in 96 well plates (200 μl/well; TPP, Trasadingen, Switzerland) and given a 24-h attachment period. Thereafter, the cells were treated with the different test compounds or the solvent control (0.1% DMSO) for 48, 72 and 120 h. Then, the cells were fixed for 55 min by the addition of 50 μl 50% trichloracetic acid (Carl Roth), before the plates were washed 6–8 times with tap water and stained with SRB solution. Finally, following a washing step, 100 μl 10 mM Tris buffer (Carl Roth) were added to each well, and the absorption (wavelength: 510 nm) was measured using an Infinite^®^ 200 plate reader (Tecan, Crailsheim, Germany).

### Assessment of cell membrane integrity in HCA-7 cells

The short-term toxicity and disturbance of the cell membrane integrity (necrosis) possibly induced by resveratrol and IRA 5 was assessed in HCA-7 cells using the CytoTox-ONE™ Homogeneous Membrane Integrity Assay (Promega, Mannheim, Germany) as described earlier [21]. Briefly, 20,000 HCA-7 cells were seeded in 96 well plates (TPP) and incubated for 24 h. Then, the culture medium was substituted by fresh medium (110 μl/well) containing increasing concentrations of resveratrol as well as IRA 5, and the cells were again incubated for 6 and 24 h. Finally, the lactate dehydrogenase (LDH) activity in the supernatant was fluorimetrically recorded (excitation wavelength: 560 nm; emission wavelength: 590 nm) on an Infinite^®^ 200 plate reader (Tecan).

### Cell cycle analysis

The cell cycle analysis was performed as detailed elsewhere [21], with slight modifications. Briefly, 1.5 x 10^6^ cells were seeded on 10 cm culture dishes (TPP) and allowed to attach for 24 hours. After a 24-h incubation in FBS-free medium, the cells were treated with the test compounds or the solvent control for 24 and 48 h. Then, the supernatants as well as the cells were collected, centrifuged and the resulting pellet re-suspended in ice-cold phosphate-buffered saline (PBS). After another centrifugation step, 1 x 10^6^ cells were fixed in 70% ethanol and stored at 4°C for not more than one week. On the day of the analysis, the cells were incubated with 50 μg/ml ribonuclease A solution (Sigma-Aldrich), followed by the addition of 50 μg/ml propidium iodide (Sigma-Aldrich). Measurement of cellular DNA content (20,000 events per sample) was performed on an Accuri C6 flow cytometer (BD Biosciences, Heidelberg, Germany) and the obtained data files were analyzed using FlowJo (version 7.6.5; FlowJo, Ashland, OR, USA). The software analysis comprised two steps: 1. exclusion of doublet cells and cellular debris by drawing a fluorescence area vs. fluorescence height plot; 2. histogram deconvolution using the “Watson pragmatic” algorithm [34] integrated in FlowJo.

### Western blotting

The expression of COX-2 in HCA-7 cells was essentially performed as previously described [35], with several changes made to the protocol and buffers. In short, 1.6 x 10^6^ HCA-7 cells were incubated with 0.1% DMSO (solvent control) as well as 1, 50 and 100 μM IRA 5 for 24 h in 10 cm^2^ dishes (TPP) and lysed using a buffer containing 4 M urea (Carl Roth), 0.5% sodium dodecyl sulfate (SDS; Carl Roth), 62.5 mM Tris (Carl Roth) as well as protease inhibitors. The Pierce™ 660nm Protein Assay (Fisher Scientific, Schwerte, Germany) performed according to the manufacturer’s instructions was used for protein determination and 20 μg protein/sample were separated on a 10% polyacrylamide gel. Thereafter, the gel content was blotted onto a nitrocellulose membrane (GE, Amersham, UK) and COX-2 was detected by incubating the membrane overnight at 4°C with a rabbit monoclonal primary antibody (Cell Signaling Technologies, Danvers, MA, USA; distributed by New England Biolabs, Frankfurt am Main, Germany) diluted 1:1,000 in 5% skimmed milk (Carl Roth). The expression of glyceraldehyde 3-phosphate dehydrogenase (GAPDH) served as loading control and was determined in parallel by using a mouse monoclonal antibody (Santa Cruz, Heidelberg, Germany) diluted 1:2,000 in 5% skimmed milk after the membrane was cut at approximately 55 kDa. The HRP-conjugated secondary antibodies were purchased from Sigma-Aldrich (COX-2; polyclonal goat anti-mouse antibody diluted 1:1,000 in skimmed milk) or Santa Cruz (GAPDH; polyclonal goat anti-mouse antibody diluted 1:10,000 in skimmed milk) and the membranes were visualized on a ChemoCam Imager 3.2 (INTAS, Göttingen, Germany) using Immobilon Western (Merck, Darmstadt, Germany) ECL substrate.

Densitometry was performed using ImageJ (v. 1.48v; [36]) on the lowermost developed image files containing the COX-2 and GAPDH bands. The intensity of the COX-2 bands was corrected for inconsistencies in the amount of protein loaded onto the gel using the bands of the housekeeping protein (GAPDH). Even though the image files with the lowest exposure were chosen for densitometry, a few pixels in the control and 1 μM resveratrol bands of the housekeeping protein of one experiment were overexposed. Nonetheless, we performed the densitometric analysis, as we deemed this not to be biologically relevant.

### COX-2 activity assay

The determination of COX-2 activity in HCA-7 cells was performed as described before [35]. Briefly, HCA-7 cells were incubated with increasing concentrations of IRA 5 (max. 50 μM) for 24 h, the supernatant was collected, and prostaglandin E_2_ (PGE_2_) levels were determined by means of online-SPE-LC-MS.

### Glucuronidation assay

The glucuronidation assay was carried out as previously described [37]. In short, 20 μM of IRA 5 were incubated in 100 mM potassium phosphate buffer (pH 7.4) containing 10 mM MgCl_2_, 5 mM saccharo-1,4-lactone and 2 mM uridine 5'-diphospho-glucuronic acid (UDPGA) with RLM’s obtained from Sprague Dawley rats (Celsis, Baltimore, MD, USA; 1 mg protein/ml) for 40 minutes. The IRA 5 solution was prepared in DMSO, resulting in a final DMSO concentration of 2%. The reaction was terminated by addition of 200 μl ice-cold acetonitrile/acetic acid (HAc) 97:3 (v/v) containing 2 μM formononetin (Sigma-Aldrich) as internal standard (IS). After centrifugation at 13,000 rcf and 4°C for 20 min, the supernatant was analyzed by LC-UV in order to detect the amount of free test compound remaining after the incubation with the RLM’s. Separation was performed on a Merck-Hitachi HPLC system consisting of a L-7000 interface, a L-7100 quaternary pump, a L-7250 autosampler, a L-7455 photodiode array (PDA) detector and a CIL column oven equipped with a 100 mm x 3 mm Kinetex RP-18 column filled with 2.6 μm “fused core” particles (Phenomenex, Aschaffenburg, Germany). The temperature during the separation was 60°C. The analytes (injection volume of 20 μl) were separated using a binary gradient of 95:5 water/acetonitrile (v/v) with 0.1% HAc as solvent A and 95:5 acetonitrile/water (v/v) with 0.1% HAc as solvent B at a flow rate of 1 ml/min. The following gradient was used: 0–1 min, isocratic 15% B; 1.1–6 min, linear 15–75% B; 6.1–6.2 min, linear 75–100% B; 6.3–8 min, isocratic 100% B; 8.1 min return to the initial conditions and reconditioning for 6 min. The analytes were detected by the above-mentioned PDA detector operating at a detection frequency of 5 Hz with a slit of 4 nm. IRA 5 and the IS were quantified at a wavelength of 260 nm.

Quantification was performed by external calibration of the LC-UV signal of standards using formononetin as IS for the UV detection of IRA 5. For calibration, the test compound was sequentially diluted (0.2, 0.3, 0.6, 1, 2, 3, 5, 7, 10, 12 and 15 μM) in 50:50 methanol/water containing IS and 0.1% HAc. The analyte/IS area ratios were then fitted in a linear way, reciprocally weighted by concentration.

### Statistical analysis

The statistical analysis was performed using Prism (version 6.04; GraphPad, La Jolla, CA, USA), with a *p*-value ≤0.05 denoting a statistically significant difference to the corresponding control group. The absolute IC_50_ values (half-maximal inhibitory concentrations) were determined using nonlinear regression and all other assays were analyzed by either a one- or two-way analysis of variance (ANOVA) followed by appropriate post hoc tests mentioned in each figure or table caption.

## Results

### Cellular proliferation

**Fig 2:**
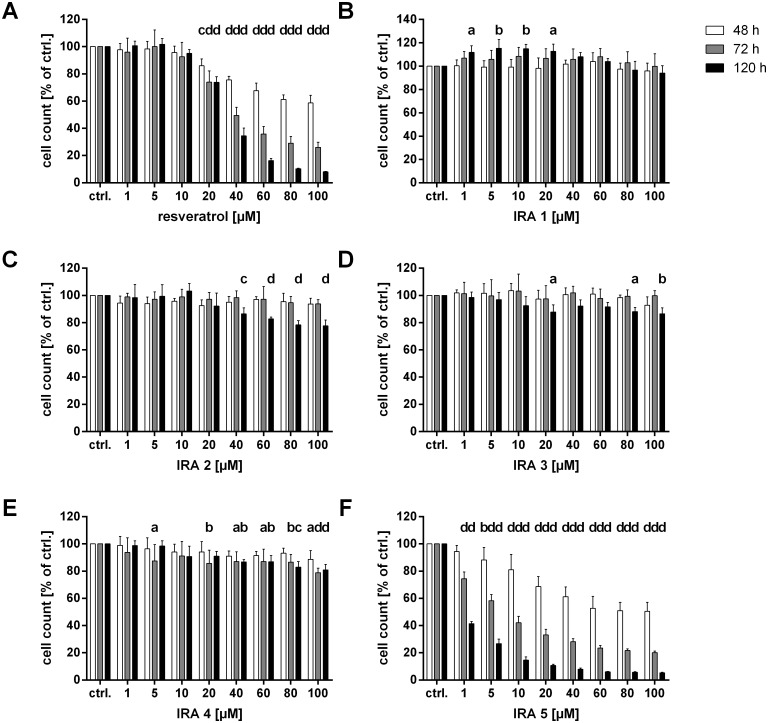
Growth-inhibitory effect of resveratrol (A) and IRA’s 1–5 (B-F) measured in HCT-116^wt^ cells. Shown are the mean and the standard deviation (SD) of five independent experiments. The data were subjected to a two-way ANOVA followed by Dunnett’s post-hoc test, in which the percentage of cells treated with the different test substance concentrations at each time point was compared to the percentage of cells in the corresponding solvent control group (ctrl.; 0.1% DMSO); a: *p* ≤ 0.05; b: *p* ≤ 0.01; c: *p* ≤ 0.001; d: *p* ≤ 0.0001.

The potential antiproliferative effect of all IRA’s as well as resveratrol was firstly investigated in the colorectal carcinoma cell line HCT-116^wt^ using the SRB assay. As depicted in Fig 2A and 2C–2F, all tested compounds, with the exception of IRA 1 (Fig 2B), inhibited the growth of the HCT-116^wt^ cells more or less in a time- and dose-dependent manner. However, notable and strong effects were only exerted by resveratrol (Fig 2A) and IRA 5 (Fig 2F), the latter substance being already significantly toxic at a concentration of 1 μM. These outcomes are also reflected by the IC_50_ values shown in Table 1. While IRA’s 1–4 mostly exhibited either no computable or very high IC_50_ values (i.e. > 100 μM), IRA 5 inhibited the proliferation of HCT-116^wt^ cells by 50% at a concentration of approx. 0.6 μM after five days of incubation (Table 1). In contrast, approx. 50 times more resveratrol was needed in order to induce the same effect in those cells (Table 1).

**Table 1:** Absolute IC_50_ values determined using the SRB assay in HCT-116^wt^ cells treated with resveratrol and five IRA’s.

Test compound	Incubation time [h]	IC_50_ [μM]	95% CI [μM]	R^2^ of fit
resveratrol	48	129.8	111.4–151.1	0.9190
	72	42.5	38.2–47.2	0.9312
	120	31.0	29.8–32.2	0.9927
IRA 1	48	160.9	28.3–916.4	0.06029
	72	c. n. p.	c. n. p.	c. n. p.
	120	151.1	19.0–1203.0	-0.5587
IRA 2	48	c. n. p.	c. n. p.	c. n. p.
	72	22584.0	3.4–1.5 x 10^8^	0.07366
	120	303.0	157.4–583.3	0.6595
IRA 3	48	c. n. p.	c. n. p.	c. n. p.
	72	c. n. p.	c. n. p.	c. n. p.
	120	c. n. p.	c. n. p.	c. n. p.
IRA 4	48	58657.0	116.0–3.0 x 10^7^	0.2003
	72	c. n. p.	c. n. p.	c. n. p.
	120	1507.0	462.5–4910.0	0.6555
IRA 5	48	84.1	66.6–106.3	0.8314
	72	7.0	6.2–7.9	0.9605
	120	0.6	0.5–0.7	0.9663

**c. n. p.**: calculation not possible

Since IRA 5 was the sole compound significantly reducing the growth of HCT-116^wt^ cells, only the cytotoxic effects of this compound and resveratrol were further investigated in other cell lines originating from colorectal tumors (Caco-2, HCA-7, HCT-116^p53-/-^) as well as in epidermoid (A-431) and prostate carcinoma (LNCaP) cells. As in the case of the HCT-116^wt^ cells, both test compounds induced significant time- and dose-dependent growth-inhibitory effects in all additional cell lines used (Figures A-E in S1 File). However, in the case of IRA 5, the effects were far less pronounced in HCT-116^p53-/-^ cells, especially at low doses (Fig 2F and Figure D2 in S1 File). Interestingly, the IC_50_ value determined for IRA 5 in those cells after 120 h of incubation was approx. 27 times higher than in the isogenic cells carrying a wildtype p53 protein, whereas the p53 status did not have an influence on the IC_50_ values detected when both cell lines were treated with resveratrol (Tables 1 and 2). Regarding the other cell lines used, the effects of resveratrol were mostly similar to the ones observed in HCT-116^wt^ cells, whereas the toxic activity of IRA 5 was much less pronounced, especially in the case of HCA-7 and LNCaP cells (Figure C2 in S1 File and Figure E2 in S1 File). Again, the IC_50_ values shown in Table 2 reflect these findings, as IRA 5 is not being consistently more potent than resveratrol in the above-mentioned cell lines.

**Table 2:** Absolute IC_50_ values determined in different cell lines treated with resveratrol and IRA 5.

Cell line	Test compound	Incubation time [h]	IC_50_ [μM]	95% CI [μM]	R^2^ of fit
A-431	resveratrol	48	88.7	71.7–109.8	0.8570
		72	20.2	17.0–24.0	0.9036
		120	9.2	8.4–10.1	0.9636
	IRA 5	48	133.4	117.7–151.1	0.9480
		72	39.5	35.7–43.7	0.9482
		120	15.4	14.1–16.8	0.9742
Caco-2	resveratrol	48	186.0	155.2–222.9	0.9293
		72	52.4	48.5–56.7	0.9655
		120	16.1	15.2–17.0	0.9895
	IRA 5	48	348.7	244.9–496.5	0.8864
		72	46.1	41.4–51.3	0.9486
		120	13.4	11.9–15.0	0.9577
HCA-7	resveratrol	48	741.3	239.8–2292.0	0.5532
		72	149.1	122.6–181.4	0.8824
		120	33.8	30.0–38.1	0.9184
	IRA 5	48	288.6	135.9–612.8	0.4600
		72	206.7	139.2–306.9	0.7196
		120	51.6	47.3–56.3	0.9349
HCT-116^p53-/-^	resveratrol	48	149.1	125.1–177.7	0.9239
		72	71.8	65.7–78.4	0.9341
		120	28.6	26.2–31.2	0.9661
	IRA 5	48	134.2	110.3–163.3	0.8943
		72	57.6	49.8–66.6	0.9020
		120	16.1	14.2–18.3	0.9458
LNCaP	resveratrol	48	265.8	204.1–346.1	0.9017
		72	161.2	128.2–202.6	0.9093
		120	29.6	25.2–34.6	0.9064
	IRA 5	48	342.3	236.5–495.4	0.8721
		72	166.3	120.9–228.7	0.8588
		120	24.9	21.8–28.4	0.9296

### Cell membrane integrity

**Fig 3:**
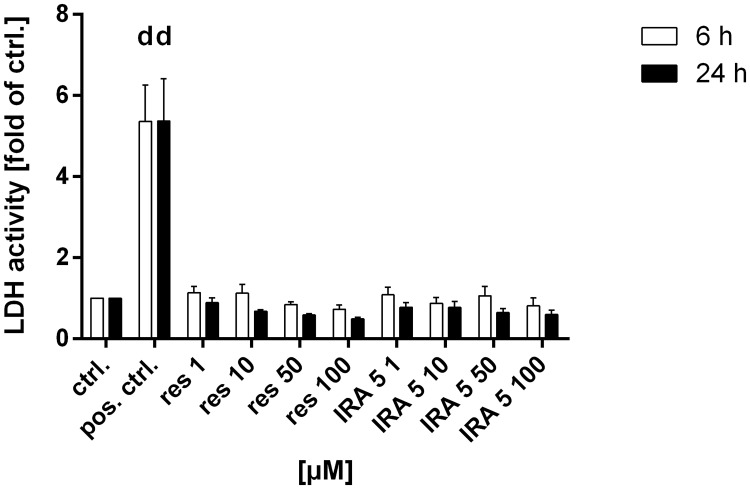
Measurement of the extracellular LDH activity in HCA-7 cells. The extracellular LDH activity is used as marker for the cell membrane integrity after treatment with resveratrol (res) and IRA 5 for 6 (white bars) and 24 h (black bars). Shown are the mean and the standard deviation (SD) of four independent experiments. The data were subjected to a two-way ANOVA followed by Dunnett’s post-hoc test, comparing all groups of each point in time to the solvent control (ctrl.; 0.1% DMSO). Only treatment with the positive control (pos. ctrl.; 0.18% Triton X-100; Promega) leads to a statistically significant increase in LDH activity. a: *p* ≤ 0.05; b: *p* ≤ 0.01; c: *p* ≤ 0.001; d: *p* ≤ 0.0001.

As shown in Fig 3, neither treatment with resveratrol nor IRA 5 led to a significant disturbance of the cell membrane integrity (i.e. an increased activity of the extracellular LDH in the culture supernatant) in HCA-7 cells after 6 or 24 h.

### Cell cycle analysis

**Fig 4:**
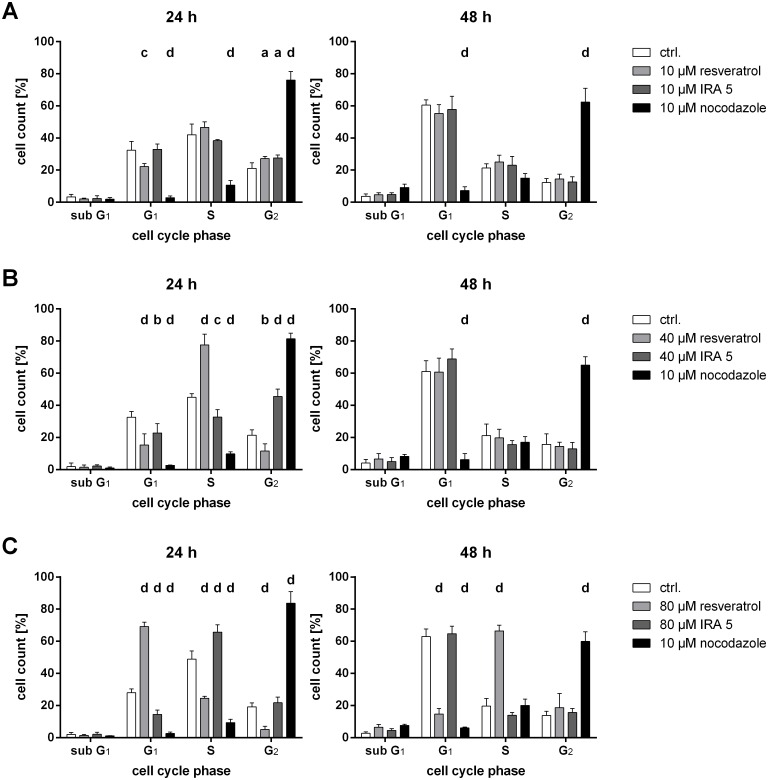
Effect of resveratrol and IRA 5 on the cell cycle distribution of HCT-116^wt^ cells. The effect of 10 μM resveratrol and IRA 5 (**A**), 40 μM resveratrol and IRA 5 (**B**) and 80 μM resveratrol and IRA 5 (**C**) was measured after 24 and 48 h of incubation. Shown are the mean and SD of four independent experiments. The data were subjected to a two-way ANOVA followed by Dunnett’s post-hoc test, comparing the fraction of resveratrol and IRA 5-treated cells with the fraction of solvent control-treated cells (ctrl.; 0.1% DMSO) in each cell cycle phase separately; a: *p* ≤ 0.05; b: *p* ≤ 0.01; c: *p* ≤ 0.001; d: *p* ≤ 0.0001.

In HCT-116^wt^ cells, both test compounds were only able to induce significant effects on the cell cycle at a concentration of ≥ 40 μM and only after a 24-h incubation (Fig 4A and 4B). While the treatment with resveratrol led to a significant accumulation of cells in the S phase, IRA 5 induced a G_2_ phase arrest (Fig 4B). Interestingly, after 24 h of incubation, 80 μM resveratrol led to a G_1_ phase accumulation which shifted to an S phase arrest after 48 h (Fig 4C), whereas IRA 5 at the same concentration merely induced an S phase arrest after a one-day treatment (Fig 4C).

In contrast, resveratrol induced a significant arrest of HCT-116^p53-/-^ cells in the S phase starting at 10 μM after 24 h of incubation, leading to a final accumulation in the sub G_1_ phase after 48 h and a 40 μM treatment (Table 3 and Figure I1 in S1 File as well as Figure I2 in S1 File). While IRA 5 at 40 μM also induced an S phase arrest after 24 h of incubation, it did not induce a sub G_1_ accumulation but rather a persisting S phase arrest until the highest incubation time of 48 hours was reached (Table 3 and Figure I2 in S1 File). Interestingly, at the highest concentration used (80 μM), both compounds induced transient accumulations over time (resveratrol 24 h: G_1_ phase arrest; resveratrol 48 h: S phase arrest; IRA 5 24 h: S phase arrest; IRA 5 48 h: G_2_ phase arrest), with only resveratrol leading to a time-dependent increase of cells in the sub G_1_ phase (Table 3 and Figure I3 in S1 File). Furthermore, as shown in Table 3, treatment with both polyphenols also led to differing concentration- and time-dependent effects on the cell cycle of the other cell lines used (a graphical representation of these data can be found in Figures F-H in S1 File as well as Figure J in S1 File). In general, the effects were more pronounced at higher concentrations (40 and 80 μM), occasional G_1_ and S phase arrests being nevertheless induced by resveratrol in A-431 as well as in LNCaP cells and by IRA 5 in HCA-7 and LNCaP cells at a concentration of 10 μM (Table 3). At 40 and 80 μM, with a few exceptions, the test compounds induced significant cell line-dependent cell cycle accumulations in the G_1_ (e.g. HCA-7 and LNCaP cells) as well as in the S phase (e.g. A-431 and Caco-2 cells; Table 3). Moreover, both compounds also induced a significant increase of the cellular fraction in the sub G_1_ phase in almost all cell lines at high concentrations, whereby A-431 cells were only affected by resveratrol and LNCaP cells by neither test substance (Table 3). Notable is also the fact that in the cell lines other than HCT-116 (both isotypes), the induced cell cycle arrests seem to be similarly transient, since, at the same concentration, they are either not present anymore after a 48-h incubation or shift to another phase (e.g. 80 μM IRA 5 in Caco-2 and HCA-7 cells; Table 3).

**Table 3:** The effect of resveratrol (res) and IRA 5 on the cell cycle distribution of different cell lines.

Cell line	Incubation time [h]	Test compound [μM]	Accumulation (↑)^1^/decrease (↓) of cellular fraction [significance]*			
			Sub G_1_	G_1_	S	G_2_/M
A-431	24	res [10]	-	**↑ [a]**	-	-
		IRA 5 [10]	-	-	-	-
	48	res [10]	-	-	-	-
		IRA 5 [10]	-	-	-	-
	24	res [40]	-	↓ [c]	-	**↑ [a]**
		IRA 5 [40]	-	↓ [d]	**↑ [d]**	-
	48	res [40]	**↑ [a]**	-	-	-
		IRA 5 [40]	-	-	-	-
	24	res [80]	**↑ [c]**	↓ [d]	**↑ [d]**	-
		IRA 5 [80]	-	↓ [d]	**↑ [d]**	-
	48	res [80]	**↑ [b]**	↓ [d]	**↑ [b]**	-
		IRA 5 [80]	-	↓ [d]	**↑ [d]**	-
Caco-2	24	res [10]	-	-	-	-
		IRA 5 [10]	-	-	-	-
	48	res [10]	-	-	-	-
		IRA 5 [10]	-	-	-	-
	24	res [40]	-	-	**↑ [d]**	↓ [d]
		IRA 5 [40]	-	↓ [d]	**↑ [d]**	-
	48	res [40]	**↑ [c]**	↓ [d]	**↑ [d]**	↓ [d]
		IRA 5 [40]	**↑ [c]**	↓ [d]	**↑ [c]**	-
	24	res [80]	**↑ [b]**	-	-	↓ [c]
		IRA 5 [80]	-	**↑ [c]**	-	↓ [d]
	48	res [80]	**↑ [d]**	-	-	↓ [d]
		IRA 5 [80]	**↑ [d]**	↓ [d]	**↑ [d]**	↓ [c]
HCA-7	24	res [10]	-	-	-	-
		IRA 5 [10]	-	**↑ [d]**	-	↓ [a]
	48	res [10]	-	**↑ [a]**	-	↓ [a]
		IRA 5 [10]	-	-	**↑ [d]**	↓ [b]
	24	res [40]	-	**↑ [d]**	↓ [d]	-
		IRA 5 [40]	-	**↑ [d]**	↓ [b]	-
	48	res [40]	-	↓ [b]	**↑ [d]**	↓ [d]
		IRA 5 [40]	-	**↑ [c]**	-	↓ [c]
	24	res [80]	-	**↑ [d]**	↓ [d]	-
		IRA 5 [80]	-	**↑ [d]**	↓ [d]	-
	48	res [80]	**↑ [c]**	**↑ [d]**	↓ [d]	↓ [d]
		IRA 5 [80]	**↑ [c]**	↓ [d]	**↑ [d]**	↓ [d]
HCT-116^p53-/-^	24	res [10]	-	↓ [a]	**↑ [b]**	-
		IRA 5 [10]	-	-	-	-
	48	res [10]	-	-	**↑ [a]**	-
		IRA 5 [10]	-	-	-	-
	24	res [40]	-	↓ [d]	**↑ [d]**	↓ [d]
		IRA 5 [40]	-	↓ [d]	**↑ [d]**	-
	48	res [40]	**↑ [b]**	↓ [b]	-	-
		IRA 5 [40]	-	↓ [b]	**↑ [a]**	-
	24	res [80]	**↑ [b]**	**↑ [d]**	↓ [d]	↓ [d]
		IRA 5 [80]	-	↓ [d]	**↑ [d]**	↓ [d]
	48	res [80]	**↑ [c]**	↓ [d]	**↑ [d]**	-
		IRA 5 [80]	-	↓ [b]	-	**↑ [b]**
LNCaP	24	res [10]	-	**↑ [b]**	↓ [b]	-
		IRA 5 [10]	-	-	↓ [c]	-
	48	res [10]	-	**↑ [d]**	↓ [d]	-
		IRA 5 [10]	-	**↑ [d]**	↓ [d]	-
	24	res [40]	-	**↑ [b]**	-	-
		IRA 5 [40]	-	-	-	-
	48	res [40]	-	**↑ [d]**	↓ [d]	↓ [a]
		IRA 5 [40]	-	**↑ [d]**	↓ [d]	-
	24	res [80]	-	-	-	-
		IRA 5 [80]	-	↓ [a]	-	-
	48	res [80]	-	**↑ [d]**	↓ [c]	↓ [b]
		IRA 5 [80]	-	**↑ [d]**	↓ [b]	↓ [a]

^**1**^ An accumulation of cells is marked in bold lettering

* Two-way ANOVA followed by Dunnett’s post-hoc test, comparing the fraction of resveratrol (res)- and IRA 5-treated cells with the fraction of solvent control-treated cells (0.1% DMSO) in each cell cycle phase separately;

**a**: *p* ≤ 0.05;

**b**: *p* ≤ 0.01;

**c**: *p* ≤ 0.001;

**d**: *p* ≤ 0.0001

### COX-2 expression and activity

**Fig 5:**
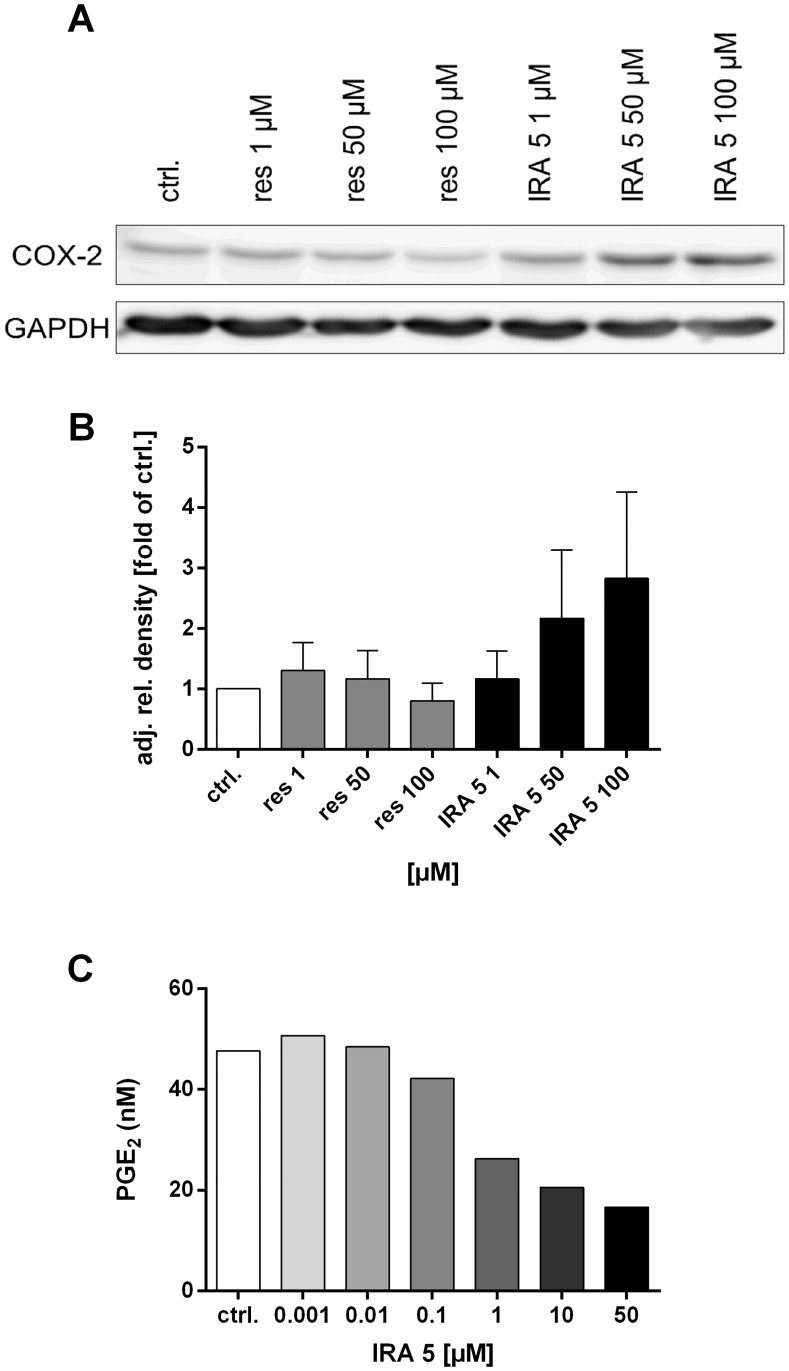
Effect of resveratrol and IRA 5 on the expression and activity of the COX-2 in HCA-7 cells. **A:** Representative western blot out of a total of three independently performed blotting experiments depicting the effect of resveratrol (res) and IRA 5 on the expression of the COX-2 (ctrl. = 0.1% DMSO). **B:** Densitometric analysis of the three western blots mentioned under point **A** (ctrl. = 0.1% DMSO; res = resveratrol). The analysis was performed using ImageJ (v. 1.48v) and depicted is the mean (± SD) adjusted relative band density (i.e. density of the COX-2 bands corrected for protein loading inconsistencies using the intensity of the GAPDH bands). **C:** The effect of ascending IRA 5 concentrations on the activity (i.e. PGE_2_ production) of the COX-2. These data were not subjected to a statistical analysis due to the low number of actual experiments performed (n = 2, thus no SD is shown).

As depicted in Fig 5A and 5B, resveratrol did not greatly influence COX-2 expression in HCA-7 cells at concentrations of up to 50 μM, while slightly lowering the expression of that protein at 100 μM. In contrast, IRA 5 induced the opposite, i.e. an increase in the expression of COX-2 (Fig 5A and 5B). Figures depicting the developed membrane fragments and the developed combined membranes of all three independently conducted western blot experiments are shown in Figures K-P in S1 File.

In contrast to the protein expression data, the activity of COX-2 (determined as the amount of PGE_2_ released into the culture medium after an incubation with arachidonic acid) was dose-dependently reduced by IRA 5 in HCA-7 cells starting at a concentration of 1 μM, while doses below that value (i.e. 0.001–0.1 μM) did not greatly influence the PGE_2_ production (Fig 5C).

### Glucuronidation of IRA 5

**Fig 6:**
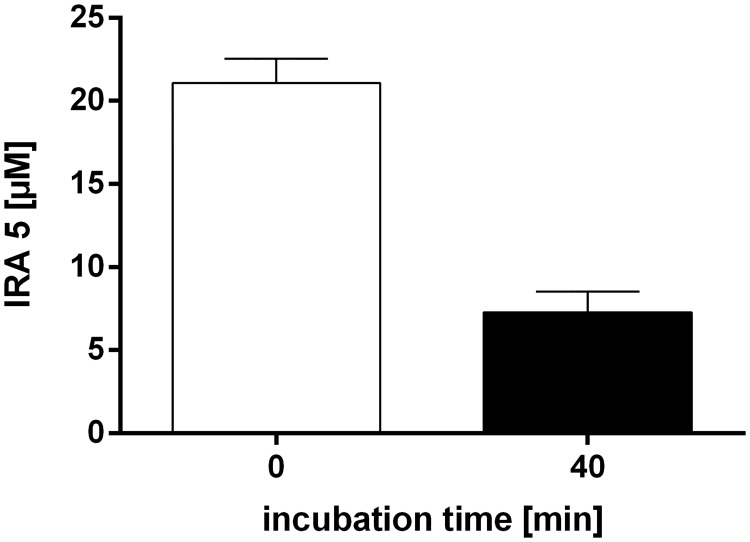
The amount of free IRA 5 found after a 40-minute incubation with RLM’s. Shown is the mean and SD of three independent incubations. These data were not subjected to a statistical analysis due to the low number of experiments performed.

After a 40-minute incubation with RLM’s, on average 7.3 ± 1.3 μM IRA 5 remained unconjugated when compared to the control incubation (Fig 6). This amounts to a mean glucuronidation rate of 65 ± 6.6%.

## Discussion

### Antiproliferative activity and cell membrane integrity

This is the first study in which the antiproliferative activity of IRA’s was investigated and compared to that of resveratrol in several human tumor cell lines. In order to identify the most potent potentially anticarcinogenic IRA, we first screened IRA’s 1–5 for the induction of a cytotoxic/antiproliferative effect in HCT-116^wt^ cells using the SRB assay. Of all five compounds tested, only IRA 5 showed a significant cytotoxic/antiproliferative activity, its IC_50_ value being approximately 50-fold lower than that of resveratrol. Consequently, only IRA 5 was chosen to be further characterized regarding its potential antitumor activity. Although this resveratrol analog inhibited the growth of HCT-116^wt^ cells in an unusually strong manner for a polyphenol, the effects were far less pronounced in the other five tumor cell lines used and therefore comparable or only slightly different to those exerted by resveratrol. The SRB assay data obtained herein using p53 knockout cells (HCT-116^p53-/-^) as well as cell lines with a mutated p53 protein (e.g. Caco-2 and HCA-7; reviewed in [38]) strongly suggest that this tumor suppressor protein is involved in the modulation rather than the actual induction of the antiproliferative effect of IRA 5, since the IC_50_ values obtained in the above-mentioned cell lines are far higher (e.g. 27 times higher in HCT-116^p53-/-^ cells) than those determined in HCT-116^wt^ cells carrying a functional p53 protein (reviewed in [39]). Although similar findings have been reported for resveratrol using the same cell lines [40], the possible involvement of other (tumor suppressor) proteins or drug/membrane transporters in these processes should be further investigated.

In contrast to IRA 5 or the other IRA’s, the antiproliferative effect of resveratrol on the tumor cell lines used in the present study is well-documented ([41–43] and reviewed in [10]). However, due to different assay systems used, they are not directly comparable in terms of potency and are not always in line with the results presented herein. For example, using a similar assay/calculation setup as we did, Fouad et al. [44] determined a (similar) IC_50_ value of 40 μM after 72 h of incubation in HCT-116^wt^ cells, while resveratrol was considerably more potent in Caco-2 cells in the present study (IC_50_ values of 52 versus 115 μM after 72 h; [44]). However, one can conclude that the extent and outcome of the antiproliferative effect induced by resveratrol is not p53-dependent, as the growth of both HCT-116 isotype cell lines is similarly impaired.

In general, the SRB assay is not well suited to investigate short-term toxicity (i.e. < 24 h of incubation time). In view of the additional analyses performed in HCA-7 cells (COX-2 expression and activity), we also opted to measure cell membrane integrity as an indicator of acute toxicity and as a mechanistic marker for the induction of necrosis (reviewed in [45]) which probably underlies the observed antiproliferative effects. However, the data clearly show that the treatment with resveratrol and IRA 5 does not entail a loss of cell membrane integrity, thus indicating that the antiproliferative effects seen in HCA-7 cells are not related to the activation of necrotic processes.

### Disturbance of the cell cycle

IRA 5 led to a clear concentration- and time-dependent change in the cell cycle distribution of all used cell lines. However, not all cell lines reacted in the same manner when incubated with this compound. Generally, very high and therefore irrelevant *in vivo* concentrations were needed to elicit an arrest in a certain cell cycle phase (mostly the G_1_ and S phase), HCT-116^wt^ cells being the most notable exception (G_2_ arrest already at a 10 μM concentration) however. Interestingly, in Caco-2, HCA-7 and HCT-116^p53-/-^ cells treated with 80 μM IRA 5, some of these arrests progressed from one phase to the next adjacent phase with increasing incubation time. The fact that those “phase shifts” were not the same across those cell lines (e.g. in Caco-2 and HCA-7 cells a shift from a G_1_ to an S phase arrest was observed, while in HCT-116^p53-/-^ cells a shift from an S to a G_2_ phase arrest occurred after 48 h) suggests that IRA 5 potentially interferes with different cell cycle control pathways in dependence of the genotype of the cell line used. This becomes particularly evident in HCT-116 cells, as IRA 5 induces different responses depending on whether p53 is present or knocked out. Moreover, in the case of some cell lines (e.g. A-431 and HCT-116^wt^ cells), an incubation with IRA 5 resulted in an arrest in a specific phase after 24 h, which did not persist after a two-day treatment. This phenomenon (i.e. a transitional, reversible or “disappearing” cell cycle accumulation) has been previously described in resveratrol-treated Caco-2 cells by Schneider et al. [46]. These authors suggested that such shifts might be explained by a metabolic or chemical degradation of resveratrol, and this might also apply to IRA 5. In addition to being arrested in the G_1_, S or G_2_ phase, some cell lines (A-431, Caco-2 and HCA-7) also displayed a significant accumulation of cells in the “sub G_1_” fraction after an IRA 5 treatment. This is indicative of apoptosis [47] and points out, together with the results of the membrane integrity assay performed in HCA-7 cells, that the antiproliferative effect of IRA 5, at least in the affected cell lines, first leads to a cell cycle arrest, which is then followed by the activation of apoptotic pathways. The latter statement (i.e. the induction of apoptosis) would need to be further confirmed by specific assays, which we did not perform due to the sub G_1_ peak only emerging at high and thus not relevant *in vivo* concentrations.

The effects of resveratrol on the cell cycle progression of different human tumor cell lines are well documented. However, as discussed in a previous publication using LNCaP cells [21], these effects often differ between different studies using the same cell line. For example, Ahmad et al. [41] reported that resveratrol at a concentration of 50 μM causes a G_1_ phase arrest after a one-day incubation in A-431 cells, while in the present study a similar concentration (40 μM) and incubation time (24 h) induced a G_2_/M phase arrest. Similarly, Kim et al. [43] mention a resveratrol-induced G_1_ arrest following a 24-h treatment with 100 μM in A-431 cells, which again differs from the results reported herein. Furthermore, the data presented herein suggest, in contrast to a study by Mahyar-Roemer et al. [48], that resveratrol-induced apoptosis is dependent on p53, since a statistically significant amount of cells in the sub G_1_ phase was only observed in the p53-knockout HCT-116 cells after treatment with 40 or 80 μM resveratrol. On the other hand though, results obtained by others in HCT-116^wt^ and Caco-2 cells [46, 49, 50] are broadly in line with the present findings.

All in all, even though IRA 5 has significant effects on the cell cycle, these mostly seem, as in the case of resveratrol, to appear at (very) high concentrations and to be strongly cell line-dependent, suggesting that the bioactivity of this compound might be limited regarding its cell cycle-disturbing potency and that it does affect more than one cell cycle-related pathway. As in the case of the SRB assay data, p53 seems to play a modulating but not decisive role on whether a cell cycle arrest is induced.

### Effects of IRA 5 on COX-2 expression and activity

COX-2 and its product PGE_2_ appear to play a significant role in cancer development (especially colorectal cancer [CRC]). Thus, inhibition of this enzyme with specific inhibitors (e.g. non-steroidal anti-inflammatory drugs [NSAID’s]) considerably reduces the incidence of CRC and other cancers (reviewed in [51] and [52]). Resveratrol and other phenolic compounds have been described as COX-2 inhibitors ([15, 53, 54] and reviewed in [47]). Based on these findings, we investigated the potential effects of IRA 5 on COX-2 expression and activity in the COX-2-overexpressing colorectal carcinoma cell line HCA-7 [55] with interesting results: Whereas 50 to 100 μM of IRA 5 induced COX-2 expression, 50 μM of that compound inhibited PGE_2_ production to a substantial degree. In contrast to previous studies showing that a reduced COX-2 activity correlates with a reduced expression in HCA-7 cells [56], this contradictory finding suggests that IRA 5 is on the one hand an inhibitor of the COX-2, while on the other hand it possibly activates/interferes with cellular cascades involved in COX-2 expression. One hypothetical explanation for the latter result (i.e. enhanced COX-2 expression) may be related to the capacity of IRA 5 to activate Nuclear factor (erythroid-derived 2)-like 2 (Nrf2) [27], a transcription factor conferring protection against oxidative stress (reviewed in [57]). Recent work has shown that the treatment with an Nrf2 activator induces COX-2 expression in vascular smooth muscle cells of the rat [58], a fact that could explain the increased expression of COX-2 observed in the present work. Conversely, the inhibition of PGE_2_ production by IRA 5 could be a first step in a cascade of events leading to the observed antiproliferative effects in HCA-7 cells, since PGE_2_ has been shown to be an inducer of proliferation in the latter [59] as well as in other colorectal tumor cell lines [60], and resveratrol has been shown to suppress this effect [60]. Although the data obtained in the membrane integrity assay suggest that the observed effects on COX-2 expression are not related to acute toxic processes, the above-mentioned hypotheses need to be confirmed with further experiments, taking into account that the quite multifaceted expression of COX-2 entails many interconnected cellular pathways, transcription factors and proteins (e.g. nuclear factor kappa B [NF-κB] and mitogen-activated protein kinases [MAPK’s]; reviewed in [54] and [61]; see [62] for details on COX-2 regulation in HCA-7 cells).

In contrast to IRA 5, the results obtained in the present study regarding the effect of resveratrol on COX-2 expression are quite consistent with previous studies using HCA-7 cells, in which no or only a very slight reduction in protein expression after a 24 h-treatment was observed [15, 63].

### Metabolism of IRA 5

Many studies investigating the potential anticarcinogenic effect of natural compounds *in vitro* often incubate the cells with very high (i.e. unrealistic) amounts of the test substance without taking into account that some phenolic compounds (e.g. resveratrol; reviewed in [14]) are prone to fast metabolization [64], or are, although not metabolized, not taken up by cells at all (e.g. the resveratrol oligomer hopeaphenol) [22]. Therefore, an analysis of the metabolism/metabolic stability of a compound should always be an integral part of the overall assessment of its biological or anticarcinogenic activity. In this context, the data from rat microsomal incubations shown in the present study demonstrate that IRA 5 is glucuronidated by RLM’s, although to a lesser extent than resveratrol in a similar assay system (approx. 65% vs > 99%; [37]).

## Conclusions

This is the first study investigating the anticarcinogenic activity/chemopreventive potential of five different resveratrol imine analogs on a panel of different human tumor cell lines. While four of these compounds did not significantly inhibit the growth in an initial cytotoxicity screen using HCT-116^wt^ colorectal carcinoma cells, IRA 5 proved to be unusually potent in this regard, with an IC_50_ value approximately 50 times lower than that of resveratrol. However, in other tumor cell lines, the growth-inhibitory effects where mostly similar to those of resveratrol. Moreover, a cell line-dependent cell cycle dysregulation followed by an activation of apoptosis in certain cell lines upon treatment with IRA 5 was observed, yet mostly at high concentrations. Based on the results obtained regarding the IRA 5-related effects on the expression and activity of COX-2, this polyphenol can be considered an inhibitor of this enzyme. Lastly, IRA 5 was shown to be metabolically more stable than resveratrol.

In conclusion, the data presented herein show that IRA 5 leads to cytotoxicity in human tumor cell lines, affects COX-2 expression and activity and is less glucuronidated than resveratrol. Therefore, as has been postulated for resveratrol in the past [17], IRA 5 and structurally similar IRA’s could serve as a starting point for the synthesis of agents with more favorable biological properties than resveratrol or other well-investigated polyphenols.

## Supporting Information

S1 File Document containing Figures A-P, including all western blot images. (PDF)

S2 File Document containing the raw data used to generate all the figures and tables in the present study. (PDF)
