# Supplementary material for: Growth-Inhibiting Activity of Resveratrol Imine Analogs on Tumor Cells In Vitro
Source: PLoS One. 2017 Jan 23;12(1):e0170502. doi: 10.1371/journal.pone.0170502 (PMC5256997; doi:10.1371/journal.pone.0170502)
Supplement: S1 File — (PDF) [file pone.0170502.s001.pdf]

S1 File to: "Growth-inhibiting activity of resveratrol imine analogs on tumor cells *in vitro"* by Shan Wang, Ina Willenberg, Michael Krohn, Tanja Hecker, Sven Meckelmann, Chang Li, Yuanjiang Pan, Nils Helge Schebb, Pablo Steinberg and Michael Telamon Empl

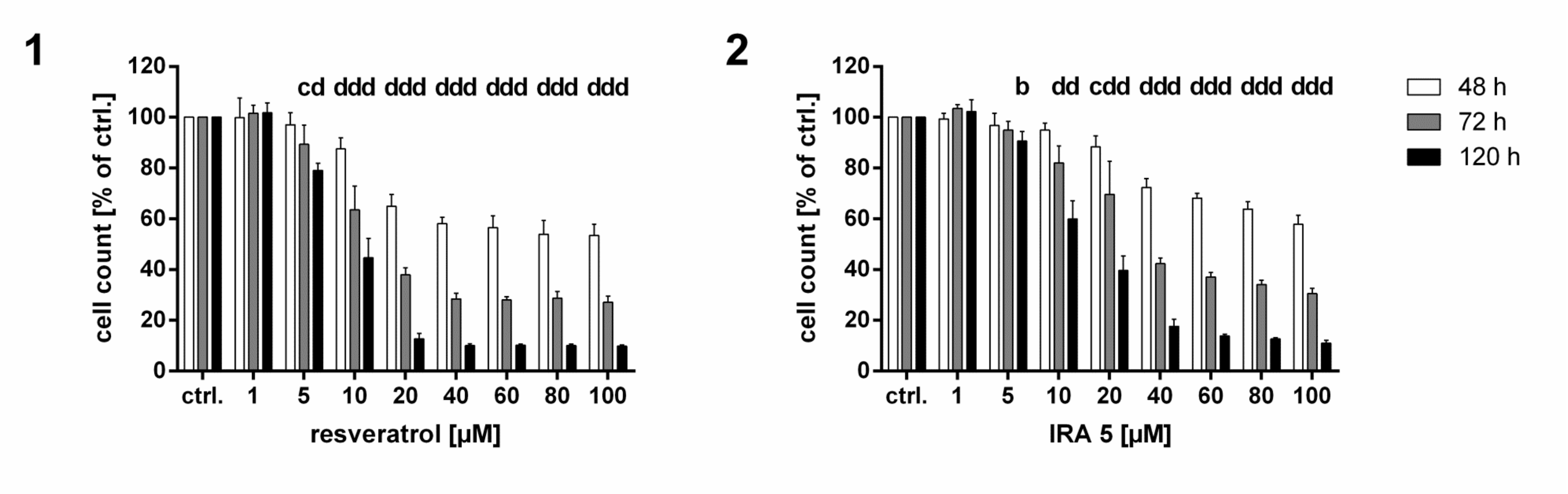

Figure A in S1 File: Growth-inhibitory effect of resveratrol (1) and IRA 5 (2) measured in A-431 cells. Shown are the mean and SD of five independent experiments. The data were subjected to a two-way ANOVA followed by Dunnett's post-hoc test, in which the percentage of cells treated with the different test substance concentrations at each time point was compared to the percentage of cells of the corresponding solvent control (ctrl.; 0.1 % DMSO); a: $p \leq 0.05$; b: $p \leq 0.01$; c: $p \leq 0.001$; d: $p \leq 0.0001$

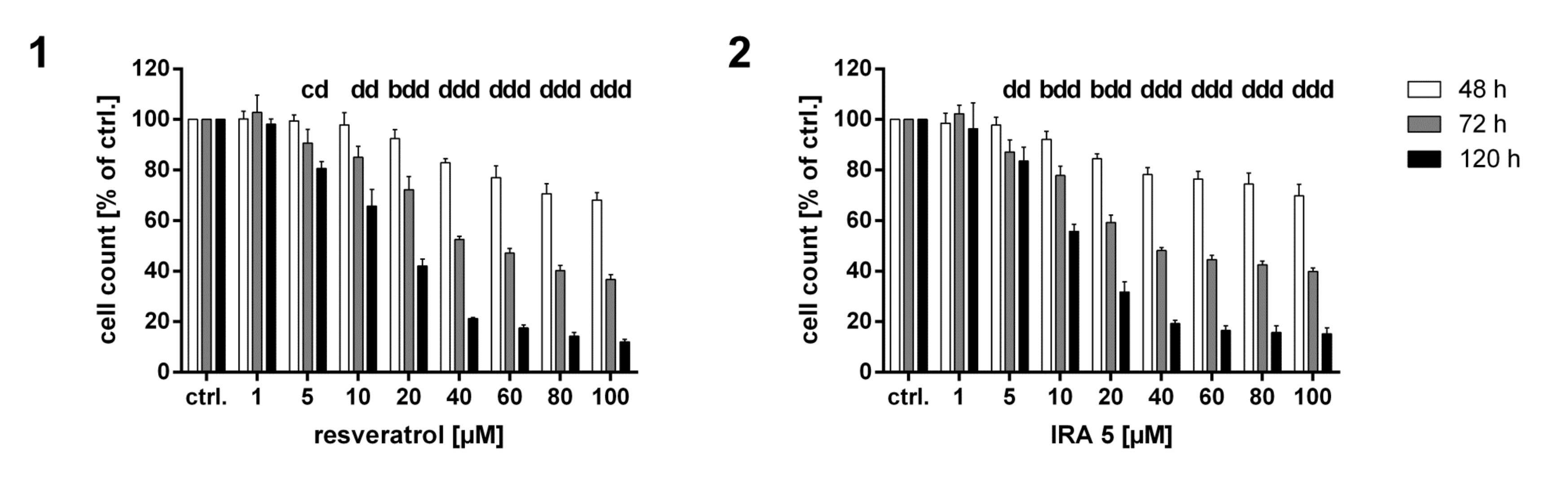

Figure B in S1 File: Growth-inhibitory effect of resveratrol (1) and IRA 5 (2) measured in Caco-2 cells. Shown are the mean and SD of five independent experiments. The data were subjected to a two-way ANOVA followed by Dunnett's post-hoc test, in which the percentage of cells treated with the different test substance concentrations at each time point was compared to the percentage of cells of the corresponding solvent control (ctrl.; 0.1 % DMSO); a: $p \leq 0.05$; b: $p \leq 0.01$; c: $p \leq 0.001$; d: $p \leq 0.0001$

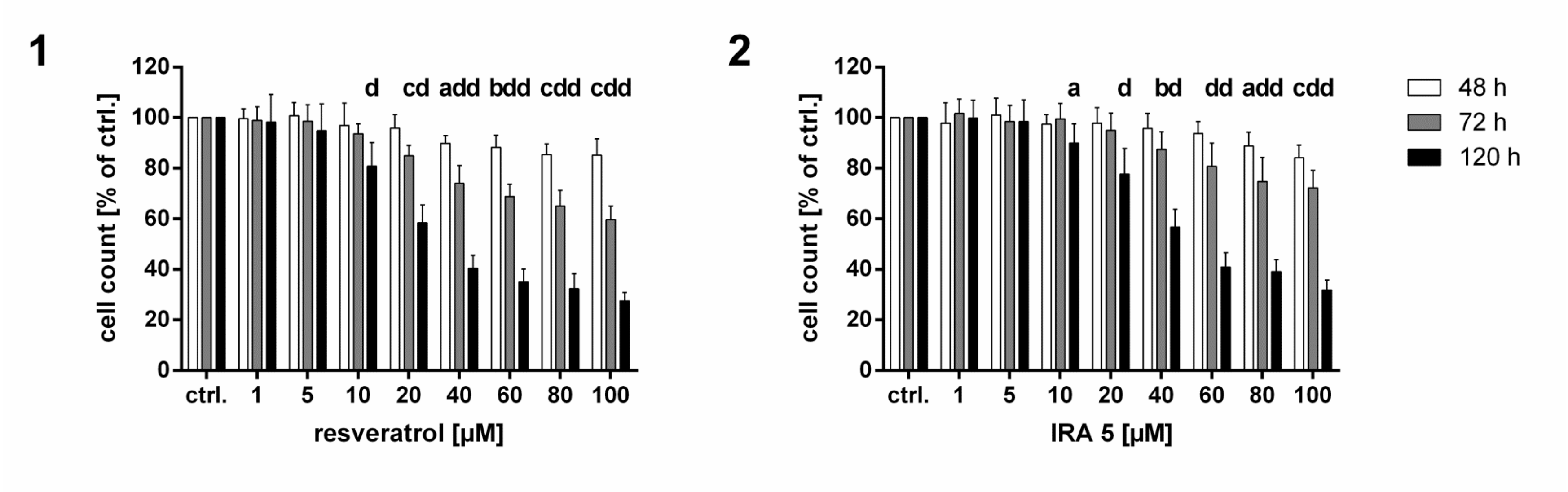

Figure C in S1 File: Growth-inhibitory effect of resveratrol (1) and IRA 5 (2) measured in HCA-7 cells. Shown are the mean and SD of six independent experiments. The data were subjected to a two-way ANOVA followed by Dunnett's post-hoc test, in which the percentage of cells treated with the different test substance concentrations at each time point was compared to the percentage of cells of the corresponding solvent control (ctrl.; 0.1 % DMSO); a: $p \leq 0.05$; b: $p \leq 0.01$; c: $p \leq 0.001$; d: $p \leq 0.0001$

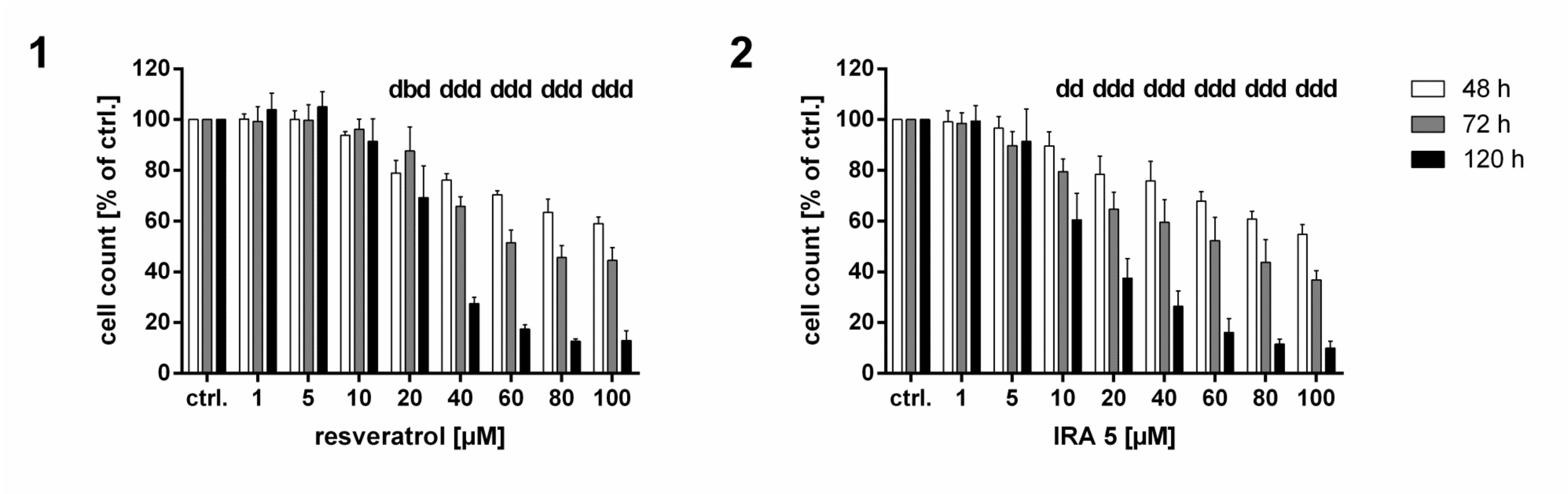

Figure D in S1 File: Growth-inhibitory effect of resveratrol (1) and IRA 5 (2) measured in HCT-116$^{p53-/-}$ cells. Shown are the mean and SD of five independent experiments. The data were subjected to a two-way ANOVA followed by Dunnett's post-hoc test, in which the percentage of cells treated with the different test substance concentrations at each time point was compared to the percentage of cells of the corresponding solvent control (ctrl.; 0.1 % DMSO); a: $p \leq 0.05$; b: $p \leq 0.01$; c: $p \leq 0.001$; d: $p \leq 0.0001$

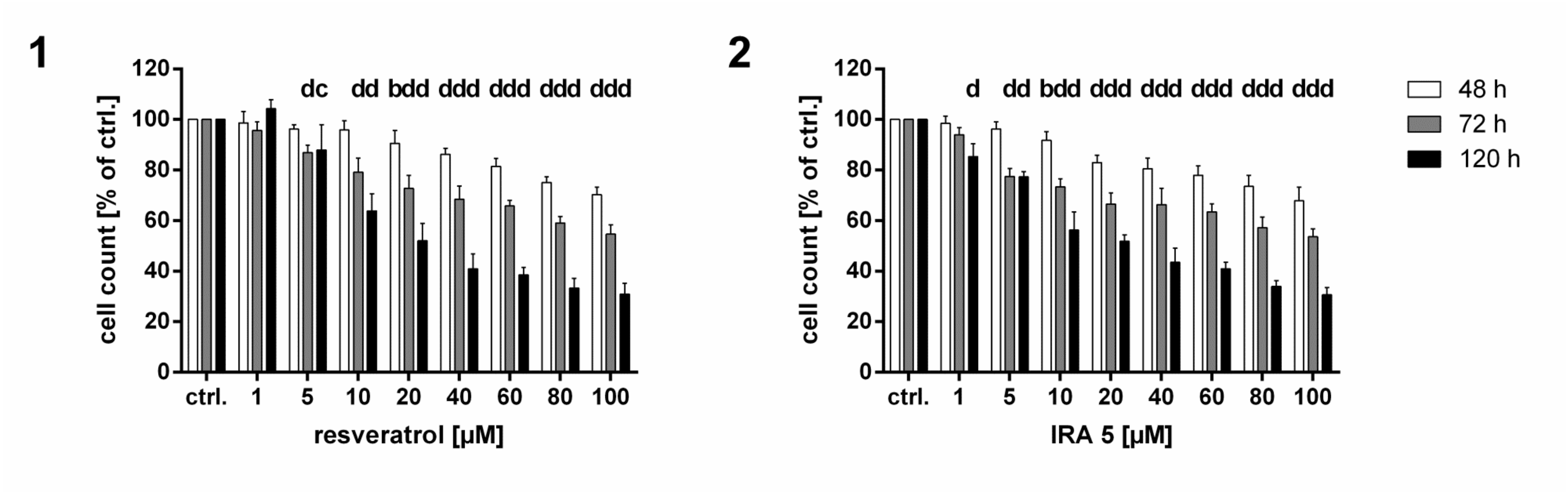

Figure E in S1 File: Growth-inhibitory effect of resveratrol (1) and IRA 5 (2) measured in LNCaP cells. Shown are the mean and SD of five independent experiments. The data were subjected to a two-way ANOVA followed by Dunnett's post-hoc test, in which the percentage of cells treated with the different test substance concentrations at each time point was compared to the percentage of cells of the corresponding solvent control (ctrl.; 0.1 % DMSO); a: $p \le 0.05$; b: $p \le 0.01$; c: $p \le 0.001$; d: $p \le 0.0001$

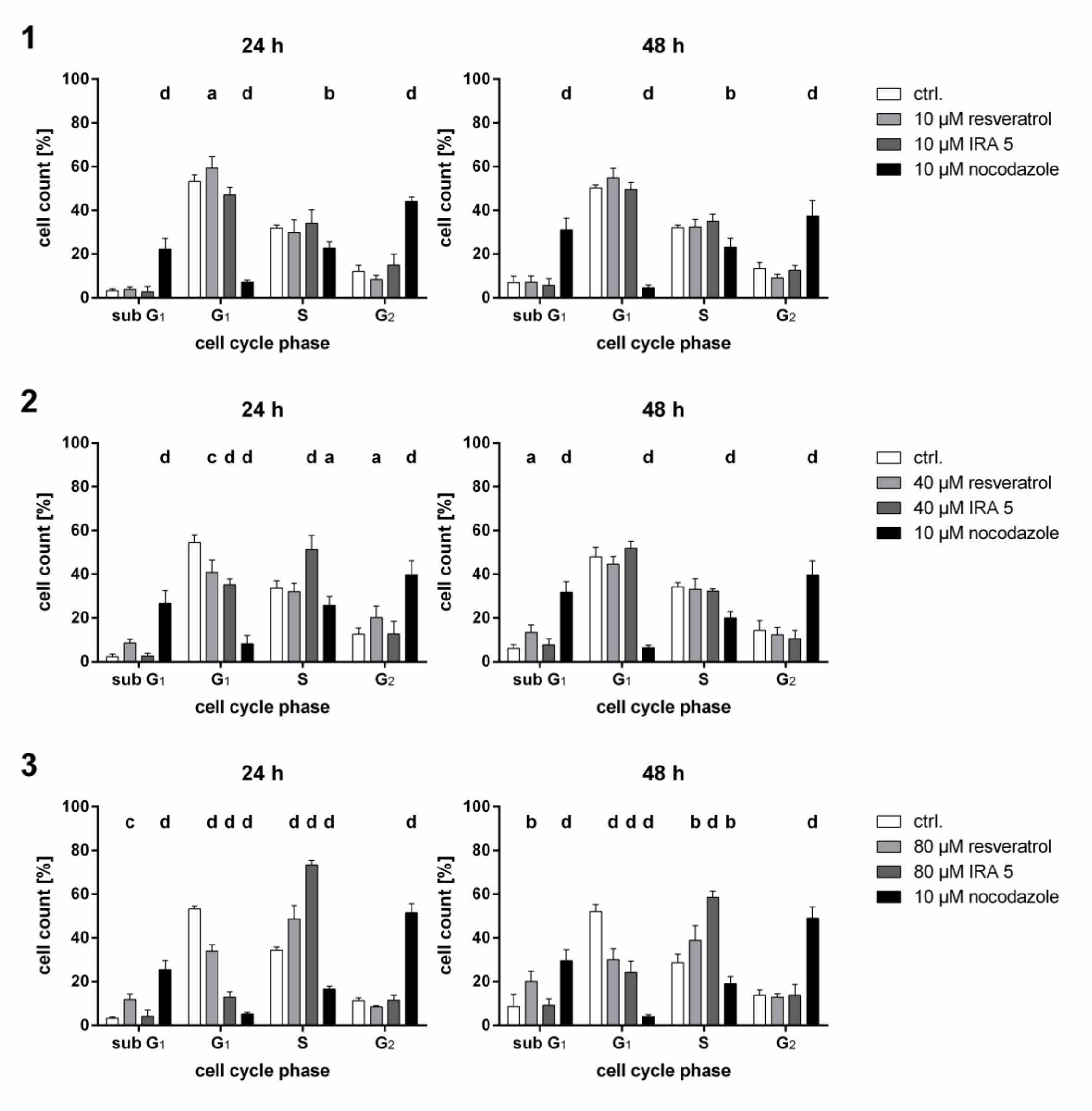

Figure F in S1 File: Effect of 10 (1), 40 (2) and 80 µM (3) resveratrol and IRA 5 on the cell cycle distribution of A-431 cells after 24 and 48 h of incubation. Shown are the mean and SD of four independent experiments. The data were subjected to a two-way ANOVA followed by Dunnett's post-hoc test, comparing the fraction of resveratrol- and RIA 5-treated cells with the fraction of solvent control-treated cells (ctrl.; 0.1 % DMSO) in each cell cycle phase separately; a: $p \leq 0.05$; b: $p \leq 0.01$; c: $p \leq 0.001$; d: $p \leq 0.0001$

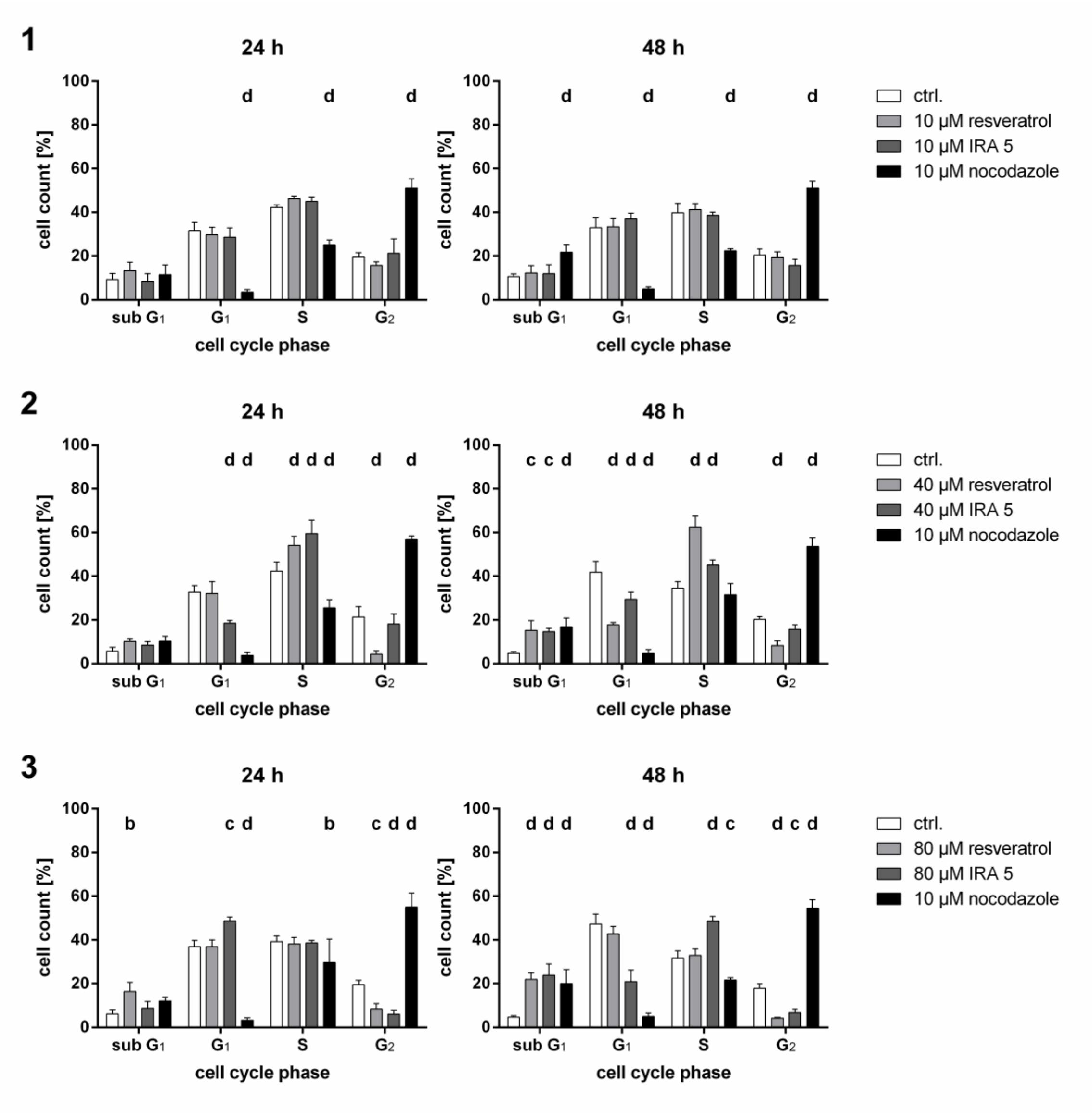

Figure G in S1 File: Effect of 10 (1), 40 (2) and 80 µM (3) resveratrol and IRA 5 on the cell cycle distribution of Caco-2 cells after 24 and 48 h of incubation. Shown are the mean and SD of four independent experiments. The data were subjected to a two-way ANOVA followed by Dunnett's post-hoc test, comparing the fraction of resveratrol- and RIA 5-treated cells with the fraction of solvent control-treated cells (ctrl.; 0.1 % DMSO) in each cell cycle phase separately; a: $p \leq 0.05$; b: $p \leq 0.01$; c: $p \leq 0.001$; d: $p \leq 0.0001$

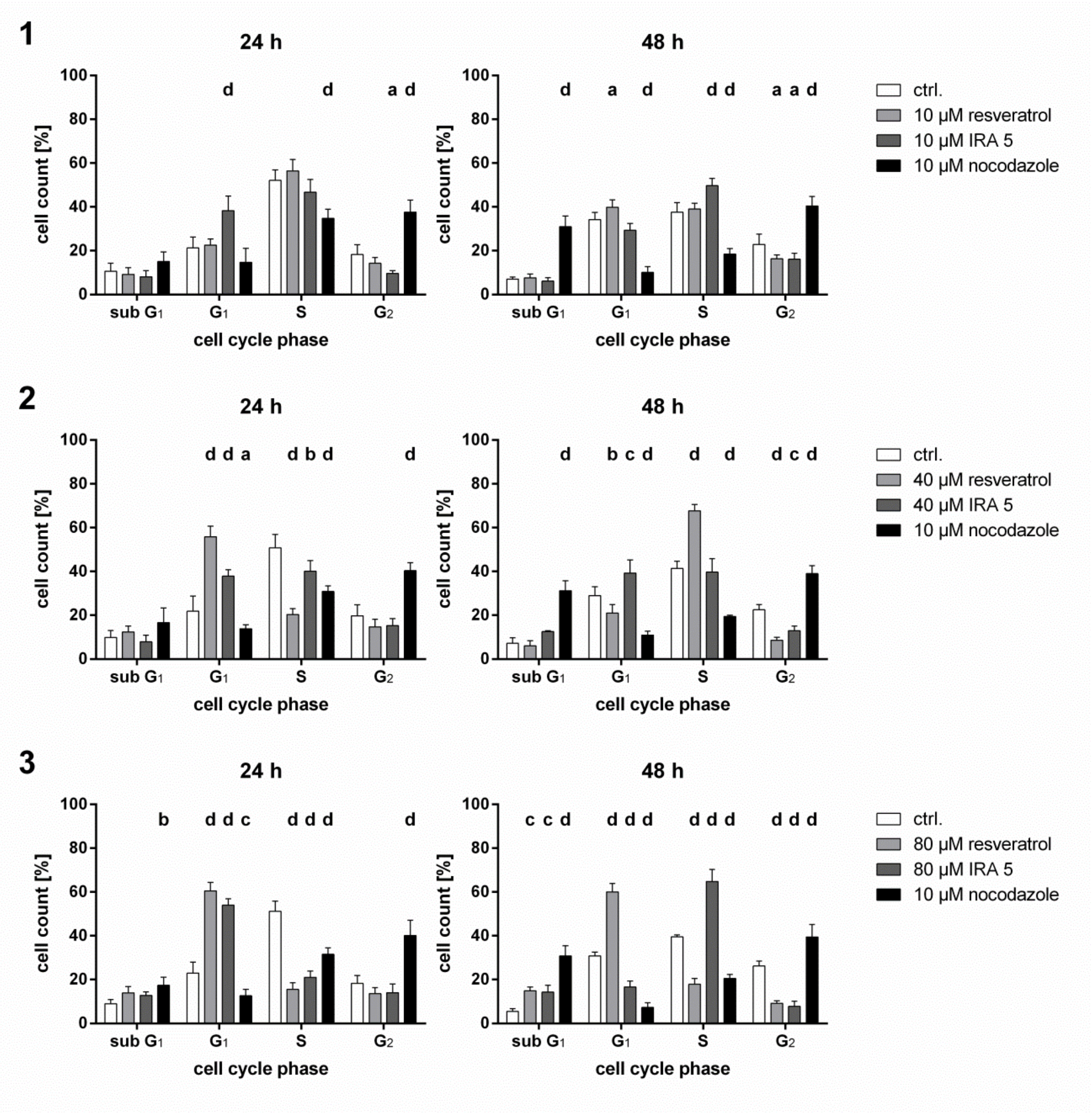

Figure H in S1 File: Effect of 10 (1), 40 (2) and 80 µM (3) resveratrol and IRA 5 on the cell cycle distribution of HCA-7 cells after 24 and 48 h of incubation. Shown are the mean and SD of four independent experiments. The data were subjected to a two-way ANOVA followed by Dunnett's post-hoc test, comparing the fraction of resveratrol- and RIA 5-treated cells with the fraction of solvent control-treated cells (ctrl.; 0.1 % DMSO) in each cell cycle phase separately; a: $p \leq 0.05$; b: $p \leq 0.01$; c: $p \leq 0.001$; d: $p \leq 0.0001$

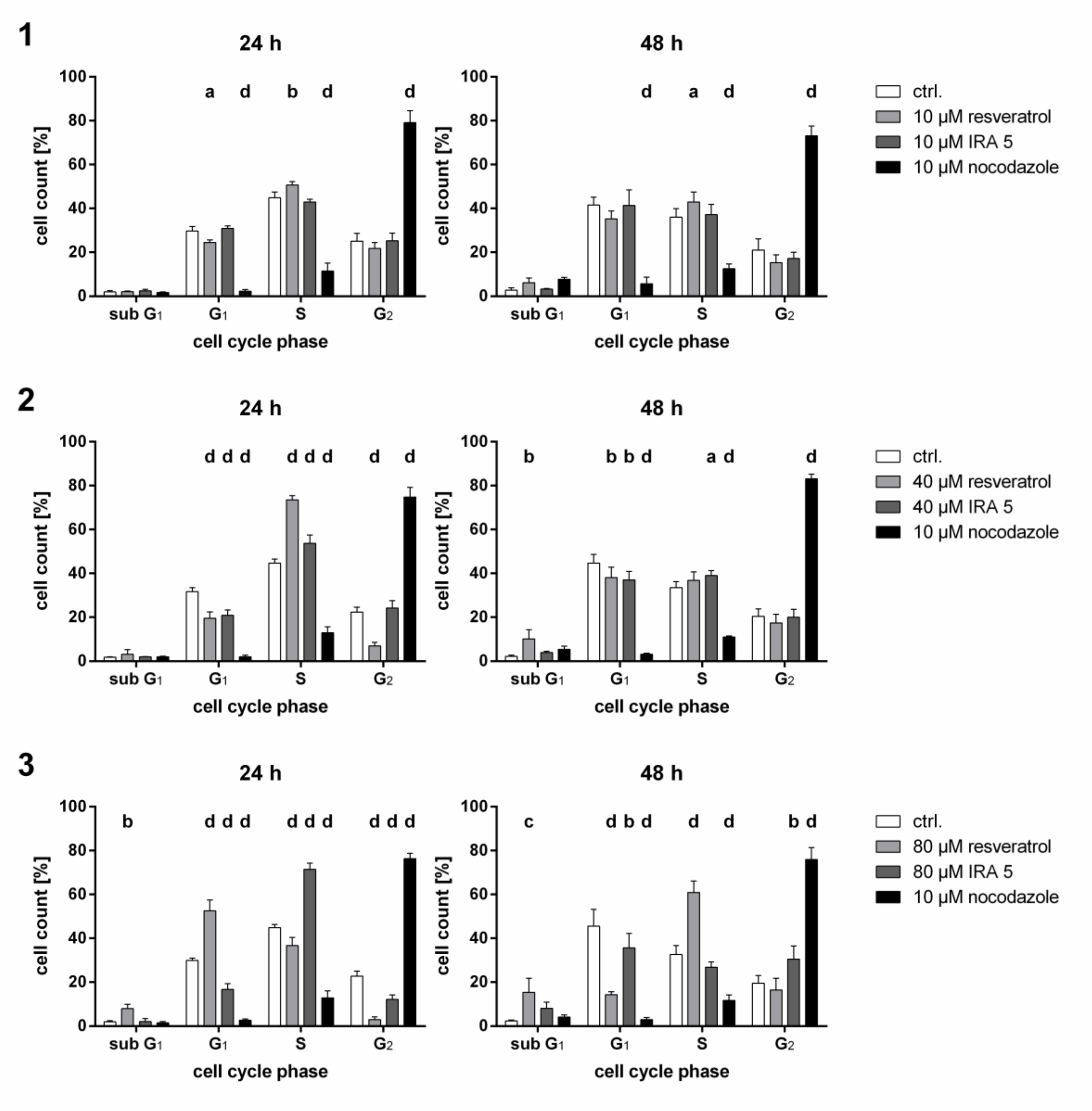

Figure I in S1 File: Effect of 10 (1), 40 (2) and 80 µM (3) resveratrol and IRA 5 on the cell cycle distribution of HCT-116$^{p53-/-}$ cells after 24 and 48 h of incubation. Shown are the mean and SD of four independent experiments. The data were subjected to a two-way ANOVA followed by Dunnett's post-hoc test, comparing the fraction of resveratrol- and RIA 5-treated cells with the fraction of solvent control-treated cells (ctrl.; 0.1 % DMSO) in each cell cycle phase separately; a: $p \leq 0.05$; b: $p \leq 0.01$; c: $p \leq 0.001$; d: $p \leq 0.0001$

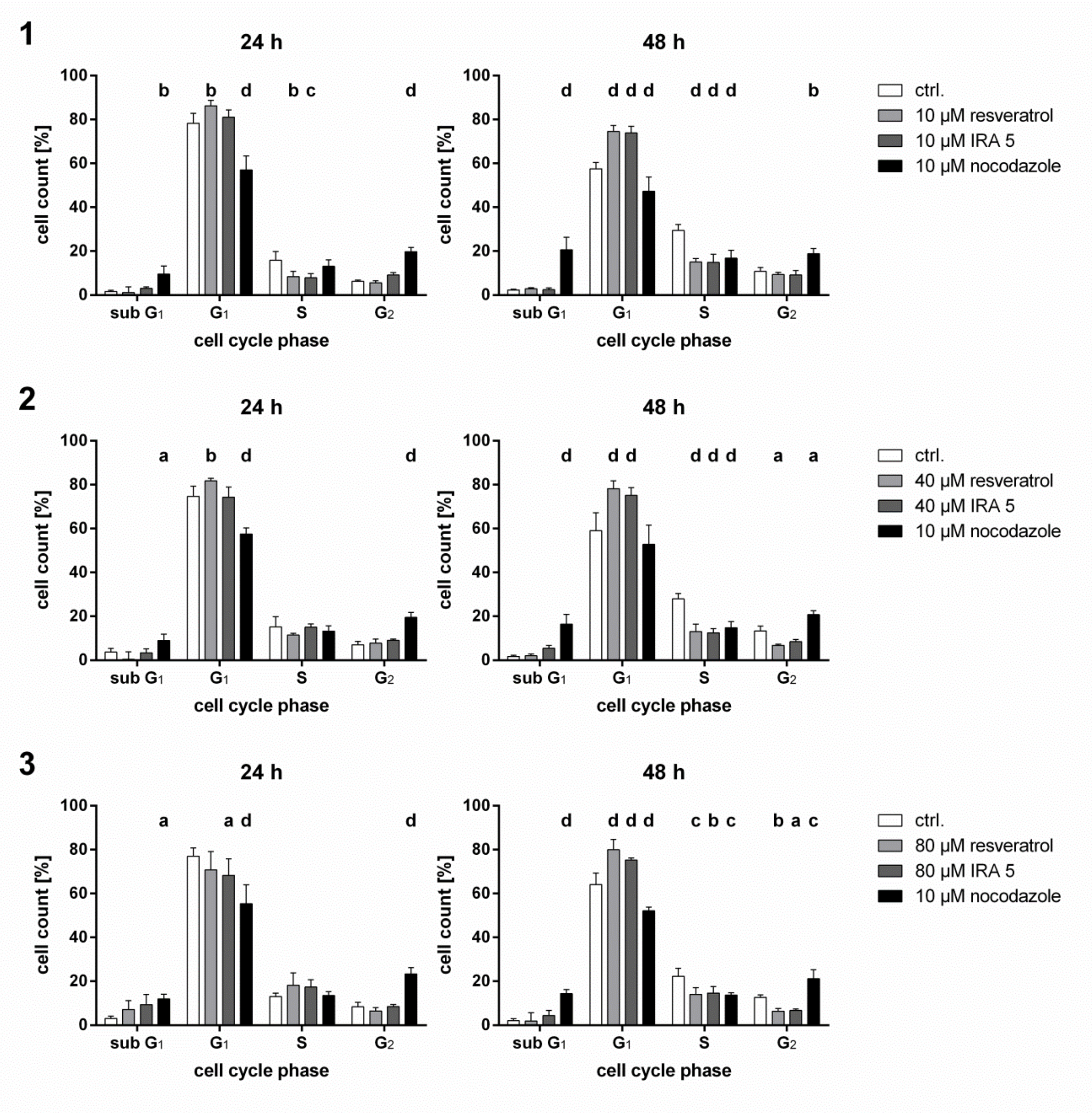

Figure J in S1 File: Effect of 10 (1), 40 (2) and 80 µM (3) resveratrol and IRA 5 on the cell cycle distribution of LNCaP cells after 24 and 48 h of incubation. Shown are the mean and SD of four independent experiments. The data were subjected to a two-way ANOVA followed by Dunnett's post-hoc test, comparing the fraction of resveratrol- and RIA 5-treated cells with the fraction of solvent control-treated cells (ctrl.; 0.1 % DMSO) in each cell cycle phase separately; a: $p \leq 0.05$; b: $p \leq 0.01$; c: $p \leq 0.001$; d: $p \leq 0.0001$

Figures K-M: Images of the cut western blot membrane pieces

After the blotting process, the nitrocellulose membranes were cut approximately at 55 kDa and the pieces were incubated with the respective antibodies for Cox-2 as well as GAPDH and developed separately. Depicted are images of three independent experiments recorded after 3 (**1**; COX-2) and 0.5 min (**2**; GAPDH) incubation with the ECL substrate.

Figure K: Experiment 1

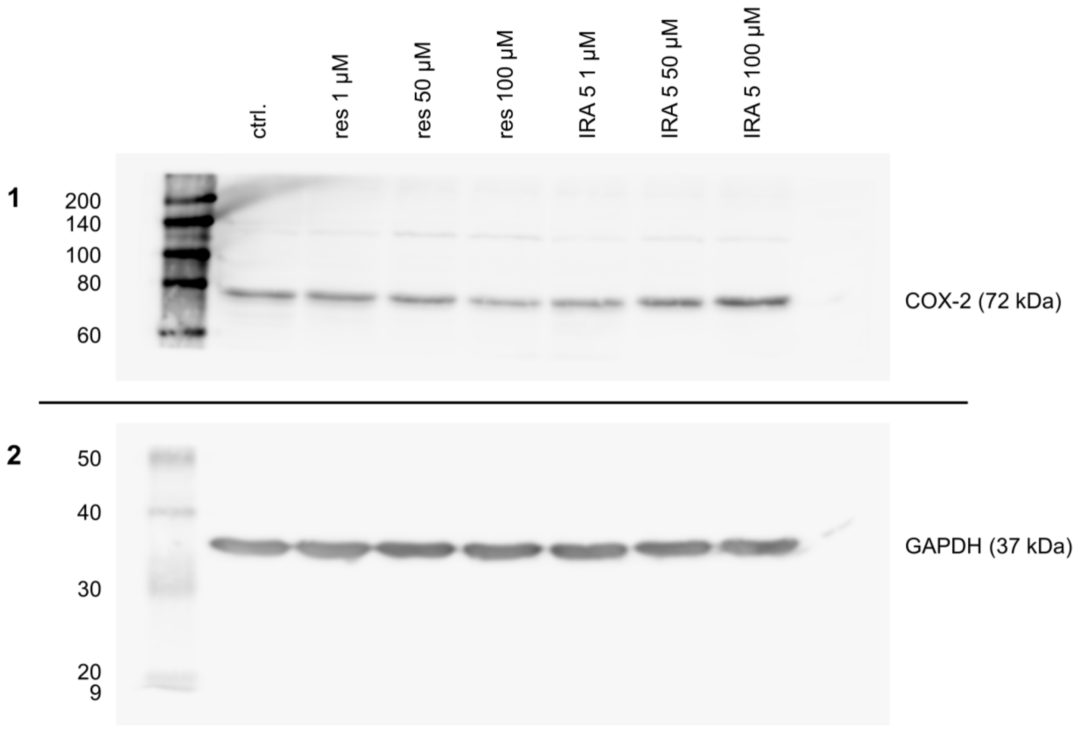

Ctrl.: 0.1 % DMSO; res.: resveratrol; The biotinylated protein ladder (Cell Signaling Technologies, Danvers, MA, USA; distributed by New England Biolabs, Frankfurt am Main, Germany) denotes the molecular mass in kDa

Figure L: Experiment 2

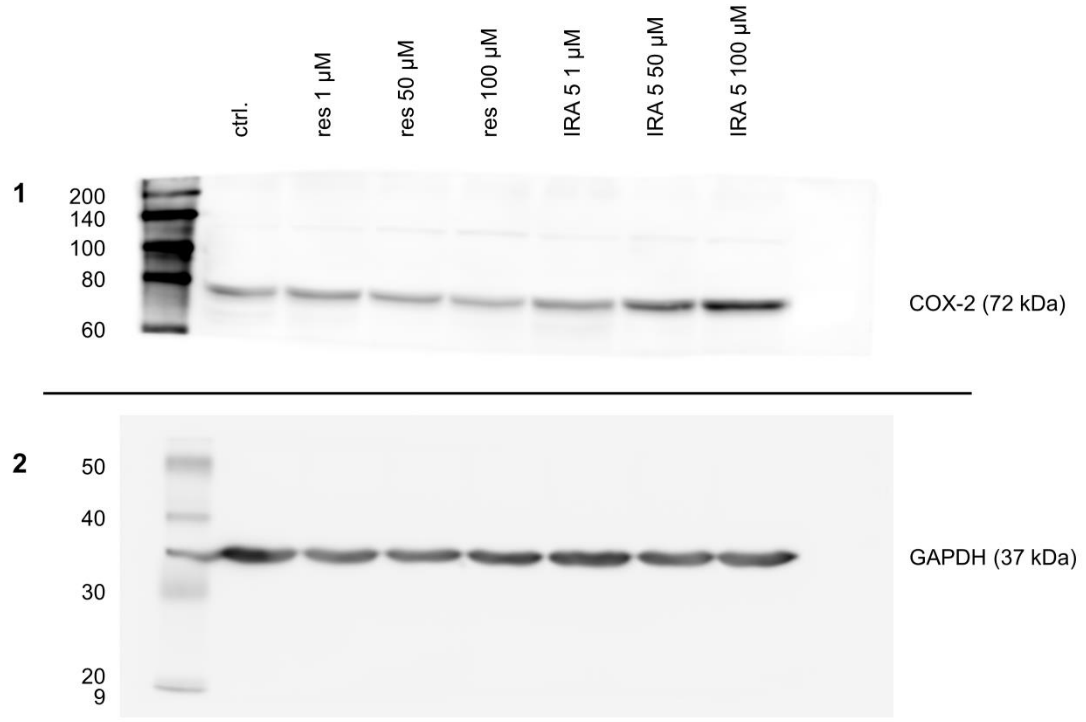

Ctrl.: 0.1 % DMSO; res.: resveratrol; The biotinylated protein ladder (Cell Signaling Technologies, Danvers, MA, USA; distributed by New England Biolabs, Frankfurt am Main, Germany) denotes the molecular mass in kDa

Figure M: Experiment 3

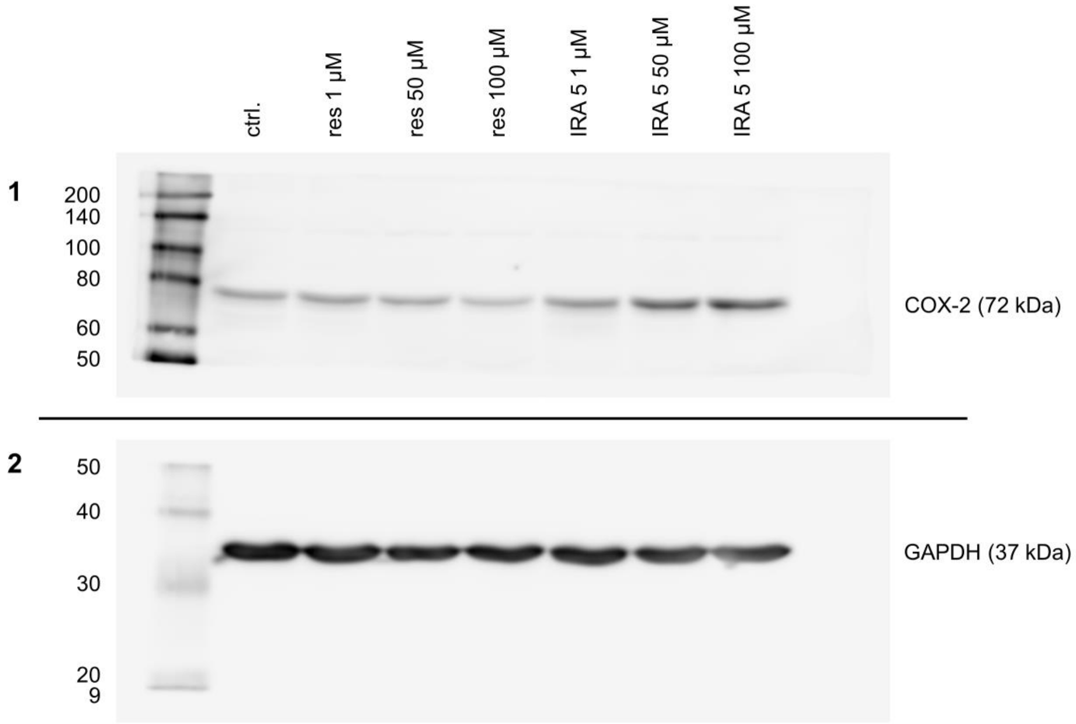

Ctrl.: 0.1 % DMSO; res.: resveratrol; The biotinylated protein ladder (Cell Signaling Technologies, Danvers, MA, USA; distributed by New England Biolabs, Frankfurt am Main, Germany) denotes the molecular mass in kDa

Figures N-P: Images of both western blot membranes combined

After the development (scan) of each membrane piece separately (COX-2 and GAPDH; see above), the fragments were combined using forceps and scanned again for 1 min without the addition of new ECL substrate.

Figure N: Experiment 1

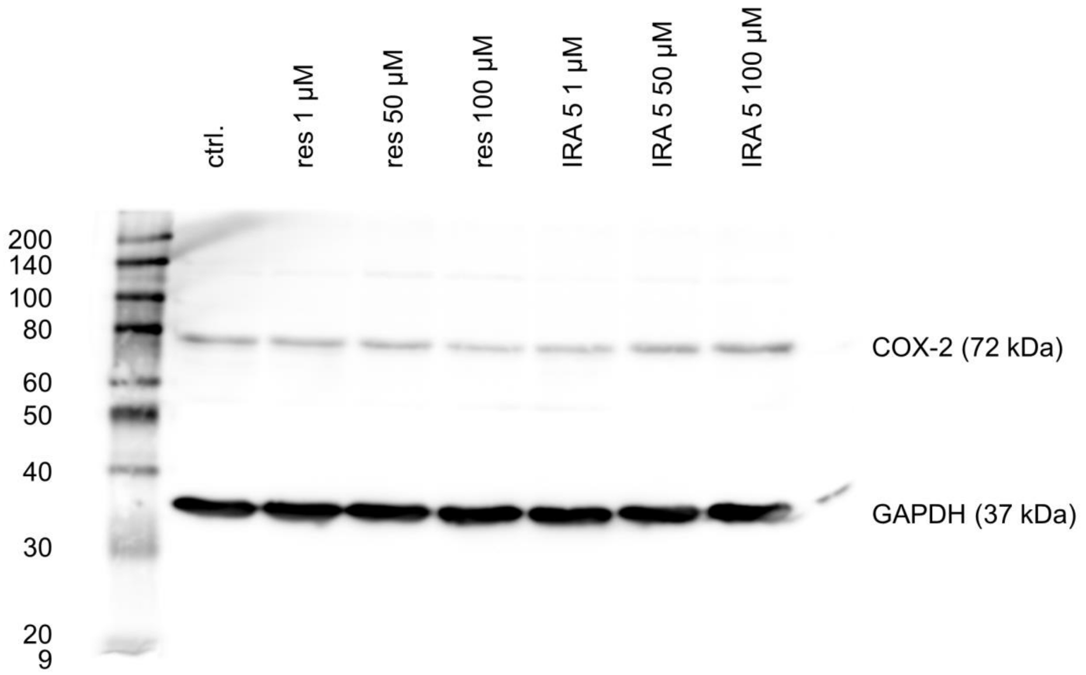

Ctrl.: 0.1 % DMSO; res.: resveratrol; The biotinylated protein ladder (Cell Signaling Technologies, Danvers, MA, USA; distributed by New England Biolabs, Frankfurt am Main, Germany) denotes the molecular mass in kDa

Figure O: Experiment 2

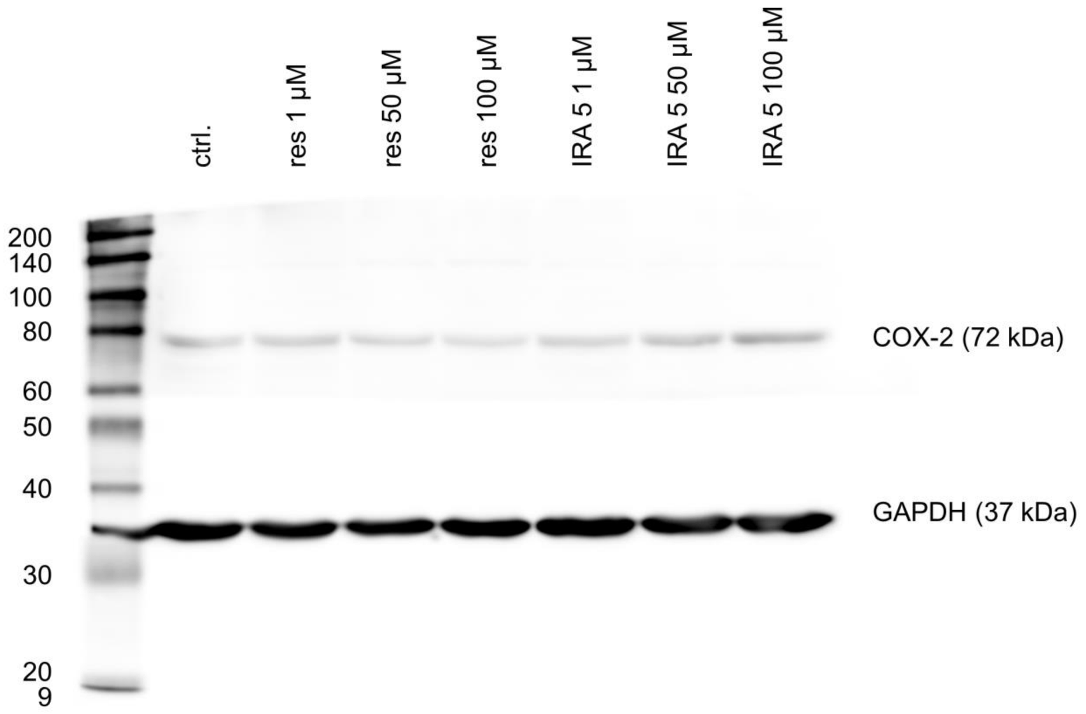

Ctrl.: 0.1 % DMSO; res.: resveratrol; The biotinylated protein ladder (Cell Signaling Technologies, Danvers, MA, USA; distributed by New England Biolabs, Frankfurt am Main, Germany) denotes the molecular mass in kDa

Figure P: Experiment 3

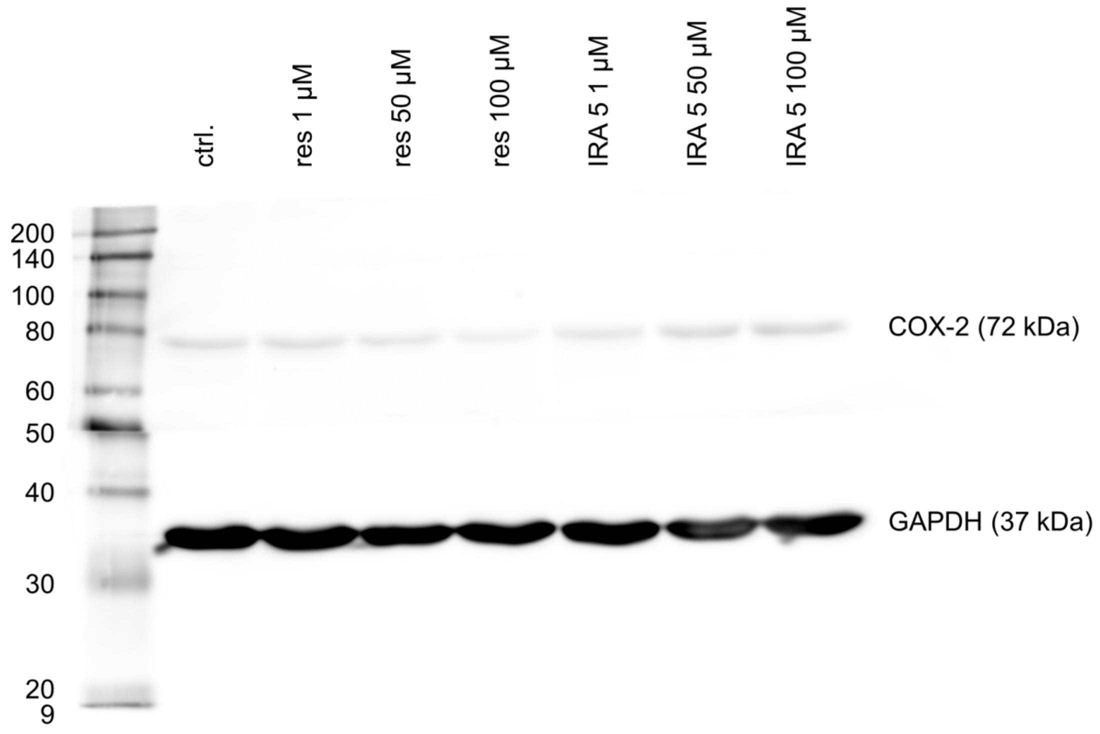

Ctrl.: 0.1 % DMSO; res.: resveratrol; The biotinylated protein ladder (Cell Signaling Technologies, Danvers, MA, USA; distributed by New England Biolabs, Frankfurt am Main, Germany) denotes the molecular mass in kDa
