# Supplementary material for: Growth-Inhibiting Activity of Resveratrol Imine Analogs on Tumor Cells In Vitro
Source: PLoS One. 2017 Jan 23;12(1):e0170502. doi: 10.1371/journal.pone.0170502 (PMC5256997; doi:10.1371/journal.pone.0170502)
Supplement: S2 File — (PDF) [file pone.0170502.s002.pdf]

S2 File to: "Growth-inhibiting activity of resveratrol imine analogs on tumor cells *in vitro"* by Shan Wang, Ina Willenberg, Michael Krohn, Tanja Hecker, Sven Meckelmann, Chang Li, Yuanjiang Pan, Nils Helge Schebb, Pablo Steinberg and Michael Telamon Empl

Raw data of the SRB assay

Abbreviations: ctrl.: solvent control (0.1 % DMSO); CV: coefficient of variation; DMSO: dimethyl sulfoxide; OD: optical density; REL: relative presentation of the data as percent of the solvent control; SD: standard deviation

HCT-116^{wt} cells

Resveratrol

Experiment 1

48 h incubation time

	ctrl.*	1 µM	5 µM	10 µM	20 µM	40 µM	60 µM	80 µM	100 µM
**OD well 1**	0.25	0.24	0.22	0.22	0.22	0.18	0.17	0.15	0.14
**OD well 2**	0.23	0.23	0.21	0.19	0.23	0.19	0.18	0.15	0.14
**OD well 3**	0.23	0.22	0.21	0.23	0.20	0.19	0.18	0.14	0.15
**MEAN**	0.24	0.23	0.22	0.22	0.21	0.19	0.18	0.15	0.15
**SD**	0.01	0.01	0.01	0.02	0.02	0.01	0.01	0.00	0.00
**REL [%]**	**100.0**	**97.5**	**92.0**	**91.8**	**91.1**	**79.4**	**74.6**	**62.8**	**61.8**
**CV [%]**	4.81	4.16	2.38	8.88	7.00	4.71	3.29	2.92	2.48

* 0.1 % DMSO

72 h incubation time

	ctrl.*	1 µM	5 µM	10 µM	20 µM	40 µM	60 µM	80 µM	100 µM
**OD well 1**	0.62	0.52	0.59	0.51	0.44	0.28	0.20	0.18	0.16
**OD well 2**	0.60	0.55	0.55	0.53	0.43	0.29	0.20	0.17	0.15
**OD well 3**	0.64	0.53	0.63	0.54	0.45	0.33	0.22	0.17	0.16
**MEAN**	0.62	0.53	0.59	0.53	0.44	0.30	0.21	0.17	0.15
**SD**	0.02	0.02	0.04	0.01	0.01	0.02	0.01	0.00	0.00
**REL [%]**	**100.0**	**86.6**	**95.2**	**85.5**	**71.5**	**48.8**	**34.1**	**28.1**	**25.0**
**CV [%]**	2.98	3.62	6.58	2.31	2.82	7.61	4.82	1.82	2.34

* 0.1 % DMSO

120 h incubation time

	ctrl.*	1 µM	5 µM	10 µM	20 µM	40 µM	60 µM	80 µM	100 µM
**OD well 1**	1.93	1.97	1.94	1.88	1.45	0.59	0.31	0.21	0.16
**OD well 2**	1.93	1.88	2.00	1.86	1.53	0.53	0.28	0.19	0.18
**OD well 3**	1.91	1.91	1.78	1.75	1.37	0.49	0.29	0.18	0.17
**MEAN**	1.92	1.92	1.91	1.83	1.45	0.54	0.29	0.19	0.17
**SD**	0.01	0.05	0.12	0.07	0.08	0.05	0.02	0.02	0.01
**REL [%]**	**100.0**	**99.8**	**99.2**	**95.2**	**75.5**	**27.9**	**15.2**	**10.0**	**8.7**
**CV [%]**	0.55	2.46	6.08	3.75	5.50	9.37	5.70	8.69	4.87

* 0.1 % DMSO

Experiment 2

48 h incubation time

	ctrl.*	1 µM	5 µM	10 µM	20 µM	40 µM	60 µM	80 µM	100 µM
**OD well 1**	0.22	0.24	0.24	0.25	0.22	0.19	0.17	0.17	0.17
**OD well 2**	0.23	0.24	0.26	0.24	0.23	0.18	0.16	0.15	0.14
**OD well 3**	0.24	0.25	0.24	0.24	0.20	0.16	0.15	0.13	0.16
**MEAN**	0.23	0.24	0.25	0.24	0.21	0.18	0.16	0.15	0.15
**SD**	0.01	0.00	0.01	0.01	0.02	0.02	0.01	0.02	0.01
**REL [%]**	**100.0**	**104.0**	**105.7**	**102.9**	**91.9**	**75.9**	**67.6**	**64.7**	**65.6**
**CV [%]**	4.03	1.22	3.76	3.12	8.74	8.61	7.62	13.08	9.16

* 0.1 % DMSO

72 h incubation time

	ctrl.*	1 µM	5 µM	10 µM	20 µM	40 µM	60 µM	80 µM	100 µM
**OD well 1**	0.69	0.70	0.73	0.73	0.59	0.35	0.24	0.20	0.18
**OD well 2**	0.64	0.70	0.78	0.65	0.53	0.33	0.24	0.19	0.19
**OD well 3**	0.62	0.70	0.78	0.71	0.54	0.35	0.23	0.19	0.17
**MEAN**	0.65	0.70	0.76	0.70	0.55	0.34	0.24	0.19	0.18
**SD**	0.04	0.00	0.03	0.04	0.03	0.01	0.00	0.01	0.01
**REL [%]**	**100.0**	**107.5**	**117.2**	**106.8**	**84.9**	**52.6**	**36.6**	**29.7**	**27.2**
**CV [%]**	5.42	0.30	3.29	6.34	5.07	2.24	2.07	2.74	6.03

* 0.1 % DMSO

120 h incubation time

	ctrl.*	1 µM	5 µM	10 µM	20 µM	40 µM	60 µM	80 µM	100 µM
**OD well 1**	2.21	2.11	2.13	2.01	1.65	0.91	0.34	0.22	0.17
**OD well 2**	2.20	2.14	2.09	2.06	1.52	0.90	0.35	0.22	0.17
**OD well 3**	2.12	2.06	2.04	1.86	1.43	0.83	0.34	0.19	0.16
**MEAN**	2.18	2.10	2.09	1.98	1.54	0.88	0.34	0.21	0.17
**SD**	0.05	0.04	0.05	0.10	0.11	0.05	0.01	0.01	0.01
**REL [%]**	**100.0**	**96.7**	**95.9**	**90.9**	**70.6**	**40.6**	**15.8**	**9.7**	**7.7**
**CV [%]**	2.36	2.03	2.32	5.14	7.28	5.23	2.29	6.78	4.01

* 0.1 % DMSO

Experiment 3

48 h incubation time

	ctrl.*	1 µM	5 µM	10 µM	20 µM	40 µM	60 µM	80 µM	100 µM
**OD well 1**	0.25	0.22	0.24	0.23	0.21	0.19	0.15	0.14	0.14
**OD well 2**	0.22	0.21	0.24	0.20	0.18	0.17	0.15	0.15	0.14
**OD well 3**	0.24	0.25	0.25	0.23	0.20	0.18	0.15	0.14	0.14
**MEAN**	0.24	0.23	0.24	0.22	0.20	0.18	0.15	0.14	0.14
**SD**	0.01	0.02	0.01	0.02	0.01	0.01	0.00	0.00	0.00
**REL [%]**	**100.0**	**95.2**	**100.2**	**92.1**	**81.3**	**75.0**	**62.8**	**59.7**	**59.1**
**CV [%]**	6.22	8.72	2.33	6.99	5.35	3.44	2.42	3.18	1.09

* 0.1 % DMSO

72 h incubation time

	ctrl.*	1 µM	5 µM	10 µM	20 µM	40 µM	60 µM	80 µM	100 µM
**OD well 1**	0.68	0.59	0.58	0.54	0.42	0.27	0.20	0.15	0.13
**OD well 2**	0.61	0.54	0.53	0.52	0.39	0.26	0.19	0.15	0.14
**OD well 3**	0.65	0.56	0.57	0.54	0.41	0.27	0.19	0.15	0.14
**MEAN**	0.65	0.57	0.56	0.54	0.40	0.27	0.19	0.15	0.14
**SD**	0.04	0.02	0.03	0.01	0.01	0.01	0.00	0.00	0.00
**REL [%]**	**100.0**	**87.8**	**87.2**	**83.0**	**62.6**	**41.3**	**29.7**	**23.0**	**21.0**
**CV [%]**	5.66	4.37	4.84	2.07	3.10	3.59	2.39	2.07	3.32

* 0.1 % DMSO

120 h incubation time

	ctrl.*	1 µM	5 µM	10 µM	20 µM	40 µM	60 µM	80 µM	100 µM
**OD well 1**	2.35	2.22	2.38	2.08	1.74	0.95	0.45	0.25	0.17
**OD well 2**	2.11	2.25	2.34	2.12	1.79	0.83	0.41	0.23	0.17
**OD well 3**	2.07	2.07	2.17	1.94	1.65	0.78	0.39	0.21	0.17
**MEAN**	2.18	2.18	2.30	2.05	1.73	0.85	0.42	0.23	0.17
**SD**	0.15	0.09	0.12	0.09	0.07	0.09	0.03	0.02	0.00
**REL [%]**	**100.0**	**100.1**	**105.4**	**94.1**	**79.3**	**39.1**	**19.2**	**10.5**	**7.8**
**CV [%]**	6.92	4.27	5.03	4.57	4.08	10.08	7.21	10.06	2.65

* 0.1 % DMSO

Experiment 4

48 h incubation time

	ctrl.*	1 µM	5 µM	10 µM	20 µM	40 µM	60 µM	80 µM	100 µM
**OD well 1**	0.24	0.21	0.23	0.21	0.19	0.18	0.17	0.14	0.14
**OD well 2**	0.23	0.23	0.21	0.22	0.19	0.17	0.17	0.14	0.12
**OD well 3**	0.25	0.22	0.24	0.25	0.21	0.20	0.18	0.17	0.14
**MEAN**	0.24	0.22	0.23	0.23	0.20	0.18	0.17	0.15	0.14
**SD**	0.01	0.01	0.01	0.02	0.01	0.02	0.00	0.01	0.01
**REL [%]**	**100.0**	**91.9**	**93.8**	**93.6**	**83.3**	**75.8**	**71.7**	**62.6**	**56.1**
**CV [%]**	4.49	3.61	5.02	8.61	5.65	9.91	2.11	9.80	9.07

* 0.1 % DMSO

72 h incubation time

	ctrl.*	1 µM	5 µM	10 µM	20 µM	40 µM	60 µM	80 µM	100 µM
**OD well 1**	0.49	0.53	0.53	0.49	0.37	0.28	0.21	0.17	0.15
**OD well 2**	0.49	0.50	0.53	0.50	0.40	0.30	0.23	0.18	0.15
**OD well 3**	0.51	0.55	0.54	0.52	0.38	0.28	0.23	0.20	0.17
**MEAN**	0.50	0.53	0.54	0.50	0.39	0.29	0.22	0.18	0.16
**SD**	0.01	0.03	0.01	0.02	0.01	0.01	0.01	0.01	0.01
**REL [%]**	**100.0**	**106.4**	**108.0**	**101.2**	**77.9**	**57.5**	**44.8**	**36.8**	**31.5**
**CV [%]**	2.40	4.91	1.23	3.81	3.77	3.56	2.94	7.70	5.43

* 0.1 % DMSO

120 h incubation time

	ctrl.*	1 µM	5 µM	10 µM	20 µM	40 µM	60 µM	80 µM	100 µM
**OD well 1**	2.16	2.11	2.22	1.98	1.42	0.63	0.32	0.23	0.16
**OD well 2**	2.09	2.18	2.12	2.09	1.50	0.58	0.33	0.23	0.17
**OD well 3**	2.00	2.07	2.06	2.00	1.40	0.60	0.31	0.19	0.17
**MEAN**	2.08	2.12	2.13	2.02	1.44	0.61	0.32	0.22	0.17
**SD**	0.08	0.06	0.08	0.06	0.06	0.03	0.01	0.02	0.01
**REL [%]**	**100.0**	**101.7**	**102.3**	**97.0**	**69.1**	**29.1**	**15.3**	**10.6**	**8.0**
**CV [%]**	3.93	2.77	3.77	2.98	3.91	4.26	3.93	10.56	4.07

* 0.1 % DMSO

Experiment 5

48 h incubation time

	ctrl.*	1 µM	5 µM	10 µM	20 µM	40 µM	60 µM	80 µM	100 µM
**OD well 1**	0.26	0.26	0.27	0.27	0.22	0.19	0.16	0.15	0.14
**OD well 2**	0.26	0.27	0.26	0.25	0.22	0.20	0.16	0.14	0.13
**OD well 3**	0.26	0.25	0.25	0.25	0.20	0.17	0.16	0.15	0.12
**MEAN**	0.26	0.26	0.26	0.25	0.21	0.19	0.16	0.14	0.13
**SD**	0.00	0.01	0.01	0.01	0.01	0.02	0.00	0.01	0.01
**REL [%]**	**100.0**	**100.0**	**100.3**	**97.7**	**82.2**	**71.7**	**61.8**	**55.7**	**50.8**
**CV [%]**	1.48	4.82	3.68	4.36	5.24	8.71	1.44	3.65	9.00

* 0.1 % DMSO

72 h incubation time

	ctrl.*	1 µM	5 µM	10 µM	20 µM	40 µM	60 µM	80 µM	100 µM
**OD well 1**	0.63	0.60	0.62	0.55	0.45	0.28	0.21	0.18	0.17
**OD well 2**	0.58	0.56	0.57	0.49	0.44	0.30	0.21	0.17	0.16
**OD well 3**	0.69	0.59	0.57	0.59	0.49	0.32	0.22	0.18	0.17
**MEAN**	0.63	0.58	0.59	0.54	0.46	0.30	0.22	0.18	0.16
**SD**	0.05	0.02	0.03	0.05	0.02	0.02	0.01	0.00	0.01
**REL [%]**	**100.0**	**92.1**	**92.5**	**86.0**	**72.6**	**47.2**	**34.0**	**27.8**	**25.6**
**CV [%]**	8.11	3.46	5.34	9.16	5.14	7.98	3.43	1.19	3.81

* 0.1 % DMSO

120 h incubation time

	ctrl.*	1 µM	5 µM	10 µM	20 µM	40 µM	60 µM	80 µM	100 µM
**OD well 1**	2.14	2.20	2.18	2.05	1.60	0.72	0.34	0.22	0.17
**OD well 2**	1.99	2.17	2.18	2.02	1.53	0.74	0.31	0.22	0.16
**OD well 3**	2.00	2.10	2.13	1.94	1.45	0.72	0.32	0.22	0.17
**MEAN**	2.04	2.16	2.16	2.01	1.53	0.73	0.32	0.22	0.16
**SD**	0.08	0.06	0.03	0.06	0.07	0.01	0.02	0.00	0.01
**REL [%]**	**100.0**	**105.6**	**105.8**	**98.3**	**74.8**	**35.6**	**15.8**	**10.8**	**8.0**
**CV [%]**	4.14	2.57	1.46	2.82	4.63	1.64	4.94	1.74	3.52

* 0.1 % DMSO

IRA 1

Experiment 1

48 h incubation time

	ctrl.*	1 µM	5 µM	10 µM	20 µM	40 µM	60 µM	80 µM	100 µM
**OD well 1**	0.26	0.25	0.28	0.28	0.27	0.27	0.25	0.25	0.26
**OD well 2**	0.25	0.27	0.26	0.26	0.29	0.25	0.27	0.27	0.26
**OD well 3**	0.29	0.27	0.26	0.27	0.28	0.25	0.27	0.24	0.26
**MEAN**	0.27	0.26	0.27	0.27	0.28	0.26	0.27	0.25	0.26
**SD**	0.02	0.01	0.01	0.01	0.01	0.01	0.01	0.01	0.00
**REL [%]**	**100.0**	**98.8**	**100.6**	**101.4**	**106.1**	**97.1**	**100.2**	**95.5**	**97.4**
**CV [%]**	7.50	4.75	3.80	3.53	3.21	4.55	3.96	5.27	1.91

* 0.1 % DMSO

72 h incubation time

	ctrl.*	1 µM	5 µM	10 µM	20 µM	40 µM	60 µM	80 µM	100 µM
**OD well 1**	0.58	0.72	0.65	0.64	0.67	0.68	0.71	0.67	0.66
**OD well 2**	0.57	0.61	0.65	0.70	0.70	0.66	0.67	0.66	0.61
**OD well 3**	0.58	0.67	0.76	0.69	0.70	0.70	0.67	0.66	0.68
**MEAN**	0.58	0.66	0.69	0.68	0.69	0.68	0.68	0.66	0.65
**SD**	0.00	0.06	0.06	0.03	0.02	0.02	0.02	0.00	0.04
**REL [%]**	**100.0**	**114.8**	**118.8**	**117.1**	**119.2**	**118.0**	**118.3**	**114.8**	**112.6**
**CV [%]**	0.80	8.45	9.29	4.38	2.85	3.46	3.20	0.41	5.49

* 0.1 % DMSO

120 h incubation time

	ctrl.*	1 µM	5 µM	10 µM	20 µM	40 µM	60 µM	80 µM	100 µM
**OD well 1**	1.80	1.99	2.20	2.07	1.89	1.83	1.90	1.85	1.79
**OD well 2**	1.80	2.10	2.27	2.15	2.02	1.99	1.93	1.98	1.78
**OD well 3**	1.84	2.14	2.12	2.12	1.96	1.93	1.96	1.84	1.79
**MEAN**	1.81	2.08	2.20	2.11	1.96	1.91	1.93	1.89	1.79
**SD**	0.02	0.08	0.07	0.04	0.06	0.08	0.03	0.08	0.01
**REL [%]**	**100.0**	**114.5**	**121.1**	**116.4**	**107.8**	**105.5**	**106.3**	**104.2**	**98.6**
**CV [%]**	1.24	3.91	3.28	2.05	3.26	4.26	1.61	4.25	0.34

* 0.1 % DMSO

Experiment 2

48 h incubation time

	ctrl.*	1 µM	5 µM	10 µM	20 µM	40 µM	60 µM	80 µM	100 µM
**OD well 1**	0.23	0.23	0.21	0.22	0.23	0.23	0.22	0.24	0.24
**OD well 2**	0.23	0.21	0.21	0.22	0.22	0.24	0.23	0.23	0.22
**OD well 3**	0.23	0.21	0.21	0.21	0.21	0.23	0.21	0.22	0.21
**MEAN**	0.23	0.22	0.21	0.22	0.22	0.23	0.22	0.23	0.22
**SD**	0.00	0.01	0.00	0.00	0.01	0.01	0.01	0.01	0.02
**REL [%]**	**100.0**	**92.6**	**90.4**	**93.0**	**92.8**	**100.1**	**93.9**	**98.4**	**94.4**
**CV [%]**	0.32	5.35	1.78	1.70	4.60	3.86	4.53	2.82	7.35

* 0.1 % DMSO

72 h incubation time

	ctrl.*	1 µM	5 µM	10 µM	20 µM	40 µM	60 µM	80 µM	100 µM
**OD well 1**	0.45	0.53	0.47	0.50	0.46	0.50	0.53	0.50	0.49
**OD well 2**	0.47	0.47	0.50	0.47	0.45	0.51	0.50	0.49	0.49
**OD well 3**	0.47	0.51	0.49	0.51	0.45	0.50	0.48	0.46	0.48
**MEAN**	0.47	0.50	0.48	0.49	0.45	0.50	0.51	0.48	0.49
**SD**	0.01	0.03	0.02	0.02	0.00	0.00	0.03	0.02	0.01
**REL [%]**	**100.0**	**108.1**	**104.1**	**105.9**	**97.4**	**108.3**	**109.1**	**103.2**	**104.5**
**CV [%]**	2.82	6.19	3.40	4.58	0.87	0.51	4.98	4.51	1.73

* 0.1 % DMSO

120 h incubation time

	ctrl.*	1 µM	5 µM	10 µM	20 µM	40 µM	60 µM	80 µM	100 µM
**OD well 1**	1.65	1.92	1.89	2.02	1.89	1.82	1.72	1.64	1.74
**OD well 2**	1.57	1.80	1.93	1.94	1.84	1.85	1.79	1.60	1.65
**OD well 3**	1.75	1.83	1.74	1.83	1.77	1.70	1.69	1.54	1.65
**MEAN**	1.66	1.85	1.85	1.93	1.83	1.79	1.73	1.60	1.68
**SD**	0.09	0.06	0.10	0.10	0.06	0.08	0.05	0.05	0.05
**REL [%]**	**100.0**	**111.6**	**111.8**	**116.7**	**110.5**	**107.8**	**104.5**	**96.4**	**101.4**
**CV [%]**	5.25	3.20	5.35	4.94	3.20	4.47	3.03	3.13	3.26

* 0.1 % DMSO

Experiment 3

48 h incubation time

	ctrl.*	1 µM	5 µM	10 µM	20 µM	40 µM	60 µM	80 µM	100 µM
**OD well 1**	0.24	0.25	0.25	0.25	0.24	0.25	0.24	0.23	0.22
**OD well 2**	0.22	0.23	0.23	0.20	0.21	0.23	0.23	0.22	0.22
**OD well 3**	0.22	0.22	0.23	0.23	0.19	0.24	0.23	0.21	0.22
**MEAN**	0.23	0.23	0.24	0.23	0.21	0.24	0.24	0.22	0.22
**SD**	0.01	0.02	0.01	0.03	0.02	0.01	0.01	0.01	0.00
**REL [%]**	**100.0**	**101.3**	**102.7**	**99.7**	**92.9**	**104.0**	**103.0**	**95.5**	**95.8**
**CV [%]**	4.27	6.87	6.10	11.20	10.52	5.39	2.35	3.74	1.16

* 0.1 % DMSO

72 h incubation time

	ctrl.*	1 µM	5 µM	10 µM	20 µM	40 µM	60 µM	80 µM	100 µM
**OD well 1**	0.46	0.47	0.50	0.48	0.50	0.44	0.48	0.48	0.47
**OD well 2**	0.45	0.46	0.48	0.50	0.49	0.46	0.48	0.46	0.44
**OD well 3**	0.50	0.47	0.48	0.51	0.49	0.45	0.45	0.47	0.46
**MEAN**	0.47	0.46	0.49	0.50	0.49	0.45	0.47	0.47	0.46
**SD**	0.03	0.01	0.01	0.02	0.01	0.01	0.02	0.01	0.02
**REL [%]**	**100.0**	**98.9**	**103.7**	**106.3**	**105.4**	**96.1**	**100.1**	**100.5**	**97.7**
**CV [%]**	5.91	1.48	1.94	3.13	1.51	2.22	3.72	2.13	3.55

* 0.1 % DMSO

120 h incubation time

	ctrl.*	1 µM	5 µM	10 µM	20 µM	40 µM	60 µM	80 µM	100 µM
**OD well 1**	1.76	2.03	2.08	2.02	2.04	1.94	1.95	1.98	1.70
**OD well 2**	1.82	1.92	2.06	2.15	2.04	1.98	1.94	1.87	1.65
**OD well 3**	1.87	1.88	1.99	2.08	2.07	1.94	1.88	1.82	1.79
**MEAN**	1.82	1.94	2.04	2.08	2.05	1.96	1.92	1.89	1.71
**SD**	0.05	0.08	0.05	0.07	0.01	0.02	0.04	0.08	0.07
**REL [%]**	**100.0**	**107.0**	**112.5**	**114.7**	**112.9**	**107.7**	**106.0**	**103.9**	**94.3**
**CV [%]**	3.00	3.89	2.41	3.19	0.72	1.22	2.02	4.31	4.25

* 0.1 % DMSO

Experiment 4

48 h incubation time

	ctrl.*	1 µM	5 µM	10 µM	20 µM	40 µM	60 µM	80 µM	100 µM
**OD well 1**	0.22	0.23	0.21	0.24	0.24	0.23	0.25	0.23	0.24
**OD well 2**	0.22	0.23	0.24	0.24	0.24	0.22	0.23	0.22	0.23
**OD well 3**	0.23	0.25	0.25	0.25	0.25	0.23	0.25	0.25	0.22
**MEAN**	0.22	0.23	0.23	0.24	0.24	0.23	0.25	0.23	0.23
**SD**	0.00	0.01	0.02	0.01	0.00	0.01	0.01	0.01	0.01
**REL [%]**	**100.0**	**105.8**	**104.5**	**108.7**	**109.4**	**103.3**	**110.7**	**105.8**	**105.1**
**CV [%]**	2.11	5.39	7.04	2.55	1.76	2.36	4.78	4.88	3.97

* 0.1 % DMSO

72 h incubation time

	ctrl.*	1 µM	5 µM	10 µM	20 µM	40 µM	60 µM	80 µM	100 µM
**OD well 1**	0.52	0.53	0.51	0.58	0.55	0.55	0.53	0.53	0.50
**OD well 2**	0.46	0.53	0.52	0.58	0.54	0.52	0.54	0.49	0.48
**OD well 3**	0.51	0.51	0.52	0.55	0.54	0.54	0.57	0.58	0.52
**MEAN**	0.50	0.52	0.52	0.57	0.54	0.54	0.55	0.53	0.50
**SD**	0.03	0.01	0.01	0.02	0.01	0.02	0.02	0.05	0.02
**REL [%]**	**100.0**	**105.0**	**104.0**	**114.6**	**109.2**	**108.7**	**109.9**	**106.9**	**100.9**
**CV [%]**	5.76	2.68	1.48	2.73	1.13	3.18	3.72	9.13	3.52

* 0.1 % DMSO

120 h incubation time

	ctrl.*	1 µM	5 µM	10 µM	20 µM	40 µM	60 µM	80 µM	100 µM
**OD well 1**	2.01	2.16	2.23	2.19	2.24	2.10	2.05	1.86	1.84
**OD well 2**	2.04	2.20	2.16	2.23	2.22	2.20	2.04	1.87	1.85
**OD well 3**	1.98	2.05	2.04	2.11	2.13	2.04	1.95	1.79	1.79
**MEAN**	2.01	2.14	2.14	2.18	2.20	2.11	2.01	1.84	1.83
**SD**	0.03	0.08	0.10	0.06	0.06	0.08	0.06	0.04	0.03
**REL [%]**	**100.0**	**106.3**	**106.4**	**108.3**	**109.2**	**104.9**	**99.9**	**91.4**	**90.7**
**CV [%]**	1.50	3.74	4.55	2.87	2.75	3.91	2.79	2.29	1.66

* 0.1 % DMSO

Experiment 5

48 h incubation time

	ctrl.*	1 µM	5 µM	10 µM	20 µM	40 µM	60 µM	80 µM	100 µM
**OD well 1**	0.28	0.28	0.28	0.27	0.26	0.27	0.30	0.27	0.26
**OD well 2**	0.27	0.27	0.27	0.25	0.24	0.32	0.35	0.24	0.23
**OD well 3**	0.28	0.31	0.26	0.26	0.24	0.28	0.28	0.25	0.23
**MEAN**	0.28	0.28	0.27	0.26	0.25	0.29	0.31	0.25	0.24
**SD**	0.01	0.02	0.01	0.01	0.01	0.03	0.04	0.02	0.02
**REL [%]**	**100.0**	**103.1**	**97.8**	**93.6**	**89.8**	**104.7**	**112.0**	**91.8**	**86.8**
**CV [%]**	2.74	6.95	4.70	2.48	4.78	8.71	11.46	6.85	6.50

* 0.1 % DMSO

72 h incubation time

	ctrl.*	1 µM	5 µM	10 µM	20 µM	40 µM	60 µM	80 µM	100 µM
**OD well 1**	0.60	0.61	0.55	0.57	0.61	0.60	0.61	0.53	0.49
**OD well 2**	0.58	0.65	0.61	0.59	0.63	0.61	0.59	0.52	0.48
**OD well 3**	0.60	0.64	0.60	0.59	0.57	0.54	0.63	0.54	0.51
**MEAN**	0.59	0.63	0.58	0.58	0.61	0.58	0.61	0.53	0.49
**SD**	0.01	0.02	0.03	0.01	0.03	0.04	0.02	0.01	0.02
**REL [%]**	**100.0**	**107.2**	**98.8**	**98.2**	**102.4**	**98.5**	**102.9**	**89.6**	**83.2**
**CV [%]**	2.28	3.61	5.66	1.62	5.21	6.64	2.62	2.35	3.17

* 0.1 % DMSO

120 h incubation time

	ctrl.*	1 µM	5 µM	10 µM	20 µM	40 µM	60 µM	80 µM	100 µM
**OD well 1**	1.73	1.85	2.05	2.04	2.08	2.02	1.78	1.48	1.48
**OD well 2**	1.71	2.10	2.17	2.04	2.11	1.92	1.75	1.55	1.44
**OD well 3**	1.62	2.12	2.11	1.90	2.05	1.84	1.72	1.40	1.42
**MEAN**	1.69	2.03	2.11	2.00	2.08	1.93	1.75	1.48	1.45
**SD**	0.06	0.15	0.06	0.08	0.03	0.09	0.03	0.08	0.03
**REL [%]**	**100.0**	**120.0**	**124.8**	**118.1**	**123.1**	**114.0**	**103.5**	**87.5**	**85.8**
**CV [%]**	3.40	7.51	2.87	3.93	1.28	4.54	1.65	5.11	2.08

* 0.1 % DMSO

IRA 2

Experiment 1

48 h incubation time

	ctrl.*	1 µM	5 µM	10 µM	20 µM	40 µM	60 µM	80 µM	100 µM
**OD well 1**	0.27	0.24	0.27	0.26	0.26	0.26	0.27	0.29	0.25
**OD well 2**	0.28	0.24	0.27	0.26	0.26	0.25	0.27	0.27	0.26
**OD well 3**	0.27	0.25	0.28	0.28	0.29	0.30	0.26	0.28	0.27
**MEAN**	0.27	0.24	0.27	0.27	0.27	0.27	0.27	0.28	0.26
**SD**	0.00	0.01	0.01	0.01	0.02	0.02	0.00	0.01	0.01
**REL [%]**	**100.0**	**90.3**	**101.7**	**98.3**	**99.3**	**100.2**	**98.2**	**103.9**	**96.6**
**CV [%]**	1.51	2.70	3.04	3.46	6.62	8.80	1.19	4.53	2.95

* 0.1 % DMSO

72 h incubation time

	ctrl.*	1 µM	5 µM	10 µM	20 µM	40 µM	60 µM	80 µM	100 µM
**OD well 1**	0.63	0.60	0.62	0.57	0.66	0.68	0.63	0.62	0.61
**OD well 2**	0.73	0.70	0.66	0.64	0.71	0.69	0.68	0.68	0.64
**OD well 3**	0.67	0.64	0.66	0.65	0.70	0.74	0.63	0.63	0.62
**MEAN**	0.68	0.65	0.65	0.62	0.69	0.70	0.65	0.64	0.62
**SD**	0.05	0.05	0.02	0.04	0.02	0.03	0.03	0.03	0.01
**REL [%]**	**100.0**	**95.2**	**95.5**	**91.9**	**101.3**	**103.6**	**95.6**	**94.6**	**91.9**
**CV [%]**	7.23	7.62	3.84	6.95	3.30	4.62	4.50	4.99	2.00

* 0.1 % DMSO

120 h incubation time

	ctrl.*	1 µM	5 µM	10 µM	20 µM	40 µM	60 µM	80 µM	100 µM
**OD well 1**	1.93	1.78	1.96	1.81	1.63	1.47	1.59	1.53	1.46
**OD well 2**	1.81	1.62	1.91	1.80	1.55	1.51	1.55	1.48	1.42
**OD well 3**	1.85	1.56	1.74	1.66	1.48	1.48	1.46	1.44	1.42
**MEAN**	1.86	1.65	1.87	1.76	1.55	1.49	1.53	1.48	1.43
**SD**	0.06	0.11	0.11	0.08	0.07	0.02	0.07	0.05	0.02
**REL [%]**	**100.0**	**88.7**	**100.5**	**94.4**	**83.2**	**79.8**	**82.2**	**79.7**	**77.0**
**CV [%]**	3.31	6.80	6.10	4.57	4.80	1.30	4.30	3.11	1.44

* 0.1 % DMSO

Experiment 2

48 h incubation time

	ctrl.*	1 µM	5 µM	10 µM	20 µM	40 µM	60 µM	80 µM	100 µM
**OD well 1**	0.23	0.22	0.23	0.23	0.21	0.22	0.22	0.22	0.21
**OD well 2**	0.22	0.21	0.21	0.23	0.19	0.21	0.21	0.21	0.21
**OD well 3**	0.24	0.21	0.22	0.22	0.21	0.22	0.23	0.22	0.21
**MEAN**	0.23	0.21	0.22	0.22	0.20	0.22	0.22	0.22	0.21
**SD**	0.01	0.00	0.01	0.01	0.01	0.01	0.01	0.01	0.00
**REL [%]**	**100.0**	**93.4**	**95.9**	**97.3**	**88.6**	**95.2**	**96.0**	**94.8**	**91.1**
**CV [%]**	4.07	1.72	3.93	2.88	5.56	4.23	4.92	2.70	1.96

* 0.1 % DMSO

72 h incubation time

	ctrl.*	1 µM	5 µM	10 µM	20 µM	40 µM	60 µM	80 µM	100 µM
**OD well 1**	0.44	0.46	0.41	0.44	0.40	0.38	0.40	0.41	0.43
**OD well 2**	0.41	0.42	0.35	0.44	0.38	0.39	0.38	0.40	0.40
**OD well 3**	0.45	0.44	0.40	0.41	0.38	0.43	0.40	0.40	0.41
**MEAN**	0.43	0.44	0.39	0.43	0.39	0.40	0.39	0.40	0.41
**SD**	0.02	0.02	0.03	0.02	0.01	0.03	0.01	0.01	0.02
**REL [%]**	**100.0**	**101.8**	**89.6**	**98.6**	**89.5**	**92.1**	**91.2**	**93.2**	**95.3**
**CV [%]**	4.65	4.89	8.76	4.38	3.51	7.84	2.29	1.68	3.77

* 0.1 % DMSO

120 h incubation time

	ctrl.*	1 µM	5 µM	10 µM	20 µM	40 µM	60 µM	80 µM	100 µM
**OD well 1**	1.53	1.52	1.41	1.67	1.43	1.36	1.26	1.25	1.25
**OD well 2**	1.51	1.57	1.34	1.60	1.37	1.37	1.27	1.25	1.27
**OD well 3**	1.50	1.45	1.35	1.59	1.25	1.43	1.30	1.27	1.32
**MEAN**	1.51	1.51	1.37	1.62	1.35	1.39	1.28	1.26	1.28
**SD**	0.02	0.06	0.04	0.04	0.09	0.03	0.02	0.01	0.04
**REL [%]**	**100.0**	**100.1**	**90.3**	**107.2**	**89.6**	**91.8**	**84.4**	**83.1**	**84.6**
**CV [%]**	1.07	3.89	2.91	2.61	6.80	2.51	1.70	0.67	2.80

* 0.1 % DMSO

Experiment 3

48 h incubation time

	ctrl.*	1 µM	5 µM	10 µM	20 µM	40 µM	60 µM	80 µM	100 µM
**OD well 1**	0.24	0.22	0.20	0.22	0.21	0.21	0.21	0.21	0.20
**OD well 2**	0.23	0.25	0.20	0.20	0.21	0.20	0.22	0.20	0.20
**OD well 3**	0.24	0.25	0.24	0.25	0.22	0.21	0.23	0.20	0.22
**MEAN**	0.24	0.24	0.21	0.22	0.22	0.21	0.22	0.20	0.21
**SD**	0.00	0.01	0.02	0.02	0.01	0.00	0.01	0.01	0.01
**REL [%]**	**100.0**	**101.4**	**90.9**	**94.5**	**91.5**	**88.4**	**93.8**	**86.5**	**87.7**
**CV [%]**	1.50	6.26	9.58	9.41	2.88	2.05	4.45	3.84	4.86

* 0.1 % DMSO

72 h incubation time

	ctrl.*	1 µM	5 µM	10 µM	20 µM	40 µM	60 µM	80 µM	100 µM
**OD well 1**	0.50	0.49	0.48	0.47	0.49	0.46	0.39	0.42	0.43
**OD well 2**	0.44	0.45	0.48	0.45	0.45	0.42	0.40	0.40	0.43
**OD well 3**	0.47	0.47	0.50	0.50	0.44	0.45	0.42	0.42	0.41
**MEAN**	0.47	0.47	0.49	0.47	0.46	0.45	0.40	0.41	0.42
**SD**	0.03	0.02	0.01	0.02	0.02	0.02	0.01	0.01	0.01
**REL [%]**	**100.0**	**99.8**	**103.6**	**100.2**	**97.4**	**94.7**	**85.9**	**88.2**	**89.2**
**CV [%]**	5.70	4.50	2.05	5.27	5.27	4.28	2.76	2.89	2.90

* 0.1 % DMSO

120 h incubation time

	ctrl.*	1 µM	5 µM	10 µM	20 µM	40 µM	60 µM	80 µM	100 µM
**OD well 1**	1.87	1.95	1.95	1.89	1.61	1.62	1.48	1.41	1.32
**OD well 2**	1.79	1.87	1.90	1.87	1.54	1.56	1.51	1.40	1.38
**OD well 3**	1.91	1.92	1.95	1.92	1.61	1.55	1.51	1.42	1.37
**MEAN**	1.86	1.91	1.94	1.89	1.59	1.58	1.50	1.41	1.35
**SD**	0.06	0.04	0.03	0.03	0.04	0.04	0.02	0.01	0.04
**REL [%]**	**100.0**	**102.9**	**104.2**	**101.9**	**85.5**	**84.9**	**80.8**	**75.9**	**72.9**
**CV [%]**	3.31	2.20	1.56	1.48	2.63	2.34	1.05	0.59	2.63

* 0.1 % DMSO

Experiment 4

48 h incubation time

	ctrl.*	1 µM	5 µM	10 µM	20 µM	40 µM	60 µM	80 µM	100 µM
**OD well 1**	0.25	0.24	0.23	0.22	0.22	0.24	0.23	0.24	0.24
**OD well 2**	0.23	0.22	0.22	0.23	0.21	0.24	0.24	0.22	0.23
**OD well 3**	0.24	0.24	0.21	0.23	0.24	0.21	0.24	0.23	0.22
**MEAN**	0.24	0.23	0.22	0.23	0.22	0.23	0.24	0.23	0.23
**SD**	0.01	0.01	0.01	0.01	0.01	0.01	0.01	0.01	0.01
**REL [%]**	**100.0**	**97.7**	**90.2**	**94.7**	**93.3**	**96.4**	**98.1**	**96.2**	**96.5**
**CV [%]**	2.82	4.90	4.29	2.32	5.89	6.13	3.70	4.94	4.84

* 0.1 % DMSO

72 h incubation time

	ctrl.*	1 µM	5 µM	10 µM	20 µM	40 µM	60 µM	80 µM	100 µM
**OD well 1**	0.53	0.48	0.51	0.50	0.42	0.53	0.56	0.50	0.49
**OD well 2**	0.50	0.49	0.50	0.48	0.52	0.52	0.50	0.47	0.46
**OD well 3**	0.47	0.48	0.49	0.46	0.48	0.48	0.52	0.49	0.49
**MEAN**	0.50	0.49	0.50	0.48	0.48	0.51	0.53	0.49	0.48
**SD**	0.03	0.00	0.01	0.02	0.05	0.02	0.03	0.02	0.02
**REL [%]**	**100.0**	**97.6**	**100.8**	**96.6**	**95.6**	**102.8**	**105.4**	**97.6**	**96.2**
**CV [%]**	5.96	0.47	2.25	4.47	10.46	4.83	5.95	3.59	3.40

* 0.1 % DMSO

120 h incubation time

	ctrl.*	1 µM	5 µM	10 µM	20 µM	40 µM	60 µM	80 µM	100 µM
**OD well 1**	1.92	2.05	2.04	1.92	1.83	1.63	1.56	1.44	1.39
**OD well 2**	1.83	2.08	2.03	2.05	1.68	1.64	1.56	1.41	1.41
**OD well 3**	1.78	2.03	2.03	1.99	1.79	1.58	1.51	1.45	1.41
**MEAN**	1.84	2.05	2.03	1.98	1.77	1.62	1.54	1.44	1.40
**SD**	0.07	0.03	0.01	0.07	0.08	0.03	0.03	0.02	0.01
**REL [%]**	**100.0**	**111.4**	**110.3**	**107.6**	**95.9**	**87.7**	**83.7**	**77.8**	**76.1**
**CV [%]**	3.97	1.24	0.40	3.30	4.46	1.96	1.65	1.58	0.71

* 0.1 % DMSO

Experiment 5

48 h incubation time

	ctrl.*	1 µM	5 µM	10 µM	20 µM	40 µM	60 µM	80 µM	100 µM
**OD well 1**	0.25	0.24	0.25	0.25	0.24	0.25	0.27	0.27	0.25
**OD well 2**	0.28	0.23	0.24	0.24	0.23	0.25	0.26	0.25	0.26
**OD well 3**	0.27	0.24	0.24	0.26	0.25	0.26	0.27	0.26	0.27
**MEAN**	0.27	0.24	0.24	0.25	0.24	0.25	0.27	0.26	0.26
**SD**	0.02	0.01	0.01	0.01	0.01	0.01	0.01	0.01	0.01
**REL [%]**	**100.0**	**88.9**	**90.8**	**93.7**	**90.2**	**94.9**	**99.1**	**95.8**	**97.1**
**CV [%]**	6.17	2.56	2.57	5.16	3.13	2.01	3.23	3.67	4.45

* 0.1 % DMSO

72 h incubation time

	ctrl.*	1 µM	5 µM	10 µM	20 µM	40 µM	60 µM	80 µM	100 µM
**OD well 1**	0.54	0.52	0.48	0.54	0.53	0.55	0.56	0.50	0.49
**OD well 2**	0.51	0.48	0.50	0.56	0.51	0.47	0.55	0.52	0.51
**OD well 3**	0.48	0.54	0.50	0.55	0.53	0.50	0.54	0.50	0.48
**MEAN**	0.51	0.51	0.50	0.55	0.52	0.51	0.55	0.51	0.49
**SD**	0.03	0.03	0.01	0.01	0.01	0.04	0.01	0.01	0.01
**REL [%]**	**100.0**	**100.2**	**97.1**	**107.3**	**102.0**	**99.0**	**108.1**	**100.0**	**96.5**
**CV [%]**	6.54	6.30	2.61	1.48	2.08	8.76	1.70	2.25	2.64

* 0.1 % DMSO

120 h incubation time

	ctrl.*	1 µM	5 µM	10 µM	20 µM	40 µM	60 µM	80 µM	100 µM
**OD well 1**	1.75	1.44	1.53	1.70	1.74	1.48	1.37	1.24	1.26
**OD well 2**	1.67	1.48	1.50	1.75	1.78	1.47	1.37	1.25	1.30
**OD well 3**	1.55	1.48	1.51	1.79	1.79	1.41	1.36	1.25	1.29
**MEAN**	1.66	1.47	1.51	1.75	1.77	1.46	1.37	1.25	1.28
**SD**	0.10	0.02	0.02	0.04	0.03	0.04	0.01	0.01	0.02
**REL [%]**	**100.0**	**88.7**	**91.4**	**105.5**	**107.0**	**87.9**	**82.5**	**75.2**	**77.4**
**CV [%]**	5.82	1.47	1.09	2.51	1.53	2.51	0.59	0.54	1.74

* 0.1 % DMSO

IRA 3

Experiment 1

48 h incubation time

	ctrl.*	1 µM	5 µM	10 µM	20 µM	40 µM	60 µM	80 µM	100 µM
**OD well 1**	0.23	0.24	0.25	0.25	0.22	0.24	0.24	0.24	0.22
**OD well 2**	0.26	0.23	0.26	0.25	0.22	0.22	0.23	0.22	0.21
**OD well 3**	0.24	0.25	0.27	0.27	0.22	0.23	0.23	0.24	0.22
**MEAN**	0.24	0.24	0.26	0.26	0.22	0.23	0.24	0.23	0.22
**SD**	0.01	0.01	0.01	0.01	0.00	0.01	0.01	0.01	0.00
**REL [%]**	**100.0**	**99.0**	**106.3**	**106.2**	**90.7**	**95.5**	**97.2**	**96.1**	**89.9**
**CV [%]**	5.68	2.61	4.62	4.19	0.76	3.07	2.28	3.16	2.00

* 0.1 % DMSO

72 h incubation time

	ctrl.*	1 µM	5 µM	10 µM	20 µM	40 µM	60 µM	80 µM	100 µM
**OD well 1**	0.61	0.66	0.72	0.70	0.64	0.69	0.69	0.69	0.63
**OD well 2**	0.59	0.65	0.72	0.74	0.70	0.64	0.61	0.64	0.67
**OD well 3**	0.67	0.65	0.70	0.72	0.70	0.63	0.62	0.62	0.64
**MEAN**	0.62	0.65	0.71	0.72	0.68	0.65	0.64	0.65	0.65
**SD**	0.04	0.01	0.01	0.02	0.03	0.03	0.04	0.04	0.02
**REL [%]**	**100.0**	**105.0**	**114.6**	**115.5**	**108.6**	**104.4**	**102.5**	**104.4**	**103.9**
**CV [%]**	6.41	1.35	2.03	2.29	5.12	5.19	6.54	5.96	3.63

* 0.1 % DMSO

120 h incubation time

	ctrl.*	1 µM	5 µM	10 µM	20 µM	40 µM	60 µM	80 µM	100 µM
**OD well 1**	2.03	1.86	1.92	1.63	1.68	1.82	1.83	1.79	1.81
**OD well 2**	1.81	1.98	1.56	1.73	1.68	1.85	1.89	1.75	1.76
**OD well 3**	1.91	1.91	1.59	1.62	1.66	1.85	1.76	1.72	1.72
**MEAN**	1.92	1.92	1.69	1.66	1.67	1.84	1.82	1.75	1.76
**SD**	0.11	0.06	0.20	0.06	0.01	0.02	0.06	0.03	0.04
**REL [%]**	**100.0**	**100.1**	**88.2**	**86.5**	**87.4**	**95.9**	**95.2**	**91.6**	**92.1**
**CV [%]**	5.68	3.22	11.83	3.73	0.51	0.98	3.52	1.94	2.43

* 0.1 % DMSO

Experiment 2

48 h incubation time

	ctrl.*	1 µM	5 µM	10 µM	20 µM	40 µM	60 µM	80 µM	100 µM
**OD well 1**	0.21	0.21	0.20	0.21	0.20	0.20	0.22	0.21	0.20
**OD well 2**	0.20	0.22	0.19	0.20	0.20	0.21	0.21	0.21	0.20
**OD well 3**	0.21	0.20	0.18	0.19	0.20	0.23	0.20	0.20	0.20
**MEAN**	0.21	0.21	0.19	0.20	0.20	0.21	0.21	0.21	0.20
**SD**	0.01	0.01	0.01	0.01	0.00	0.01	0.01	0.00	0.00
**REL [%]**	**100.0**	**103.6**	**92.0**	**97.2**	**98.6**	**103.9**	**102.6**	**100.3**	**97.3**
**CV [%]**	3.67	4.36	4.49	4.25	0.57	5.29	5.31	0.93	1.99

* 0.1 % DMSO

72 h incubation time

	ctrl.*	1 µM	5 µM	10 µM	20 µM	40 µM	60 µM	80 µM	100 µM
**OD well 1**	0.42	0.35	0.35	0.37	0.38	0.41	0.37	0.40	0.37
**OD well 2**	0.39	0.39	0.33	0.37	0.38	0.42	0.40	0.41	0.38
**OD well 3**	0.40	0.37	0.34	0.35	0.39	0.40	0.35	0.39	0.39
**MEAN**	0.40	0.37	0.34	0.36	0.38	0.41	0.38	0.40	0.38
**SD**	0.01	0.02	0.01	0.01	0.00	0.01	0.03	0.01	0.01
**REL [%]**	**100.0**	**91.9**	**84.9**	**89.7**	**95.1**	**102.6**	**93.4**	**99.9**	**94.1**
**CV [%]**	3.60	5.52	3.30	3.29	0.62	2.14	7.25	2.82	1.78

* 0.1 % DMSO

120 h incubation time

	ctrl.*	1 µM	5 µM	10 µM	20 µM	40 µM	60 µM	80 µM	100 µM
**OD well 1**	1.74	1.59	1.66	1.36	1.50	1.71	1.74	1.62	1.50
**OD well 2**	1.81	1.56	1.84	1.52	1.50	1.76	1.62	1.54	1.42
**OD well 3**	1.77	1.71	1.92	1.61	1.66	1.70	1.66	1.53	1.36
**MEAN**	1.77	1.62	1.81	1.49	1.55	1.72	1.68	1.56	1.43
**SD**	0.03	0.08	0.13	0.13	0.09	0.03	0.06	0.05	0.07
**REL [%]**	**100.0**	**91.4**	**101.8**	**84.3**	**87.5**	**97.1**	**94.4**	**88.1**	**80.3**
**CV [%]**	1.75	5.02	7.43	8.47	5.99	1.84	3.78	3.12	5.10

* 0.1 % DMSO

Experiment 3

48 h incubation time

	ctrl.*	1 µM	5 µM	10 µM	20 µM	40 µM	60 µM	80 µM	100 µM
**OD well 1**	0.24	0.24	0.24	0.22	0.22	0.22	0.22	0.22	0.20
**OD well 2**	0.22	0.25	0.25	0.25	0.22	0.22	0.22	0.24	0.22
**OD well 3**	0.24	0.22	0.23	0.25	0.21	0.23	0.24	0.23	0.24
**MEAN**	0.24	0.24	0.24	0.24	0.22	0.22	0.23	0.23	0.22
**SD**	0.01	0.02	0.01	0.02	0.00	0.00	0.01	0.01	0.02
**REL [%]**	**100.0**	**100.2**	**101.3**	**100.6**	**91.2**	**94.9**	**95.5**	**97.5**	**93.5**
**CV [%]**	4.37	6.59	5.18	6.54	0.72	1.41	4.43	3.82	9.44

* 0.1 % DMSO

72 h incubation time

	ctrl.*	1 µM	5 µM	10 µM	20 µM	40 µM	60 µM	80 µM	100 µM
**OD well 1**	0.44	0.45	0.46	0.44	0.39	0.43	0.42	0.45	0.50
**OD well 2**	0.46	0.44	0.40	0.40	0.38	0.46	0.39	0.41	0.46
**OD well 3**	0.45	0.39	0.38	0.38	0.37	0.45	0.38	0.42	0.42
**MEAN**	0.45	0.43	0.41	0.41	0.38	0.45	0.40	0.43	0.46
**SD**	0.01	0.03	0.04	0.03	0.01	0.01	0.02	0.02	0.04
**REL [%]**	**100.0**	**94.4**	**91.3**	**90.2**	**84.7**	**98.8**	**88.0**	**95.0**	**101.2**
**CV [%]**	1.74	7.62	10.81	8.45	2.51	2.64	5.09	4.25	8.64

* 0.1 % DMSO

120 h incubation time

	ctrl.*	1 µM	5 µM	10 µM	20 µM	40 µM	60 µM	80 µM	100 µM
**OD well 1**	1.76	1.83	1.78	1.75	1.62	1.67	1.64	1.68	1.57
**OD well 2**	1.78	1.84	1.77	1.72	1.66	1.62	1.59	1.57	1.49
**OD well 3**	1.77	1.70	1.70	1.74	1.61	1.64	1.55	1.53	1.47
**MEAN**	1.77	1.79	1.75	1.74	1.63	1.64	1.59	1.59	1.51
**SD**	0.01	0.08	0.04	0.02	0.03	0.02	0.05	0.08	0.05
**REL [%]**	**100.0**	**101.1**	**98.9**	**98.2**	**92.1**	**92.8**	**90.2**	**90.1**	**85.5**
**CV [%]**	0.54	4.34	2.51	1.00	1.68	1.48	2.91	4.86	3.53

* 0.1 % DMSO

Experiment 4

48 h incubation time

	ctrl.*	1 µM	5 µM	10 µM	20 µM	40 µM	60 µM	80 µM	100 µM
**OD well 1**	0.22	0.24	0.24	0.25	0.22	0.23	0.25	0.21	0.23
**OD well 2**	0.20	0.23	0.24	0.24	0.22	0.23	0.22	0.22	0.21
**OD well 3**	0.23	0.23	0.24	0.24	0.22	0.22	0.22	0.21	0.21
**MEAN**	0.22	0.23	0.24	0.24	0.22	0.23	0.23	0.21	0.22
**SD**	0.02	0.01	0.00	0.01	0.00	0.00	0.02	0.00	0.01
**REL [%]**	**100.0**	**104.7**	**110.1**	**110.9**	**100.2**	**104.4**	**105.2**	**97.9**	**99.4**
**CV [%]**	6.86	2.85	0.65	3.17	1.51	2.15	6.51	1.59	6.55

* 0.1 % DMSO

72 h incubation time

	ctrl.*	1 µM	5 µM	10 µM	20 µM	40 µM	60 µM	80 µM	100 µM
**OD well 1**	0.54	0.57	0.58	0.58	0.51	0.56	0.56	0.50	0.53
**OD well 2**	0.50	0.53	0.51	0.57	0.51	0.46	0.52	0.50	0.53
**OD well 3**	0.56	0.55	0.53	0.56	0.47	0.51	0.54	0.50	0.54
**MEAN**	0.53	0.55	0.54	0.57	0.50	0.51	0.54	0.50	0.53
**SD**	0.03	0.02	0.04	0.01	0.02	0.05	0.02	0.00	0.00
**REL [%]**	**100.0**	**103.4**	**101.0**	**107.0**	**93.2**	**95.8**	**100.9**	**93.9**	**100.1**
**CV [%]**	5.58	2.96	7.09	2.40	4.52	10.04	4.20	0.67	0.45

* 0.1 % DMSO

120 h incubation time

	ctrl.*	1 µM	5 µM	10 µM	20 µM	40 µM	60 µM	80 µM	100 µM
**OD well 1**	1.90	1.94	1.86	1.84	1.50	1.58	1.66	1.54	1.68
**OD well 2**	1.81	1.81	1.86	1.76	1.49	1.59	1.69	1.56	1.63
**OD well 3**	1.81	1.78	1.74	1.68	1.41	1.60	1.67	1.55	1.58
**MEAN**	1.84	1.84	1.82	1.76	1.46	1.59	1.67	1.55	1.63
**SD**	0.05	0.08	0.07	0.08	0.05	0.01	0.01	0.01	0.05
**REL [%]**	**100.0**	**100.0**	**98.7**	**95.5**	**79.5**	**86.3**	**90.9**	**84.0**	**88.4**
**CV [%]**	2.85	4.55	3.88	4.61	3.49	0.69	0.64	0.73	2.89

* 0.1 % DMSO

Experiment 5

48 h incubation time

	ctrl.*	1 µM	5 µM	10 µM	20 µM	40 µM	60 µM	80 µM	100 µM
**OD well 1**	0.29	0.31	0.29	0.30	0.32	0.32	0.32	0.31	0.26
**OD well 2**	0.31	0.32	0.30	0.33	0.32	0.32	0.32	0.31	0.25
**OD well 3**	0.29	0.28	0.29	0.29	0.31	0.29	0.30	0.28	0.24
**MEAN**	0.30	0.30	0.29	0.31	0.32	0.31	0.31	0.30	0.25
**SD**	0.01	0.02	0.00	0.02	0.01	0.02	0.01	0.02	0.01
**REL [%]**	**100.0**	**101.7**	**98.8**	**102.8**	**105.9**	**104.4**	**104.5**	**100.5**	**84.2**
**CV [%]**	4.88	6.95	1.45	6.15	1.71	5.96	3.78	6.30	4.07

* 0.1 % DMSO

72 h incubation time

	ctrl.*	1 µM	5 µM	10 µM	20 µM	40 µM	60 µM	80 µM	100 µM
**OD well 1**	0.59	0.64	0.65	0.66	0.58	0.66	0.61	0.60	0.57
**OD well 2**	0.58	0.67	0.60	0.67	0.64	0.61	0.62	0.62	0.60
**OD well 3**	0.56	0.64	0.61	0.63	0.62	0.60	0.59	0.58	0.57
**MEAN**	0.58	0.65	0.62	0.66	0.61	0.63	0.60	0.60	0.58
**SD**	0.01	0.02	0.03	0.02	0.03	0.03	0.02	0.02	0.02
**REL [%]**	**100.0**	**112.3**	**106.7**	**113.3**	**105.9**	**108.1**	**104.4**	**103.4**	**99.9**
**CV [%]**	2.51	2.73	4.17	3.04	5.34	4.71	2.82	3.37	3.01

* 0.1 % DMSO

120 h incubation time

	ctrl.*	1 µM	5 µM	10 µM	20 µM	40 µM	60 µM	80 µM	100 µM
**OD well 1**	1.90	1.87	1.92	1.87	1.72	1.69	1.68	1.75	1.62
**OD well 2**	1.84	1.84	1.81	1.76	1.72	1.64	1.63	1.55	1.56
**OD well 3**	1.84	1.88	1.70	1.84	1.74	1.65	1.58	1.56	1.64
**MEAN**	1.86	1.86	1.81	1.82	1.73	1.66	1.63	1.62	1.60
**SD**	0.03	0.02	0.11	0.06	0.01	0.03	0.05	0.11	0.04
**REL [%]**	**100.0**	**100.2**	**97.2**	**97.9**	**92.7**	**89.3**	**87.6**	**87.1**	**86.2**
**CV [%]**	1.74	0.98	6.14	3.15	0.52	1.57	3.05	6.92	2.60

* 0.1 % DMSO

IRA 4

Experiment 1

48 h incubation time

	ctrl.*	1 µM	5 µM	10 µM	20 µM	40 µM	60 µM	80 µM	100 µM
**OD well 1**	0.26	0.25	0.23	0.23	0.22	0.23	0.23	0.22	0.20
**OD well 2**	0.25	0.23	0.21	0.21	0.21	0.22	0.22	0.23	0.21
**OD well 3**	0.26	0.25	0.23	0.23	0.23	0.23	0.23	0.22	0.19
**MEAN**	0.26	0.24	0.23	0.22	0.22	0.22	0.23	0.22	0.20
**SD**	0.01	0.01	0.01	0.01	0.01	0.01	0.01	0.01	0.01
**REL [%]**	**100.0**	**94.6**	**87.7**	**87.0**	**84.7**	**87.4**	**88.4**	**87.1**	**77.7**
**CV [%]**	3.14	4.21	4.72	5.32	4.72	3.04	2.25	3.93	4.48

* 0.1 % DMSO

72 h incubation time

	ctrl.*	1 µM	5 µM	10 µM	20 µM	40 µM	60 µM	80 µM	100 µM
**OD well 1**	0.56	0.55	0.56	0.57	0.46	0.51	0.55	0.57	0.48
**OD well 2**	0.64	0.61	0.62	0.61	0.52	0.52	0.59	0.63	0.48
**OD well 3**	0.74	0.59	0.61	0.61	0.50	0.53	0.60	0.60	0.50
**MEAN**	0.65	0.58	0.60	0.60	0.49	0.52	0.58	0.60	0.48
**SD**	0.09	0.03	0.03	0.02	0.03	0.01	0.03	0.03	0.01
**REL [%]**	**100.0**	**89.7**	**92.2**	**91.7**	**76.3**	**80.3**	**89.5**	**91.9**	**74.7**
**CV [%]**	13.86	5.49	5.29	3.44	6.64	1.81	4.64	4.87	2.30

* 0.1 % DMSO

120 h incubation time

	ctrl.*	1 µM	5 µM	10 µM	20 µM	40 µM	60 µM	80 µM	100 µM
**OD well 1**	1.70	1.71	1.69	1.53	1.64	1.61	1.65	1.55	1.55
**OD well 2**	1.80	1.68	1.64	1.61	1.58	1.51	1.55	1.41	1.41
**OD well 3**	1.83	1.64	1.81	1.68	1.68	1.54	1.48	1.35	1.38
**MEAN**	1.77	1.68	1.71	1.60	1.64	1.55	1.56	1.44	1.44
**SD**	0.07	0.03	0.09	0.08	0.05	0.06	0.08	0.10	0.09
**REL [%]**	**100.0**	**94.5**	**96.6**	**90.4**	**92.2**	**87.5**	**87.9**	**81.0**	**81.4**
**CV [%]**	3.73	1.99	5.15	4.73	2.95	3.58	5.23	7.13	6.16

* 0.1 % DMSO

Experiment 2

48 h incubation time

	ctrl.*	1 µM	5 µM	10 µM	20 µM	40 µM	60 µM	80 µM	100 µM
**OD well 1**	0.20	0.20	0.19	0.19	0.19	0.19	0.18	0.19	0.19
**OD well 2**	0.22	0.19	0.17	0.17	0.20	0.17	0.18	0.19	0.18
**OD well 3**	0.21	0.21	0.19	0.20	0.20	0.19	0.19	0.20	0.18
**MEAN**	0.21	0.20	0.18	0.19	0.20	0.18	0.18	0.19	0.18
**SD**	0.01	0.01	0.01	0.01	0.01	0.01	0.00	0.01	0.01
**REL [%]**	**100.0**	**95.8**	**88.3**	**90.0**	**95.4**	**88.3**	**89.1**	**93.1**	**89.0**
**CV [%]**	5.35	4.67	5.03	6.75	3.10	6.17	2.00	4.18	3.88

* 0.1 % DMSO

72 h incubation time

	ctrl.*	1 µM	5 µM	10 µM	20 µM	40 µM	60 µM	80 µM	100 µM
**OD well 1**	0.40	0.32	0.32	0.32	0.34	0.35	0.32	0.32	0.30
**OD well 2**	0.39	0.32	0.31	0.33	0.32	0.32	0.28	0.30	0.31
**OD well 3**	0.41	0.32	0.31	0.37	0.31	0.36	0.30	0.34	0.30
**MEAN**	0.40	0.32	0.31	0.34	0.32	0.34	0.30	0.32	0.31
**SD**	0.01	0.00	0.01	0.03	0.01	0.02	0.02	0.02	0.01
**REL [%]**	**100.0**	**79.9**	**77.4**	**84.8**	**80.3**	**84.8**	**75.0**	**79.6**	**76.3**
**CV [%]**	2.05	1.14	2.34	8.86	3.65	5.99	5.92	5.77	2.38

* 0.1 % DMSO

120 h incubation time

	ctrl.*	1 µM	5 µM	10 µM	20 µM	40 µM	60 µM	80 µM	100 µM
**OD well 1**	1.57	1.66	1.67	1.25	1.42	1.47	1.32	1.29	1.32
**OD well 2**	1.48	1.43	1.43	1.16	1.22	1.27	1.32	1.22	1.17
**OD well 3**	1.49	1.38	1.55	1.10	1.22	1.32	1.24	1.32	1.24
**MEAN**	1.51	1.49	1.55	1.17	1.29	1.35	1.29	1.28	1.24
**SD**	0.05	0.15	0.12	0.08	0.11	0.11	0.05	0.05	0.08
**REL [%]**	**100.0**	**98.7**	**102.7**	**77.6**	**85.2**	**89.6**	**85.5**	**84.6**	**82.2**
**CV [%]**	3.28	9.88	7.65	6.56	8.77	7.92	3.60	4.08	6.23

* 0.1 % DMSO

Experiment 3

48 h incubation time

	ctrl.*	1 µM	5 µM	10 µM	20 µM	40 µM	60 µM	80 µM	100 µM
**OD well 1**	0.24	0.23	0.25	0.23	0.23	0.24	0.23	0.23	0.22
**OD well 2**	0.23	0.21	0.23	0.22	0.20	0.22	0.21	0.21	0.21
**OD well 3**	0.23	0.22	0.23	0.24	0.21	0.22	0.22	0.22	0.21
**MEAN**	0.24	0.22	0.24	0.23	0.21	0.23	0.22	0.22	0.21
**SD**	0.01	0.01	0.01	0.01	0.01	0.01	0.01	0.01	0.01
**REL [%]**	**100.0**	**92.8**	**101.0**	**96.7**	**91.0**	**96.8**	**93.1**	**93.1**	**89.9**
**CV [%]**	3.50	4.48	6.14	4.70	6.16	3.10	4.58	3.65	4.91

* 0.1 % DMSO

72 h incubation time

	ctrl.*	1 µM	5 µM	10 µM	20 µM	40 µM	60 µM	80 µM	100 µM
**OD well 1**	0.46	0.44	0.39	0.40	0.37	0.41	0.40	0.40	0.39
**OD well 2**	0.43	0.44	0.37	0.39	0.36	0.40	0.38	0.38	0.38
**OD well 3**	0.51	0.44	0.37	0.34	0.38	0.42	0.40	0.37	0.35
**MEAN**	0.47	0.44	0.38	0.37	0.37	0.41	0.39	0.38	0.37
**SD**	0.04	0.00	0.01	0.03	0.01	0.01	0.01	0.02	0.02
**REL [%]**	**100.0**	**94.1**	**81.4**	**80.0**	**79.4**	**87.6**	**84.0**	**81.8**	**80.3**
**CV [%]**	9.01	0.35	2.53	8.57	3.74	3.17	2.41	4.17	4.96

* 0.1 % DMSO

120 h incubation time

	ctrl.*	1 µM	5 µM	10 µM	20 µM	40 µM	60 µM	80 µM	100 µM
**OD well 1**	1.74	1.68	1.69	1.62	1.63	1.50	1.51	1.42	1.31
**OD well 2**	1.73	1.71	1.58	1.66	1.57	1.43	1.45	1.35	1.36
**OD well 3**	1.68	1.59	1.66	1.63	1.47	1.44	1.41	1.37	1.22
**MEAN**	1.72	1.66	1.65	1.63	1.55	1.46	1.46	1.38	1.30
**SD**	0.03	0.06	0.06	0.02	0.08	0.04	0.05	0.04	0.07
**REL [%]**	**100.0**	**96.7**	**95.9**	**95.3**	**90.6**	**85.0**	**84.9**	**80.5**	**75.6**
**CV [%]**	1.70	3.61	3.56	1.45	5.31	2.77	3.53	2.79	5.37

* 0.1 % DMSO

Experiment 4

48 h incubation time

	ctrl.*	1 µM	5 µM	10 µM	20 µM	40 µM	60 µM	80 µM	100 µM
**OD well 1**	0.22	0.24	0.24	0.23	0.24	0.20	0.21	0.21	0.21
**OD well 2**	0.22	0.23	0.23	0.23	0.24	0.20	0.21	0.20	0.20
**OD well 3**	0.22	0.26	0.24	0.22	0.23	0.21	0.22	0.23	0.21
**MEAN**	0.22	0.24	0.24	0.23	0.24	0.20	0.21	0.21	0.21
**SD**	0.00	0.01	0.01	0.00	0.01	0.01	0.00	0.01	0.00
**REL [%]**	**100.0**	**109.4**	**106.3**	**101.7**	**105.6**	**91.2**	**95.6**	**95.7**	**92.7**
**CV [%]**	0.94	4.69	2.93	1.83	3.30	3.95	1.63	6.81	1.96

* 0.1 % DMSO

72 h incubation time

	ctrl.*	1 µM	5 µM	10 µM	20 µM	40 µM	60 µM	80 µM	100 µM
**OD well 1**	0.53	0.53	0.45	0.54	0.55	0.50	0.48	0.47	0.46
**OD well 2**	0.53	0.49	0.42	0.47	0.52	0.45	0.49	0.47	0.42
**OD well 3**	0.58	0.54	0.44	0.47	0.51	0.43	0.45	0.49	0.43
**MEAN**	0.55	0.52	0.44	0.50	0.53	0.46	0.47	0.48	0.43
**SD**	0.03	0.03	0.01	0.04	0.02	0.04	0.02	0.01	0.02
**REL [%]**	**100.0**	**95.7**	**79.7**	**90.7**	**96.4**	**84.0**	**86.8**	**87.8**	**79.6**
**CV [%]**	5.28	4.85	3.12	8.13	4.30	8.71	4.34	2.69	4.45

* 0.1 % DMSO

120 h incubation time

	ctrl.*	1 µM	5 µM	10 µM	20 µM	40 µM	60 µM	80 µM	100 µM
**OD well 1**	1.87	1.84	1.84	1.69	1.76	1.56	1.71	1.59	1.61
**OD well 2**	1.74	1.76	1.74	1.70	1.57	1.48	1.61	1.57	1.50
**OD well 3**	1.72	1.92	1.88	1.75	1.72	1.53	1.68	1.61	1.51
**MEAN**	1.78	1.84	1.82	1.71	1.68	1.52	1.67	1.59	1.54
**SD**	0.08	0.08	0.07	0.03	0.10	0.04	0.05	0.02	0.06
**REL [%]**	**100.0**	**103.7**	**102.4**	**96.6**	**94.8**	**85.6**	**94.1**	**89.4**	**86.6**
**CV [%]**	4.48	4.48	3.72	1.92	5.93	2.57	3.07	1.23	3.84

* 0.1 % DMSO

Experiment 5

48 h incubation time

	ctrl.*	1 µM	5 µM	10 µM	20 µM	40 µM	60 µM	80 µM	100 µM
**OD well 1**	0.27	0.25	0.26	0.26	0.25	0.26	0.24	0.26	0.27
**OD well 2**	0.27	0.28	0.28	0.27	0.26	0.23	0.25	0.27	0.24
**OD well 3**	0.31	0.33	0.29	0.28	0.28	0.28	0.29	0.29	0.28
**MEAN**	0.28	0.29	0.28	0.27	0.27	0.26	0.26	0.27	0.27
**SD**	0.03	0.04	0.01	0.01	0.02	0.02	0.03	0.02	0.02
**REL [%]**	**100.0**	**101.5**	**98.5**	**95.0**	**93.8**	**91.7**	**90.9**	**96.5**	**93.8**
**CV [%]**	9.46	14.57	4.76	3.03	5.89	8.97	10.37	6.47	7.15

* 0.1 % DMSO

72 h incubation time

	ctrl.*	1 µM	5 µM	10 µM	20 µM	40 µM	60 µM	80 µM	100 µM
**OD well 1**	0.54	0.59	0.56	0.60	0.53	0.51	0.61	0.50	0.46
**OD well 2**	0.54	0.60	0.59	0.56	0.54	0.56	0.53	0.51	0.49
**OD well 3**	0.56	0.61	0.60	0.62	0.51	0.55	0.50	0.50	0.43
**MEAN**	0.55	0.60	0.58	0.59	0.53	0.54	0.55	0.50	0.46
**SD**	0.01	0.01	0.02	0.03	0.01	0.02	0.05	0.01	0.03
**REL [%]**	**100.0**	**109.5**	**106.6**	**108.3**	**96.0**	**98.8**	**99.9**	**91.7**	**83.3**
**CV [%]**	2.41	1.84	4.04	5.50	2.42	4.60	9.70	1.61	6.80

* 0.1 % DMSO

120 h incubation time

	ctrl.*	1 µM	5 µM	10 µM	20 µM	40 µM	60 µM	80 µM	100 µM
**OD well 1**	1.86	1.84	1.66	1.70	1.68	1.57	1.60	1.44	1.45
**OD well 2**	1.89	1.85	1.80	1.75	1.70	1.60	1.48	1.47	1.49
**OD well 3**	1.90	1.96	1.88	1.83	1.80	1.67	1.53	1.56	1.49
**MEAN**	1.88	1.88	1.78	1.76	1.73	1.61	1.54	1.49	1.48
**SD**	0.02	0.07	0.11	0.06	0.07	0.05	0.06	0.06	0.02
**REL [%]**	**100.0**	**100.2**	**94.8**	**93.7**	**91.8**	**85.8**	**81.7**	**79.4**	**78.5**
**CV [%]**	1.15	3.53	6.22	3.62	3.81	3.13	4.01	4.16	1.37

* 0.1 % DMSO

IRA 5

Experiment 1

48 h incubation time

	ctrl.*	1 µM	5 µM	10 µM	20 µM	40 µM	60 µM	80 µM	100 µM
**OD well 1**	0.18	0.19	0.18	0.19	0.15	0.13	0.12	0.11	0.12
**OD well 2**	0.18	0.17	0.18	0.17	0.14	0.12	0.11	0.10	0.11
**OD well 3**	0.18	0.18	0.17	0.18	0.14	0.14	0.13	0.12	0.10
**MEAN**	0.18	0.18	0.18	0.18	0.15	0.13	0.12	0.11	0.11
**SD**	0.00	0.01	0.00	0.01	0.01	0.01	0.01	0.01	0.01
**REL [%]**	**100.0**	**100.0**	**99.0**	**98.5**	**80.9**	**73.5**	**68.1**	**61.5**	**61.3**
**CV [%]**	2.19	3.49	2.33	6.35	4.85	7.09	8.75	4.86	5.46

* 0.1 % DMSO

72 h incubation time

	ctrl.*	1 µM	5 µM	10 µM	20 µM	40 µM	60 µM	80 µM	100 µM
**OD well 1**	0.69	0.46	0.40	0.23	0.18	0.17	0.17	0.15	0.13
**OD well 2**	0.62	0.43	0.37	0.21	0.16	0.16	0.16	0.14	0.13
**OD well 3**	0.64	0.45	0.40	0.23	0.17	0.18	0.17	0.15	0.14
**MEAN**	0.65	0.45	0.39	0.22	0.17	0.17	0.17	0.15	0.13
**SD**	0.04	0.02	0.02	0.01	0.01	0.01	0.01	0.01	0.01
**REL [%]**	**100.0**	**68.7**	**60.4**	**34.5**	**26.2**	**26.2**	**25.4**	**22.6**	**20.1**
**CV [%]**	5.83	3.93	4.14	5.89	6.49	4.44	5.22	4.14	4.61

* 0.1 % DMSO

120 h incubation time

	ctrl.*	1 µM	5 µM	10 µM	20 µM	40 µM	60 µM	80 µM	100 µM
**OD well 1**	1.88	0.74	0.43	0.31	0.21	0.15	0.13	0.13	0.13
**OD well 2**	1.88	0.73	0.35	0.29	0.19	0.17	0.11	0.11	0.10
**OD well 3**	1.88	0.78	0.48	0.24	0.19	0.13	0.10	0.10	0.10
**MEAN**	1.88	0.75	0.42	0.28	0.20	0.15	0.11	0.11	0.11
**SD**	0.00	0.03	0.06	0.03	0.01	0.02	0.02	0.01	0.02
**REL [%]**	**100.0**	**39.8**	**22.3**	**15.0**	**10.5**	**8.1**	**6.0**	**5.8**	**5.8**
**CV [%]**	0.18	4.03	15.30	11.84	5.10	14.91	15.29	13.22	16.78

* 0.1 % DMSO

Experiment 2

48 h incubation time

	ctrl.*	1 µM	5 µM	10 µM	20 µM	40 µM	60 µM	80 µM	100 µM
**OD well 1**	0.25	0.23	0.23	0.21	0.16	0.13	0.11	0.11	0.12
**OD well 2**	0.25	0.26	0.25	0.22	0.19	0.14	0.12	0.12	0.13
**OD well 3**	0.27	0.25	0.26	0.21	0.18	0.15	0.13	0.13	0.13
**MEAN**	0.26	0.25	0.25	0.21	0.17	0.14	0.12	0.12	0.13
**SD**	0.01	0.01	0.01	0.01	0.01	0.01	0.01	0.01	0.00
**REL [%]**	**100.0**	**96.3**	**96.9**	**83.9**	**68.3**	**54.9**	**46.9**	**46.7**	**50.6**
**CV [%]**	3.30	4.76	6.01	3.14	6.88	9.58	11.19	4.98	3.45

* 0.1 % DMSO

72 h incubation time

	ctrl.*	1 µM	5 µM	10 µM	20 µM	40 µM	60 µM	80 µM	100 µM
**OD well 1**	0.66	0.53	0.45	0.30	0.22	0.18	0.16	0.14	0.12
**OD well 2**	0.63	0.54	0.45	0.30	0.24	0.18	0.15	0.14	0.12
**OD well 3**	0.71	0.58	0.41	0.33	0.27	0.18	0.14	0.14	0.14
**MEAN**	0.67	0.55	0.43	0.31	0.24	0.18	0.15	0.14	0.13
**SD**	0.04	0.02	0.02	0.02	0.02	0.00	0.01	0.00	0.01
**REL [%]**	**100.0**	**82.0**	**65.0**	**46.5**	**36.4**	**26.8**	**22.7**	**21.1**	**19.0**
**CV [%]**	6.09	4.51	5.34	6.58	9.41	2.39	5.52	2.79	8.79

* 0.1 % DMSO

120 h incubation time

	ctrl.*	1 µM	5 µM	10 µM	20 µM	40 µM	60 µM	80 µM	100 µM
**OD well 1**	2.09	0.89	0.60	0.35	0.22	0.16	0.11	0.10	0.10
**OD well 2**	2.10	0.87	0.63	0.42	0.24	0.14	0.14	0.13	0.11
**OD well 3**	2.14	0.89	0.64	0.38	0.23	0.18	0.12	0.13	0.10
**MEAN**	2.11	0.88	0.62	0.38	0.23	0.16	0.12	0.12	0.10
**SD**	0.03	0.02	0.02	0.04	0.01	0.02	0.01	0.02	0.01
**REL [%]**	**100.0**	**41.9**	**29.5**	**18.2**	**10.8**	**7.6**	**5.9**	**5.7**	**4.9**
**CV [%]**	1.26	1.72	3.66	9.41	4.57	14.89	11.52	12.84	7.69

* 0.1 % DMSO

Experiment 3

48 h incubation time

	ctrl.*	1 µM	5 µM	10 µM	20 µM	40 µM	60 µM	80 µM	100 µM
**OD well 1**	0.26	0.23	0.21	0.18	0.16	0.15	0.12	0.12	0.11
**OD well 2**	0.23	0.22	0.19	0.17	0.16	0.15	0.11	0.11	0.11
**OD well 3**	0.26	0.23	0.20	0.17	0.17	0.16	0.13	0.13	0.11
**MEAN**	0.25	0.22	0.20	0.17	0.16	0.15	0.12	0.12	0.11
**SD**	0.02	0.01	0.01	0.01	0.00	0.01	0.01	0.01	0.00
**REL [%]**	**100.0**	**88.9**	**79.3**	**68.2**	**64.0**	**60.8**	**48.0**	**47.6**	**43.2**
**CV [%]**	7.14	4.17	2.92	4.26	2.66	5.19	7.75	7.72	0.75

* 0.1 % DMSO

72 h incubation time

	ctrl.*	1 µM	5 µM	10 µM	20 µM	40 µM	60 µM	80 µM	100 µM
**OD well 1**	0.52	0.38	0.30	0.21	0.17	0.16	0.12	0.11	0.10
**OD well 2**	0.47	0.34	0.25	0.19	0.16	0.16	0.13	0.11	0.11
**OD well 3**	0.53	0.38	0.28	0.21	0.18	0.17	0.14	0.12	0.12
**MEAN**	0.51	0.37	0.27	0.21	0.17	0.16	0.13	0.11	0.11
**SD**	0.03	0.03	0.03	0.01	0.01	0.01	0.01	0.01	0.01
**REL [%]**	**100.0**	**71.8**	**53.9**	**40.3**	**33.6**	**31.6**	**25.3**	**22.2**	**21.6**
**CV [%]**	6.06	7.15	9.21	6.67	3.70	5.03	8.38	6.02	5.37

* 0.1 % DMSO

120 h incubation time

	ctrl.*	1 µM	5 µM	10 µM	20 µM	40 µM	60 µM	80 µM	100 µM
**OD well 1**	1.98	0.82	0.62	0.26	0.27	0.19	0.14	0.12	0.10
**OD well 2**	1.97	0.82	0.58	0.23	0.23	0.20	0.12	0.11	0.10
**OD well 3**	2.12	0.83	0.61	0.23	0.24	0.18	0.13	0.13	0.11
**MEAN**	2.02	0.82	0.60	0.24	0.25	0.19	0.13	0.12	0.11
**SD**	0.08	0.01	0.02	0.02	0.02	0.01	0.01	0.01	0.01
**REL [%]**	**100.0**	**40.7**	**29.8**	**11.9**	**12.2**	**9.3**	**6.4**	**6.1**	**5.2**
**CV [%]**	4.10	0.75	3.59	8.42	6.85	3.57	7.87	8.38	5.77

* 0.1 % DMSO

Experiment 4

48 h incubation time

	ctrl.*	1 µM	5 µM	10 µM	20 µM	40 µM	60 µM	80 µM	100 µM
**OD well 1**	0.24	0.22	0.19	0.18	0.16	0.15	0.12	0.11	0.12
**OD well 2**	0.23	0.22	0.20	0.18	0.15	0.12	0.12	0.11	0.12
**OD well 3**	0.23	0.25	0.21	0.19	0.16	0.14	0.12	0.12	0.11
**MEAN**	0.24	0.23	0.20	0.19	0.16	0.14	0.12	0.11	0.11
**SD**	0.01	0.02	0.01	0.00	0.01	0.01	0.00	0.01	0.01
**REL [%]**	**100.0**	**96.1**	**85.5**	**79.0**	**67.8**	**58.3**	**51.0**	**47.9**	**48.7**
**CV [%]**	2.94	7.22	4.66	1.82	3.69	9.51	1.46	7.00	5.65

* 0.1 % DMSO

72 h incubation time

	ctrl.*	1 µM	5 µM	10 µM	20 µM	40 µM	60 µM	80 µM	100 µM
**OD well 1**	0.64	0.48	0.34	0.30	0.22	0.18	0.15	0.16	0.15
**OD well 2**	0.65	0.49	0.34	0.27	0.21	0.16	0.15	0.13	0.11
**OD well 3**	0.56	0.43	0.35	0.25	0.21	0.19	0.14	0.13	0.13
**MEAN**	0.62	0.47	0.34	0.27	0.21	0.18	0.14	0.14	0.13
**SD**	0.05	0.03	0.01	0.02	0.01	0.01	0.01	0.02	0.02
**REL [%]**	**100.0**	**75.7**	**55.4**	**44.4**	**34.6**	**28.8**	**23.5**	**22.5**	**21.0**
**CV [%]**	8.17	6.74	2.26	7.92	3.41	8.21	3.99	12.57	12.52

* 0.1 % DMSO

120 h incubation time

	ctrl.*	1 µM	5 µM	10 µM	20 µM	40 µM	60 µM	80 µM	100 µM
**OD well 1**	2.12	0.85	0.59	0.32	0.20	0.15	0.12	0.10	0.13
**OD well 2**	2.01	0.85	0.61	0.31	0.21	0.15	0.11	0.10	0.12
**OD well 3**	2.11	0.85	0.57	0.29	0.20	0.15	0.12	0.10	0.10
**MEAN**	2.08	0.85	0.59	0.30	0.20	0.15	0.11	0.10	0.11
**SD**	0.06	0.00	0.02	0.01	0.01	0.00	0.01	0.00	0.02
**REL [%]**	**100.0**	**40.8**	**28.3**	**14.6**	**9.8**	**7.2**	**5.5**	**4.8**	**5.5**
**CV [%]**	2.99	0.12	3.23	3.74	2.49	2.95	6.12	0.23	13.95

* 0.1 % DMSO

Experiment 5

48 h incubation time

	ctrl.*	1 µM	5 µM	10 µM	20 µM	40 µM	60 µM	80 µM	100 µM
**OD well 1**	0.27	0.23	0.23	0.19	0.17	0.15	0.13	0.13	0.13
**OD well 2**	0.24	0.21	0.19	0.19	0.14	0.14	0.12	0.12	0.12
**OD well 3**	0.24	0.23	0.19	0.18	0.16	0.15	0.13	0.13	0.12
**MEAN**	0.25	0.23	0.20	0.19	0.16	0.15	0.12	0.13	0.12
**SD**	0.02	0.01	0.02	0.01	0.02	0.01	0.00	0.01	0.01
**REL [%]**	**100.0**	**90.2**	**80.8**	**75.4**	**62.2**	**58.7**	**49.8**	**50.9**	**48.4**
**CV [%]**	6.60	4.33	12.25	2.90	10.20	4.41	3.81	4.90	6.75

* 0.1 % DMSO

72 h incubation time

	ctrl.*	1 µM	5 µM	10 µM	20 µM	40 µM	60 µM	80 µM	100 µM
**OD well 1**	0.64	0.46	0.36	0.30	0.22	0.17	0.14	0.13	0.12
**OD well 2**	0.60	0.44	0.33	0.25	0.20	0.16	0.12	0.11	0.13
**OD well 3**	0.60	0.47	0.36	0.27	0.22	0.17	0.13	0.12	0.11
**MEAN**	0.61	0.46	0.35	0.27	0.22	0.17	0.13	0.12	0.12
**SD**	0.03	0.02	0.01	0.02	0.02	0.01	0.01	0.01	0.01
**REL [%]**	**100.0**	**74.2**	**56.7**	**44.6**	**35.1**	**27.3**	**21.2**	**19.8**	**19.3**
**CV [%]**	4.31	4.20	3.86	8.53	7.08	3.03	7.95	10.79	9.56

* 0.1 % DMSO

120 h incubation time

	ctrl.*	1 µM	5 µM	10 µM	20 µM	40 µM	60 µM	80 µM	100 µM
**OD well 1**	1.91	0.91	0.44	0.27	0.20	0.14	0.13	0.12	0.11
**OD well 2**	1.88	0.82	0.45	0.25	0.19	0.14	0.11	0.10	0.10
**OD well 3**	1.94	0.80	0.47	0.25	0.19	0.15	0.11	0.11	0.11
**MEAN**	1.91	0.84	0.45	0.26	0.19	0.15	0.12	0.11	0.11
**SD**	0.03	0.06	0.01	0.01	0.01	0.01	0.01	0.01	0.01
**REL [%]**	**100.0**	**44.1**	**23.7**	**13.4**	**10.1**	**7.6**	**6.2**	**5.8**	**5.5**
**CV [%]**	1.54	6.92	3.06	4.45	3.75	3.58	5.76	5.90	7.55

* 0.1 % DMSO

A-431 cells

Resveratrol

Experiment 1

48 h incubation time

	ctrl.*	1 µM	5 µM	10 µM	20 µM	40 µM	60 µM	80 µM	100 µM
**OD well 1**	0.22	0.24	0.23	0.20	0.16	0.13	0.13	0.12	0.12
**OD well 2**	0.23	0.24	0.23	0.21	0.15	0.13	0.13	0.13	0.13
**OD well 3**	0.23	0.23	0.23	0.19	0.14	0.13	0.11	0.12	0.12
**MEAN**	0.23	0.24	0.23	0.20	0.15	0.13	0.12	0.12	0.13
**SD**	0.00	0.01	0.00	0.01	0.01	0.00	0.01	0.00	0.01
**REL [%]**	**100.0**	**104.5**	**100.8**	**88.6**	**66.0**	**57.0**	**55.2**	**54.6**	**55.5**
**CV [%]**	1.61	3.71	0.38	3.00	8.60	0.92	9.59	2.92	5.09

* 0.1 % DMSO

72 h incubation time

	ctrl.*	1 µM	5 µM	10 µM	20 µM	40 µM	60 µM	80 µM	100 µM
**OD well 1**	0.41	0.41	0.37	0.27	0.16	0.12	0.13	0.12	0.12
**OD well 2**	0.40	0.41	0.35	0.25	0.15	0.11	0.11	0.10	0.10
**OD well 3**	0.39	0.44	0.38	0.27	0.15	0.12	0.12	0.14	0.13
**MEAN**	0.40	0.42	0.36	0.26	0.15	0.12	0.12	0.12	0.11
**SD**	0.01	0.02	0.02	0.01	0.01	0.01	0.01	0.02	0.02
**REL [%]**	**100.0**	**104.5**	**91.1**	**65.5**	**38.6**	**29.3**	**29.8**	**30.0**	**28.7**
**CV [%]**	1.82	4.20	4.37	4.92	3.79	7.29	7.66	16.23	13.71

* 0.1 % DMSO

120 h incubation time

	ctrl.*	1 µM	5 µM	10 µM	20 µM	40 µM	60 µM	80 µM	100 µM
**OD well 1**	1.12	1.12	0.92	0.62	0.15	0.11	0.12	0.12	0.12
**OD well 2**	1.18	1.24	0.92	0.55	0.14	0.10	0.12	0.10	0.10
**OD well 3**	1.06	1.14	0.82	0.53	0.12	0.09	0.11	0.10	0.10
**MEAN**	1.12	1.17	0.89	0.57	0.14	0.10	0.11	0.11	0.11
**SD**	0.06	0.06	0.06	0.05	0.01	0.01	0.01	0.01	0.01
**REL [%]**	**100.0**	**104.5**	**79.4**	**50.9**	**12.4**	**8.8**	**10.2**	**9.5**	**9.7**
**CV [%]**	5.38	5.52	6.75	8.87	8.94	8.76	7.57	8.47	10.54

* 0.1 % DMSO

Experiment 2

48 h incubation time

	ctrl.*	1 µM	5 µM	10 µM	20 µM	40 µM	60 µM	80 µM	100 µM
**OD well 1**	0.22	0.19	0.20	0.20	0.13	0.13	0.13	0.13	0.12
**OD well 2**	0.21	0.19	0.19	0.20	0.13	0.12	0.13	0.13	0.11
**OD well 3**	0.21	0.18	0.20	0.19	0.13	0.13	0.14	0.13	0.12
**MEAN**	0.21	0.19	0.20	0.20	0.13	0.12	0.13	0.13	0.12
**SD**	0.00	0.00	0.00	0.01	0.00	0.00	0.01	0.00	0.01
**REL [%]**	**100.0**	**87.0**	**92.2**	**92.2**	**59.6**	**57.8**	**62.0**	**61.4**	**54.6**
**CV [%]**	2.16	2.46	2.28	3.75	0.68	2.48	3.77	1.50	4.60

* 0.1 % DMSO

72 h incubation time

	ctrl.*	1 µM	5 µM	10 µM	20 µM	40 µM	60 µM	80 µM	100 µM
**OD well 1**	0.40	0.37	0.41	0.29	0.14	0.10	0.11	0.10	0.09
**OD well 2**	0.37	0.37	0.38	0.30	0.13	0.10	0.09	0.10	0.09
**OD well 3**	0.37	0.37	0.37	0.31	0.14	0.09	0.11	0.10	0.09
**MEAN**	0.38	0.37	0.39	0.30	0.14	0.10	0.10	0.10	0.09
**SD**	0.02	0.00	0.02	0.01	0.00	0.00	0.01	0.00	0.00
**REL [%]**	**100.0**	**96.4**	**101.9**	**78.3**	**36.3**	**25.6**	**26.7**	**25.6**	**24.0**
**CV [%]**	4.75	0.94	5.42	3.03	2.69	4.04	10.84	2.75	4.51

* 0.1 % DMSO

120 h incubation time

	ctrl.*	1 µM	5 µM	10 µM	20 µM	40 µM	60 µM	80 µM	100 µM
**OD well 1**	1.05	1.11	0.88	0.54	0.12	0.11	0.12	0.11	0.12
**OD well 2**	1.06	1.05	0.83	0.59	0.10	0.09	0.10	0.10	0.10
**OD well 3**	1.02	1.13	0.89	0.53	0.12	0.11	0.11	0.13	0.12
**MEAN**	1.04	1.09	0.87	0.55	0.11	0.10	0.11	0.11	0.11
**SD**	0.02	0.04	0.03	0.03	0.01	0.01	0.01	0.01	0.01
**REL [%]**	**100.0**	**105.0**	**83.1**	**53.2**	**10.8**	**9.8**	**10.4**	**10.8**	**10.8**
**CV [%]**	2.07	3.81	3.73	6.02	6.37	7.48	10.76	10.90	10.92

* 0.1 % DMSO

Experiment 3

48 h incubation time

	ctrl.*	1 µM	5 µM	10 µM	20 µM	40 µM	60 µM	80 µM	100 µM
**OD well 1**	0.24	0.27	0.27	0.21	0.16	0.15	0.13	0.12	0.13
**OD well 2**	0.25	0.26	0.24	0.20	0.15	0.15	0.13	0.11	0.09
**OD well 3**	0.25	0.26	0.24	0.19	0.15	0.14	0.11	0.11	0.12
**MEAN**	0.24	0.26	0.25	0.20	0.15	0.15	0.12	0.11	0.11
**SD**	0.01	0.01	0.01	0.01	0.00	0.00	0.01	0.01	0.02
**REL [%]**	**100.0**	**106.9**	**102.8**	**82.8**	**63.0**	**60.1**	**50.3**	**46.3**	**46.2**
**CV [%]**	2.68	2.65	5.80	4.69	2.32	3.05	9.46	7.98	14.67

* 0.1 % DMSO

72 h incubation time

	ctrl.*	1 µM	5 µM	10 µM	20 µM	40 µM	60 µM	80 µM	100 µM
**OD well 1**	0.46	0.43	0.39	0.27	0.15	0.11	0.12	0.11	0.11
**OD well 2**	0.42	0.43	0.35	0.25	0.14	0.11	0.11	0.10	0.10
**OD well 3**	0.42	0.45	0.37	0.27	0.15	0.12	0.11	0.12	0.11
**MEAN**	0.43	0.44	0.37	0.27	0.15	0.11	0.11	0.11	0.11
**SD**	0.02	0.01	0.02	0.01	0.00	0.00	0.00	0.01	0.01
**REL [%]**	**100.0**	**101.0**	**85.3**	**61.4**	**34.4**	**26.4**	**26.6**	**26.1**	**25.1**
**CV [%]**	4.94	3.43	5.77	4.09	2.91	1.44	3.41	9.35	6.32

* 0.1 % DMSO

120 h incubation time

	ctrl.*	1 µM	5 µM	10 µM	20 µM	40 µM	60 µM	80 µM	100 µM
**OD well 1**	1.13	1.21	0.92	0.43	0.11	0.11	0.10	0.10	0.11
**OD well 2**	1.15	1.18	0.86	0.42	0.14	0.12	0.12	0.10	0.12
**OD well 3**	1.14	1.10	0.79	0.36	0.12	0.12	0.11	0.12	0.11
**MEAN**	1.14	1.16	0.86	0.40	0.12	0.12	0.11	0.11	0.11
**SD**	0.01	0.06	0.06	0.04	0.01	0.01	0.01	0.01	0.01
**REL [%]**	**100.0**	**101.9**	**75.3**	**35.4**	**10.5**	**10.3**	**9.6**	**9.6**	**9.8**
**CV [%]**	1.09	4.76	7.39	8.71	11.44	7.30	6.11	9.19	6.00

* 0.1 % DMSO

Experiment 4

48 h incubation time

	**ctrl.***	**1 µM**	**5 µM**	**10 µM**	**20 µM**	**40 µM**	**60 µM**	**80 µM**	**100 µM**
**OD well 1**	0.22	0.23	0.23	0.21	0.18	0.14	0.13	0.12	0.14
**OD well 2**	0.23	0.23	0.22	0.21	0.16	0.14	0.16	0.14	0.13
**OD well 3**	0.24	0.23	0.22	0.21	0.16	0.14	0.13	0.12	0.13
**MEAN**	0.23	0.23	0.22	0.21	0.17	0.14	0.14	0.13	0.13
**SD**	0.01	0.00	0.00	0.00	0.01	0.00	0.02	0.01	0.01
**REL [%]**	**100.0**	**100.7**	**96.6**	**91.0**	**72.3**	**60.9**	**60.2**	**54.8**	**57.9**
**CV [%]**	4.16	1.45	1.51	0.45	7.20	2.19	11.83	9.05	5.76

* 0.1 % DMSO

72 h incubation time

	**ctrl.***	**1 µM**	**5 µM**	**10 µM**	**20 µM**	**40 µM**	**60 µM**	**80 µM**	**100 µM**
**OD well 1**	0.38	0.43	0.34	0.24	0.17	0.13	0.12	0.13	0.11
**OD well 2**	0.42	0.42	0.36	0.24	0.16	0.12	0.12	0.12	0.11
**OD well 3**	0.41	0.41	0.31	0.24	0.16	0.11	0.11	0.12	0.13
**MEAN**	0.40	0.42	0.34	0.24	0.17	0.12	0.11	0.12	0.11
**SD**	0.02	0.01	0.02	0.00	0.01	0.01	0.01	0.00	0.01
**REL [%]**	**100.0**	**104.1**	**83.9**	**59.4**	**41.4**	**30.0**	**28.4**	**30.8**	**28.5**
**CV [%]**	4.38	2.59	6.26	1.55	3.88	10.51	7.25	1.66	9.70

* 0.1 % DMSO

120 h incubation time

	**ctrl.***	**1 µM**	**5 µM**	**10 µM**	**20 µM**	**40 µM**	**60 µM**	**80 µM**	**100 µM**
**OD well 1**	1.13	1.15	0.88	0.46	0.17	0.12	0.13	0.13	0.12
**OD well 2**	1.19	1.08	0.92	0.44	0.17	0.13	0.12	0.11	0.10
**OD well 3**	1.14	1.06	0.89	0.45	0.17	0.11	0.11	0.11	0.11
**MEAN**	1.15	1.10	0.90	0.45	0.17	0.12	0.12	0.12	0.11
**SD**	0.04	0.05	0.02	0.01	0.00	0.01	0.01	0.01	0.01
**REL [%]**	**100.0**	**95.1**	**77.9**	**39.1**	**14.7**	**10.5**	**10.2**	**10.2**	**9.4**
**CV [%]**	3.04	4.32	2.33	2.36	1.53	7.77	6.30	10.91	7.07

* 0.1 % DMSO

Experiment 5

48 h incubation time

	ctrl.*	1 µM	5 µM	10 µM	20 µM	40 µM	60 µM	80 µM	100 µM
**OD well 1**	0.24	0.25	0.22	0.20	0.17	0.13	0.13	0.12	0.14
**OD well 2**	0.25	0.25	0.23	0.21	0.16	0.14	0.15	0.14	0.13
**OD well 3**	0.26	0.24	0.24	0.21	0.15	0.13	0.13	0.13	0.13
**MEAN**	0.25	0.25	0.23	0.21	0.16	0.14	0.14	0.13	0.13
**SD**	0.01	0.00	0.01	0.01	0.01	0.01	0.01	0.01	0.01
**REL [%]**	**100.0**	**100.2**	**92.7**	**83.1**	**64.0**	**54.7**	**55.2**	**52.4**	**52.9**
**CV [%]**	3.25	2.01	3.12	3.99	7.22	3.97	9.35	8.12	4.14

* 0.1 % DMSO

72 h incubation time

	ctrl.*	1 µM	5 µM	10 µM	20 µM	40 µM	60 µM	80 µM	100 µM
**OD well 1**	0.43	0.47	0.38	0.24	0.17	0.14	0.12	0.14	0.12
**OD well 2**	0.44	0.42	0.34	0.21	0.16	0.13	0.13	0.12	0.11
**OD well 3**	0.40	0.40	0.35	0.23	0.17	0.12	0.11	0.14	0.15
**MEAN**	0.42	0.43	0.36	0.23	0.17	0.13	0.12	0.13	0.12
**SD**	0.02	0.04	0.02	0.01	0.00	0.01	0.01	0.01	0.02
**REL [%]**	**100.0**	**101.7**	**84.3**	**53.3**	**39.1**	**30.6**	**28.5**	**31.1**	**29.5**
**CV [%]**	4.11	8.59	6.49	5.59	2.48	8.67	6.46	5.07	15.45

* 0.1 % DMSO

120 h incubation time

	ctrl.*	1 µM	5 µM	10 µM	20 µM	40 µM	60 µM	80 µM	100 µM
**OD well 1**	1.19	1.21	0.93	0.55	0.16	0.13	0.12	0.13	0.12
**OD well 2**	1.13	1.11	0.90	0.48	0.18	0.12	0.12	0.11	0.10
**OD well 3**	1.10	1.17	0.89	0.51	0.18	0.12	0.12	0.11	0.10
**MEAN**	1.14	1.16	0.91	0.51	0.17	0.12	0.12	0.12	0.11
**SD**	0.05	0.05	0.02	0.04	0.01	0.00	0.01	0.01	0.01
**REL [%]**	**100.0**	**101.9**	**79.4**	**44.8**	**15.1**	**10.8**	**10.6**	**10.4**	**9.6**
**CV [%]**	4.30	4.22	1.94	7.23	5.38	2.52	4.12	10.62	9.99

* 0.1 % DMSO

IRA 5

Experiment 1

48 h incubation time

	ctrl.*	1 µM	5 µM	10 µM	20 µM	40 µM	60 µM	80 µM	100 µM
**OD well 1**	0.20	0.22	0.22	0.20	0.19	0.16	0.15	0.14	0.13
**OD well 2**	0.21	0.21	0.21	0.20	0.19	0.16	0.15	0.14	0.13
**OD well 3**	0.21	0.20	0.21	0.20	0.20	0.16	0.14	0.14	0.12
**MEAN**	0.21	0.21	0.21	0.20	0.20	0.16	0.15	0.14	0.13
**SD**	0.00	0.01	0.01	0.00	0.01	0.00	0.00	0.00	0.01
**REL [%]**	**100.0**	**102.2**	**104.0**	**98.7**	**95.2**	**77.0**	**70.5**	**67.8**	**62.5**
**CV [%]**	0.69	3.33	2.35	0.20	3.60	0.39	1.38	2.79	4.35

* 0.1 % DMSO

72 h incubation time

	ctrl.*	1 µM	5 µM	10 µM	20 µM	40 µM	60 µM	80 µM	100 µM
**OD well 1**	0.39	0.38	0.36	0.32	0.23	0.16	0.15	0.15	0.12
**OD well 2**	0.39	0.38	0.35	0.31	0.23	0.17	0.14	0.14	0.12
**OD well 3**	0.38	0.43	0.36	0.32	0.25	0.17	0.15	0.14	0.12
**MEAN**	0.39	0.40	0.35	0.32	0.24	0.17	0.15	0.14	0.12
**SD**	0.00	0.03	0.01	0.01	0.01	0.00	0.01	0.01	0.00
**REL [%]**	**100.0**	**102.7**	**91.6**	**82.6**	**61.6**	**43.5**	**38.7**	**36.4**	**31.2**
**CV [%]**	1.28	7.67	2.60	2.33	4.90	2.62	3.90	3.55	0.99

* 0.1 % DMSO

120 h incubation time

	ctrl.*	1 µM	5 µM	10 µM	20 µM	40 µM	60 µM	80 µM	100 µM
**OD well 1**	1.13	1.12	0.97	0.77	0.48	0.19	0.16	0.14	0.12
**OD well 2**	1.03	1.05	0.87	0.72	0.48	0.18	0.16	0.13	0.12
**OD well 3**	1.09	1.15	1.01	0.79	0.59	0.18	0.15	0.14	0.12
**MEAN**	1.08	1.11	0.95	0.76	0.52	0.18	0.16	0.14	0.12
**SD**	0.05	0.05	0.07	0.04	0.06	0.01	0.00	0.01	0.00
**REL [%]**	**100.0**	**102.3**	**87.6**	**70.2**	**47.8**	**17.0**	**14.5**	**12.8**	**11.0**
**CV [%]**	4.68	4.39	7.51	5.02	12.21	3.86	2.18	6.54	2.13

* 0.1 % DMSO

Experiment 2

48 h incubation time

	ctrl.*	1 µM	5 µM	10 µM	20 µM	40 µM	60 µM	80 µM	100 µM
**OD well 1**	0.22	0.22	0.23	0.20	0.19	0.15	0.16	0.13	0.13
**OD well 2**	0.24	0.22	0.21	0.21	0.20	0.14	0.15	0.15	0.12
**OD well 3**	0.23	0.25	0.23	0.23	0.20	0.17	0.15	0.15	0.13
**MEAN**	0.23	0.23	0.22	0.22	0.20	0.16	0.16	0.14	0.13
**SD**	0.01	0.02	0.01	0.01	0.01	0.02	0.00	0.01	0.00
**REL [%]**	**100.0**	**99.5**	**96.3**	**93.9**	**86.3**	**67.2**	**67.3**	**61.8**	**54.9**
**CV [%]**	2.77	6.82	3.62	6.56	4.73	10.21	2.66	6.65	3.17

* 0.1 % DMSO

72 h incubation time

	ctrl.*	1 µM	5 µM	10 µM	20 µM	40 µM	60 µM	80 µM	100 µM
**OD well 1**	0.41	0.41	0.38	0.32	0.25	0.17	0.15	0.14	0.11
**OD well 2**	0.40	0.44	0.41	0.33	0.26	0.15	0.15	0.13	0.10
**OD well 3**	0.41	0.44	0.43	0.33	0.26	0.16	0.17	0.14	0.13
**MEAN**	0.41	0.43	0.40	0.33	0.26	0.16	0.16	0.14	0.11
**SD**	0.01	0.02	0.02	0.00	0.00	0.01	0.01	0.01	0.01
**REL [%]**	**100.0**	**105.9**	**99.2**	**80.6**	**62.9**	**40.2**	**38.6**	**34.0**	**27.5**
**CV [%]**	1.91	3.63	6.09	1.33	1.24	5.65	8.78	3.87	10.99

* 0.1 % DMSO

120 h incubation time

	ctrl.*	1 µM	5 µM	10 µM	20 µM	40 µM	60 µM	80 µM	100 µM
**OD well 1**	1.02	1.06	1.00	0.65	0.35	0.16	0.14	0.14	0.13
**OD well 2**	1.04	1.05	0.93	0.58	0.38	0.16	0.15	0.13	0.14
**OD well 3**	1.11	1.01	0.90	0.60	0.46	0.19	0.16	0.15	0.13
**MEAN**	1.05	1.04	0.94	0.61	0.40	0.17	0.15	0.14	0.13
**SD**	0.05	0.03	0.05	0.04	0.06	0.02	0.01	0.01	0.01
**REL [%]**	**100.0**	**98.3**	**89.4**	**58.1**	**37.5**	**16.2**	**14.2**	**13.1**	**12.4**
**CV [%]**	4.32	2.59	5.51	5.86	14.07	10.87	7.77	6.64	4.61

* 0.1 % DMSO

Experiment 3

48 h incubation time

	ctrl.*	1 µM	5 µM	10 µM	20 µM	40 µM	60 µM	80 µM	100 µM
**OD well 1**	0.24	0.23	0.24	0.23	0.22	0.17	0.17	0.14	0.12
**OD well 2**	0.23	0.25	0.23	0.23	0.19	0.18	0.16	0.15	0.13
**OD well 3**	0.26	0.26	0.24	0.26	0.21	0.18	0.17	0.16	0.15
**MEAN**	0.25	0.25	0.24	0.24	0.21	0.18	0.17	0.15	0.14
**SD**	0.02	0.01	0.01	0.02	0.02	0.01	0.01	0.01	0.02
**REL [%]**	**100.0**	**99.8**	**95.9**	**96.8**	**83.6**	**71.6**	**68.3**	**61.7**	**55.0**
**CV [%]**	6.16	5.45	3.56	6.84	7.87	4.62	3.57	7.53	11.73

* 0.1 % DMSO

72 h incubation time

	ctrl.*	1 µM	5 µM	10 µM	20 µM	40 µM	60 µM	80 µM	100 µM
**OD well 1**	0.43	0.43	0.40	0.34	0.23	0.16	0.16	0.15	0.13
**OD well 2**	0.44	0.42	0.38	0.32	0.24	0.17	0.15	0.14	0.13
**OD well 3**	0.40	0.45	0.39	0.33	0.25	0.17	0.14	0.14	0.13
**MEAN**	0.42	0.44	0.39	0.33	0.24	0.17	0.15	0.14	0.13
**SD**	0.02	0.01	0.01	0.01	0.01	0.00	0.01	0.00	0.00
**REL [%]**	**100.0**	**103.4**	**92.6**	**78.7**	**56.8**	**39.9**	**35.2**	**33.7**	**30.1**
**CV [%]**	5.39	3.03	1.87	3.54	5.46	2.90	4.85	3.15	0.95

* 0.1 % DMSO

120 h incubation time

	ctrl.*	1 µM	5 µM	10 µM	20 µM	40 µM	60 µM	80 µM	100 µM
**OD well 1**	1.14	1.19	0.99	0.62	0.37	0.17	0.14	0.14	0.13
**OD well 2**	1.03	1.11	1.01	0.61	0.33	0.16	0.13	0.13	0.13
**OD well 3**	1.04	1.17	0.88	0.52	0.34	0.16	0.15	0.13	0.13
**MEAN**	1.07	1.16	0.96	0.58	0.35	0.16	0.14	0.14	0.13
**SD**	0.06	0.04	0.07	0.06	0.02	0.01	0.01	0.01	0.00
**REL [%]**	**100.0**	**108.4**	**89.9**	**54.6**	**32.5**	**15.1**	**13.1**	**12.8**	**12.0**
**CV [%]**	5.98	3.48	7.24	9.85	6.09	4.91	6.05	5.18	1.18

* 0.1 % DMSO

Experiment 4

48 h incubation time

	ctrl.*	1 µM	5 µM	10 µM	20 µM	40 µM	60 µM	80 µM	100 µM
**OD well 1**	0.23	0.23	0.20	0.21	0.20	0.15	0.15	0.15	0.15
**OD well 2**	0.23	0.23	0.21	0.22	0.21	0.17	0.16	0.15	0.13
**OD well 3**	0.24	0.23	0.21	0.22	0.20	0.18	0.17	0.16	0.14
**MEAN**	0.23	0.23	0.21	0.22	0.20	0.17	0.16	0.15	0.14
**SD**	0.01	0.00	0.01	0.01	0.00	0.01	0.01	0.00	0.01
**REL [%]**	**100.0**	**99.3**	**90.5**	**93.8**	**87.8**	**72.9**	**69.1**	**66.1**	**60.5**
**CV [%]**	2.67	1.36	3.15	2.96	2.03	8.29	5.48	1.81	7.01

* 0.1 % DMSO

72 h incubation time

	ctrl.*	1 µM	5 µM	10 µM	20 µM	40 µM	60 µM	80 µM	100 µM
**OD well 1**	0.37	0.38	0.36	0.34	0.33	0.17	0.14	0.13	0.14
**OD well 2**	0.38	0.40	0.38	0.38	0.34	0.17	0.15	0.13	0.14
**OD well 3**	0.41	0.41	0.39	0.37	0.34	0.18	0.14	0.14	0.11
**MEAN**	0.39	0.40	0.38	0.36	0.34	0.17	0.15	0.13	0.13
**SD**	0.02	0.01	0.01	0.02	0.00	0.01	0.01	0.00	0.01
**REL [%]**	**100.0**	**103.0**	**97.9**	**93.0**	**86.7**	**45.2**	**37.7**	**34.7**	**33.5**
**CV [%]**	5.75	3.22	3.76	5.54	1.10	4.69	3.82	3.24	10.89

* 0.1 % DMSO

120 h incubation time

	ctrl.*	1 µM	5 µM	10 µM	20 µM	40 µM	60 µM	80 µM	100 µM
**OD well 1**	1.21	1.14	1.08	0.77	0.45	0.20	0.17	0.15	0.11
**OD well 2**	1.10	1.16	1.09	0.77	0.46	0.20	0.15	0.15	0.12
**OD well 3**	1.28	1.20	1.04	0.77	0.47	0.24	0.16	0.13	0.12
**MEAN**	1.20	1.17	1.07	0.77	0.46	0.21	0.16	0.14	0.12
**SD**	0.09	0.03	0.03	0.00	0.01	0.02	0.01	0.01	0.00
**REL [%]**	**100.0**	**97.3**	**89.1**	**64.2**	**38.3**	**17.8**	**13.3**	**11.9**	**9.8**
**CV [%]**	7.51	2.41	2.42	0.31	2.63	9.60	7.66	5.84	3.85

* 0.1 % DMSO

Experiment 5

48 h incubation time

	ctrl.*	1 µM	5 µM	10 µM	20 µM	40 µM	60 µM	80 µM	100 µM
**OD well 1**	0.23	0.23	0.23	0.22	0.22	0.17	0.16	0.16	0.14
**OD well 2**	0.24	0.23	0.23	0.22	0.22	0.18	0.16	0.14	0.14
**OD well 3**	0.26	0.23	0.24	0.22	0.21	0.19	0.16	0.16	0.13
**MEAN**	0.24	0.23	0.24	0.22	0.22	0.18	0.16	0.15	0.14
**SD**	0.01	0.00	0.00	0.00	0.00	0.01	0.00	0.01	0.00
**REL [%]**	**100.0**	**95.9**	**97.1**	**91.7**	**88.8**	**72.8**	**65.6**	**62.0**	**56.4**
**CV [%]**	6.10	0.43	1.08	0.70	0.65	5.63	1.72	7.82	2.99

* 0.1 % DMSO

72 h incubation time

	ctrl.*	1 µM	5 µM	10 µM	20 µM	40 µM	60 µM	80 µM	100 µM
**OD well 1**	0.40	0.42	0.39	0.29	0.34	0.17	0.14	0.12	0.14
**OD well 2**	0.45	0.46	0.41	0.34	0.36	0.19	0.17	0.14	0.15
**OD well 3**	0.46	0.46	0.44	0.35	0.35	0.21	0.15	0.15	0.11
**MEAN**	0.44	0.45	0.41	0.33	0.35	0.19	0.15	0.14	0.13
**SD**	0.03	0.02	0.02	0.03	0.01	0.02	0.02	0.01	0.02
**REL [%]**	**100.0**	**102.4**	**93.6**	**75.2**	**80.3**	**42.8**	**35.0**	**32.0**	**30.2**
**CV [%]**	7.60	5.05	6.05	9.56	2.69	10.56	10.68	10.23	14.27

* 0.1 % DMSO

120 h incubation time

	ctrl.*	1 µM	5 µM	10 µM	20 µM	40 µM	60 µM	80 µM	100 µM
**OD well 1**	1.12	1.22	1.12	0.64	0.48	0.22	0.18	0.14	0.11
**OD well 2**	1.14	1.20	1.15	0.54	0.46	0.26	0.16	0.18	0.13
**OD well 3**	1.22	1.23	1.10	0.66	0.52	0.28	0.16	0.13	0.12
**MEAN**	1.16	1.22	1.13	0.61	0.49	0.26	0.17	0.15	0.12
**SD**	0.05	0.01	0.03	0.06	0.03	0.03	0.01	0.02	0.01
**REL [%]**	**100.0**	**105.2**	**97.2**	**52.8**	**42.3**	**22.2**	**14.3**	**12.9**	**10.2**
**CV [%]**	4.46	1.01	2.33	10.60	6.21	11.83	6.15	16.44	6.61

* 0.1 % DMSO

Caco-2 cells

Resveratrol

Experiment 1

48 h incubation time

	ctrl.*	1 µM	5 µM	10 µM	20 µM	40 µM	60 µM	80 µM	100 µM
**OD well 1**	0.19	0.19	0.19	0.19	0.17	0.16	0.14	0.13	0.13
**OD well 2**	0.17	0.18	0.20	0.20	0.18	0.15	0.15	0.13	0.11
**OD well 3**	0.20	0.19	0.18	0.20	0.17	0.16	0.16	0.13	0.12
**MEAN**	0.19	0.19	0.19	0.20	0.17	0.16	0.15	0.13	0.12
**SD**	0.01	0.00	0.01	0.01	0.01	0.01	0.01	0.00	0.01
**REL [%]**	**100.0**	**100.6**	**101.8**	**105.6**	**92.5**	**83.7**	**79.8**	**70.8**	**63.6**
**CV [%]**	7.18	2.12	3.82	3.66	5.84	3.35	5.70	2.63	6.34

* 0.1 % DMSO

72 h incubation time

	ctrl.*	1 µM	5 µM	10 µM	20 µM	40 µM	60 µM	80 µM	100 µM
**OD well 1**	0.45	0.43	0.39	0.35	0.29	0.23	0.21	0.19	0.17
**OD well 2**	0.45	0.39	0.35	0.36	0.28	0.24	0.21	0.17	0.15
**OD well 3**	0.43	0.42	0.34	0.37	0.28	0.24	0.20	0.17	0.16
**MEAN**	0.44	0.41	0.36	0.36	0.28	0.24	0.21	0.18	0.16
**SD**	0.01	0.02	0.03	0.01	0.00	0.00	0.00	0.01	0.01
**REL [%]**	**100.0**	**92.6**	**81.2**	**81.2**	**63.9**	**53.1**	**46.6**	**39.7**	**36.0**
**CV [%]**	2.52	4.63	7.27	3.95	1.22	1.84	2.06	6.66	4.64

* 0.1 % DMSO

120 h incubation time

	ctrl.*	1 µM	5 µM	10 µM	20 µM	40 µM	60 µM	80 µM	100 µM
**OD well 1**	1.22	1.18	0.95	0.78	0.46	0.26	0.23	0.19	0.16
**OD well 2**	1.18	1.23	1.03	0.83	0.50	0.26	0.23	0.16	0.15
**OD well 3**	1.20	1.12	1.01	0.87	0.47	0.25	0.21	0.16	0.14
**MEAN**	1.20	1.17	1.00	0.82	0.48	0.25	0.22	0.17	0.15
**SD**	0.02	0.06	0.04	0.05	0.02	0.00	0.01	0.02	0.01
**REL [%]**	**100.0**	**97.8**	**83.3**	**68.7**	**39.8**	**21.2**	**18.5**	**14.1**	**12.4**
**CV [%]**	1.68	4.85	4.18	5.73	4.72	1.82	6.43	12.30	6.91

* 0.1 % DMSO

Experiment 2

48 h incubation time

	ctrl.*	1 µM	5 µM	10 µM	20 µM	40 µM	60 µM	80 µM	100 µM
**OD well 1**	0.20	0.21	0.19	0.19	0.17	0.17	0.14	0.13	0.13
**OD well 2**	0.20	0.20	0.20	0.20	0.19	0.17	0.14	0.13	0.13
**OD well 3**	0.19	0.20	0.20	0.18	0.18	0.15	0.13	0.13	0.14
**MEAN**	0.20	0.20	0.19	0.19	0.18	0.16	0.14	0.13	0.13
**SD**	0.00	0.00	0.00	0.01	0.01	0.01	0.00	0.00	0.01
**REL [%]**	**100.0**	**103.0**	**99.5**	**96.1**	**92.0**	**82.6**	**69.6**	**67.5**	**67.4**
**CV [%]**	2.01	1.81	1.61	4.60	4.38	4.56	1.12	0.40	3.97

* 0.1 % DMSO

72 h incubation time

	ctrl.*	1 µM	5 µM	10 µM	20 µM	40 µM	60 µM	80 µM	100 µM
**OD well 1**	0.39	0.41	0.38	0.37	0.30	0.21	0.19	0.17	0.17
**OD well 2**	0.40	0.42	0.38	0.36	0.31	0.23	0.19	0.16	0.15
**OD well 3**	0.38	0.42	0.38	0.36	0.31	0.20	0.18	0.16	0.15
**MEAN**	0.39	0.42	0.38	0.36	0.30	0.21	0.19	0.16	0.15
**SD**	0.01	0.01	0.00	0.01	0.00	0.01	0.01	0.00	0.01
**REL [%]**	**100.0**	**106.8**	**95.9**	**92.4**	**77.7**	**54.3**	**48.3**	**41.3**	**39.5**
**CV [%]**	2.10	1.32	0.29	2.24	0.79	5.70	4.05	3.01	5.93

* 0.1 % DMSO

120 h incubation time

	ctrl.*	1 µM	5 µM	10 µM	20 µM	40 µM	60 µM	80 µM	100 µM
**OD well 1**	1.01	1.00	0.82	0.63	0.45	0.25	0.20	0.15	0.13
**OD well 2**	1.04	1.02	0.83	0.61	0.44	0.24	0.18	0.15	0.14
**OD well 3**	1.14	1.02	0.86	0.60	0.44	0.21	0.18	0.15	0.12
**MEAN**	1.06	1.01	0.84	0.61	0.44	0.23	0.19	0.15	0.13
**SD**	0.07	0.01	0.02	0.02	0.01	0.02	0.01	0.00	0.01
**REL [%]**	**100.0**	**95.5**	**78.7**	**57.7**	**41.7**	**21.9**	**17.5**	**14.0**	**12.1**
**CV [%]**	6.63	0.97	2.70	2.94	1.37	7.80	4.59	2.23	5.60

* 0.1 % DMSO

Experiment 3

48 h incubation time

	ctrl.*	1 µM	5 µM	10 µM	20 µM	40 µM	60 µM	80 µM	100 µM
**OD well 1**	0.19	0.20	0.19	0.19	0.21	0.17	0.17	0.16	0.14
**OD well 2**	0.20	0.20	0.20	0.20	0.19	0.16	0.17	0.15	0.14
**OD well 3**	0.21	0.20	0.19	0.20	0.20	0.17	0.16	0.15	0.15
**MEAN**	0.20	0.20	0.19	0.20	0.20	0.17	0.16	0.15	0.14
**SD**	0.01	0.00	0.00	0.01	0.01	0.01	0.01	0.01	0.01
**REL [%]**	**100.0**	**101.1**	**95.5**	**99.0**	**98.3**	**82.6**	**81.6**	**76.2**	**70.5**
**CV [%]**	4.09	0.88	2.30	3.16	5.34	5.81	4.50	4.07	5.00

* 0.1 % DMSO

72 h incubation time

	ctrl.*	1 µM	5 µM	10 µM	20 µM	40 µM	60 µM	80 µM	100 µM
**OD well 1**	0.42	0.46	0.35	0.35	0.32	0.21	0.20	0.17	0.16
**OD well 2**	0.38	0.46	0.39	0.32	0.30	0.23	0.20	0.19	0.15
**OD well 3**	0.44	0.45	0.39	0.38	0.31	0.21	0.21	0.17	0.16
**MEAN**	0.41	0.46	0.38	0.35	0.31	0.22	0.20	0.18	0.16
**SD**	0.03	0.00	0.03	0.03	0.01	0.01	0.01	0.01	0.01
**REL [%]**	**100.0**	**110.6**	**90.9**	**85.1**	**75.3**	**52.6**	**48.5**	**43.2**	**37.8**
**CV [%]**	6.35	1.06	6.81	8.87	3.82	4.02	2.86	7.43	3.77

* 0.1 % DMSO

120 h incubation time

	ctrl.*	1 µM	5 µM	10 µM	20 µM	40 µM	60 µM	80 µM	100 µM
**OD well 1**	1.11	1.10	0.93	0.83	0.49	0.23	0.21	0.19	0.16
**OD well 2**	1.13	1.07	0.90	0.84	0.52	0.23	0.22	0.19	0.14
**OD well 3**	1.08	1.10	0.92	0.83	0.48	0.22	0.19	0.18	0.14
**MEAN**	1.11	1.09	0.92	0.83	0.50	0.23	0.21	0.19	0.15
**SD**	0.02	0.01	0.02	0.00	0.02	0.00	0.02	0.00	0.01
**REL [%]**	**100.0**	**98.5**	**83.2**	**75.4**	**45.1**	**20.7**	**18.6**	**16.8**	**13.4**
**CV [%]**	2.07	1.35	1.67	0.60	4.37	1.74	7.46	2.10	6.22

* 0.1 % DMSO

Experiment 4

48 h incubation time

	ctrl.*	1 µM	5 µM	10 µM	20 µM	40 µM	60 µM	80 µM	100 µM
**OD well 1**	0.20	0.20	0.20	0.19	0.19	0.17	0.16	0.14	0.14
**OD well 2**	0.21	0.20	0.20	0.20	0.18	0.17	0.15	0.14	0.13
**OD well 3**	0.21	0.20	0.21	0.18	0.17	0.16	0.16	0.14	0.14
**MEAN**	0.21	0.20	0.21	0.19	0.18	0.17	0.16	0.14	0.14
**SD**	0.01	0.00	0.01	0.01	0.01	0.01	0.01	0.00	0.01
**REL [%]**	**100.0**	**95.2**	**99.4**	**92.7**	**88.5**	**80.9**	**77.4**	**66.1**	**68.1**
**CV [%]**	2.49	0.21	2.83	5.47	5.46	3.84	3.18	0.96	4.79

* 0.1 % DMSO

72 h incubation time

	ctrl.*	1 µM	5 µM	10 µM	20 µM	40 µM	60 µM	80 µM	100 µM
**OD well 1**	0.45	0.45	0.41	0.33	0.31	0.23	0.23	0.18	0.16
**OD well 2**	0.40	0.41	0.38	0.36	0.29	0.21	0.20	0.16	0.14
**OD well 3**	0.44	0.45	0.40	0.36	0.31	0.23	0.20	0.17	0.14
**MEAN**	0.43	0.44	0.39	0.35	0.31	0.22	0.21	0.17	0.15
**SD**	0.03	0.03	0.01	0.02	0.02	0.01	0.02	0.01	0.01
**REL [%]**	**100.0**	**101.6**	**91.9**	**81.8**	**71.4**	**51.7**	**48.2**	**39.4**	**34.9**
**CV [%]**	6.58	6.03	3.48	5.25	5.07	6.03	8.11	5.12	8.26

* 0.1 % DMSO

120 h incubation time

	ctrl.*	1 µM	5 µM	10 µM	20 µM	40 µM	60 µM	80 µM	100 µM
**OD well 1**	1.40	1.42	1.13	0.91	0.53	0.29	0.24	0.19	0.16
**OD well 2**	1.25	1.32	1.05	0.86	0.55	0.30	0.25	0.19	0.14
**OD well 3**	1.50	1.47	1.17	0.85	0.55	0.27	0.24	0.17	0.14
**MEAN**	1.38	1.40	1.12	0.87	0.54	0.29	0.25	0.18	0.15
**SD**	0.12	0.08	0.06	0.03	0.02	0.02	0.00	0.01	0.01
**REL [%]**	**100.0**	**101.4**	**80.9**	**63.2**	**39.3**	**20.8**	**17.8**	**13.2**	**10.5**
**CV [%]**	9.03	5.45	5.60	3.22	2.78	6.13	1.89	7.76	6.69

* 0.1 % DMSO

Experiment 5

48 h incubation time

	ctrl.*	1 µM	5 µM	10 µM	20 µM	40 µM	60 µM	80 µM	100 µM
**OD well 1**	0.21	0.22	0.22	0.21	0.20	0.19	0.17	0.13	0.16
**OD well 2**	0.23	0.22	0.20	0.21	0.19	0.19	0.17	0.16	0.15
**OD well 3**	0.21	0.23	0.24	0.21	0.20	0.18	0.17	0.18	0.15
**MEAN**	0.22	0.22	0.22	0.21	0.20	0.19	0.17	0.16	0.15
**SD**	0.01	0.01	0.02	0.00	0.01	0.01	0.00	0.03	0.00
**REL [%]**	**100.0**	**101.5**	**101.0**	**96.0**	**90.7**	**84.9**	**77.1**	**72.5**	**70.9**
**CV [%]**	4.28	2.75	8.45	1.27	3.00	3.55	1.52	16.50	1.10

* 0.1 % DMSO

72 h incubation time

	ctrl.*	1 µM	5 µM	10 µM	20 µM	40 µM	60 µM	80 µM	100 µM
**OD well 1**	0.47	0.46	0.44	0.41	0.34	0.24	0.21	0.17	0.19
**OD well 2**	0.47	0.49	0.43	0.36	0.33	0.24	0.20	0.17	0.15
**OD well 3**	0.42	0.46	0.40	0.37	0.32	0.22	0.19	0.17	0.15
**MEAN**	0.45	0.47	0.42	0.38	0.33	0.23	0.20	0.17	0.16
**SD**	0.03	0.02	0.02	0.03	0.01	0.01	0.01	0.00	0.02
**REL [%]**	**100.0**	**102.8**	**93.1**	**84.4**	**72.8**	**51.3**	**43.9**	**37.9**	**35.7**
**CV [%]**	6.86	3.60	5.02	7.13	2.86	5.00	4.56	1.45	14.24

* 0.1 % DMSO

120 h incubation time

	ctrl.*	1 µM	5 µM	10 µM	20 µM	40 µM	60 µM	80 µM	100 µM
**OD well 1**	1.34	1.35	0.94	0.86	0.57	0.28	0.21	0.18	0.15
**OD well 2**	1.35	1.32	1.13	0.83	0.59	0.28	0.21	0.18	0.16
**OD well 3**	1.24	1.16	0.95	0.82	0.59	0.29	0.20	0.18	0.15
**MEAN**	1.31	1.28	1.01	0.84	0.58	0.28	0.20	0.18	0.15
**SD**	0.06	0.10	0.11	0.02	0.01	0.00	0.01	0.00	0.00
**REL [%]**	**100.0**	**97.5**	**77.2**	**63.8**	**44.6**	**21.7**	**15.6**	**13.7**	**11.8**
**CV [%]**	4.83	7.94	10.62	2.71	2.02	1.55	2.75	1.05	0.91

* 0.1 % DMSO

IRA 5

Experiment 1

48 h incubation time

	ctrl.*	1 µM	5 µM	10 µM	20 µM	40 µM	60 µM	80 µM	100 µM
**OD well 1**	0.21	0.22	0.19	0.21	0.18	0.18	0.16	0.16	0.13
**OD well 2**	0.23	0.21	0.22	0.21	0.19	0.19	0.17	0.15	0.13
**OD well 3**	0.21	0.21	0.22	0.20	0.19	0.18	0.15	0.16	0.15
**MEAN**	0.22	0.21	0.21	0.21	0.19	0.18	0.16	0.16	0.14
**SD**	0.01	0.00	0.02	0.00	0.01	0.01	0.01	0.01	0.01
**REL [%]**	**100.0**	**98.3**	**95.6**	**94.9**	**86.4**	**82.3**	**73.2**	**71.8**	**63.1**
**CV [%]**	5.75	0.72	8.16	1.21	2.93	3.79	4.19	5.45	4.91

* 0.1 % DMSO

72 h incubation time

	ctrl.*	1 µM	5 µM	10 µM	20 µM	40 µM	60 µM	80 µM	100 µM
**OD well 1**	0.41	0.38	0.29	0.30	0.23	0.19	0.20	0.17	0.16
**OD well 2**	0.38	0.42	0.33	0.33	0.20	0.20	0.18	0.16	0.16
**OD well 3**	0.44	0.41	0.36	0.33	0.24	0.20	0.20	0.20	0.16
**MEAN**	0.41	0.40	0.33	0.32	0.22	0.20	0.19	0.18	0.16
**SD**	0.03	0.02	0.03	0.02	0.02	0.01	0.01	0.02	0.00
**REL [%]**	**100.0**	**98.1**	**79.8**	**78.1**	**54.5**	**48.5**	**47.1**	**43.2**	**38.4**
**CV [%]**	6.69	5.37	10.69	4.92	9.52	2.59	5.17	11.39	0.26

* 0.1 % DMSO

120 h incubation time

	ctrl.*	1 µM	5 µM	10 µM	20 µM	40 µM	60 µM	80 µM	100 µM
**OD well 1**	1.14	0.84	1.08	0.68	0.42	0.24	0.19	0.17	0.16
**OD well 2**	1.04	0.86	0.97	0.56	0.27	0.20	0.17	0.17	0.17
**OD well 3**	1.11	0.91	0.91	0.63	0.35	0.21	0.18	0.18	0.15
**MEAN**	1.10	0.87	0.99	0.63	0.35	0.22	0.18	0.17	0.16
**SD**	0.05	0.04	0.08	0.06	0.07	0.02	0.01	0.00	0.01
**REL [%]**	**100.0**	**79.6**	**90.1**	**57.1**	**31.6**	**19.6**	**16.5**	**15.6**	**14.8**
**CV [%]**	4.82	4.12	8.43	9.97	21.59	9.53	3.36	2.56	5.61

* 0.1 % DMSO

Experiment 2

48 h incubation time

	ctrl.*	1 µM	5 µM	10 µM	20 µM	40 µM	60 µM	80 µM	100 µM
**OD well 1**	0.20	0.19	0.17	0.19	0.16	0.15	0.15	0.16	0.13
**OD well 2**	0.18	0.18	0.18	0.17	0.14	0.13	0.15	0.15	0.12
**OD well 3**	0.19	0.18	0.18	0.17	0.17	0.15	0.17	0.15	0.15
**MEAN**	0.19	0.18	0.18	0.18	0.16	0.14	0.15	0.15	0.13
**SD**	0.01	0.01	0.01	0.01	0.01	0.01	0.01	0.00	0.01
**REL [%]**	**100.0**	**96.1**	**94.2**	**93.7**	**83.4**	**75.3**	**81.5**	**81.4**	**70.1**
**CV [%]**	5.11	3.09	3.72	6.11	8.19	9.95	6.50	3.09	11.12

* 0.1 % DMSO

72 h incubation time

	ctrl.*	1 µM	5 µM	10 µM	20 µM	40 µM	60 µM	80 µM	100 µM
**OD well 1**	0.38	0.38	0.33	0.29	0.24	0.19	0.17	0.16	0.14
**OD well 2**	0.36	0.39	0.30	0.29	0.20	0.18	0.17	0.15	0.15
**OD well 3**	0.40	0.43	0.34	0.34	0.25	0.20	0.17	0.18	0.17
**MEAN**	0.38	0.40	0.32	0.31	0.23	0.19	0.17	0.16	0.16
**SD**	0.02	0.03	0.02	0.03	0.03	0.01	0.00	0.02	0.02
**REL [%]**	**100.0**	**104.7**	**85.6**	**80.4**	**60.8**	**49.9**	**44.7**	**43.4**	**40.8**
**CV [%]**	4.12	6.42	6.62	8.67	11.69	5.36	2.21	9.93	10.31

* 0.1 % DMSO

120 h incubation time

	ctrl.*	1 µM	5 µM	10 µM	20 µM	40 µM	60 µM	80 µM	100 µM
**OD well 1**	1.06	0.99	0.80	0.53	0.31	0.17	0.16	0.15	0.14
**OD well 2**	0.89	0.93	0.79	0.58	0.26	0.17	0.14	0.15	0.15
**OD well 3**	0.99	1.11	0.72	0.57	0.30	0.19	0.14	0.13	0.14
**MEAN**	0.98	1.01	0.77	0.56	0.29	0.18	0.15	0.14	0.14
**SD**	0.09	0.09	0.05	0.03	0.03	0.01	0.01	0.01	0.01
**REL [%]**	**100.0**	**103.0**	**78.6**	**57.2**	**29.9**	**18.2**	**15.2**	**14.6**	**14.6**
**CV [%]**	8.79	9.00	5.85	4.62	8.57	5.98	8.89	9.30	4.86

* 0.1 % DMSO

Experiment 3

48 h incubation time

	ctrl.*	1 µM	5 µM	10 µM	20 µM	40 µM	60 µM	80 µM	100 µM
**OD well 1**	0.23	0.21	0.23	0.21	0.21	0.19	0.18	0.16	0.17
**OD well 2**	0.21	0.21	0.21	0.21	0.19	0.17	0.16	0.16	0.15
**OD well 3**	0.23	0.21	0.23	0.21	0.19	0.16	0.17	0.16	0.15
**MEAN**	0.22	0.21	0.22	0.21	0.19	0.17	0.17	0.16	0.16
**SD**	0.01	0.00	0.01	0.00	0.01	0.01	0.01	0.00	0.01
**REL [%]**	**100.0**	**93.5**	**97.9**	**94.3**	**86.1**	**76.9**	**76.3**	**72.1**	**69.9**
**CV [%]**	4.49	1.77	4.96	0.38	5.84	7.24	6.40	0.23	6.40

* 0.1 % DMSO

72 h incubation time

	ctrl.*	1 µM	5 µM	10 µM	20 µM	40 µM	60 µM	80 µM	100 µM
**OD well 1**	0.44	0.42	0.37	0.34	0.26	0.20	0.19	0.19	0.17
**OD well 2**	0.40	0.44	0.38	0.29	0.23	0.20	0.18	0.18	0.17
**OD well 3**	0.43	0.43	0.38	0.29	0.27	0.19	0.20	0.19	0.18
**MEAN**	0.42	0.43	0.38	0.31	0.25	0.20	0.19	0.19	0.17
**SD**	0.02	0.01	0.01	0.02	0.02	0.01	0.01	0.01	0.01
**REL [%]**	**100.0**	**101.7**	**89.2**	**72.7**	**59.7**	**46.7**	**44.9**	**44.2**	**40.9**
**CV [%]**	4.16	2.14	1.71	8.06	9.29	2.56	5.35	3.00	4.30

* 0.1 % DMSO

120 h incubation time

	ctrl.*	1 µM	5 µM	10 µM	20 µM	40 µM	60 µM	80 µM	100 µM
**OD well 1**	0.94	0.91	0.70	0.51	0.38	0.18	0.17	0.16	0.17
**OD well 2**	0.85	0.84	0.69	0.44	0.32	0.17	0.16	0.18	0.18
**OD well 3**	0.91	0.83	0.80	0.50	0.34	0.20	0.18	0.20	0.17
**MEAN**	0.90	0.86	0.73	0.48	0.35	0.19	0.17	0.18	0.17
**SD**	0.05	0.04	0.06	0.04	0.03	0.02	0.01	0.02	0.01
**REL [%]**	**100.0**	**95.9**	**81.2**	**53.7**	**38.5**	**20.8**	**18.9**	**20.1**	**19.0**
**CV [%]**	5.09	4.91	8.90	8.21	8.11	8.36	7.06	10.92	3.21

* 0.1 % DMSO

Experiment 4

48 h incubation time

	ctrl.*	1 µM	5 µM	10 µM	20 µM	40 µM	60 µM	80 µM	100 µM
**OD well 1**	0.21	0.21	0.21	0.17	0.16	0.16	0.16	0.15	0.16
**OD well 2**	0.19	0.19	0.20	0.19	0.18	0.17	0.17	0.14	0.14
**OD well 3**	0.22	0.23	0.21	0.20	0.18	0.15	0.14	0.15	0.14
**MEAN**	0.21	0.21	0.21	0.19	0.17	0.16	0.16	0.15	0.15
**SD**	0.02	0.02	0.00	0.02	0.01	0.01	0.01	0.01	0.01
**REL [%]**	**100.0**	**101.3**	**100.7**	**90.2**	**84.8**	**77.2**	**76.6**	**70.9**	**70.8**
**CV [%]**	8.60	8.26	2.18	9.55	7.22	7.60	8.44	6.57	5.94

* 0.1 % DMSO

72 h incubation time

	ctrl.*	1 µM	5 µM	10 µM	20 µM	40 µM	60 µM	80 µM	100 µM
**OD well 1**	0.44	0.45	0.41	0.33	0.29	0.22	0.19	0.19	0.17
**OD well 2**	0.46	0.40	0.40	0.32	0.27	0.20	0.18	0.18	0.19
**OD well 3**	0.40	0.44	0.36	0.34	0.26	0.20	0.19	0.17	0.17
**MEAN**	0.43	0.43	0.39	0.33	0.27	0.21	0.19	0.18	0.18
**SD**	0.03	0.03	0.02	0.01	0.01	0.01	0.01	0.01	0.01
**REL [%]**	**100.0**	**99.9**	**90.9**	**76.0**	**62.7**	**48.3**	**43.0**	**41.0**	**40.8**
**CV [%]**	7.62	6.02	6.35	3.82	5.51	4.04	4.76	5.40	5.35

* 0.1 % DMSO

120 h incubation time

	ctrl.*	1 µM	5 µM	10 µM	20 µM	40 µM	60 µM	80 µM	100 µM
**OD well 1**	1.50	1.37	1.24	0.78	0.42	0.24	0.26	0.20	0.20
**OD well 2**	1.24	1.52	1.21	0.61	0.36	0.22	0.15	0.14	0.16
**OD well 3**	1.33	1.41	1.17	0.73	0.36	0.25	0.18	0.19	0.15
**MEAN**	1.36	1.44	1.21	0.71	0.38	0.24	0.20	0.18	0.17
**SD**	0.13	0.08	0.04	0.09	0.03	0.02	0.05	0.03	0.03
**REL [%]**	**100.0**	**105.8**	**88.8**	**52.1**	**28.2**	**17.5**	**14.5**	**13.0**	**12.6**
**CV [%]**	9.86	5.47	3.04	12.04	8.89	6.96	26.72	16.41	15.48

* 0.1 % DMSO

Experiment 5

48 h incubation time

	ctrl.*	1 µM	5 µM	10 µM	20 µM	40 µM	60 µM	80 µM	100 µM
**OD well 1**	0.26	0.27	0.25	0.21	0.21	0.19	0.19	0.19	0.18
**OD well 2**	0.24	0.24	0.25	0.21	0.19	0.20	0.18	0.18	0.19
**OD well 3**	0.24	0.26	0.25	0.23	0.20	0.20	0.19	0.19	0.19
**MEAN**	0.25	0.26	0.25	0.22	0.20	0.20	0.19	0.19	0.19
**SD**	0.01	0.01	0.00	0.01	0.01	0.00	0.00	0.01	0.00
**REL [%]**	**100.0**	**103.4**	**100.8**	**87.4**	**81.6**	**79.5**	**74.9**	**76.2**	**75.5**
**CV [%]**	2.86	4.34	0.53	5.59	4.59	1.35	1.87	4.53	1.70

* 0.1 % DMSO

72 h incubation time

	ctrl.*	1 µM	5 µM	10 µM	20 µM	40 µM	60 µM	80 µM	100 µM
**OD well 1**	0.44	0.44	0.40	0.35	0.27	0.21	0.19	0.18	0.17
**OD well 2**	0.45	0.48	0.39	0.37	0.23	0.20	0.19	0.16	0.16
**OD well 3**	0.45	0.51	0.42	0.38	0.28	0.22	0.20	0.20	0.19
**MEAN**	0.45	0.47	0.40	0.37	0.26	0.21	0.19	0.18	0.17
**SD**	0.01	0.04	0.01	0.01	0.03	0.01	0.01	0.02	0.01
**REL [%]**	**100.0**	**106.6**	**90.5**	**82.1**	**58.5**	**47.1**	**43.1**	**40.9**	**38.7**
**CV [%]**	1.24	7.56	3.20	2.78	10.70	4.22	4.15	11.87	8.69

* 0.1 % DMSO

120 h incubation time

	ctrl.*	1 µM	5 µM	10 µM	20 µM	40 µM	60 µM	80 µM	100 µM
**OD well 1**	1.39	1.27	1.15	0.72	0.41	0.27	0.24	0.20	0.19
**OD well 2**	1.29	1.13	0.97	0.71	0.38	0.25	0.22	0.19	0.20
**OD well 3**	1.17	1.35	0.92	0.83	0.38	0.24	0.22	0.20	0.20
**MEAN**	1.28	1.25	1.01	0.75	0.39	0.25	0.23	0.20	0.20
**SD**	0.11	0.11	0.12	0.07	0.02	0.02	0.01	0.00	0.01
**REL [%]**	**100.0**	**97.5**	**79.0**	**58.7**	**30.5**	**19.8**	**17.6**	**15.3**	**15.3**
**CV [%]**	8.54	8.69	11.94	8.91	5.01	7.00	3.86	1.60	3.79

* 0.1 % DMSO

HCA-7 cells

Resveratrol

Experiment 1

48 h incubation time

	ctrl.*	1 µM	5 µM	10 µM	20 µM	40 µM	60 µM	80 µM	100 µM
**OD well 1**	0.16	0.15	0.17	0.14	0.16	0.15	0.13	0.14	0.14
**OD well 2**	0.16	0.17	0.15	0.13	0.14	0.15	0.15	0.13	0.14
**OD well 3**	0.17	0.15	0.14	0.14	0.15	0.15	0.13	0.15	0.15
**MEAN**	0.16	0.15	0.16	0.14	0.15	0.15	0.14	0.14	0.14
**SD**	0.01	0.01	0.01	0.00	0.01	0.00	0.01	0.01	0.01
**REL [%]**	**100.0**	**93.8**	**94.4**	**82.7**	**92.1**	**91.0**	**84.3**	**85.8**	**86.8**
**CV [%]**	4.27	7.36	8.08	2.89	4.43	2.53	6.71	7.12	5.39

* 0.1 % DMSO

72 h incubation time

	ctrl.*	1 µM	5 µM	10 µM	20 µM	40 µM	60 µM	80 µM	100 µM
**OD well 1**	0.17	0.17	0.16	0.15	0.15	0.15	0.13	0.12	0.11
**OD well 2**	0.16	0.16	0.17	0.15	0.14	0.14	0.12	0.12	0.11
**OD well 3**	0.16	0.16	0.18	0.16	0.15	0.13	0.13	0.12	0.11
**MEAN**	0.16	0.16	0.17	0.16	0.15	0.14	0.13	0.12	0.11
**SD**	0.00	0.00	0.01	0.01	0.01	0.01	0.01	0.00	0.00
**REL [%]**	**100.0**	**100.1**	**104.9**	**95.5**	**89.0**	**85.4**	**78.4**	**74.8**	**65.0**
**CV [%]**	1.78	2.72	6.00	4.57	5.20	6.21	4.67	1.82	0.66

* 0.1 % DMSO

120 h incubation time

	ctrl.*	1 µM	5 µM	10 µM	20 µM	40 µM	60 µM	80 µM	100 µM
**OD well 1**	0.47	0.48	0.44	0.35	0.35	0.22	0.16	0.15	0.11
**OD well 2**	0.42	0.46	0.50	0.44	0.32	0.22	0.15	0.14	0.11
**OD well 3**	0.49	0.48	0.47	0.45	0.31	0.19	0.15	0.15	0.11
**MEAN**	0.46	0.47	0.47	0.41	0.33	0.21	0.15	0.14	0.11
**SD**	0.04	0.01	0.03	0.05	0.02	0.02	0.00	0.00	0.00
**REL [%]**	**100.0**	**102.0**	**101.5**	**88.4**	**70.2**	**45.6**	**33.1**	**31.0**	**24.6**
**CV [%]**	7.57	3.13	7.00	13.16	6.30	7.76	2.13	3.31	0.53

* 0.1 % DMSO

Experiment 2

48 h incubation time

	ctrl.*	1 µM	5 µM	10 µM	20 µM	40 µM	60 µM	80 µM	100 µM
**OD well 1**	0.14	0.14	0.15	0.15	0.14	0.13	0.13	0.13	0.13
**OD well 2**	0.14	0.15	0.13	0.15	0.13	0.12	0.13	0.12	0.13
**OD well 3**	0.13	0.14	0.14	0.15	0.13	0.13	0.12	0.11	0.12
**MEAN**	0.14	0.14	0.14	0.15	0.14	0.13	0.13	0.12	0.13
**SD**	0.00	0.00	0.01	0.00	0.00	0.00	0.00	0.01	0.01
**REL [%]**	**100.0**	**105.5**	**103.0**	**108.6**	**98.7**	**92.7**	**94.0**	**86.3**	**92.1**
**CV [%]**	3.57	1.21	8.62	1.24	3.45	3.10	3.73	11.88	6.94

* 0.1 % DMSO

72 h incubation time

	ctrl.*	1 µM	5 µM	10 µM	20 µM	40 µM	60 µM	80 µM	100 µM
**OD well 1**	0.23	0.23	0.24	0.20	0.19	0.14	0.13	0.14	0.12
**OD well 2**	0.22	0.23	0.21	0.20	0.19	0.14	0.16	0.13	0.13
**OD well 3**	0.22	0.24	0.21	0.23	0.18	0.15	0.14	0.12	0.11
**MEAN**	0.22	0.23	0.22	0.21	0.18	0.15	0.14	0.13	0.12
**SD**	0.00	0.00	0.02	0.02	0.00	0.01	0.01	0.01	0.01
**REL [%]**	**100.0**	**104.5**	**99.1**	**94.0**	**83.0**	**65.7**	**64.0**	**59.3**	**54.5**
**CV [%]**	2.10	2.08	6.91	7.34	1.86	4.39	9.32	6.28	5.99

* 0.1 % DMSO

120 h incubation time

	ctrl.*	1 µM	5 µM	10 µM	20 µM	40 µM	60 µM	80 µM	100 µM
**OD well 1**	0.36	0.36	0.39	0.31	0.21	0.16	0.13	0.13	0.11
**OD well 2**	0.40	0.37	0.40	0.34	0.21	0.15	0.15	0.13	0.11
**OD well 3**	0.39	0.37	0.36	0.33	0.22	0.14	0.14	0.13	0.11
**MEAN**	0.39	0.36	0.38	0.33	0.21	0.15	0.14	0.13	0.11
**SD**	0.02	0.01	0.02	0.02	0.00	0.01	0.01	0.00	0.00
**REL [%]**	**100.0**	**94.2**	**99.8**	**85.1**	**54.9**	**39.4**	**36.6**	**33.4**	**28.9**
**CV [%]**	6.41	1.69	5.39	4.69	1.62	5.60	6.03	0.96	2.26

* 0.1 % DMSO

Experiment 3

48 h incubation time

	ctrl.*	1 µM	5 µM	10 µM	20 µM	40 µM	60 µM	80 µM	100 µM
**OD well 1**	0.16	0.17	0.18	0.16	0.15	0.14	0.16	0.14	0.14
**OD well 2**	0.15	0.16	0.16	0.15	0.15	0.14	0.13	0.15	0.13
**OD well 3**	0.16	0.14	0.16	0.16	0.16	0.15	0.15	0.13	0.14
**MEAN**	0.16	0.16	0.17	0.16	0.15	0.14	0.15	0.14	0.14
**SD**	0.01	0.02	0.01	0.00	0.01	0.01	0.01	0.01	0.01
**REL [%]**	**100.0**	**101.2**	**106.5**	**101.3**	**98.0**	**92.5**	**92.8**	**87.9**	**86.5**
**CV [%]**	5.39	9.54	7.63	2.10	3.81	5.00	7.50	8.88	6.70

* 0.1 % DMSO

72 h incubation time

	ctrl.*	1 µM	5 µM	10 µM	20 µM	40 µM	60 µM	80 µM	100 µM
**OD well 1**	0.21	0.19	0.18	0.18	0.17	0.14	0.13	0.14	0.12
**OD well 2**	0.20	0.19	0.21	0.20	0.18	0.16	0.16	0.15	0.14
**OD well 3**	0.22	0.21	0.20	0.20	0.16	0.17	0.14	0.14	0.15
**MEAN**	0.21	0.20	0.20	0.19	0.17	0.16	0.14	0.14	0.13
**SD**	0.01	0.01	0.01	0.01	0.01	0.01	0.02	0.01	0.02
**REL [%]**	**100.0**	**95.6**	**96.0**	**93.4**	**82.3**	**76.7**	**68.3**	**68.8**	**64.9**
**CV [%]**	6.43	4.16	7.45	5.90	3.65	8.69	10.76	4.01	11.55

* 0.1 % DMSO

120 h incubation time

	ctrl.*	1 µM	5 µM	10 µM	20 µM	40 µM	60 µM	80 µM	100 µM
**OD well 1**	0.33	0.33	0.37	0.29	0.21	0.15	0.12	0.12	0.10
**OD well 2**	0.37	0.38	0.34	0.32	0.23	0.15	0.11	0.13	0.11
**OD well 3**	0.35	0.36	0.39	0.35	0.23	0.15	0.12	0.11	0.09
**MEAN**	0.35	0.36	0.37	0.32	0.22	0.15	0.12	0.12	0.10
**SD**	0.02	0.02	0.03	0.03	0.01	0.00	0.01	0.01	0.01
**REL [%]**	**100.0**	**102.6**	**105.4**	**92.4**	**63.9**	**43.2**	**34.5**	**34.8**	**28.8**
**CV [%]**	6.09	6.61	7.07	9.38	4.68	2.49	4.43	8.06	8.66

* 0.1 % DMSO

Experiment 4

48 h incubation time

	ctrl.*	1 µM	5 µM	10 µM	20 µM	40 µM	60 µM	80 µM	100 µM
**OD well 1**	0.16	0.17	0.17	0.16	0.16	0.14	0.15	0.15	0.13
**OD well 2**	0.17	0.16	0.17	0.16	0.17	0.16	0.13	0.16	0.15
**OD well 3**	0.17	0.17	0.18	0.16	0.17	0.15	0.16	0.14	0.16
**MEAN**	0.17	0.16	0.18	0.16	0.17	0.15	0.15	0.15	0.15
**SD**	0.00	0.01	0.00	0.00	0.01	0.01	0.01	0.01	0.01
**REL [%]**	**100.0**	**99.3**	**106.3**	**97.3**	**102.5**	**91.1**	**89.6**	**89.1**	**88.0**
**CV [%]**	1.06	4.53	1.11	0.54	3.45	9.15	8.75	6.25	8.58

* 0.1 % DMSO

72 h incubation time

	ctrl.*	1 µM	5 µM	10 µM	20 µM	40 µM	60 µM	80 µM	100 µM
**OD well 1**	0.18	0.15	0.16	0.15	0.13	0.14	0.12	0.12	0.11
**OD well 2**	0.19	0.18	0.15	0.16	0.14	0.12	0.12	0.12	0.11
**OD well 3**	0.16	0.16	0.16	0.16	0.15	0.13	0.12	0.12	0.12
**MEAN**	0.18	0.16	0.16	0.16	0.14	0.13	0.12	0.12	0.11
**SD**	0.01	0.01	0.00	0.01	0.01	0.01	0.00	0.00	0.01
**REL [%]**	**100.0**	**91.2**	**87.3**	**87.4**	**80.0**	**71.9**	**67.8**	**66.3**	**63.6**
**CV [%]**	7.58	8.14	1.82	3.53	7.01	7.05	2.98	1.86	4.61

* 0.1 % DMSO

120 h incubation time

	ctrl.*	1 µM	5 µM	10 µM	20 µM	40 µM	60 µM	80 µM	100 µM
**OD well 1**	0.38	0.26	0.27	0.27	0.18	0.16	0.16	0.15	0.12
**OD well 2**	0.36	0.27	0.26	0.22	0.17	0.15	0.15	0.14	0.10
**OD well 3**	0.31	0.28	0.27	0.23	0.19	0.16	0.15	0.15	0.13
**MEAN**	0.35	0.27	0.27	0.24	0.18	0.16	0.15	0.15	0.11
**SD**	0.03	0.01	0.00	0.02	0.01	0.00	0.00	0.00	0.01
**REL [%]**	**100.0**	**77.9**	**76.1**	**68.7**	**51.9**	**44.4**	**44.3**	**42.0**	**32.8**
**CV [%]**	9.46	3.38	1.15	10.20	4.37	3.22	2.30	2.89	11.27

* 0.1 % DMSO

Experiment 5

48 h incubation time

	ctrl.*	1 µM	5 µM	10 µM	20 µM	40 µM	60 µM	80 µM	100 µM
**OD well 1**	0.14	0.15	0.15	0.14	0.13	0.13	0.12	0.12	0.11
**OD well 2**	0.16	0.15	0.14	0.14	0.14	0.13	0.13	0.12	0.11
**OD well 3**	0.16	0.14	0.14	0.14	0.14	0.13	0.13	0.12	0.11
**MEAN**	0.15	0.15	0.15	0.14	0.13	0.13	0.12	0.12	0.11
**SD**	0.01	0.01	0.00	0.00	0.01	0.00	0.00	0.00	0.00
**REL [%]**	**100.0**	**97.2**	**95.2**	**92.4**	**87.1**	**85.6**	**81.6**	**77.2**	**72.9**
**CV [%]**	6.47	5.04	0.56	2.06	3.96	1.60	2.84	0.90	2.89

* 0.1 % DMSO

72 h incubation time

	ctrl.*	1 µM	5 µM	10 µM	20 µM	40 µM	60 µM	80 µM	100 µM
**OD well 1**	0.22	0.22	0.23	0.20	0.19	0.15	0.15	0.13	0.13
**OD well 2**	0.22	0.21	0.22	0.20	0.19	0.15	0.15	0.13	0.11
**OD well 3**	0.23	0.23	0.23	0.22	0.19	0.17	0.15	0.14	0.12
**MEAN**	0.22	0.22	0.23	0.21	0.19	0.15	0.15	0.13	0.12
**SD**	0.01	0.01	0.00	0.01	0.00	0.01	0.00	0.01	0.01
**REL [%]**	**100.0**	**97.1**	**102.1**	**92.5**	**84.8**	**68.6**	**66.2**	**58.3**	**54.1**
**CV [%]**	3.74	3.15	2.02	5.24	1.22	7.10	2.33	3.84	6.56

* 0.1 % DMSO

120 h incubation time

	ctrl.*	1 µM	5 µM	10 µM	20 µM	40 µM	60 µM	80 µM	100 µM
**OD well 1**	0.44	0.46	0.41	0.33	0.24	0.16	0.14	0.12	0.12
**OD well 2**	0.45	0.47	0.43	0.33	0.25	0.17	0.14	0.11	0.12
**OD well 3**	0.43	0.45	0.42	0.30	0.24	0.17	0.14	0.13	0.12
**MEAN**	0.44	0.46	0.42	0.32	0.24	0.17	0.14	0.12	0.12
**SD**	0.01	0.01	0.01	0.02	0.00	0.00	0.00	0.01	0.00
**REL [%]**	**100.0**	**105.4**	**96.4**	**73.0**	**55.7**	**38.1**	**31.8**	**27.8**	**26.8**
**CV [%]**	2.47	2.71	1.73	5.25	1.92	2.84	1.53	5.89	0.68

* 0.1 % DMSO

Experiment 6

48 h incubation time

	ctrl.*	1 µM	5 µM	10 µM	20 µM	40 µM	60 µM	80 µM	100 µM
**OD well 1**	0.13	0.14	0.14	0.14	0.13	0.12	0.12	0.12	0.11
**OD well 2**	0.14	0.13	0.13	0.13	0.13	0.12	0.12	0.12	0.12
**OD well 3**	0.14	0.14	0.14	0.14	0.13	0.11	0.11	0.12	0.12
**MEAN**	0.14	0.14	0.14	0.14	0.13	0.12	0.12	0.12	0.12
**SD**	0.00	0.00	0.00	0.00	0.00	0.00	0.01	0.01	0.00
**REL [%]**	**100.0**	**100.7**	**99.1**	**99.5**	**96.6**	**86.4**	**87.1**	**86.1**	**84.7**
**CV [%]**	3.37	3.43	3.41	2.94	1.35	4.16	5.19	4.33	2.24

* 0.1 % DMSO

72 h incubation time

	ctrl.*	1 µM	5 µM	10 µM	20 µM	40 µM	60 µM	80 µM	100 µM
**OD well 1**	0.18	0.20	0.20	0.18	0.17	0.15	0.13	0.12	0.11
**OD well 2**	0.20	0.21	0.20	0.19	0.18	0.14	0.13	0.12	0.11
**OD well 3**	0.19	0.19	0.19	0.19	0.17	0.14	0.13	0.12	0.10
**MEAN**	0.19	0.20	0.20	0.19	0.17	0.15	0.13	0.12	0.11
**SD**	0.01	0.01	0.00	0.01	0.01	0.01	0.00	0.00	0.00
**REL [%]**	**100.0**	**105.1**	**102.6**	**99.1**	**90.5**	**76.0**	**68.2**	**63.0**	**56.6**
**CV [%]**	3.51	4.78	1.69	2.94	4.78	3.56	1.99	2.72	4.60

* 0.1 % DMSO

120 h incubation time

	ctrl.*	1 µM	5 µM	10 µM	20 µM	40 µM	60 µM	80 µM	100 µM
**OD well 1**	0.46	0.52	0.43	0.35	0.24	0.15	0.14	0.11	0.11
**OD well 2**	0.46	0.49	0.40	0.36	0.24	0.14	0.14	0.11	0.10
**OD well 3**	0.43	0.44	0.38	0.34	0.26	0.14	0.13	0.12	0.11
**MEAN**	0.45	0.48	0.40	0.35	0.24	0.14	0.14	0.11	0.10
**SD**	0.02	0.04	0.02	0.01	0.01	0.00	0.00	0.00	0.01
**REL [%]**	**100.0**	**107.1**	**89.6**	**77.5**	**54.2**	**31.7**	**30.0**	**25.4**	**23.2**
**CV [%]**	3.53	7.79	5.13	1.99	4.93	2.05	3.28	0.69	7.76

* 0.1 % DMSO

IRA 5

Experiment 1

48 h incubation time

	ctrl.*	1 µM	5 µM	10 µM	20 µM	40 µM	60 µM	80 µM	100 µM
**OD well 1**	0.16	0.16	0.15	0.13	0.16	0.16	0.17	0.15	0.13
**OD well 2**	0.15	0.13	0.13	0.14	0.15	0.15	0.14	0.15	0.13
**OD well 3**	0.14	0.11	0.14	0.15	0.17	0.17	0.15	0.14	0.14
**MEAN**	0.15	0.13	0.14	0.14	0.16	0.16	0.15	0.15	0.13
**SD**	0.01	0.02	0.01	0.01	0.01	0.01	0.01	0.00	0.00
**REL [%]**	**100.0**	**87.9**	**90.7**	**92.4**	**108.5**	**106.3**	**101.8**	**99.5**	**89.7**
**CV [%]**	5.39	18.32	6.50	5.52	4.78	8.89	9.17	2.99	2.21

* 0.1 % DMSO

72 h incubation time

	ctrl.*	1 µM	5 µM	10 µM	20 µM	40 µM	60 µM	80 µM	100 µM
**OD well 1**	0.16	0.16	0.15	0.16	0.16	0.15	0.15	0.14	0.12
**OD well 2**	0.14	0.14	0.14	0.14	0.16	0.16	0.15	0.14	0.12
**OD well 3**	0.18	0.17	0.16	0.15	0.17	0.15	0.15	0.14	0.13
**MEAN**	0.16	0.16	0.15	0.15	0.16	0.15	0.15	0.14	0.13
**SD**	0.02	0.01	0.01	0.01	0.01	0.01	0.00	0.00	0.00
**REL [%]**	**100.0**	**97.5**	**93.4**	**94.9**	**102.1**	**96.3**	**93.0**	**88.1**	**79.2**
**CV [%]**	13.90	8.58	5.51	6.73	4.48	4.67	1.22	1.01	3.46

* 0.1 % DMSO

120 h incubation time

	ctrl.*	1 µM	5 µM	10 µM	20 µM	40 µM	60 µM	80 µM	100 µM
**OD well 1**	0.44	0.52	0.49	0.47	0.40	0.29	0.19	0.16	0.13
**OD well 2**	0.50	0.51	0.56	0.44	0.41	0.28	0.18	0.17	0.14
**OD well 3**	0.44	0.51	0.53	0.41	0.48	0.26	0.19	0.17	0.14
**MEAN**	0.46	0.51	0.53	0.44	0.43	0.28	0.18	0.17	0.14
**SD**	0.03	0.01	0.04	0.03	0.04	0.02	0.01	0.00	0.01
**REL [%]**	**100.0**	**110.8**	**113.6**	**94.8**	**93.0**	**60.4**	**39.8**	**36.2**	**29.9**
**CV [%]**	7.23	1.08	6.96	7.44	9.16	5.53	2.94	2.23	4.17

* 0.1 % DMSO

Experiment 2

48 h incubation time

	ctrl.*	1 µM	5 µM	10 µM	20 µM	40 µM	60 µM	80 µM	100 µM
**OD well 1**	0.14	0.12	0.15	0.15	0.15	0.12	0.12	0.12	0.12
**OD well 2**	0.14	0.14	0.14	0.14	0.15	0.12	0.13	0.12	0.12
**OD well 3**	0.14	0.14	0.15	0.14	0.13	0.13	0.12	0.13	0.12
**MEAN**	0.14	0.14	0.14	0.14	0.14	0.13	0.13	0.13	0.12
**SD**	0.00	0.01	0.01	0.00	0.01	0.00	0.00	0.00	0.00
**REL [%]**	**100.0**	**96.3**	**103.0**	**100.8**	**101.4**	**89.0**	**89.2**	**88.9**	**84.1**
**CV [%]**	1.22	7.45	4.07	2.75	5.12	3.39	3.55	2.57	1.68

* 0.1 % DMSO

72 h incubation time

	ctrl.*	1 µM	5 µM	10 µM	20 µM	40 µM	60 µM	80 µM	100 µM
**OD well 1**	0.20	0.21	0.22	0.21	0.19	0.17	0.17	0.16	0.16
**OD well 2**	0.21	0.23	0.23	0.22	0.20	0.16	0.15	0.15	0.15
**OD well 3**	0.23	0.24	0.22	0.23	0.22	0.17	0.17	0.17	0.20
**MEAN**	0.21	0.23	0.22	0.22	0.21	0.16	0.16	0.16	0.17
**SD**	0.01	0.02	0.01	0.01	0.01	0.00	0.01	0.01	0.03
**REL [%]**	**100.0**	**105.7**	**103.7**	**103.1**	**95.8**	**76.2**	**76.1**	**74.3**	**79.5**
**CV [%]**	4.87	8.05	3.26	4.05	5.98	2.45	5.87	4.46	17.94

* 0.1 % DMSO

120 h incubation time

	ctrl.*	1 µM	5 µM	10 µM	20 µM	40 µM	60 µM	80 µM	100 µM
**OD well 1**	0.41	0.39	0.40	0.40	0.35	0.22	0.14	0.14	0.13
**OD well 2**	0.39	0.38	0.39	0.36	0.30	0.21	0.16	0.16	0.14
**OD well 3**	0.44	0.42	0.35	0.36	0.35	0.28	0.17	0.18	0.14
**MEAN**	0.42	0.40	0.38	0.37	0.33	0.24	0.16	0.16	0.14
**SD**	0.03	0.02	0.03	0.02	0.02	0.04	0.02	0.02	0.00
**REL [%]**	**100.0**	**95.9**	**91.0**	**89.6**	**79.9**	**56.9**	**37.5**	**38.4**	**33.2**
**CV [%]**	6.31	5.38	7.26	5.92	7.41	15.70	11.61	10.41	3.06

* 0.1 % DMSO

Experiment 3

48 h incubation time

	ctrl.*	1 µM	5 µM	10 µM	20 µM	40 µM	60 µM	80 µM	100 µM
**OD well 1**	0.16	0.17	0.17	0.15	0.14	0.16	0.17	0.13	0.12
**OD well 2**	0.17	0.16	0.17	0.16	0.17	0.16	0.16	0.13	0.13
**OD well 3**	0.17	0.18	0.18	0.17	0.16	0.14	0.14	0.15	0.16
**MEAN**	0.17	0.17	0.17	0.16	0.16	0.15	0.15	0.14	0.13
**SD**	0.01	0.01	0.00	0.01	0.01	0.01	0.02	0.01	0.02
**REL [%]**	**100.0**	**102.3**	**104.5**	**98.0**	**95.0**	**93.7**	**93.9**	**84.8**	**81.6**
**CV [%]**	4.05	5.60	2.81	6.63	7.56	7.54	11.28	7.22	13.83

* 0.1 % DMSO

72 h incubation time

	ctrl.*	1 µM	5 µM	10 µM	20 µM	40 µM	60 µM	80 µM	100 µM
**OD well 1**	0.18	0.22	0.21	0.21	0.19	0.19	0.15	0.13	0.14
**OD well 2**	0.22	0.22	0.22	0.22	0.18	0.16	0.15	0.10	0.13
**OD well 3**	0.19	0.21	0.21	0.21	0.20	0.17	0.14	0.14	0.16
**MEAN**	0.20	0.22	0.21	0.21	0.19	0.17	0.15	0.12	0.14
**SD**	0.02	0.00	0.00	0.00	0.01	0.01	0.00	0.02	0.01
**REL [%]**	**100.0**	**111.2**	**108.7**	**108.8**	**96.8**	**88.0**	**76.0**	**64.0**	**72.5**
**CV [%]**	11.66	1.62	2.19	1.75	6.42	7.38	2.91	13.90	9.72

* 0.1 % DMSO

120 h incubation time

	ctrl.*	1 µM	5 µM	10 µM	20 µM	40 µM	60 µM	80 µM	100 µM
**OD well 1**	0.40	0.41	0.40	0.42	0.31	0.18	0.14	0.14	0.12
**OD well 2**	0.42	0.39	0.41	0.36	0.28	0.17	0.12	0.13	0.11
**OD well 3**	0.38	0.38	0.38	0.40	0.28	0.18	0.13	0.13	0.10
**MEAN**	0.40	0.39	0.40	0.39	0.29	0.18	0.13	0.13	0.11
**SD**	0.02	0.02	0.02	0.03	0.02	0.00	0.01	0.01	0.01
**REL [%]**	**100.0**	**99.2**	**99.9**	**99.0**	**73.3**	**44.8**	**32.7**	**33.6**	**27.7**
**CV [%]**	5.29	4.29	4.01	7.23	6.09	2.65	7.39	4.43	7.98

* 0.1 % DMSO

Experiment 4

48 h incubation time

	ctrl.*	1 µM	5 µM	10 µM	20 µM	40 µM	60 µM	80 µM	100 µM
**OD well 1**	0.18	0.17	0.18	0.16	0.15	0.17	0.17	0.13	0.15
**OD well 2**	0.16	0.17	0.17	0.15	0.17	0.16	0.16	0.14	0.14
**OD well 3**	0.16	0.19	0.18	0.17	0.14	0.15	0.15	0.15	0.15
**MEAN**	0.16	0.17	0.18	0.16	0.16	0.16	0.16	0.14	0.15
**SD**	0.01	0.01	0.01	0.01	0.01	0.01	0.01	0.01	0.01
**REL [%]**	**100.0**	**105.8**	**107.1**	**98.3**	**94.4**	**94.6**	**94.9**	**86.8**	**88.2**
**CV [%]**	5.86	6.32	3.42	5.35	7.22	6.80	6.38	7.14	4.50

* 0.1 % DMSO

72 h incubation time

	ctrl.*	1 µM	5 µM	10 µM	20 µM	40 µM	60 µM	80 µM	100 µM
**OD well 1**	0.17	0.18	0.17	0.17	0.16	0.15	0.13	0.11	0.12
**OD well 2**	0.19	0.17	0.16	0.15	0.14	0.16	0.12	0.12	0.11
**OD well 3**	0.18	0.18	0.18	0.17	0.16	0.15	0.12	0.12	0.13
**MEAN**	0.18	0.18	0.17	0.16	0.15	0.15	0.12	0.12	0.12
**SD**	0.01	0.01	0.01	0.01	0.01	0.01	0.00	0.01	0.01
**REL [%]**	**100.0**	**99.0**	**93.7**	**91.3**	**83.5**	**84.6**	**68.8**	**64.9**	**65.5**
**CV [%]**	5.32	3.78	6.44	5.32	6.68	3.91	3.76	5.63	5.62

* 0.1 % DMSO

120 h incubation time

	ctrl.*	1 µM	5 µM	10 µM	20 µM	40 µM	60 µM	80 µM	100 µM
**OD well 1**	0.32	0.31	0.29	0.28	0.25	0.18	0.15	0.15	0.13
**OD well 2**	0.34	0.30	0.30	0.25	0.22	0.18	0.15	0.16	0.12
**OD well 3**	0.33	0.33	0.29	0.24	0.23	0.17	0.14	0.15	0.14
**MEAN**	0.33	0.31	0.30	0.26	0.23	0.18	0.15	0.15	0.13
**SD**	0.01	0.01	0.01	0.02	0.02	0.01	0.01	0.01	0.01
**REL [%]**	**100.0**	**95.0**	**90.6**	**78.1**	**70.6**	**54.2**	**45.6**	**46.5**	**38.9**
**CV [%]**	3.36	4.07	2.08	6.48	6.78	4.79	3.57	3.63	6.14

* 0.1 % DMSO

Experiment 5

48 h incubation time

	ctrl.*	1 µM	5 µM	10 µM	20 µM	40 µM	60 µM	80 µM	100 µM
**OD well 1**	0.14	0.15	0.15	0.15	0.14	0.14	0.14	0.13	0.12
**OD well 2**	0.15	0.15	0.15	0.14	0.13	0.14	0.14	0.12	0.10
**OD well 3**	0.16	0.17	0.17	0.16	0.15	0.15	0.14	0.13	0.11
**MEAN**	0.15	0.16	0.16	0.15	0.14	0.14	0.14	0.13	0.11
**SD**	0.01	0.01	0.01	0.01	0.01	0.01	0.00	0.00	0.01
**REL [%]**	**100.0**	**105.8**	**106.1**	**101.7**	**95.9**	**97.8**	**94.2**	**85.1**	**75.7**
**CV [%]**	7.09	7.70	6.25	6.47	8.21	4.95	2.64	3.01	5.30

* 0.1 % DMSO

72 h incubation time

	ctrl.*	1 µM	5 µM	10 µM	20 µM	40 µM	60 µM	80 µM	100 µM
**OD well 1**	0.23	0.22	0.20	0.22	0.21	0.21	0.18	0.16	0.13
**OD well 2**	0.22	0.21	0.21	0.21	0.19	0.21	0.19	0.16	0.14
**OD well 3**	0.23	0.21	0.22	0.23	0.21	0.21	0.18	0.18	0.15
**MEAN**	0.22	0.22	0.21	0.22	0.20	0.21	0.18	0.17	0.14
**SD**	0.01	0.01	0.01	0.01	0.01	0.00	0.01	0.01	0.01
**REL [%]**	**100.0**	**96.5**	**94.9**	**98.6**	**91.4**	**93.0**	**81.2**	**74.4**	**62.8**
**CV [%]**	2.95	3.46	5.05	6.36	4.70	1.61	3.08	8.60	4.98

* 0.1 % DMSO

120 h incubation time

	ctrl.*	1 µM	5 µM	10 µM	20 µM	40 µM	60 µM	80 µM	100 µM
**OD well 1**	0.44	0.40	0.41	0.35	0.27	0.27	0.20	0.18	0.14
**OD well 2**	0.39	0.38	0.40	0.37	0.26	0.27	0.20	0.17	0.12
**OD well 3**	0.41	0.37	0.37	0.33	0.27	0.28	0.21	0.18	0.14
**MEAN**	0.41	0.38	0.39	0.35	0.27	0.27	0.20	0.18	0.13
**SD**	0.03	0.02	0.02	0.02	0.01	0.01	0.01	0.00	0.01
**REL [%]**	**100.0**	**92.3**	**94.7**	**84.6**	**64.8**	**65.9**	**48.4**	**42.7**	**31.8**
**CV [%]**	6.06	4.09	4.88	5.19	2.59	3.63	2.64	2.68	5.50

* 0.1 % DMSO

Experiment 6

48 h incubation time

	ctrl.*	1 µM	5 µM	10 µM	20 µM	40 µM	60 µM	80 µM	100 µM
**OD well 1**	0.15	0.12	0.13	0.13	0.13	0.13	0.13	0.13	0.11
**OD well 2**	0.14	0.12	0.12	0.13	0.12	0.14	0.12	0.12	0.12
**OD well 3**	0.14	0.13	0.14	0.13	0.13	0.13	0.12	0.13	0.12
**MEAN**	0.14	0.12	0.13	0.13	0.13	0.13	0.12	0.12	0.12
**SD**	0.01	0.00	0.01	0.00	0.00	0.00	0.00	0.01	0.01
**REL [%]**	**100.0**	**89.3**	**95.0**	**93.7**	**91.9**	**93.0**	**88.4**	**87.9**	**85.5**
**CV [%]**	3.84	1.35	7.17	2.53	3.66	3.66	3.17	5.21	4.36

* 0.1 % DMSO

72 h incubation time

	ctrl.*	1 µM	5 µM	10 µM	20 µM	40 µM	60 µM	80 µM	100 µM
**OD well 1**	0.18	0.19	0.18	0.18	0.18	0.16	0.17	0.16	0.13
**OD well 2**	0.18	0.17	0.16	0.18	0.17	0.15	0.15	0.14	0.13
**OD well 3**	0.18	0.18	0.18	0.18	0.19	0.16	0.17	0.15	0.14
**MEAN**	0.18	0.18	0.18	0.18	0.18	0.16	0.16	0.15	0.13
**SD**	0.00	0.01	0.01	0.00	0.01	0.01	0.01	0.01	0.00
**REL [%]**	**100.0**	**100.0**	**97.3**	**100.5**	**100.2**	**86.5**	**89.7**	**82.6**	**73.9**
**CV [%]**	1.72	8.21	5.56	1.29	4.64	5.40	5.17	5.81	3.47

* 0.1 % DMSO

120 h incubation time

	ctrl.*	1 µM	5 µM	10 µM	20 µM	40 µM	60 µM	80 µM	100 µM
**OD well 1**	0.46	0.46	0.42	0.41	0.37	0.27	0.19	0.16	0.13
**OD well 2**	0.43	0.45	0.46	0.41	0.36	0.24	0.18	0.16	0.13
**OD well 3**	0.44	0.49	0.46	0.42	0.39	0.26	0.18	0.18	0.13
**MEAN**	0.44	0.47	0.45	0.41	0.37	0.26	0.18	0.17	0.13
**SD**	0.01	0.02	0.02	0.01	0.02	0.02	0.01	0.01	0.00
**REL [%]**	**100.0**	**105.9**	**100.9**	**93.8**	**84.2**	**58.4**	**41.7**	**37.5**	**29.8**
**CV [%]**	3.20	3.96	5.55	1.82	5.07	6.84	3.22	5.33	2.68

* 0.1 % DMSO

HCT-116$^{p53-/-}$ cells

Resveratrol

Experiment 1

48 h incubation time

	ctrl.*	1 µM	5 µM	10 µM	20 µM	40 µM	60 µM	80 µM	100 µM
**OD well 1**	0.23	0.23	0.23	0.21	0.17	0.17	0.16	0.14	0.13
**OD well 2**	0.23	0.23	0.22	0.23	0.19	0.17	0.16	0.14	0.13
**OD well 3**	0.24	0.23	0.22	0.21	0.17	0.17	0.16	0.15	0.13
**MEAN**	0.23	0.23	0.22	0.22	0.18	0.17	0.16	0.14	0.13
**SD**	0.00	0.00	0.01	0.01	0.01	0.00	0.00	0.00	0.00
**REL [%]**	**100.0**	**99.5**	**95.2**	**94.1**	**76.1**	**73.0**	**69.9**	**60.7**	**56.0**
**CV [%]**	1.70	1.70	4.04	4.15	6.13	2.18	1.60	3.33	3.10

* 0.1 % DMSO

72 h incubation time

	ctrl.*	1 µM	5 µM	10 µM	20 µM	40 µM	60 µM	80 µM	100 µM
**OD well 1**	0.35	0.35	0.36	0.35	0.29	0.23	0.18	0.17	0.18
**OD well 2**	0.36	0.39	0.37	0.34	0.27	0.26	0.21	0.20	0.18
**OD well 3**	0.33	0.37	0.37	0.33	0.30	0.25	0.22	0.17	0.17
**MEAN**	0.34	0.37	0.36	0.34	0.29	0.25	0.21	0.18	0.17
**SD**	0.01	0.02	0.01	0.01	0.02	0.01	0.02	0.02	0.01
**REL [%]**	**100.0**	**108.0**	**106.1**	**99.0**	**83.8**	**71.4**	**59.8**	**52.9**	**50.5**
**CV [%]**	4.10	5.85	1.59	2.91	5.50	5.27	10.25	8.90	3.06

* 0.1 % DMSO

120 h incubation time

	ctrl.*	1 µM	5 µM	10 µM	20 µM	40 µM	60 µM	80 µM	100 µM
**OD well 1**	1.56	1.58	1.68	1.58	1.16	0.44	0.28	0.21	0.17
**OD well 2**	1.59	1.47	1.56	1.63	1.08	0.39	0.26	0.18	0.17
**OD well 3**	1.65	1.52	1.66	1.57	1.05	0.43	0.28	0.20	0.15
**MEAN**	1.60	1.52	1.63	1.60	1.10	0.42	0.27	0.20	0.17
**SD**	0.05	0.06	0.06	0.03	0.06	0.03	0.01	0.02	0.01
**REL [%]**	**100.0**	**95.1**	**102.2**	**99.8**	**68.6**	**26.3**	**17.1**	**12.3**	**10.3**
**CV [%]**	2.88	3.75	3.96	1.78	5.58	6.08	3.53	8.41	7.37

* 0.1 % DMSO

Experiment 2

48 h incubation time

	ctrl.*	1 µM	5 µM	10 µM	20 µM	40 µM	60 µM	80 µM	100 µM
**OD well 1**	0.22	0.22	0.22	0.21	0.19	0.18	0.17	0.14	0.14
**OD well 2**	0.23	0.22	0.22	0.22	0.19	0.19	0.15	0.13	0.13
**OD well 3**	0.22	0.24	0.23	0.21	0.21	0.17	0.16	0.14	0.13
**MEAN**	0.23	0.22	0.22	0.21	0.20	0.18	0.16	0.14	0.13
**SD**	0.01	0.01	0.01	0.00	0.01	0.01	0.01	0.01	0.00
**REL [%]**	**100.0**	**99.4**	**98.6**	**94.2**	**86.9**	**79.2**	**70.8**	**61.4**	**58.4**
**CV [%]**	2.24	4.98	4.32	1.64	4.64	3.62	5.02	3.84	3.37

* 0.1 % DMSO

72 h incubation time

	ctrl.*	1 µM	5 µM	10 µM	20 µM	40 µM	60 µM	80 µM	100 µM
**OD well 1**	0.41	0.41	0.43	0.39	0.36	0.24	0.18	0.16	0.16
**OD well 2**	0.43	0.38	0.46	0.38	0.31	0.26	0.19	0.17	0.15
**OD well 3**	0.43	0.44	0.46	0.43	0.36	0.29	0.22	0.18	0.15
**MEAN**	0.42	0.41	0.45	0.40	0.34	0.26	0.20	0.17	0.16
**SD**	0.01	0.03	0.02	0.03	0.02	0.02	0.02	0.01	0.01
**REL [%]**	**100.0**	**96.9**	**106.4**	**94.6**	**80.7**	**62.1**	**46.6**	**39.9**	**36.9**
**CV [%]**	2.53	7.01	3.64	6.93	7.28	8.42	10.91	5.43	4.38

* 0.1 % DMSO

120 h incubation time

	ctrl.*	1 µM	5 µM	10 µM	20 µM	40 µM	60 µM	80 µM	100 µM
**OD well 1**	1.76	1.79	1.67	1.31	1.20	0.51	0.32	0.23	0.26
**OD well 2**	1.76	1.91	1.71	1.57	1.21	0.57	0.32	0.23	0.25
**OD well 3**	1.72	1.74	1.70	1.59	1.14	0.53	0.31	0.27	0.22
**MEAN**	1.75	1.81	1.69	1.49	1.18	0.54	0.32	0.24	0.24
**SD**	0.02	0.08	0.02	0.16	0.04	0.03	0.01	0.02	0.02
**REL [%]**	**100.0**	**103.7**	**96.8**	**85.0**	**67.6**	**30.7**	**18.1**	**13.9**	**13.8**
**CV [%]**	1.23	4.68	1.07	10.59	3.44	6.25	2.71	10.21	7.57

* 0.1 % DMSO

Experiment 3

48 h incubation time

	ctrl.*	1 µM	5 µM	10 µM	20 µM	40 µM	60 µM	80 µM	100 µM
**OD well 1**	0.25	0.24	0.24	0.21	0.20	0.18	0.18	0.13	0.13
**OD well 2**	0.25	0.26	0.27	0.26	0.19	0.20	0.18	0.15	0.15
**OD well 3**	0.23	0.26	0.24	0.23	0.19	0.18	0.15	0.15	0.14
**MEAN**	0.25	0.25	0.25	0.23	0.20	0.19	0.17	0.14	0.14
**SD**	0.01	0.01	0.02	0.02	0.01	0.01	0.01	0.01	0.01
**REL [%]**	**100.0**	**103.5**	**101.1**	**95.0**	**79.5**	**75.7**	**69.2**	**57.8**	**57.3**
**CV [%]**	4.01	4.36	7.52	9.44	3.71	5.75	7.86	5.93	5.79

* 0.1 % DMSO

72 h incubation time

	ctrl.*	1 µM	5 µM	10 µM	20 µM	40 µM	60 µM	80 µM	100 µM
**OD well 1**	0.35	0.34	0.31	0.33	0.27	0.23	0.16	0.15	0.16
**OD well 2**	0.33	0.34	0.33	0.30	0.26	0.19	0.16	0.14	0.16
**OD well 3**	0.34	0.35	0.30	0.29	0.27	0.23	0.17	0.15	0.15
**MEAN**	0.34	0.34	0.31	0.30	0.27	0.22	0.16	0.15	0.15
**SD**	0.01	0.00	0.01	0.02	0.01	0.02	0.00	0.01	0.01
**REL [%]**	**100.0**	**102.3**	**93.5**	**90.3**	**78.9**	**64.8**	**48.3**	**43.4**	**45.9**
**CV [%]**	2.64	1.30	3.40	6.34	3.27	9.37	2.18	4.01	3.91

* 0.1 % DMSO

120 h incubation time

	ctrl.*	1 µM	5 µM	10 µM	20 µM	40 µM	60 µM	80 µM	100 µM
**OD well 1**	1.85	1.68	1.87	1.54	1.34	0.43	0.27	0.20	0.18
**OD well 2**	1.76	1.79	1.93	1.67	1.04	0.45	0.26	0.19	0.20
**OD well 3**	1.56	1.79	1.71	1.55	1.03	0.41	0.28	0.19	0.17
**MEAN**	1.72	1.75	1.84	1.58	1.13	0.43	0.27	0.19	0.18
**SD**	0.15	0.06	0.11	0.07	0.17	0.02	0.01	0.00	0.01
**REL [%]**	**100.0**	**101.6**	**106.4**	**91.8**	**65.8**	**24.9**	**15.6**	**11.1**	**10.7**
**CV [%]**	8.67	3.66	6.24	4.57	15.39	5.39	4.36	2.46	6.12

* 0.1 % DMSO

Experiment 4

48 h incubation time

	ctrl.*	1 µM	5 µM	10 µM	20 µM	40 µM	60 µM	80 µM	100 µM
**OD well 1**	0.23	0.24	0.24	0.22	0.19	0.19	0.20	0.17	0.15
**OD well 2**	0.24	0.24	0.25	0.22	0.19	0.18	0.17	0.16	0.14
**OD well 3**	0.26	0.23	0.27	0.23	0.19	0.20	0.16	0.15	0.16
**MEAN**	0.24	0.24	0.25	0.22	0.19	0.19	0.18	0.16	0.15
**SD**	0.02	0.01	0.01	0.01	0.00	0.01	0.02	0.01	0.01
**REL [%]**	**100.0**	**98.4**	**104.7**	**91.2**	**78.6**	**78.5**	**72.8**	**66.5**	**62.1**
**CV [%]**	7.74	2.37	5.71	2.72	1.67	2.86	12.43	4.05	5.51

* 0.1 % DMSO

72 h incubation time

	ctrl.*	1 µM	5 µM	10 µM	20 µM	40 µM	60 µM	80 µM	100 µM
**OD well 1**	0.35	0.32	0.32	0.34	0.35	0.24	0.17	0.16	0.14
**OD well 2**	0.34	0.33	0.34	0.34	0.35	0.23	0.17	0.15	0.18
**OD well 3**	0.37	0.36	0.35	0.35	0.36	0.24	0.20	0.17	0.17
**MEAN**	0.35	0.33	0.34	0.34	0.36	0.24	0.18	0.16	0.16
**SD**	0.01	0.02	0.01	0.00	0.00	0.01	0.01	0.01	0.02
**REL [%]**	**100.0**	**95.1**	**95.6**	**97.1**	**100.9**	**67.4**	**51.7**	**46.3**	**46.1**
**CV [%]**	3.42	5.91	3.80	1.24	1.01	2.60	8.12	6.49	11.84

* 0.1 % DMSO

120 h incubation time

	ctrl.*	1 µM	5 µM	10 µM	20 µM	40 µM	60 µM	80 µM	100 µM
**OD well 1**	1.93	2.07	2.04	1.47	1.07	0.59	0.40	0.27	0.35
**OD well 2**	1.98	2.28	1.94	1.58	1.06	0.55	0.36	0.25	0.37
**OD well 3**	1.79	2.06	2.14	1.50	0.99	0.55	0.38	0.23	0.38
**MEAN**	1.90	2.14	2.04	1.52	1.04	0.56	0.38	0.25	0.37
**SD**	0.10	0.12	0.10	0.05	0.04	0.02	0.02	0.02	0.02
**REL [%]**	**100.0**	**112.5**	**107.5**	**80.0**	**54.9**	**29.7**	**20.1**	**13.2**	**19.3**
**CV [%]**	5.24	5.71	5.07	3.48	4.10	3.98	4.94	7.69	4.94

* 0.1 % DMSO

Experiment 5

48 h incubation time

	ctrl.*	1 µM	5 µM	10 µM	20 µM	40 µM	60 µM	80 µM	100 µM
**OD well 1**	0.21	0.21	0.20	0.19	0.13	0.14	0.14	0.15	0.12
**OD well 2**	0.20	0.20	0.21	0.19	0.15	0.15	0.14	0.14	0.12
**OD well 3**	0.20	0.20	0.20	0.19	0.16	0.16	0.15	0.14	0.14
**MEAN**	0.20	0.20	0.20	0.19	0.15	0.15	0.14	0.14	0.12
**SD**	0.01	0.01	0.01	0.00	0.01	0.01	0.01	0.01	0.01
**REL [%]**	**100.0**	**100.5**	**100.5**	**94.8**	**73.7**	**74.5**	**69.8**	**70.8**	**61.3**
**CV [%]**	4.16	3.51	2.56	1.06	9.42	5.43	3.67	5.58	8.64

* 0.1 % DMSO

72 h incubation time

	ctrl.*	1 µM	5 µM	10 µM	20 µM	40 µM	60 µM	80 µM	100 µM
**OD well 1**	0.32	0.29	0.31	0.31	0.30	0.21	0.17	0.15	0.14
**OD well 2**	0.32	0.30	0.32	0.32	0.29	0.20	0.15	0.14	0.14
**OD well 3**	0.31	0.30	0.29	0.31	0.30	0.19	0.16	0.15	0.14
**MEAN**	0.31	0.30	0.31	0.32	0.30	0.20	0.16	0.14	0.14
**SD**	0.00	0.01	0.01	0.01	0.00	0.01	0.01	0.01	0.00
**REL [%]**	**100.0**	**94.2**	**97.3**	**100.4**	**94.0**	**63.3**	**51.0**	**45.6**	**43.6**
**CV [%]**	0.73	2.48	3.88	1.78	0.56	4.91	6.16	4.18	0.62

* 0.1 % DMSO

120 h incubation time

	ctrl.*	1 µM	5 µM	10 µM	20 µM	40 µM	60 µM	80 µM	100 µM
**OD well 1**	1.56	1.67	1.88	1.51	1.43	0.39	0.26	0.20	0.17
**OD well 2**	1.60	1.72	1.81	1.65	1.41	0.44	0.25	0.20	0.17
**OD well 3**	1.66	1.76	1.72	1.68	1.46	0.42	0.26	0.20	0.17
**MEAN**	1.60	1.72	1.81	1.61	1.43	0.42	0.26	0.20	0.17
**SD**	0.05	0.05	0.08	0.09	0.02	0.03	0.01	0.01	0.00
**REL [%]**	**100.0**	**106.9**	**112.6**	**100.4**	**89.3**	**25.9**	**16.1**	**12.6**	**10.6**
**CV [%]**	3.15	2.84	4.34	5.52	1.52	6.45	2.90	2.63	1.72

* 0.1 % DMSO

IRA 5

Experiment 1

48 h incubation time

	ctrl.*	1 µM	5 µM	10 µM	20 µM	40 µM	60 µM	80 µM	100 µM
**OD well 1**	0.22	0.24	0.25	0.24	0.18	0.14	0.13	0.13	0.11
**OD well 2**	0.24	0.23	0.24	0.22	0.16	0.15	0.16	0.14	0.12
**OD well 3**	0.23	0.25	0.24	0.22	0.15	0.16	0.14	0.13	0.13
**MEAN**	0.23	0.24	0.24	0.23	0.16	0.15	0.14	0.13	0.12
**SD**	0.01	0.01	0.01	0.01	0.01	0.01	0.01	0.01	0.01
**REL [%]**	**100.0**	**103.0**	**104.7**	**97.6**	**70.4**	**63.4**	**61.6**	**57.3**	**52.3**
**CV [%]**	3.52	3.85	2.08	4.58	8.98	7.83	8.61	5.82	8.64

* 0.1 % DMSO

72 h incubation time

	ctrl.*	1 µM	5 µM	10 µM	20 µM	40 µM	60 µM	80 µM	100 µM
**OD well 1**	0.31	0.32	0.33	0.27	0.25	0.25	0.23	0.20	0.15
**OD well 2**	0.33	0.32	0.31	0.29	0.24	0.24	0.20	0.19	0.13
**OD well 3**	0.36	0.34	0.33	0.29	0.26	0.26	0.24	0.21	0.14
**MEAN**	0.34	0.33	0.32	0.28	0.25	0.25	0.22	0.20	0.14
**SD**	0.03	0.01	0.01	0.01	0.01	0.01	0.02	0.01	0.01
**REL [%]**	**100.0**	**96.8**	**95.6**	**84.3**	**75.1**	**74.9**	**66.4**	**59.6**	**41.5**
**CV [%]**	7.92	3.47	2.29	5.22	3.82	3.99	9.21	5.66	7.28

* 0.1 % DMSO

120 h incubation time

	ctrl.*	1 µM	5 µM	10 µM	20 µM	40 µM	60 µM	80 µM	100 µM
**OD well 1**	1.49	1.51	1.39	1.02	0.59	0.39	0.24	0.19	0.15
**OD well 2**	1.49	1.61	1.41	1.05	0.62	0.42	0.26	0.20	0.15
**OD well 3**	1.45	1.53	1.38	1.18	0.58	0.41	0.28	0.19	0.15
**MEAN**	1.48	1.55	1.40	1.08	0.60	0.41	0.26	0.19	0.15
**SD**	0.02	0.05	0.02	0.08	0.02	0.01	0.02	0.00	0.00
**REL [%]**	**100.0**	**104.5**	**94.3**	**73.2**	**40.3**	**27.5**	**17.6**	**13.0**	**10.1**
**CV [%]**	1.63	3.42	1.09	7.61	2.93	3.17	8.74	2.00	2.57

* 0.1 % DMSO

Experiment 2

48 h incubation time

	ctrl.*	1 µM	5 µM	10 µM	20 µM	40 µM	60 µM	80 µM	100 µM
**OD well 1**	0.23	0.23	0.20	0.21	0.17	0.18	0.17	0.14	0.12
**OD well 2**	0.23	0.23	0.23	0.21	0.18	0.17	0.15	0.15	0.12
**OD well 3**	0.24	0.25	0.23	0.21	0.19	0.18	0.17	0.14	0.13
**MEAN**	0.24	0.24	0.22	0.21	0.18	0.17	0.16	0.14	0.12
**SD**	0.00	0.01	0.02	0.00	0.01	0.00	0.01	0.00	0.01
**REL [%]**	**100.0**	**100.3**	**94.2**	**88.3**	**76.0**	**74.0**	**68.8**	**60.8**	**51.4**
**CV [%]**	1.90	5.45	7.24	0.92	3.44	1.66	4.59	2.94	5.62

* 0.1 % DMSO

72 h incubation time

	ctrl.*	1 µM	5 µM	10 µM	20 µM	40 µM	60 µM	80 µM	100 µM
**OD well 1**	0.41	0.39	0.34	0.28	0.22	0.22	0.19	0.16	0.15
**OD well 2**	0.37	0.37	0.35	0.29	0.22	0.20	0.17	0.16	0.15
**OD well 3**	0.42	0.36	0.32	0.28	0.24	0.20	0.17	0.16	0.14
**MEAN**	0.40	0.38	0.33	0.29	0.22	0.20	0.18	0.16	0.15
**SD**	0.03	0.01	0.01	0.01	0.01	0.01	0.01	0.00	0.00
**REL [%]**	**100.0**	**94.3**	**84.1**	**71.7**	**56.4**	**51.5**	**45.1**	**40.3**	**36.7**
**CV [%]**	7.41	3.18	3.33	2.45	5.92	5.12	5.60	0.68	2.61

* 0.1 % DMSO

120 h incubation time

	ctrl.*	1 µM	5 µM	10 µM	20 µM	40 µM	60 µM	80 µM	100 µM
**OD well 1**	1.72	1.59	1.25	0.75	0.49	0.32	0.26	0.20	0.22
**OD well 2**	1.72	1.60	1.28	0.83	0.45	0.31	0.23	0.22	0.20
**OD well 3**	1.77	1.46	1.29	0.81	0.50	0.28	0.24	0.17	0.14
**MEAN**	1.73	1.55	1.27	0.79	0.48	0.30	0.24	0.20	0.19
**SD**	0.03	0.08	0.02	0.04	0.03	0.02	0.01	0.03	0.04
**REL [%]**	**100.0**	**89.4**	**73.5**	**45.8**	**27.6**	**17.4**	**14.0**	**11.4**	**10.9**
**CV [%]**	1.81	5.27	1.68	5.35	5.62	6.15	5.39	13.36	21.80

* 0.1 % DMSO

Experiment 3

48 h incubation time

	ctrl.*	1 µM	5 µM	10 µM	20 µM	40 µM	60 µM	80 µM	100 µM
**OD well 1**	0.25	0.25	0.24	0.22	0.19	0.20	0.18	0.15	0.14
**OD well 2**	0.24	0.26	0.23	0.22	0.22	0.21	0.16	0.15	0.14
**OD well 3**	0.25	0.24	0.23	0.22	0.19	0.20	0.16	0.15	0.15
**MEAN**	0.25	0.25	0.24	0.22	0.20	0.20	0.17	0.15	0.14
**SD**	0.00	0.01	0.00	0.00	0.02	0.00	0.01	0.00	0.00
**REL [%]**	**100.0**	**102.2**	**95.3**	**88.5**	**80.9**	**82.0**	**67.7**	**60.2**	**57.8**
**CV [%]**	1.89	4.63	1.37	0.32	9.23	2.00	5.19	1.17	2.93

* 0.1 % DMSO

72 h incubation time

	ctrl.*	1 µM	5 µM	10 µM	20 µM	40 µM	60 µM	80 µM	100 µM
**OD well 1**	0.33	0.35	0.29	0.29	0.24	0.21	0.20	0.13	0.11
**OD well 2**	0.36	0.34	0.29	0.29	0.22	0.19	0.16	0.14	0.12
**OD well 3**	0.37	0.33	0.31	0.30	0.23	0.20	0.18	0.14	0.11
**MEAN**	0.36	0.34	0.30	0.29	0.23	0.20	0.18	0.13	0.11
**SD**	0.02	0.01	0.01	0.01	0.01	0.01	0.02	0.00	0.00
**REL [%]**	**100.0**	**96.0**	**83.4**	**81.6**	**64.4**	**56.7**	**50.4**	**37.8**	**31.8**
**CV [%]**	5.67	3.12	2.49	1.98	2.77	4.55	10.99	2.27	3.60

* 0.1 % DMSO

120 h incubation time

	ctrl.*	1 µM	5 µM	10 µM	20 µM	40 µM	60 µM	80 µM	100 µM
**OD well 1**	1.62	1.67	1.51	0.99	0.51	0.54	0.30	0.18	0.12
**OD well 2**	1.66	1.61	1.41	0.99	0.50	0.47	0.28	0.16	0.14
**OD well 3**	1.74	1.71	1.51	0.98	0.65	0.55	0.28	0.18	0.14
**MEAN**	1.68	1.66	1.48	0.99	0.55	0.52	0.29	0.17	0.14
**SD**	0.06	0.05	0.06	0.00	0.08	0.04	0.01	0.01	0.01
**REL [%]**	**100.0**	**99.2**	**88.1**	**58.9**	**32.9**	**31.0**	**17.1**	**10.4**	**8.1**
**CV [%]**	3.53	2.86	4.09	0.46	15.19	8.29	4.71	6.70	7.48

* 0.1 % DMSO

Experiment 4

48 h incubation time

	ctrl.*	1 µM	5 µM	10 µM	20 µM	40 µM	60 µM	80 µM	100 µM
**OD well 1**	0.25	0.26	0.24	0.24	0.24	0.22	0.18	0.16	0.13
**OD well 2**	0.27	0.24	0.23	0.22	0.23	0.20	0.18	0.13	0.13
**OD well 3**	0.24	0.24	0.25	0.24	0.21	0.20	0.17	0.16	0.14
**MEAN**	0.25	0.25	0.24	0.23	0.23	0.21	0.18	0.15	0.13
**SD**	0.02	0.01	0.01	0.01	0.01	0.01	0.00	0.02	0.01
**REL [%]**	**100.0**	**97.8**	**95.5**	**91.5**	**89.4**	**82.3**	**69.8**	**60.2**	**52.4**
**CV [%]**	6.56	5.13	5.96	4.55	5.28	6.70	1.84	10.07	4.71

* 0.1 % DMSO

72 h incubation time

	ctrl.*	1 µM	5 µM	10 µM	20 µM	40 µM	60 µM	80 µM	100 µM
**OD well 1**	0.37	0.37	0.32	0.27	0.22	0.21	0.21	0.15	0.14
**OD well 2**	0.35	0.36	0.32	0.30	0.22	0.21	0.18	0.15	0.13
**OD well 3**	0.35	0.35	0.35	0.31	0.22	0.21	0.20	0.15	0.14
**MEAN**	0.36	0.36	0.33	0.29	0.22	0.21	0.20	0.15	0.14
**SD**	0.01	0.01	0.01	0.02	0.00	0.00	0.02	0.00	0.00
**REL [%]**	**100.0**	**101.4**	**92.3**	**82.0**	**62.6**	**58.1**	**56.0**	**42.4**	**38.4**
**CV [%]**	3.02	2.76	4.52	6.80	0.92	1.06	8.38	3.23	1.99

* 0.1 % DMSO

120 h incubation time

	ctrl.*	1 µM	5 µM	10 µM	20 µM	40 µM	60 µM	80 µM	100 µM
**OD well 1**	1.74	1.80	1.91	0.96	0.83	0.56	0.43	0.24	0.23
**OD well 2**	1.68	1.65	1.85	1.00	0.77	0.55	0.38	0.21	0.25
**OD well 3**	1.72	1.90	1.83	1.01	0.84	0.57	0.40	0.26	0.23
**MEAN**	1.71	1.78	1.86	0.99	0.81	0.56	0.40	0.24	0.24
**SD**	0.03	0.12	0.04	0.03	0.03	0.01	0.03	0.03	0.01
**REL [%]**	**100.0**	**104.1**	**108.7**	**57.6**	**47.5**	**32.7**	**23.3**	**13.9**	**13.7**
**CV [%]**	1.99	6.96	2.05	2.59	4.13	2.11	6.29	10.70	4.21

* 0.1 % DMSO

Experiment 5

48 h incubation time

	ctrl.*	1 µM	5 µM	10 µM	20 µM	40 µM	60 µM	80 µM	100 µM
**OD well 1**	0.20	0.19	0.19	0.17	0.16	0.15	0.16	0.14	0.13
**OD well 2**	0.22	0.19	0.19	0.17	0.15	0.17	0.15	0.14	0.12
**OD well 3**	0.20	0.19	0.20	0.17	0.15	0.16	0.14	0.13	0.13
**MEAN**	0.21	0.19	0.19	0.17	0.16	0.16	0.15	0.14	0.12
**SD**	0.01	0.00	0.01	0.00	0.01	0.01	0.01	0.01	0.00
**REL [%]**	**100.0**	**92.3**	**93.6**	**82.3**	**75.8**	**77.6**	**71.3**	**65.8**	**60.1**
**CV [%]**	5.20	0.59	4.20	0.87	3.64	4.10	4.57	4.85	2.83

* 0.1 % DMSO

72 h incubation time

	ctrl.*	1 µM	5 µM	10 µM	20 µM	40 µM	60 µM	80 µM	100 µM
**OD well 1**	0.35	0.38	0.33	0.27	0.23	0.19	0.15	0.14	0.13
**OD well 2**	0.34	0.35	0.32	0.29	0.23	0.20	0.15	0.13	0.12
**OD well 3**	0.36	0.36	0.33	0.27	0.21	0.20	0.15	0.13	0.13
**MEAN**	0.35	0.36	0.33	0.27	0.23	0.20	0.15	0.13	0.13
**SD**	0.01	0.01	0.01	0.01	0.01	0.00	0.00	0.01	0.01
**REL [%]**	**100.0**	**104.4**	**93.4**	**78.2**	**65.1**	**56.8**	**43.7**	**38.6**	**36.0**
**CV [%]**	2.55	3.43	2.12	4.08	5.14	2.46	1.50	3.99	4.66

* 0.1 % DMSO

120 h incubation time

	ctrl.*	1 µM	5 µM	10 µM	20 µM	40 µM	60 µM	80 µM	100 µM
**OD well 1**	1.69	1.63	1.45	1.10	0.63	0.37	0.15	0.14	0.11
**OD well 2**	1.54	1.52	1.41	0.94	0.52	0.32	0.12	0.13	0.10
**OD well 3**	1.43	1.51	1.46	1.09	0.70	0.41	0.14	0.15	0.11
**MEAN**	1.55	1.56	1.44	1.04	0.62	0.37	0.13	0.14	0.11
**SD**	0.13	0.07	0.03	0.09	0.09	0.04	0.02	0.01	0.00
**REL [%]**	**100.0**	**100.1**	**92.7**	**67.1**	**39.7**	**23.7**	**8.7**	**8.8**	**6.8**
**CV [%]**	8.32	4.28	1.76	8.53	14.42	11.55	11.77	7.60	1.74

* 0.1 % DMSO

LNCaP cells

Resveratrol

Experiment 1

48 h incubation time

	ctrl.*	1 µM	5 µM	10 µM	20 µM	40 µM	60 µM	80 µM	100 µM
**OD well 1**	0.21	0.20	0.20	0.20	0.18	0.17	0.16	0.15	0.15
**OD well 2**	0.20	0.20	0.18	0.18	0.16	0.18	0.18	0.15	0.14
**OD well 3**	0.19	0.19	0.19	0.18	0.17	0.17	0.16	0.14	0.14
**MEAN**	0.20	0.20	0.19	0.19	0.17	0.17	0.17	0.15	0.14
**SD**	0.01	0.01	0.01	0.01	0.01	0.01	0.01	0.00	0.00
**REL [%]**	**100.0**	**98.3**	**95.3**	**93.4**	**86.6**	**86.6**	**83.7**	**74.2**	**71.3**
**CV [%]**	6.57	3.22	4.82	5.86	6.35	4.25	5.20	2.27	1.81

* 0.1 % DMSO

72 h incubation time

	ctrl.*	1 µM	5 µM	10 µM	20 µM	40 µM	60 µM	80 µM	100 µM
**OD well 1**	0.26	0.25	0.23	0.20	0.20	0.18	0.19	0.16	0.14
**OD well 2**	0.27	0.26	0.23	0.20	0.19	0.17	0.17	0.15	0.13
**OD well 3**	0.25	0.24	0.21	0.18	0.17	0.17	0.17	0.14	0.12
**MEAN**	0.26	0.25	0.22	0.20	0.18	0.18	0.18	0.15	0.13
**SD**	0.01	0.01	0.01	0.01	0.02	0.01	0.01	0.01	0.01
**REL [%]**	**100.0**	**95.5**	**84.1**	**75.2**	**70.4**	**67.4**	**67.3**	**56.8**	**50.6**
**CV [%]**	3.92	5.71	5.25	6.84	8.57	4.41	8.11	6.39	7.33

* 0.1 % DMSO

120 h incubation time

	ctrl.*	1 µM	5 µM	10 µM	20 µM	40 µM	60 µM	80 µM	100 µM
**OD well 1**	0.45	0.47	0.42	0.31	0.23	0.18	0.20	0.15	0.13
**OD well 2**	0.47	0.49	0.39	0.30	0.23	0.19	0.20	0.16	0.14
**OD well 3**	0.47	0.51	0.37	0.28	0.21	0.18	0.18	0.15	0.13
**MEAN**	0.46	0.49	0.39	0.30	0.23	0.18	0.19	0.15	0.13
**SD**	0.01	0.02	0.02	0.02	0.01	0.00	0.01	0.00	0.00
**REL [%]**	**100.0**	**105.8**	**84.6**	**64.4**	**48.5**	**39.3**	**41.0**	**32.5**	**28.7**
**CV [%]**	2.42	3.62	5.64	5.16	5.56	2.44	6.78	3.22	3.75

* 0.1 % DMSO

Experiment 2

48 h incubation time

	ctrl.*	1 µM	5 µM	10 µM	20 µM	40 µM	60 µM	80 µM	100 µM
**OD well 1**	0.23	0.24	0.23	0.22	0.20	0.19	0.19	0.17	0.15
**OD well 2**	0.22	0.22	0.20	0.21	0.19	0.19	0.18	0.17	0.15
**OD well 3**	0.22	0.22	0.21	0.20	0.20	0.18	0.18	0.17	0.16
**MEAN**	0.22	0.23	0.21	0.21	0.19	0.19	0.18	0.17	0.16
**SD**	0.01	0.01	0.01	0.01	0.01	0.00	0.00	0.00	0.01
**REL [%]**	**100.0**	**101.6**	**95.9**	**93.0**	**86.4**	**83.5**	**82.5**	**75.0**	**69.4**
**CV [%]**	3.10	5.02	5.88	4.68	3.50	1.28	1.90	0.57	5.33

* 0.1 % DMSO

72 h incubation time

	ctrl.*	1 µM	5 µM	10 µM	20 µM	40 µM	60 µM	80 µM	100 µM
**OD well 1**	0.28	0.27	0.25	0.22	0.20	0.19	0.19	0.16	0.15
**OD well 2**	0.28	0.28	0.23	0.22	0.18	0.17	0.17	0.16	0.16
**OD well 3**	0.28	0.26	0.22	0.20	0.19	0.17	0.17	0.15	0.14
**MEAN**	0.28	0.27	0.24	0.21	0.19	0.18	0.18	0.16	0.15
**SD**	0.00	0.01	0.01	0.01	0.01	0.01	0.01	0.00	0.01
**REL [%]**	**100.0**	**96.5**	**84.0**	**75.0**	**67.9**	**63.0**	**63.2**	**55.7**	**53.0**
**CV [%]**	0.38	3.36	5.93	6.05	6.33	5.56	5.17	3.14	6.41

* 0.1 % DMSO

120 h incubation time

	ctrl.*	1 µM	5 µM	10 µM	20 µM	40 µM	60 µM	80 µM	100 µM
**OD well 1**	0.55	0.52	0.46	0.32	0.27	0.18	0.21	0.15	0.14
**OD well 2**	0.54	0.54	0.39	0.33	0.25	0.17	0.18	0.15	0.14
**OD well 3**	0.54	0.55	0.38	0.28	0.23	0.18	0.18	0.15	0.14
**MEAN**	0.54	0.54	0.41	0.31	0.25	0.18	0.19	0.15	0.14
**SD**	0.01	0.01	0.04	0.02	0.02	0.00	0.01	0.00	0.00
**REL [%]**	**100.0**	**99.6**	**76.3**	**57.5**	**45.9**	**32.6**	**35.2**	**28.0**	**26.0**
**CV [%]**	1.24	2.21	10.30	7.50	8.53	2.40	7.11	2.36	2.16

* 0.1 % DMSO

Experiment 3

48 h incubation time

	ctrl.*	1 µM	5 µM	10 µM	20 µM	40 µM	60 µM	80 µM	100 µM
**OD well 1**	0.21	0.22	0.23	0.23	0.20	0.19	0.18	0.17	0.16
**OD well 2**	0.22	0.24	0.21	0.22	0.21	0.19	0.18	0.17	0.14
**OD well 3**	0.21	0.22	0.20	0.22	0.22	0.20	0.18	0.16	0.13
**MEAN**	0.22	0.23	0.21	0.22	0.21	0.19	0.18	0.17	0.14
**SD**	0.00	0.01	0.01	0.01	0.01	0.01	0.00	0.01	0.01
**REL [%]**	**100.0**	**104.3**	**99.2**	**102.3**	**97.0**	**88.6**	**83.4**	**77.8**	**66.6**
**CV [%]**	1.78	4.15	6.93	2.29	3.72	4.05	1.39	3.97	7.45

* 0.1 % DMSO

72 h incubation time

	ctrl.*	1 µM	5 µM	10 µM	20 µM	40 µM	60 µM	80 µM	100 µM
**OD well 1**	0.26	0.26	0.24	0.21	0.19	0.17	0.17	0.17	0.14
**OD well 2**	0.25	0.25	0.24	0.21	0.19	0.17	0.17	0.15	0.13
**OD well 3**	0.25	0.26	0.21	0.20	0.18	0.16	0.16	0.15	0.13
**MEAN**	0.25	0.26	0.23	0.20	0.19	0.17	0.16	0.16	0.13
**SD**	0.00	0.01	0.02	0.01	0.00	0.01	0.00	0.01	0.00
**REL [%]**	**100.0**	**100.8**	**90.5**	**80.3**	**73.4**	**66.5**	**64.7**	**61.3**	**53.3**
**CV [%]**	1.82	3.79	6.61	3.37	2.29	4.16	2.58	6.36	2.75

* 0.1 % DMSO

120 h incubation time

	ctrl.*	1 µM	5 µM	10 µM	20 µM	40 µM	60 µM	80 µM	100 µM
**OD well 1**	0.44	0.47	0.46	0.28	0.26	0.18	0.18	0.14	0.13
**OD well 2**	0.45	0.46	0.45	0.33	0.24	0.17	0.18	0.12	0.13
**OD well 3**	0.44	0.44	0.45	0.33	0.28	0.18	0.18	0.16	0.12
**MEAN**	0.44	0.46	0.45	0.31	0.26	0.18	0.18	0.14	0.13
**SD**	0.01	0.01	0.01	0.03	0.02	0.01	0.00	0.02	0.01
**REL [%]**	**100.0**	**103.5**	**102.2**	**71.2**	**58.8**	**39.7**	**40.5**	**31.9**	**29.1**
**CV [%]**	1.18	2.79	1.34	8.53	6.94	3.96	1.79	12.09	5.29

* 0.1 % DMSO

Experiment 4

48 h incubation time

	ctrl.*	1 µM	5 µM	10 µM	20 µM	40 µM	60 µM	80 µM	100 µM
**OD well 1**	0.20	0.20	0.19	0.20	0.19	0.18	0.18	0.15	0.14
**OD well 2**	0.21	0.21	0.21	0.20	0.18	0.19	0.17	0.15	0.15
**OD well 3**	0.23	0.20	0.21	0.20	0.18	0.19	0.17	0.15	0.15
**MEAN**	0.21	0.20	0.20	0.20	0.18	0.19	0.17	0.15	0.15
**SD**	0.01	0.00	0.01	0.00	0.01	0.01	0.00	0.00	0.01
**REL [%]**	**100.0**	**96.1**	**95.9**	**95.7**	**87.5**	**88.2**	**81.9**	**71.9**	**69.2**
**CV [%]**	6.76	2.25	3.76	1.59	3.12	3.34	2.04	1.96	4.53

* 0.1 % DMSO

72 h incubation time

	ctrl.*	1 µM	5 µM	10 µM	20 µM	40 µM	60 µM	80 µM	100 µM
**OD well 1**	0.24	0.22	0.21	0.22	0.20	0.19	0.15	0.14	0.14
**OD well 2**	0.24	0.23	0.21	0.22	0.20	0.19	0.16	0.14	0.14
**OD well 3**	0.25	0.22	0.22	0.22	0.20	0.18	0.16	0.16	0.16
**MEAN**	0.25	0.23	0.21	0.22	0.20	0.19	0.16	0.15	0.15
**SD**	0.01	0.01	0.01	0.00	0.00	0.01	0.01	0.01	0.01
**REL [%]**	**100.0**	**92.0**	**87.2**	**88.5**	**81.2**	**77.1**	**65.4**	**60.5**	**60.3**
**CV [%]**	2.10	2.85	3.51	0.74	1.46	4.83	4.17	6.21	6.26

* 0.1 % DMSO

120 h incubation time

	ctrl.*	1 µM	5 µM	10 µM	20 µM	40 µM	60 µM	80 µM	100 µM
**OD well 1**	0.47	0.48	0.35	0.35	0.28	0.22	0.18	0.17	0.18
**OD well 2**	0.46	0.50	0.40	0.29	0.26	0.20	0.19	0.17	0.15
**OD well 3**	0.47	0.54	0.41	0.33	0.30	0.22	0.19	0.17	0.16
**MEAN**	0.46	0.51	0.39	0.32	0.28	0.21	0.19	0.17	0.16
**SD**	0.01	0.03	0.03	0.03	0.02	0.01	0.00	0.00	0.01
**REL [%]**	**100.0**	**109.4**	**83.4**	**69.7**	**59.9**	**46.2**	**40.4**	**36.5**	**35.1**
**CV [%]**	1.67	5.69	7.95	9.82	7.73	5.67	2.49	2.02	7.67

* 0.1 % DMSO

Experiment 5

48 h incubation time

	ctrl.*	1 µM	5 µM	10 µM	20 µM	40 µM	60 µM	80 µM	100 µM
**OD well 1**	0.18	0.17	0.19	0.18	0.20	0.18	0.15	0.16	0.14
**OD well 2**	0.20	0.19	0.18	0.19	0.17	0.15	0.14	0.15	0.15
**OD well 3**	0.20	0.18	0.18	0.18	0.18	0.16	0.15	0.14	0.14
**MEAN**	0.19	0.18	0.18	0.18	0.18	0.16	0.15	0.15	0.15
**SD**	0.01	0.01	0.01	0.00	0.01	0.01	0.00	0.01	0.01
**REL [%]**	**100.0**	**93.0**	**94.8**	**94.7**	**95.2**	**84.4**	**76.0**	**76.5**	**74.7**
**CV [%]**	6.11	3.64	3.15	0.99	6.14	8.46	2.06	4.95	5.45

* 0.1 % DMSO

72 h incubation time

	ctrl.*	1 µM	5 µM	10 µM	20 µM	40 µM	60 µM	80 µM	100 µM
**OD well 1**	0.25	0.24	0.23	0.19	0.18	0.18	0.17	0.17	0.15
**OD well 2**	0.26	0.23	0.23	0.20	0.18	0.16	0.18	0.16	0.14
**OD well 3**	0.24	0.23	0.21	0.18	0.17	0.17	0.16	0.14	0.13
**MEAN**	0.25	0.23	0.22	0.19	0.18	0.17	0.17	0.15	0.14
**SD**	0.01	0.01	0.01	0.01	0.00	0.01	0.01	0.01	0.01
**REL [%]**	**100.0**	**93.3**	**88.9**	**76.6**	**70.9**	**68.1**	**68.6**	**61.0**	**56.1**
**CV [%]**	3.07	4.07	3.90	3.08	1.27	5.57	4.03	9.23	5.78

* 0.1 % DMSO

120 h incubation time

	ctrl.*	1 µM	5 µM	10 µM	20 µM	40 µM	60 µM	80 µM	100 µM
**OD well 1**	0.46	0.45	0.41	0.25	0.19	0.18	0.12	0.17	0.14
**OD well 2**	0.42	0.45	0.43	0.24	0.21	0.20	0.16	0.16	0.16
**OD well 3**	0.42	0.43	0.37	0.24	0.20	0.22	0.18	0.16	0.16
**MEAN**	0.43	0.45	0.40	0.24	0.20	0.20	0.15	0.16	0.15
**SD**	0.03	0.01	0.03	0.00	0.01	0.02	0.03	0.00	0.01
**REL [%]**	**100.0**	**103.2**	**93.2**	**56.4**	**46.9**	**47.0**	**35.8**	**37.5**	**35.9**
**CV [%]**	5.95	3.29	7.89	1.96	5.02	11.36	19.45	1.81	6.86

* 0.1 % DMSO

IRA 5

Experiment 1

48 h incubation time

	ctrl.*	1 µM	5 µM	10 µM	20 µM	40 µM	60 µM	80 µM	100 µM
**OD well 1**	0.20	0.20	0.18	0.17	0.15	0.15	0.14	0.14	0.13
**OD well 2**	0.18	0.18	0.18	0.18	0.15	0.16	0.14	0.15	0.14
**OD well 3**	0.20	0.21	0.20	0.17	0.16	0.16	0.17	0.15	0.13
**MEAN**	0.19	0.20	0.19	0.17	0.15	0.16	0.15	0.15	0.13
**SD**	0.01	0.01	0.01	0.00	0.01	0.00	0.01	0.01	0.00
**REL [%]**	**100.0**	**101.9**	**95.9**	**89.5**	**79.6**	**80.1**	**78.3**	**75.0**	**67.4**
**CV [%]**	5.40	6.17	4.73	2.16	3.30	1.62	8.76	4.63	3.60

* 0.1 % DMSO

72 h incubation time

	ctrl.*	1 µM	5 µM	10 µM	20 µM	40 µM	60 µM	80 µM	100 µM
**OD well 1**	0.26	0.23	0.20	0.18	0.17	0.16	0.15	0.13	0.13
**OD well 2**	0.25	0.22	0.18	0.17	0.15	0.15	0.15	0.13	0.12
**OD well 3**	0.24	0.24	0.18	0.18	0.17	0.16	0.16	0.15	0.13
**MEAN**	0.25	0.23	0.18	0.18	0.17	0.15	0.15	0.14	0.13
**SD**	0.01	0.01	0.01	0.01	0.01	0.01	0.01	0.01	0.00
**REL [%]**	**100.0**	**94.2**	**74.3**	**72.0**	**67.1**	**62.7**	**60.4**	**55.1**	**50.8**
**CV [%]**	4.06	3.93	6.14	3.90	6.46	4.62	4.60	6.34	2.06

* 0.1 % DMSO

120 h incubation time

	ctrl.*	1 µM	5 µM	10 µM	20 µM	40 µM	60 µM	80 µM	100 µM
**OD well 1**	0.41	0.38	0.33	0.26	0.22	0.17	0.16	0.13	0.13
**OD well 2**	0.42	0.35	0.34	0.27	0.22	0.17	0.16	0.13	0.12
**OD well 3**	0.40	0.35	0.31	0.23	0.20	0.17	0.18	0.14	0.13
**MEAN**	0.41	0.36	0.33	0.25	0.21	0.17	0.17	0.13	0.13
**SD**	0.01	0.02	0.01	0.02	0.01	0.00	0.01	0.01	0.00
**REL [%]**	**100.0**	**88.5**	**80.0**	**61.3**	**51.7**	**40.9**	**41.2**	**32.9**	**30.9**
**CV [%]**	3.00	4.77	4.46	7.58	3.00	1.22	5.39	4.51	3.64

* 0.1 % DMSO

Experiment 2

48 h incubation time

	ctrl.*	1 µM	5 µM	10 µM	20 µM	40 µM	60 µM	80 µM	100 µM
**OD well 1**	0.22	0.22	0.21	0.20	0.17	0.16	0.17	0.16	0.14
**OD well 2**	0.20	0.21	0.20	0.19	0.18	0.17	0.16	0.15	0.15
**OD well 3**	0.22	0.22	0.22	0.18	0.18	0.17	0.17	0.16	0.15
**MEAN**	0.22	0.22	0.21	0.19	0.18	0.17	0.17	0.16	0.15
**SD**	0.01	0.01	0.01	0.01	0.01	0.00	0.00	0.00	0.00
**REL [%]**	**100.0**	**99.9**	**98.2**	**89.3**	**81.6**	**78.4**	**78.3**	**73.5**	**68.1**
**CV [%]**	6.12	3.21	6.49	4.41	3.25	2.39	2.62	2.45	3.17

* 0.1 % DMSO

72 h incubation time

	ctrl.*	1 µM	5 µM	10 µM	20 µM	40 µM	60 µM	80 µM	100 µM
**OD well 1**	0.27	0.24	0.20	0.19	0.17	0.16	0.15	0.13	0.14
**OD well 2**	0.27	0.24	0.18	0.18	0.17	0.15	0.15	0.13	0.13
**OD well 3**	0.23	0.26	0.18	0.19	0.17	0.17	0.16	0.14	0.13
**MEAN**	0.25	0.25	0.19	0.18	0.17	0.16	0.15	0.14	0.13
**SD**	0.02	0.01	0.01	0.00	0.00	0.01	0.01	0.00	0.00
**REL [%]**	**100.0**	**96.5**	**74.2**	**72.5**	**67.1**	**62.1**	**60.2**	**53.6**	**52.6**
**CV [%]**	8.53	4.11	5.52	1.72	2.78	6.14	4.15	3.21	2.77

* 0.1 % DMSO

120 h incubation time

	ctrl.*	1 µM	5 µM	10 µM	20 µM	40 µM	60 µM	80 µM	100 µM
**OD well 1**	0.45	0.38	0.34	0.25	0.24	0.17	0.18	0.13	0.13
**OD well 2**	0.44	0.38	0.35	0.28	0.23	0.16	0.17	0.14	0.12
**OD well 3**	0.43	0.39	0.34	0.25	0.22	0.16	0.17	0.14	0.13
**MEAN**	0.44	0.38	0.34	0.26	0.23	0.16	0.17	0.14	0.13
**SD**	0.01	0.00	0.01	0.02	0.01	0.01	0.01	0.00	0.00
**REL [%]**	**100.0**	**86.1**	**77.3**	**58.5**	**52.0**	**36.5**	**38.8**	**30.9**	**28.7**
**CV [%]**	2.51	1.22	1.71	6.22	2.54	3.45	3.02	3.11	2.23

* 0.1 % DMSO

Experiment 3

48 h incubation time

	ctrl.*	1 µM	5 µM	10 µM	20 µM	40 µM	60 µM	80 µM	100 µM
**OD well 1**	0.25	0.20	0.22	0.22	0.19	0.18	0.17	0.16	0.13
**OD well 2**	0.23	0.23	0.22	0.20	0.20	0.19	0.16	0.17	0.15
**OD well 3**	0.22	0.23	0.21	0.21	0.20	0.18	0.19	0.18	0.14
**MEAN**	0.23	0.22	0.22	0.21	0.20	0.18	0.18	0.17	0.14
**SD**	0.02	0.02	0.00	0.01	0.00	0.01	0.01	0.01	0.01
**REL [%]**	**100.0**	**95.6**	**91.8**	**90.1**	**83.7**	**78.7**	**75.3**	**72.3**	**59.3**
**CV [%]**	7.91	8.41	2.27	5.27	1.76	3.97	8.45	5.12	7.00

* 0.1 % DMSO

72 h incubation time

	ctrl.*	1 µM	5 µM	10 µM	20 µM	40 µM	60 µM	80 µM	100 µM
**OD well 1**	0.26	0.24	0.20	0.18	0.16	0.17	0.16	0.16	0.13
**OD well 2**	0.25	0.25	0.20	0.18	0.14	0.16	0.16	0.14	0.13
**OD well 3**	0.27	0.25	0.21	0.19	0.16	0.17	0.17	0.16	0.14
**MEAN**	0.26	0.25	0.20	0.18	0.15	0.17	0.17	0.15	0.13
**SD**	0.01	0.00	0.00	0.01	0.01	0.00	0.01	0.01	0.01
**REL [%]**	**100.0**	**94.5**	**78.3**	**69.5**	**59.2**	**63.6**	**63.6**	**58.8**	**51.1**
**CV [%]**	2.85	1.10	1.15	3.33	6.42	2.96	3.42	5.64	3.95

* 0.1 % DMSO

120 h incubation time

	ctrl.*	1 µM	5 µM	10 µM	20 µM	40 µM	60 µM	80 µM	100 µM
**OD well 1**	0.40	0.38	0.33	0.26	0.23	0.17	0.16	0.13	0.11
**OD well 2**	0.39	0.38	0.31	0.25	0.21	0.16	0.16	0.15	0.11
**OD well 3**	0.42	0.36	0.31	0.27	0.23	0.18	0.15	0.13	0.12
**MEAN**	0.40	0.37	0.32	0.26	0.22	0.17	0.16	0.14	0.11
**SD**	0.01	0.01	0.01	0.01	0.01	0.01	0.01	0.01	0.00
**REL [%]**	**100.0**	**91.7**	**78.2**	**64.1**	**55.4**	**42.6**	**39.3**	**33.7**	**27.8**
**CV [%]**	3.16	3.17	3.64	4.27	3.72	4.61	4.93	7.66	3.40

* 0.1 % DMSO

Experiment 4

48 h incubation time

	ctrl.*	1 µM	5 µM	10 µM	20 µM	40 µM	60 µM	80 µM	100 µM
**OD well 1**	0.19	0.19	0.19	0.19	0.17	0.17	0.16	0.14	0.15
**OD well 2**	0.20	0.20	0.19	0.17	0.17	0.17	0.16	0.16	0.14
**OD well 3**	0.21	0.20	0.20	0.19	0.18	0.18	0.18	0.17	0.15
**MEAN**	0.20	0.20	0.20	0.18	0.17	0.17	0.17	0.16	0.15
**SD**	0.01	0.01	0.01	0.01	0.01	0.01	0.01	0.02	0.00
**REL [%]**	**100.0**	**99.5**	**99.1**	**92.4**	**87.3**	**87.9**	**83.7**	**79.5**	**74.5**
**CV [%]**	3.38	5.36	3.22	4.83	4.09	3.57	5.84	10.12	3.24

* 0.1 % DMSO

72 h incubation time

	ctrl.*	1 µM	5 µM	10 µM	20 µM	40 µM	60 µM	80 µM	100 µM
**OD well 1**	0.25	0.21	0.20	0.18	0.17	0.16	0.16	0.13	0.14
**OD well 2**	0.23	0.22	0.18	0.17	0.16	0.16	0.14	0.12	0.13
**OD well 3**	0.25	0.23	0.20	0.20	0.18	0.17	0.17	0.14	0.15
**MEAN**	0.24	0.22	0.19	0.18	0.17	0.16	0.16	0.13	0.14
**SD**	0.01	0.01	0.01	0.01	0.01	0.01	0.01	0.01	0.01
**REL [%]**	**100.0**	**89.2**	**78.2**	**75.2**	**70.5**	**66.0**	**64.9**	**54.9**	**57.5**
**CV [%]**	4.76	3.15	5.95	6.56	6.26	3.30	8.81	7.46	5.27

* 0.1 % DMSO

120 h incubation time

	ctrl.*	1 µM	5 µM	10 µM	20 µM	40 µM	60 µM	80 µM	100 µM
**OD well 1**	0.41	0.34	0.31	0.22	0.21	0.20	0.20	0.16	0.13
**OD well 2**	0.40	0.32	0.29	0.18	0.21	0.18	0.16	0.15	0.12
**OD well 3**	0.40	0.30	0.30	0.20	0.20	0.19	0.18	0.14	0.12
**MEAN**	0.40	0.32	0.30	0.20	0.21	0.19	0.18	0.15	0.12
**SD**	0.01	0.02	0.01	0.02	0.01	0.01	0.02	0.01	0.00
**REL [%]**	**100.0**	**80.1**	**74.6**	**49.9**	**51.5**	**46.7**	**45.4**	**36.7**	**30.3**
**CV [%]**	1.89	6.22	4.09	8.76	3.02	5.30	10.02	5.87	3.34

* 0.1 % DMSO

Experiment 5

48 h incubation time

	ctrl.*	1 µM	5 µM	10 µM	20 µM	40 µM	60 µM	80 µM	100 µM
**OD well 1**	0.20	0.18	0.17	0.18	0.15	0.14	0.15	0.14	0.13
**OD well 2**	0.17	0.18	0.18	0.18	0.17	0.14	0.13	0.12	0.12
**OD well 3**	0.20	0.18	0.20	0.19	0.15	0.16	0.14	0.12	0.15
**MEAN**	0.19	0.18	0.18	0.18	0.16	0.15	0.14	0.13	0.13
**SD**	0.01	0.00	0.01	0.00	0.01	0.01	0.01	0.01	0.01
**REL [%]**	**100.0**	**95.3**	**96.2**	**97.4**	**82.7**	**77.8**	**74.2**	**67.6**	**69.7**
**CV [%]**	7.30	1.30	6.61	1.91	5.41	5.50	7.56	9.65	10.69

* 0.1 % DMSO

72 h incubation time

	ctrl.*	1 µM	5 µM	10 µM	20 µM	40 µM	60 µM	80 µM	100 µM
**OD well 1**	0.21	0.22	0.20	0.17	0.16	0.17	0.16	0.15	0.13
**OD well 2**	0.23	0.20	0.17	0.16	0.14	0.17	0.15	0.14	0.12
**OD well 3**	0.25	0.23	0.19	0.20	0.18	0.19	0.16	0.15	0.13
**MEAN**	0.23	0.22	0.19	0.18	0.16	0.18	0.16	0.15	0.13
**SD**	0.02	0.01	0.02	0.02	0.02	0.01	0.01	0.00	0.01
**REL [%]**	**100.0**	**95.3**	**82.0**	**77.7**	**69.1**	**77.4**	**68.0**	**63.9**	**56.4**
**CV [%]**	8.14	6.64	8.45	11.25	11.11	6.30	4.05	2.32	5.53

* 0.1 % DMSO

120 h incubation time

	ctrl.*	1 µM	5 µM	10 µM	20 µM	40 µM	60 µM	80 µM	100 µM
**OD well 1**	0.43	0.30	0.33	0.20	0.20	0.20	0.16	0.16	0.15
**OD well 2**	0.37	0.31	0.29	0.18	0.18	0.23	0.16	0.13	0.14
**OD well 3**	0.39	0.34	0.29	0.19	0.20	0.18	0.15	0.14	0.13
**MEAN**	0.40	0.32	0.31	0.19	0.19	0.20	0.16	0.14	0.14
**SD**	0.03	0.02	0.02	0.01	0.01	0.03	0.01	0.02	0.01
**REL [%]**	**100.0**	**80.3**	**76.6**	**47.7**	**48.4**	**51.1**	**39.8**	**35.7**	**35.5**
**CV [%]**	7.00	6.11	7.83	6.19	3.82	14.11	3.66	10.77	6.80

* 0.1 % DMSO

Raw data of the LDH activity assay

Abbreviations: B: blank; ctrl.: solvent control (0.1 % DMSO); CV: coefficient of variation; DMSO: dimethyl sulfoxide; I5: IRA 5 (1 = 1 µM, 10 = 10 µM, 50 = 50 µM, 100 = 100 µM); P: positive control (0.18 % Triton® X-100); R: resveratrol (1 = 1 µM, 10 = 10 µM, 50 = 50 µM, 100 = 100 µM); REL: relative presentation of the data as fold of the solvent control; RFU: relative fluorescence units; SD: standard deviation

HCA-7 cells

Experiment 1

6 h incubation time

	ctrl.*	P	R 1	R 10	R 50	R 100	I5 1	I5 10	I5 50	I5 100	B
**RFU well 1**	20879	39824	20362	22057	19762	19377	23020	20848	22251	21059	16542
**RFU well 2**	21360	38703	23323	21641	21668	20643	22740	20818	22632	19450	17492
**RFU well 3**	21954	43359	22027	22609	21715	20972	20829	20525	23660	22460	17174
**MEAN Blank**											17069.3
**Well 1 corr.**	3810	22755	3293	4988	2693	2308	5951	3779	5182	3990	
**Well 2 corr.**	4291	21634	6254	4572	4599	3574	5671	3749	5563	2381	
**Well 3 corr.**	4885	26290	4958	5540	4646	3903	3760	3456	6591	5391	
**MEAN**	4328.3	23559.3	4834.7	5033.0	3979.0	3261.3	5127.0	3661.0	5778.3	3920.3	
**SD**	538.5	2430.1	1484.3	485.6	1114.2	842.1	1192.4	178.5	728.8	1506.2	
**REL**	**1.0**	**5.4**	**1.1**	**1.2**	**0.9**	**0.8**	**1.2**	**0.8**	**1.3**	**0.9**	
**CV**	12.4	10.3	30.7	9.6	28.0	25.8	23.3	4.9	12.6	38.4	

* 0.1 % DMSO

24 h incubation time

	ctrl.*	P	R 1	R 10	R 50	R 100	I5 1	I5 10	I5 50	I5 100	B
**RFU well 1**	18993	39063	16441	17457	16249	15852	17689	16175	15858	15785	12522
**RFU well 2**	19863	42178	17332	15125	16924	16320	16496	16586	15550	15529	12355
**RFU well 3**	18363	42180	17980	16898	15409	14252	15928	16384	15893	15319	12422
**MEAN Blank**											12433.0
**Well 1 corr.**	6560	26630	4008	5024	3816	3419	5256	3742	3425	3352	
**Well 2 corr.**	7430	29745	4899	2692	4491	3887	4063	4153	3117	3096	
**Well 3 corr.**	5930	29747	5547	4465	2976	1819	3495	3951	3460	2886	
**MEAN**	6640.0	28707.3	4818.0	4060.3	3761.0	3041.7	4271.3	3948.7	3334.0	3111.3	
**SD**	753.2	1799.0	772.7	1217.5	759.0	1084.4	898.8	205.5	188.7	233.4	
**REL**	**1.0**	**4.3**	**0.7**	**0.6**	**0.6**	**0.5**	**0.6**	**0.6**	**0.5**	**0.5**	
**CV**	11.3	6.3	16.0	30.0	20.2	35.7	21.0	5.2	5.7	7.5	

* 0.1 % DMSO

Experiment 2

6 h incubation time

	ctrl.*	P	R 1	R 10	R 50	R 100	I5 1	I5 10	I5 50	I5 100	B
**RFU well 1**	22199	46535	22978	22507	20748	20274	22361	21065	22231	22968	16930
**RFU well 2**	20745	44699	22451	22896	20429	20572	23958	22746	22795	22086	17036
**RFU well 3**	21298	45573	23282	24101	20855	21668	21918	21559	21247	19525	17216
**MEAN Blank**											17060.7
**Well 1 corr.**	5138	29474	5917	5446	3687	3213	5300	4004	5170	5907	
**Well 2 corr.**	3684	27638	5390	5835	3368	3511	6897	5685	5734	5025	
**Well 3 corr.**	4237	28512	6221	7040	3794	4607	4857	4498	4186	2464	
**MEAN**	4353.3	28541.7	5843.0	6107.3	3616.7	3777.3	5685.0	4729.3	5030.3	4465.7	
**SD**	733.9	918.4	420.5	831.1	221.6	734.1	1073.0	864.0	783.4	1788.4	
**REL**	**1.0**	**6.6**	**1.3**	**1.4**	**0.8**	**0.9**	**1.3**	**1.1**	**1.2**	**1.0**	
**CV**	16.9	3.2	7.2	13.6	6.1	19.4	18.9	18.3	15.6	40.0	

* 0.1 % DMSO

24 h incubation time

	ctrl.*	P	R 1	R 10	R 50	R 100	I5 1	I5 10	I5 50	I5 100	B
**RFU well 1**	17767	38207	16858	16145	16019	14461	17078	16868	15946	15658	12195
**RFU well 2**	16274	36087	17497	15893	15496	14827	16685	17094	16026	15454	12844
**RFU well 3**	18921	36334	17670	16140	15769	15330	17349	17134	16558	14754	12444
**MEAN Blank**											12494.3
**Well 1 corr.**	5273	25713	4364	3651	3525	1967	4584	4374	3452	3164	
**Well 2 corr.**	3780	23593	5003	3399	3002	2333	4191	4600	3532	2960	
**Well 3 corr.**	6427	23840	5176	3646	3275	2836	4855	4640	4064	2260	
**MEAN**	5159.7	24381.7	4847.3	3565.0	3267.0	2378.3	4543.0	4537.7	3682.3	2794.3	
**SD**	1327.1	1159.3	427.7	144.1	261.6	436.3	333.9	143.4	332.7	474.1	
**REL**	**1.0**	**4.7**	**0.9**	**0.7**	**0.6**	**0.5**	**0.9**	**0.9**	**0.7**	**0.5**	
**CV**	25.7	4.8	8.8	4.0	8.0	18.3	7.3	3.2	9.0	17.0	

* 0.1 % DMSO

Experiment 3

6 h incubation time

	ctrl.*	P	R 1	R 10	R 50	R 100	I5 1	I5 10	I5 50	I5 100	B
**RFU well 1**	22260	42038	22623	22408	20342	20735	21544	21518	21668	20926	17381
**RFU well 2**	22536	41070	21803	21409	20455	19603	22477	21982	22184	20322	17397
**RFU well 3**	21851	43211	21867	21450	22332	21335	21753	20207	20514	19196	17048
**MEAN Blank**											17275.3
**Well 1 corr.**	4985	24763	5348	5133	3067	3460	4269	4243	4393	3651	
**Well 2 corr.**	5261	23795	4528	4134	3180	2328	5202	4707	4909	3047	
**Well 3 corr.**	4576	25936	4592	4175	5057	4060	4478	2932	3239	1921	
**MEAN**	4940.3	24831.0	4822.3	4480.3	3767.7	3282.3	4649.3	3960.3	4180.0	2872.7	
**SD**	344.6	1072.1	456.1	565.3	1117.7	879.5	489.6	920.6	855.1	878.0	
**REL**	**1.0**	**5.0**	**1.0**	**0.9**	**0.8**	**0.7**	**0.9**	**0.8**	**0.8**	**0.6**	
**CV**	7.0	4.3	9.5	12.6	29.7	26.8	10.5	23.2	20.5	30.6	

* 0.1 % DMSO

24 h incubation time

	ctrl.*	P	R 1	R 10	R 50	R 100	I5 1	I5 10	I5 50	I5 100	B
**RFU well 1**	18117	48436	18058	17079	16596	16100	17247	18781	17435	17415	13572
**RFU well 2**	18259	48529	18403	17048	16511	16427	16812	17859	17250	16721	13402
**RFU well 3**	19034	43795	19326	16847	16256	16061	16804	17195	16260	16877	13316
**MEAN Blank**											13430.0
**Well 1 corr.**	4687	35006	4628	3649	3166	2670	3817	5351	4005	3985	
**Well 2 corr.**	4829	35099	4973	3618	3081	2997	3382	4429	3820	3291	
**Well 3 corr.**	5604	30365	5896	3417	2826	2631	3374	3765	2830	3447	
**MEAN**	5040.0	33490.0	5165.7	3561.3	3024.3	2766.0	3524.3	4515.0	3551.7	3574.3	
**SD**	493.6	2706.7	655.6	126.0	176.9	201.0	253.5	796.5	631.8	364.1	
**REL**	**1.0**	**6.6**	**1.0**	**0.7**	**0.6**	**0.5**	**0.7**	**0.9**	**0.7**	**0.7**	
**CV**	9.8	8.1	12.7	3.5	5.9	7.3	7.2	17.6	17.8	10.2	

* 0.1 % DMSO

Experiment 4

6 h incubation time

	ctrl.*	P	R 1	R 10	R 50	R 100	I5 1	I5 10	I5 50	I5 100	B
**RFU well 1**	21106	38369	22043	22393	20379	19441	20960	19967	20176	20397	16600
**RFU well 2**	22296	39504	22539	21102	20932	19728	20935	19491	20919	19528	16328
**RFU well 3**	21368	39702	21898	21676	21278	19713	21249	21544	21700	20502	16354
**MEAN Blank**											16427.3
**Well 1 corr.**	4679	21942	5616	5966	3952	3014	4533	3540	3749	3970	
**Well 2 corr.**	5869	23077	6112	4675	4505	3301	4508	3064	4492	3101	
**Well 3 corr.**	4941	23275	5471	5249	4851	3286	4822	5117	5273	4075	
**MEAN**	5162.7	22764.3	5732.7	5296.3	4435.7	3200.0	4620.7	3906.7	4504.3	3715.0	
**SD**	625.3	719.3	336.1	646.8	453.5	161.5	174.5	1074.6	762.1	534.6	
**REL**	**1.0**	**4.4**	**1.1**	**1.0**	**0.9**	**0.6**	**0.9**	**0.8**	**0.9**	**0.7**	
**CV**	12.1	3.2	5.9	12.2	10.2	5.0	3.8	27.5	16.9	14.4	

* 0.1 % DMSO

24 h incubation time

	ctrl.*	P	R 1	R 10	R 50	R 100	I5 1	I5 10	I5 50	I5 100	B
**RFU well 1**	19168	44271	18342	17328	17114	16378	18770	17906	16219	16949	14060
**RFU well 2**	18424	45739	17935	17156	16535	16343	18410	17141	17969	17149	13787
**RFU well 3**	20090	44682	19186	18150	16850	16788	18619	17966	18217	18053	13734
**MEAN Blank**											13860.3
**Well 1 corr.**	5308	30411	4482	3468	3254	2518	4910	4046	2359	3089	
**Well 2 corr.**	4564	31879	4075	3296	2675	2483	4550	3281	4109	3289	
**Well 3 corr.**	6230	30822	5326	4290	2990	2928	4759	4106	4357	4193	
**MEAN**	5367.0	31037.0	4627.3	3684.3	2972.7	2642.7	4739.3	3810.7	3608.0	3523.3	
**SD**	834.6	757.3	638.1	531.2	289.9	247.4	180.8	460.0	1089.0	588.2	
**REL**	**1.0**	**5.8**	**0.9**	**0.7**	**0.6**	**0.5**	**0.9**	**0.7**	**0.7**	**0.7**	
**CV**	15.6	2.4	13.8	14.4	9.8	9.4	3.8	12.1	30.2	16.7	

* 0.1 % DMSO

Raw data of the cell cycle analysis

Abbreviations: CV: coefficient of variation; DMSO: dimethyl sulfoxide; noc.: nocodazole; RMS: root mean square

HCT-116^{wt} cells, 10 µM resveratrol and IRA 5

Experiment 1

24 h incubation time

	**0.1 % DMSO**	**resveratrol**	**IRA 5**	**10 µM noc.**
**Model used to fit histogram**	Watson	Watson	Watson	Watson
**Goodness of fit (RMS)**	2.78	2.44	5.01	18.60
**Events in sub G_1 [%]**	1.94	2.62	0.02	0.96
**Events in G_1 [%]**	24.60	20.30	35.70	1.65
**Events in S [%]**	51.80	44.20	38.70	14.60
**Events in G_2 [%]**	16.00	28.30	28.40	71.20
**Events in super G_2 [%]**	3.35	2.85	-2.18	-3.47
**Mean G_1 peak value**	232	235	220	234
**Mean G_2 peak value**	452	456	429	436
**G_1 peak CV**	3.91	5.77	4.63	8.93
**G_2 peak CV**	3.23	4.15	4.54	4.18

48 h incubation time

	**0.1 % DMSO**	**resveratrol**	**IRA 5**	**10 µM noc.**
**Model used to fit histogram**	Watson	Watson	Watson	Watson
**Goodness of fit (RMS)**	4.92	5.55	4.89	7.07
**Events in sub G_1 [%]**	5.46	5.13	5.42	11.50
**Events in G_1 [%]**	57.40	49.30	56.00	9.03
**Events in S [%]**	23.80	29.90	24.90	17.40
**Events in G_2 [%]**	11.10	14.40	11.90	56.20
**Events in super G_2 [%]**	1.94	1.40	1.31	0.03
**Mean G_1 peak value**	229	234	233	234
**Mean G_2 peak value**	444	451	451	457
**G_1 peak CV**	4.09	4.40	4.27	19.90
**G_2 peak CV**	4.59	3.87	4.05	5.42

Experiment 2

24 h incubation time

	0.1 % DMSO	resveratrol	IRA 5	10 μM noc.
**Model used to fit histogram**	Watson	Watson	Watson	Watson
**Goodness of fit (RMS)**	6.63	3.45	3.70	9.04
**Events in sub G_1 [%]**	5.01	1.68	2.82	2.36
**Events in G_1 [%]**	35.10	22.00	28.20	3.56
**Events in S [%]**	36.40	51.80	39.20	10.70
**Events in G_2 [%]**	21.90	26.40	29.60	77.10
**Events in super G_2 [%]**	1.33	0.03	1.63	6.56
**Mean G_1 peak value**	211	213	213	209
**Mean G_2 peak value**	412	416	416	401
**G_1 peak CV**	3.30	4.58	3.95	6.12
**G_2 peak CV**	4.01	4.41	3.55	4.92

48 h incubation time

	0.1 % DMSO	resveratrol	IRA 5	10 μM noc.
**Model used to fit histogram**	Watson	Watson	Watson	Watson
**Goodness of fit (RMS)**	4.92	4.77	3.81	9.45
**Events in sub G_1 [%]**	2.81	2.86	3.29	6.51
**Events in G_1 [%]**	58.40	53.00	56.50	3.94
**Events in S [%]**	22.70	27.20	23.00	15.80
**Events in G_2 [%]**	16.00	18.50	17.10	62.30
**Events in super G_2 [%]**	1.44	-0.98	1.41	2.74
**Mean G_1 peak value**	207	198	208	212
**Mean G_2 peak value**	402	390	404	402
**G_1 peak CV**	3.78	4.20	3.91	6.96
**G_2 peak CV**	4.21	4.34	4.19	4.32

Experiment 3

24 h incubation time

	0.1 % DMSO	resveratrol	IRA 5	10 µM noc.
**Model used to fit histogram**	Watson	Watson	Watson	Watson
**Goodness of fit (RMS)**	5.45	2.26	3.84	10.00
**Events in sub G_1 [%]**	4.50	1.73	3.32	1.42
**Events in G_1 [%]**	37.10	24.70	35.00	3.97
**Events in S [%]**	39.70	44.60	37.70	8.42
**Events in G_2 [%]**	23.20	25.70	25.20	72.90
**Events in super G_2 [%]**	0.17	2.24	-0.87	9.22
**Mean G_1 peak value**	218	215	221	220
**Mean G_2 peak value**	425	419	431	404
**G_1 peak CV**	6.59	4.57	3.78	14.00
**G_2 peak CV**	6.49	4.44	4.71	3.94

48 h incubation time

	0.1 % DMSO	resveratrol	IRA 5	10 µM noc.
**Model used to fit histogram**	Watson	Watson	Watson	Watson
**Goodness of fit (RMS)**	5.15	3.81	4.58	8.09
**Events in sub G_1 [%]**	2.11	4.97	4.75	10.00
**Events in G_1 [%]**	61.40	56.60	49.60	8.64
**Events in S [%]**	21.20	22.10	28.70	16.00
**Events in G_2 [%]**	11.60	14.10	12.10	56.50
**Events in super G_2 [%]**	2.08	2.54	2.56	2.91
**Mean G_1 peak value**	216	217	209	221
**Mean G_2 peak value**	421	421	412	421
**G_1 peak CV**	3.99	3.93	4.75	12.50
**G_2 peak CV**	4.74	4.33	4.85	4.80

Experiment 4

24 h incubation time

	**0.1 % DMSO**	**resveratrol**	**IRA 5**	**10 µM noc.**
**Model used to fit histogram**	Watson	Watson	Watson	Watson
**Goodness of fit (RMS)**	2.73	1.99	5.80	8.18
**Events in sub G_1 [%]**	2.05	2.37	3.45	3.17
**Events in G_1 [%]**	32.60	21.70	32.80	2.33
**Events in S [%]**	40.10	45.40	37.90	9.03
**Events in G_2 [%]**	23.20	28.40	27.00	83.10
**Events in super G_2 [%]**	2.06	1.37	2.45	7.04
**Mean G_1 peak value**	237	242	233	230
**Mean G_2 peak value**	463	472	453	436
**G_1 peak CV**	3.53	3.91	5.50	15.00
**G_2 peak CV**	3.70	3.94	6.69	4.49

48 h incubation time

	**0.1 % DMSO**	**resveratrol**	**IRA 5**	**10 µM noc.**
**Model used to fit histogram**	Watson	Watson	Watson	Watson
**Goodness of fit (RMS)**	3.41	3.99	2.39	6.69
**Events in sub G_1 [%]**	4.37	5.63	5.68	8.72
**Events in G_1 [%]**	64.70	62.10	69.20	7.58
**Events in S [%]**	17.80	20.90	15.60	11.00
**Events in G_2 [%]**	10.40	11.20	9.42	74.50
**Events in super G_2 [%]**	1.82	-0.05	0.59	-4.04
**Mean G_1 peak value**	224	233	236	221
**Mean G_2 peak value**	437	457	460	414
**G_1 peak CV**	4.62	3.84	5.03	14.70
**G_2 peak CV**	5.62	5.37	5.34	5.72

HCT-116^{wt} cells, 40 µM resveratrol and IRA 5

Experiment 1

24 h incubation time

	0.1 % DMSO	resveratrol	IRA 5	10 µM noc.
**Model used to fit histogram**	Watson	Watson	Watson	Watson
**Goodness of fit (RMS)**	2.32	3.19	2.77	10.20
**Events in sub G_1 [%]**	2.55	0.98	2.13	0.15
**Events in G_1 [%]**	34.70	10.20	26.20	2.55
**Events in S [%]**	41.60	79.60	29.70	10.30
**Events in G_2 [%]**	20.30	17.20	41.30	79.30
**Events in super G_2 [%]**	0.48	-4.08	2.15	6.63
**Mean G_1 peak value**	211	220	215	220
**Mean G_2 peak value**	411	429	412	400
**G_1 peak CV**	4.96	6.52	5.13	14.00
**G_2 peak CV**	5.91	12.90	6.12	4.68

48 h incubation time

	0.1 % DMSO	resveratrol	IRA 5	10 µM noc.
**Model used to fit histogram**	Watson	Watson	Watson	Watson
**Goodness of fit (RMS)**	2.80	3.82	2.53	5.02
**Events in sub G_1 [%]**	3.63	6.08	4.70	9.56
**Events in G_1 [%]**	64.90	57.20	69.80	6.30
**Events in S [%]**	15.40	17.90	13.40	15.20
**Events in G_2 [%]**	14.80	15.00	10.10	64.30
**Events in super G_2 [%]**	0.18	1.18	1.38	6.08
**Mean G_1 peak value**	215	228	224	231
**Mean G_2 peak value**	418	445	437	418
**G_1 peak CV**	6.09	5.93	6.30	13.10
**G_2 peak CV**	7.62	4.50	5.63	7.88

Experiment 2

24 h incubation time

	0.1 % DMSO	resveratrol	IRA 5	10 µM noc.
**Model used to fit histogram**	Watson	Watson	Watson	Watson
**Goodness of fit (RMS)**	2.95	3.23	2.41	6.93
**Events in sub G_1 [%]**	4.33	3.50	3.24	1.09
**Events in G_1 [%]**	27.50	25.30	17.80	2.26
**Events in S [%]**	45.80	68.20	36.20	9.65
**Events in G_2 [%]**	23.00	6.81	43.00	86.70
**Events in super G_2 [%]**	2.20	-0.46	1.94	3.67
**Mean G_1 peak value**	195	212	196	194
**Mean G_2 peak value**	381	414	381	361
**G_1 peak CV**	7.69	15.20	7.88	10.20
**G_2 peak CV**	7.66	9.79	7.59	6.88

48 h incubation time

	0.1 % DMSO	resveratrol	IRA 5	10 µM noc.
**Model used to fit histogram**	Watson	Watson	Watson	Watson
**Goodness of fit (RMS)**	8.09	9.62	8.94	5.63
**Events in sub G_1 [%]**	7.39	11.40	8.62	8.73
**Events in G_1 [%]**	61.20	68.20	76.60	11.30
**Events in S [%]**	26.50	19.00	15.20	21.20
**Events in G_2 [%]**	7.78	11.90	10.20	60.30
**Events in super G_2 [%]**	1.84	-0.44	-0.38	4.73
**Mean G_1 peak value**	198	215	217	187
**Mean G_2 peak value**	397	418	424	399
**G_1 peak CV**	10.50	9.70	12.10	29.10
**G_2 peak CV**	7.79	6.81	9.53	7.57

Experiment 3

24 h incubation time

	0.1 % DMSO	resveratrol	IRA 5	10 µM noc.
**Model used to fit histogram**	Watson	Watson	Watson	Watson
**Goodness of fit (RMS)**	5.33	4.66	5.11	7.86
**Events in sub G_1 [%]**	-1.10	0.85	1.70	1.22
**Events in G_1 [%]**	32.60	14.70	18.00	2.82
**Events in S [%]**	45.90	78.80	37.00	11.10
**Events in G_2 [%]**	24.90	12.70	46.30	79.40
**Events in super G_2 [%]**	-0.79	-2.10	1.50	2.05
**Mean G_1 peak value**	210	226	215	210
**Mean G_2 peak value**	409	441	413	395
**G_1 peak CV**	4.75	6.53	4.84	8.25
**G_2 peak CV**	5.09	9.13	5.95	5.18

48 h incubation time

	0.1 % DMSO	resveratrol	IRA 5	10 µM noc.
**Model used to fit histogram**	Watson	Watson	Watson	Watson
**Goodness of fit (RMS)**	2.61	3.35	3.24	5.17
**Events in sub G_1 [%]**	2.17	4.57	3.35	8.03
**Events in G_1 [%]**	51.40	50.00	61.60	3.93
**Events in S [%]**	28.20	27.30	19.20	18.20
**Events in G_2 [%]**	17.00	17.80	13.10	63.20
**Events in super G_2 [%]**	1.08	-0.57	1.40	6.38
**Mean G_1 peak value**	211	221	212	218
**Mean G_2 peak value**	411	431	414	411
**G_1 peak CV**	4.23	5.10	4.36	5.92
**G_2 peak CV**	4.90	5.30	4.50	5.06

Experiment 4

24 h incubation time

	0.1 % DMSO	resveratrol	IRA 5	10 µM noc.
**Model used to fit histogram**	Watson	Watson	Watson	Watson
**Goodness of fit (RMS)**	2.76	3.70	8.53	7.40
**Events in sub G_1 [%]**	1.23	0.62	1.77	1.36
**Events in G_1 [%]**	35.40	11.00	29.10	2.84
**Events in S [%]**	46.70	83.90	28.00	8.01
**Events in G_2 [%]**	17.20	9.29	51.50	80.10
**Events in super G_2 [%]**	0.64	-2.08	-8.25	6.20
**Mean G_1 peak value**	189	198	199	198
**Mean G_2 peak value**	369	397	387	369
**G_1 peak CV**	4.80	4.82	4.89	8.61
**G_2 peak CV**	4.89	6.32	5.93	5.05

48 h incubation time

	0.1 % DMSO	resveratrol	IRA 5	10 µM noc.
**Model used to fit histogram**	Watson	Watson	Watson	Watson
**Goodness of fit (RMS)**	5.25	5.68	3.90	7.46
**Events in sub G_1 [%]**	3.01	4.00	3.14	6.52
**Events in G_1 [%]**	66.60	67.30	67.50	3.22
**Events in S [%]**	14.30	14.70	14.30	13.20
**Events in G_2 [%]**	23.40	12.90	18.40	72.40
**Events in super G_2 [%]**	-4.05	1.41	-1.86	7.52
**Mean G_1 peak value**	198	206	197	201
**Mean G_2 peak value**	386	401	384	381
**G_1 peak CV**	4.20	4.88	5.24	4.76
**G_2 peak CV**	8.46	3.42	7.85	5.99

HCT-116^{wt} cells, 80 µM resveratrol and IRA 5

Experiment 1

24 h incubation time

	0.1 % DMSO	resveratrol	IRA 5	10 µM noc.
**Model used to fit histogram**	Watson	Watson	Watson	Watson
**Goodness of fit (RMS)**	2.57	1.93	5.72	7.46
**Events in sub G_1 [%]**	1.43	1.97	3.90	1.26
**Events in G_1 [%]**	28.40	66.60	14.60	3.10
**Events in S [%]**	48.50	25.00	69.00	8.74
**Events in G_2 [%]**	18.20	7.10	21.00	81.70
**Events in super G_2 [%]**	1.65	-1.13	-0.82	5.76
**Mean G_1 peak value**	213	232	226	220
**Mean G_2 peak value**	415	453	416	407
**G_1 peak CV**	4.37	8.39	6.40	8.46
**G_2 peak CV**	4.18	8.58	7.80	4.71

48 h incubation time

	0.1 % DMSO	resveratrol	IRA 5	10 µM noc.
**Model used to fit histogram**	Watson	Watson	Watson	Watson
**Goodness of fit (RMS)**	2.57	6.40	3.79	8.18
**Events in sub G_1 [%]**	2.42	6.95	5.57	8.47
**Events in G_1 [%]**	56.80	10.00	58.10	6.59
**Events in S [%]**	26.10	62.80	15.90	22.00
**Events in G_2 [%]**	15.30	31.60	19.10	60.40
**Events in super G_2 [%]**	-0.53	-6.52	1.72	-4.52
**Mean G_1 peak value**	210	217	219	215
**Mean G_2 peak value**	409	424	427	420
**G_1 peak CV**	4.67	6.77	4.76	11.70
**G_2 peak CV**	5.24	10.20	4.99	5.32

Experiment 2

24 h incubation time

	0.1 % DMSO	resveratrol	IRA 5	10 µM noc.
**Model used to fit histogram**	Watson	Watson	Watson	Watson
**Goodness of fit (RMS)**	5.29	2.11	5.98	8.35
**Events in sub G_1 [%]**	3.96	1.62	1.40	1.17
**Events in G_1 [%]**	29.90	66.80	18.30	2.94
**Events in S [%]**	41.80	23.50	68.80	8.91
**Events in G_2 [%]**	20.50	6.35	19.20	93.60
**Events in super G_2 [%]**	2.23	-0.25	0.19	-3.44
**Mean G_1 peak value**	190	214	205	201
**Mean G_2 peak value**	370	417	383	372
**G_1 peak CV**	3.75	7.67	6.42	7.66
**G_2 peak CV**	3.45	5.21	7.74	5.35

48 h incubation time

	0.1 % DMSO	resveratrol	IRA 5	10 µM noc.
**Model used to fit histogram**	Watson	Watson	Watson	Watson
**Goodness of fit (RMS)**	6.41	3.77	3.46	4.15
**Events in sub G_1 [%]**	3.88	5.81	4.73	7.72
**Events in G_1 [%]**	61.60	14.80	64.60	5.99
**Events in S [%]**	19.50	66.90	14.50	15.20
**Events in G_2 [%]**	16.70	14.70	15.80	68.10
**Events in super G_2 [%]**	0.06	0.56	0.41	0.93
**Mean G_1 peak value**	182	194	203	201
**Mean G_2 peak value**	354	379	395	380
**G_1 peak CV**	5.03	5.10	4.92	8.89
**G_2 peak CV**	5.21	5.90	5.19	5.23

Experiment 3

24 h incubation time

	0.1 % DMSO	resveratrol	IRA 5	10 µM noc.
**Model used to fit histogram**	Watson	Watson	Watson	Watson
**Goodness of fit (RMS)**	3.11	3.52	3.44	9.26
**Events in sub G_1 [%]**	1.29	0.40	1.94	0.95
**Events in G_1 [%]**	29.10	71.60	12.40	1.45
**Events in S [%]**	52.40	23.20	65.70	7.44
**Events in G_2 [%]**	16.30	3.22	19.50	83.20
**Events in super G_2 [%]**	2.12	0.55	-1.56	1.12
**Mean G_1 peak value**	188	216	201	201
**Mean G_2 peak value**	366	421	401	372
**G_1 peak CV**	5.04	11.80	4.81	6.93
**G_2 peak CV**	5.10	7.20	3.52	5.03

48 h incubation time

	0.1 % DMSO	resveratrol	IRA 5	10 µM noc.
**Model used to fit histogram**	Watson	Watson	Watson	Watson
**Goodness of fit (RMS)**	3.06	6.41	2.91	7.51
**Events in sub G_1 [%]**	2.71	8.58	4.55	7.03
**Events in G_1 [%]**	67.30	18.00	68.00	6.25
**Events in S [%]**	14.80	71.20	11.80	18.80
**Events in G_2 [%]**	12.50	12.10	13.70	56.40
**Events in super G_2 [%]**	1.90	-2.44	1.06	6.40
**Mean G_1 peak value**	196	206	204	205
**Mean G_2 peak value**	382	402	401	389
**G_1 peak CV**	4.67	6.46	5.07	10.50
**G_2 peak CV**	5.07	6.54	5.03	4.22

Experiment 4

24 h incubation time

	0.1 % DMSO	resveratrol	IRA 5	10 µM noc.
**Model used to fit histogram**	Watson	Watson	Watson	Watson
**Goodness of fit (RMS)**	2.19	3.23	3.10	7.83
**Events in sub G_1 [%]**	1.11	1.41	1.24	1.10
**Events in G_1 [%]**	24.50	71.40	12.60	3.05
**Events in S [%]**	52.70	26.00	59.20	12.40
**Events in G_2 [%]**	21.80	3.72	27.00	76.20
**Events in super G_2 [%]**	-0.29	-1.06	-0.85	6.74
**Mean G_1 peak value**	225	266	229	219
**Mean G_2 peak value**	439	518	451	395
**G_1 peak CV**	4.84	13.60	5.82	20.50
**G_2 peak CV**	4.99	10.60	3.79	8.08

48 h incubation time

	0.1 % DMSO	resveratrol	IRA 5	10 µM noc.
**Model used to fit histogram**	Watson	Watson	Watson	Watson
**Goodness of fit (RMS)**	4.27	3.19	3.00	7.38
**Events in sub G_1 [%]**	1.73	4.41	2.95	7.38
**Events in G_1 [%]**	66.00	16.10	68.00	5.50
**Events in S [%]**	18.20	64.90	13.60	24.20
**Events in G_2 [%]**	10.80	16.50	13.90	54.50
**Events in super G_2 [%]**	1.26	0.07	1.66	-0.06
**Mean G_1 peak value**	216	225	227	230
**Mean G_2 peak value**	422	438	444	428
**G_1 peak CV**	5.02	5.56	6.26	11.70
**G_2 peak CV**	5.21	6.90	4.86	6.10

A-431 cells, 10 µM resveratrol and IRA 5

Experiment 1

24 h incubation time

	0.1 % DMSO	resveratrol	IRA 5	10 µM noc.
**Model used to fit histogram**	Watson	Watson	Watson	Watson
**Goodness of fit (RMS)**	2.81	5.52	7.73	12.60
**Events in sub G_1 [%]**	2.56	3.15	1.00	21.40
**Events in G_1 [%]**	55.10	65.60	41.90	7.39
**Events in S [%]**	30.90	25.30	42.30	20.90
**Events in G_2 [%]**	10.30	8.76	22.20	45.80
**Events in super G_2 [%]**	0.79	-0.59	-0.79	0.67
**Mean G_1 peak value**	324	312	316	308
**Mean G_2 peak value**	629	612	600	585
**G_1 peak CV**	7.86	7.40	8.52	7.75
**G_2 peak CV**	6.68	6.53	9.36	6.97

48 h incubation time

	0.1 % DMSO	resveratrol	IRA 5	10 µM noc.
**Model used to fit histogram**	Watson	Watson	Watson	Watson
**Goodness of fit (RMS)**	4.66	7.03	4.41	15.60
**Events in sub G_1 [%]**	5.30	3.38	3.12	33.80
**Events in G_1 [%]**	51.50	60.10	47.80	5.84
**Events in S [%]**	31.80	31.30	39.70	26.00
**Events in G_2 [%]**	11.40	7.19	11.00	32.30
**Events in super G_2 [%]**	0.15	-0.28	0.65	2.18
**Mean G_1 peak value**	301	301	308	301
**Mean G_2 peak value**	581	581	581	557
**G_1 peak CV**	7.11	7.07	8.75	7.79
**G_2 peak CV**	6.74	6.30	8.79	8.27

Experiment 2

24 h incubation time

	0.1 % DMSO	resveratrol	IRA 5	10 µM noc.
**Model used to fit histogram**	Watson	Watson	Watson	Watson
**Goodness of fit (RMS)**	4.58	5.27	7.38	11.00
**Events in sub G_1 [%]**	3.25	3.94	5.25	15.90
**Events in G_1 [%]**	54.00	61.50	49.00	5.44
**Events in S [%]**	30.60	24.30	27.30	26.00
**Events in G_2 [%]**	14.10	10.80	12.90	43.70
**Events in super G_2 [%]**	0.79	1.35	0.73	4.64
**Mean G_1 peak value**	352	342	336	359
**Mean G_2 peak value**	675	657	648	684
**G_1 peak CV**	6.99	7.82	6.72	10.60
**G_2 peak CV**	6.85	6.79	7.66	6.82

48 h incubation time

	0.1 % DMSO	resveratrol	IRA 5	10 µM noc.
**Model used to fit histogram**	Watson	Watson	Watson	Watson
**Goodness of fit (RMS)**	3.84	6.23	2.35	13.50
**Events in sub G_1 [%]**	3.93	6.55	3.91	26.30
**Events in G_1 [%]**	50.30	51.40	46.40	4.86
**Events in S [%]**	32.40	36.60	33.90	17.10
**Events in G_2 [%]**	17.70	8.87	15.70	47.50
**Events in super G_2 [%]**	-1.90	1.74	-0.01	2.82
**Mean G_1 peak value**	341	338	342	342
**Mean G_2 peak value**	659	653	652	627
**G_1 peak CV**	7.99	7.61	7.46	7.98
**G_2 peak CV**	9.77	4.19	8.86	8.20

Experiment 3

24 h incubation time

	0.1 % DMSO	resveratrol	IRA 5	10 µM noc.
**Model used to fit histogram**	Watson	Watson	Watson	Watson
**Goodness of fit (RMS)**	3.38	4.26	2.79	12.00
**Events in sub G_1 [%]**	4.36	5.44	4.41	25.00
**Events in G_1 [%]**	48.60	56.00	49.20	7.88
**Events in S [%]**	33.30	34.10	32.70	24.60
**Events in G_2 [%]**	14.90	7.95	13.90	45.60
**Events in super G_2 [%]**	-0.36	0.27	-0.29	-1.25
**Mean G_1 peak value**	368	383	379	397
**Mean G_2 peak value**	700	739	725	713
**G_1 peak CV**	6.49	8.45	6.26	11.40
**G_2 peak CV**	7.61	4.46	6.77	8.24

48 h incubation time

	0.1 % DMSO	resveratrol	IRA 5	10 µM noc.
**Model used to fit histogram**	Watson	Watson	Watson	Watson
**Goodness of fit (RMS)**	6.27	5.92	6.03	14.10
**Events in sub G_1 [%]**	10.50	9.53	5.43	27.60
**Events in G_1 [%]**	48.40	51.40	53.00	2.88
**Events in S [%]**	33.70	33.40	34.50	25.90
**Events in G_2 [%]**	12.80	9.50	10.80	37.20
**Events in super G_2 [%]**	0.22	-0.16	0.24	-0.34
**Mean G_1 peak value**	377	370	377	367
**Mean G_2 peak value**	724	709	724	700
**G_1 peak CV**	6.96	6.30	6.18	6.35
**G_2 peak CV**	5.69	5.86	5.73	7.27

Experiment 4

24 h incubation time

	0.1 % DMSO	resveratrol	IRA 5	10 µM noc.
**Model used to fit histogram**	Watson	Watson	Watson	Watson
**Goodness of fit (RMS)**	2.29	2.06	7.69	15.50
**Events in sub G_1 [%]**	3.47	3.45	0.68	27.00
**Events in G_1 [%]**	55.00	54.20	48.50	7.65
**Events in S [%]**	32.90	35.70	34.20	19.80
**Events in G_2 [%]**	8.82	6.20	11.10	41.60
**Events in super G_2 [%]**	0.37	0.13	-0.07	-0.02
**Mean G_1 peak value**	367	365	365	358
**Mean G_2 peak value**	707	715	706	674
**G_1 peak CV**	6.36	7.08	6.15	10.00
**G_2 peak CV**	6.87	6.90	8.07	7.59

48 h incubation time

	0.1 % DMSO	resveratrol	IRA 5	10 µM noc.
**Model used to fit histogram**	Watson	Watson	Watson	Watson
**Goodness of fit (RMS)**	4.46	6.71	7.09	19.20
**Events in sub G_1 [%]**	8.18	9.19	10.30	37.10
**Events in G_1 [%]**	51.00	56.80	51.40	4.93
**Events in S [%]**	31.00	28.60	31.70	23.50
**Events in G_2 [%]**	11.60	11.10	12.80	33.10
**Events in super G_2 [%]**	0.14	-0.57	-1.05	0.12
**Mean G_1 peak value**	349	358	359	349
**Mean G_2 peak value**	671	687	690	671
**G_1 peak CV**	7.36	7.19	6.39	7.36
**G_2 peak CV**	6.77	6.74	6.49	8.06

A-431 cells, 40 µM resveratrol and IRA 5

Experiment 1

24 h incubation time

	0.1 % DMSO	resveratrol	IRA 5	10 µM noc.
**Model used to fit histogram**	Watson	Watson	Watson	Watson
**Goodness of fit (RMS)**	4.10	5.45	17.90	12.10
**Events in sub G_1 [%]**	2.13	6.73	0.67	22.90
**Events in G_1 [%]**	57.50	37.30	35.50	5.57
**Events in S [%]**	30.90	29.90	45.10	21.60
**Events in G_2 [%]**	12.30	24.80	6.23	45.90
**Events in super G_2 [%]**	-0.83	0.80	0.92	0.12
**Mean G_1 peak value**	307	304	301	333
**Mean G_2 peak value**	596	589	613	599
**G_1 peak CV**	6.40	8.19	4.87	4.70
**G_2 peak CV**	7.25	7.08	3.59	6.51

48 h incubation time

	0.1 % DMSO	resveratrol	IRA 5	10 µM noc.
**Model used to fit histogram**	Watson	Watson	Watson	Watson
**Goodness of fit (RMS)**	5.88	9.44	3.33	14.90
**Events in sub G_1 [%]**	8.54	18.00	3.50	28.40
**Events in G_1 [%]**	52.20	45.10	48.80	7.91
**Events in S [%]**	32.20	28.20	33.00	19.40
**Events in G_2 [%]**	12.20	12.00	15.40	41.60
**Events in super G_2 [%]**	-0.04	0.46	0.16	1.73
**Mean G_1 peak value**	320	301	306	304
**Mean G_2 peak value**	615	577	588	572
**G_1 peak CV**	9.56	7.71	6.36	10.10
**G_2 peak CV**	7.70	7.31	7.39	7.01

Experiment 2

24 h incubation time

	0.1 % DMSO	resveratrol	IRA 5	10 µM noc.
**Model used to fit histogram**	Watson	Watson	Watson	Watson
**Goodness of fit (RMS)**	5.22	5.21	5.92	11.80
**Events in sub G_1 [%]**	1.02	8.72	3.40	20.30
**Events in G_1 [%]**	57.60	48.80	38.50	4.19
**Events in S [%]**	30.70	27.80	54.20	24.70
**Events in G_2 [%]**	15.20	15.70	10.10	44.90
**Events in super G_2 [%]**	-0.48	0.03	-0.02	-1.96
**Mean G_1 peak value**	346	340	354	354
**Mean G_2 peak value**	668	656	682	643
**G_1 peak CV**	7.95	7.80	9.00	5.05
**G_2 peak CV**	7.08	6.88	5.63	6.62

48 h incubation time

	0.1 % DMSO	resveratrol	IRA 5	10 µM noc.
**Model used to fit histogram**	Watson	Watson	Watson	Watson
**Goodness of fit (RMS)**	7.09	6.75	5.45	13.60
**Events in sub G_1 [%]**	4.74	10.40	9.31	27.00
**Events in G_1 [%]**	41.70	43.00	52.30	6.65
**Events in S [%]**	34.10	37.60	33.40	16.60
**Events in G_2 [%]**	21.10	10.40	9.20	48.10
**Events in super G_2 [%]**	-0.25	-0.53	-0.82	-0.39
**Mean G_1 peak value**	324	336	337	344
**Mean G_2 peak value**	616	644	647	638
**G_1 peak CV**	8.92	6.70	8.08	10.70
**G_2 peak CV**	8.66	6.55	7.93	6.44

Experiment 3

24 h incubation time

	0.1 % DMSO	resveratrol	IRA 5	10 µM noc.
**Model used to fit histogram**	Watson	Watson	Watson	Watson
**Goodness of fit (RMS)**	4.89	5.80	3.34	20.00
**Events in sub G_1 [%]**	2.10	7.92	2.51	32.30
**Events in G_1 [%]**	52.40	35.90	35.00	12.50
**Events in S [%]**	37.50	33.80	47.00	25.60
**Events in G_2 [%]**	14.00	24.70	19.20	33.60
**Events in super G_2 [%]**	-2.19	-0.42	-1.53	0.25
**Mean G_1 peak value**	319	318	322	315
**Mean G_2 peak value**	615	605	621	593
**G_1 peak CV**	8.55	8.93	10.30	24.30
**G_2 peak CV**	9.19	7.72	9.46	8.49

48 h incubation time

	0.1 % DMSO	resveratrol	IRA 5	10 µM noc.
**Model used to fit histogram**	Watson	Watson	Watson	Watson
**Goodness of fit (RMS)**	4.49	6.25	5.67	20.70
**Events in sub G_1 [%]**	5.85	11.00	7.96	37.20
**Events in G_1 [%]**	48.60	40.80	50.70	5.13
**Events in S [%]**	36.90	36.90	31.40	20.60
**Events in G_2 [%]**	12.70	17.10	11.10	34.30
**Events in super G_2 [%]**	-1.40	-3.23	-0.16	0.03
**Mean G_1 peak value**	312	312	321	312
**Mean G_2 peak value**	602	602	602	602
**G_1 peak CV**	7.43	8.32	8.13	7.43
**G_2 peak CV**	7.89	10.40	9.84	8.39

Experiment 4

24 h incubation time

	0.1 % DMSO	resveratrol	IRA 5	10 µM noc.
**Model used to fit histogram**	Watson	Watson	Watson	Watson
**Goodness of fit (RMS)**	2.26	6.10	7.30	18.60
**Events in sub G_1 [%]**	3.83	10.80	3.62	31.00
**Events in G_1 [%]**	50.80	41.40	32.20	10.50
**Events in S [%]**	35.50	36.60	59.00	31.40
**Events in G_2 [%]**	8.87	15.50	15.70	34.80
**Events in super G_2 [%]**	0.45	-1.64	-1.53	-2.35
**Mean G_1 peak value**	357	365	377	333
**Mean G_2 peak value**	690	704	695	660
**G_1 peak CV**	5.92	6.42	9.63	27.30
**G_2 peak CV**	6.45	6.72	7.72	10.30

48 h incubation time

	0.1 % DMSO	resveratrol	IRA 5	10 µM noc.
**Model used to fit histogram**	Watson	Watson	Watson	Watson
**Goodness of fit (RMS)**	3.03	8.94	5.51	18.00
**Events in sub G_1 [%]**	5.63	14.40	9.92	34.40
**Events in G_1 [%]**	49.40	49.40	56.00	6.17
**Events in S [%]**	33.70	29.90	31.30	23.70
**Events in G_2 [%]**	11.20	9.95	6.31	34.70
**Events in super G_2 [%]**	-0.41	-0.79	-0.20	1.31
**Mean G_1 peak value**	337	359	362	345
**Mean G_2 peak value**	650	693	698	666
**G_1 peak CV**	8.32	9.22	7.40	13.00
**G_2 peak CV**	8.89	7.08	5.02	7.22

A-431 cells, 80 µM resveratrol and IRA 5

Experiment 1

24 h incubation time

	**0.1 % DMSO**	**resveratrol**	**IRA 5**	**10 µM noc.**
**Model used to fit histogram**	Watson	Watson	Watson	Watson
**Goodness of fit (RMS)**	3.01	9.45	4.00	10.50
**Events in sub G_1 [%]**	2.41	13.80	1.81	20.60
**Events in G_1 [%]**	52.60	37.80	13.00	6.01
**Events in S [%]**	33.50	42.20	74.10	16.10
**Events in G_2 [%]**	12.60	8.79	11.70	56.60
**Events in super G_2 [%]**	0.56	-0.04	1.75	0.70
**Mean G_1 peak value**	337	338	330	351
**Mean G_2 peak value**	647	653	637	618
**G_1 peak CV**	7.25	9.78	7.29	8.86
**G_2 peak CV**	6.88	6.89	8.17	8.60

48 h incubation time

	**0.1 % DMSO**	**resveratrol**	**IRA 5**	**10 µM noc.**
**Model used to fit histogram**	Watson	Watson	Watson	Watson
**Goodness of fit (RMS)**	3.38	10.50	6.28	12.20
**Events in sub G_1 [%]**	3.83	19.90	5.78	23.60
**Events in G_1 [%]**	49.70	24.60	29.60	5.03
**Events in S [%]**	33.40	47.90	56.60	22.90
**Events in G_2 [%]**	15.90	13.10	16.60	54.20
**Events in super G_2 [%]**	-0.94	0.00	-1.48	0.37
**Mean G_1 peak value**	333	342	355	345
**Mean G_2 peak value**	639	631	616	607
**G_1 peak CV**	7.86	8.89	14.80	9.97
**G_2 peak CV**	9.03	9.25	12.60	9.58

Experiment 2

24 h incubation time

	0.1 % DMSO	resveratrol	IRA 5	10 µM noc.
**Model used to fit histogram**	Watson	Watson	Watson	Watson
**Goodness of fit (RMS)**	2.97	9.80	3.68	12.30
**Events in sub G_1 [%]**	3.58	14.10	3.05	23.90
**Events in G_1 [%]**	52.30	31.40	16.00	5.52
**Events in S [%]**	35.50	45.00	70.90	16.90
**Events in G_2 [%]**	10.80	8.28	12.70	46.40
**Events in super G_2 [%]**	-0.52	1.78	-0.94	2.74
**Mean G_1 peak value**	359	380	365	384
**Mean G_2 peak value**	689	735	700	715
**G_1 peak CV**	7.24	11.20	6.92	11.00
**G_2 peak CV**	7.74	5.68	11.30	6.88

48 h incubation time

	0.1 % DMSO	resveratrol	IRA 5	10 µM noc.
**Model used to fit histogram**	Watson	Watson	Watson	Watson
**Goodness of fit (RMS)**	10.30	13.80	6.01	14.10
**Events in sub G_1 [%]**	16.40	26.70	11.10	27.10
**Events in G_1 [%]**	51.30	26.90	17.20	3.77
**Events in S [%]**	24.40	32.50	58.90	15.20
**Events in G_2 [%]**	13.50	14.00	19.20	52.10
**Events in super G_2 [%]**	-0.49	-0.88	-3.01	-2.26
**Mean G_1 peak value**	374	363	374	374
**Mean G_2 peak value**	719	708	719	696
**G_1 peak CV**	9.05	10.70	8.26	8.26
**G_2 peak CV**	8.81	7.21	8.61	6.80

Experiment 3

24 h incubation time

	0.1 % DMSO	resveratrol	IRA 5	10 µM noc.
**Model used to fit histogram**	Watson	Watson	Watson	Watson
**Goodness of fit (RMS)**	4.58	7.35	3.24	16.30
**Events in sub G_1 [%]**	3.85	8.68	3.43	29.70
**Events in G_1 [%]**	55.10	32.50	9.94	5.22
**Events in S [%]**	32.70	56.20	75.80	18.20
**Events in G_2 [%]**	12.00	7.86	13.40	51.00
**Events in super G_2 [%]**	-0.84	-0.20	-0.23	-0.95
**Mean G_1 peak value**	352	363	352	348
**Mean G_2 peak value**	679	685	680	639
**G_1 peak CV**	7.86	8.32	6.38	11.60
**G_2 peak CV**	7.65	8.39	10.40	8.70

48 h incubation time

	0.1 % DMSO	resveratrol	IRA 5	10 µM noc.
**Model used to fit histogram**	Watson	Watson	Watson	Watson
**Goodness of fit (RMS)**	5.67	7.95	5.39	16.20
**Events in sub G_1 [%]**	5.76	17.80	12.10	34.20
**Events in G_1 [%]**	56.90	33.80	24.90	3.00
**Events in S [%]**	26.70	40.00	56.00	17.60
**Events in G_2 [%]**	15.40	10.40	10.80	47.30
**Events in super G_2 [%]**	-1.84	-0.17	-0.97	-1.42
**Mean G_1 peak value**	333	351	358	348
**Mean G_2 peak value**	642	677	690	654
**G_1 peak CV**	6.82	6.46	9.05	6.52
**G_2 peak CV**	8.67	5.46	5.34	7.74

Experiment 4

24 h incubation time

	0.1 % DMSO	resveratrol	IRA 5	10 µM noc.
**Model used to fit histogram**	Watson	Watson	Watson	Watson
**Goodness of fit (RMS)**	3.68	7.49	4.18	16.10
**Events in sub G_1 [%]**	3.53	10.60	8.33	28.00
**Events in G_1 [%]**	53.00	34.60	12.60	4.23
**Events in S [%]**	35.80	51.00	72.70	15.20
**Events in G_2 [%]**	9.77	9.10	8.22	52.30
**Events in super G_2 [%]**	-0.57	0.32	0.21	0.29
**Mean G_1 peak value**	343	354	351	329
**Mean G_2 peak value**	666	663	677	623
**G_1 peak CV**	6.30	8.22	7.23	9.80
**G_2 peak CV**	5.99	6.25	6.68	9.61

48 h incubation time

	0.1 % DMSO	resveratrol	IRA 5	10 µM noc.
**Model used to fit histogram**	Watson	Watson	Watson	Watson
**Goodness of fit (RMS)**	4.87	7.29	4.77	16.80
**Events in sub G_1 [%]**	8.70	16.40	8.13	33.30
**Events in G_1 [%]**	50.10	34.80	25.20	4.34
**Events in S [%]**	30.30	35.30	62.50	20.40
**Events in G_2 [%]**	10.80	13.90	8.82	42.50
**Events in super G_2 [%]**	-0.29	0.38	-0.66	-1.02
**Mean G_1 peak value**	338	345	351	326
**Mean G_2 peak value**	653	653	677	629
**G_1 peak CV**	6.46	7.19	8.75	6.71
**G_2 peak CV**	7.01	7.75	5.53	8.89

Caco-2 cells, 10 µM resveratrol and IRA 5

Experiment 1

24 h incubation time

	0.1 % DMSO	resveratrol	IRA 5	10 µM noc.
**Model used to fit histogram**	Watson	Watson	Watson	Watson
**Goodness of fit (RMS)**	1.93	2.74	1.79	4.89
**Events in sub G_1 [%]**	7.90	14.20	4.93	7.22
**Events in G_1 [%]**	29.00	32.70	31.00	3.17
**Events in S [%]**	40.90	46.70	45.60	26.30
**Events in G_2 [%]**	22.50	14.10	16.90	54.80
**Events in super G_2 [%]**	0.42	-1.70	1.84	0.97
**Mean G_1 peak value**	391	405	370	373
**Mean G_2 peak value**	759	785	722	729
**G_1 peak CV**	6.57	11.10	6.90	7.81
**G_2 peak CV**	7.16	7.76	7.03	5.98

48 h incubation time

	0.1 % DMSO	resveratrol	IRA 5	10 µM noc.
**Model used to fit histogram**	Watson	Watson	Watson	Watson
**Goodness of fit (RMS)**	2.71	3.33	2.81	4.56
**Events in sub G_1 [%]**	9.77	7.57	9.06	17.30
**Events in G_1 [%]**	39.50	35.90	39.80	6.35
**Events in S [%]**	35.60	40.00	36.90	22.60
**Events in G_2 [%]**	17.30	20.70	15.40	50.90
**Events in super G_2 [%]**	0.40	-0.74	0.80	5.07
**Mean G_1 peak value**	377	324	374	380
**Mean G_2 peak value**	728	626	721	733
**G_1 peak CV**	7.18	7.25	6.39	11.40
**G_2 peak CV**	7.87	9.65	7.58	6.19

Experiment 2

24 h incubation time

	0.1 % DMSO	resveratrol	IRA 5	10 µM noc.
**Model used to fit histogram**	Watson	Watson	Watson	Watson
**Goodness of fit (RMS)**	2.21	2.93	2.21	5.24
**Events in sub G_1 [%]**	10.40	10.20	9.20	11.60
**Events in G_1 [%]**	28.90	29.90	23.70	2.48
**Events in S [%]**	43.10	45.60	47.00	21.30
**Events in G_2 [%]**	18.10	17.70	21.40	54.50
**Events in super G_2 [%]**	0.68	0.10	0.65	2.51
**Mean G_1 peak value**	427	427	427	416
**Mean G_2 peak value**	824	824	824	770
**G_1 peak CV**	6.15	6.12	6.01	7.70
**G_2 peak CV**	6.35	6.79	8.22	7.47

48 h incubation time

	0.1 % DMSO	resveratrol	IRA 5	10 µM noc.
**Model used to fit histogram**	Watson	Watson	Watson	Watson
**Goodness of fit (RMS)**	2.38	3.39	2.55	3.42
**Events in sub G_1 [%]**	9.56	15.40	17.80	23.10
**Events in G_1 [%]**	30.80	28.00	36.10	4.20
**Events in S [%]**	41.40	45.40	38.60	23.40
**Events in G_2 [%]**	21.70	16.10	12.10	53.30
**Events in super G_2 [%]**	0.63	-1.27	-1.25	-0.10
**Mean G_1 peak value**	406	436	439	415
**Mean G_2 peak value**	784	842	848	800
**G_1 peak CV**	7.63	15.70	12.30	6.77
**G_2 peak CV**	8.80	11.30	8.53	10.70

Experiment 3

24 h incubation time

	0.1 % DMSO	resveratrol	IRA 5	10 µM noc.
**Model used to fit histogram**	Watson	Watson	Watson	Watson
**Goodness of fit (RMS)**	2.65	1.89	1.83	3.52
**Events in sub G_1 [%]**	6.19	10.60	5.87	9.90
**Events in G_1 [%]**	37.40	31.50	26.80	3.28
**Events in S [%]**	41.90	45.80	45.20	25.60
**Events in G_2 [%]**	18.50	16.60	30.60	49.20
**Events in super G_2 [%]**	0.31	-0.42	-0.86	0.21
**Mean G_1 peak value**	422	428	422	425
**Mean G_2 peak value**	809	825	809	819
**G_1 peak CV**	10.20	10.00	10.20	10.10
**G_2 peak CV**	8.45	9.29	11.00	10.20

48 h incubation time

	0.1 % DMSO	resveratrol	IRA 5	10 µM noc.
**Model used to fit histogram**	Watson	Watson	Watson	Watson
**Goodness of fit (RMS)**	2.72	1.73	2.47	2.51
**Events in sub G_1 [%]**	12.20	12.20	9.45	21.60
**Events in G_1 [%]**	32.60	34.90	38.40	4.22
**Events in S [%]**	37.50	40.50	39.30	21.00
**Events in G_2 [%]**	23.70	22.00	18.20	53.60
**Events in super G_2 [%]**	-0.25	-1.49	1.45	-0.34
**Mean G_1 peak value**	417	419	409	413
**Mean G_2 peak value**	805	805	789	797
**G_1 peak CV**	8.02	18.60	11.20	8.10
**G_2 peak CV**	11.00	13.20	10.20	10.70

Experiment 4

24 h incubation time

	**0.1 % DMSO**	**resveratrol**	**IRA 5**	**10 µM noc.**
**Model used to fit histogram**	Watson	Watson	Watson	Watson
**Goodness of fit (RMS)**	3.85	4.32	4.59	4.50
**Events in sub G_1 [%]**	12.70	18.40	13.10	17.60
**Events in G_1 [%]**	30.40	24.90	33.20	5.18
**Events in S [%]**	43.30	47.50	42.30	26.50
**Events in G_2 [%]**	19.10	14.70	16.20	46.40
**Events in super G_2 [%]**	0.23	-0.37	0.72	2.20
**Mean G_1 peak value**	406	406	399	388
**Mean G_2 peak value**	784	784	771	749
**G_1 peak CV**	9.69	11.60	11.90	8.62
**G_2 peak CV**	6.45	7.69	7.79	8.03

48 h incubation time

	**0.1 % DMSO**	**resveratrol**	**IRA 5**	**10 µM noc.**
**Model used to fit histogram**	Watson	Watson	Watson	Watson
**Goodness of fit (RMS)**	3.04	3.90	3.29	5.37
**Events in sub G_1 [%]**	11.10	13.90	11.60	25.10
**Events in G_1 [%]**	29.30	35.10	34.00	5.10
**Events in S [%]**	45.10	39.20	40.10	22.80
**Events in G_2 [%]**	19.20	18.60	17.50	47.10
**Events in super G_2 [%]**	-0.37	0.78	1.03	1.46
**Mean G_1 peak value**	394	410	379	397
**Mean G_2 peak value**	760	760	731	765
**G_1 peak CV**	8.23	11.50	9.30	8.18
**G_2 peak CV**	7.87	8.12	7.80	10.10

Caco-2 cells, 40 µM resveratrol and IRA 5

Experiment 1

24 h incubation time

	0.1 % DMSO	resveratrol	IRA 5	10 µM noc.
**Model used to fit histogram**	Watson	Watson	Watson	Watson
**Goodness of fit (RMS)**	2.52	2.86	2.40	3.43
**Events in sub G_1 [%]**	8.40	8.73	5.99	10.40
**Events in G_1 [%]**	30.10	27.90	18.00	5.60
**Events in S [%]**	44.60	59.40	64.50	29.00
**Events in G_2 [%]**	19.80	3.59	15.80	59.10
**Events in super G_2 [%]**	-0.33	0.41	-1.23	-0.74
**Mean G_1 peak value**	320	357	347	340
**Mean G_2 peak value**	618	689	669	656
**G_1 peak CV**	7.96	8.17	8.56	13.20
**G_2 peak CV**	9.02	4.17	7.11	8.97

48 h incubation time

	0.1 % DMSO	resveratrol	IRA 5	10 µM noc.
**Model used to fit histogram**	Watson	Watson	Watson	Watson
**Goodness of fit (RMS)**	2.00	5.34	4.12	5.66
**Events in sub G_1 [%]**	5.78	20.30	16.30	20.00
**Events in G_1 [%]**	39.60	18.30	26.70	6.84
**Events in S [%]**	36.20	56.90	44.40	26.30
**Events in G_2 [%]**	21.40	7.48	18.60	56.40
**Events in super G_2 [%]**	-1.01	-0.25	-0.90	-2.70
**Mean G_1 peak value**	343	356	363	345
**Mean G_2 peak value**	663	688	688	666
**G_1 peak CV**	7.51	9.12	9.95	17.00
**G_2 peak CV**	9.58	8.69	9.05	10.00

Experiment 2

24 h incubation time

	0.1 % DMSO	resveratrol	IRA 5	10 µM noc.
**Model used to fit histogram**	Watson	Watson	Watson	Watson
**Goodness of fit (RMS)**	1.89	2.73	3.08	4.64
**Events in sub G_1 [%]**	5.26	11.10	9.04	7.54
**Events in G_1 [%]**	31.70	26.90	17.30	2.55
**Events in S [%]**	41.80	55.50	60.80	28.50
**Events in G_2 [%]**	23.90	6.64	20.10	56.10
**Events in super G_2 [%]**	-0.81	0.39	-1.95	-4.05
**Mean G_1 peak value**	370	376	377	374
**Mean G_2 peak value**	715	725	728	723
**G_1 peak CV**	8.12	9.76	7.34	7.59
**G_2 peak CV**	8.59	7.37	8.21	7.77

48 h incubation time

	0.1 % DMSO	resveratrol	IRA 5	10 µM noc.
**Model used to fit histogram**	Watson	Watson	Watson	Watson
**Goodness of fit (RMS)**	1.47	2.86	3.55	3.46
**Events in sub G_1 [%]**	4.42	11.10	15.60	14.10
**Events in G_1 [%]**	40.50	16.80	27.10	3.64
**Events in S [%]**	34.30	68.60	48.00	31.30
**Events in G_2 [%]**	21.30	7.11	15.80	57.60
**Events in super G_2 [%]**	0.41	-0.51	-1.32	-2.20
**Mean G_1 peak value**	371	383	387	379
**Mean G_2 peak value**	717	739	747	731
**G_1 peak CV**	7.70	7.70	9.51	14.30
**G_2 peak CV**	8.51	6.63	8.14	8.48

Experiment 3

24 h incubation time

	0.1 % DMSO	resveratrol	IRA 5	10 µM noc.
**Model used to fit histogram**	Watson	Watson	Watson	Watson
**Goodness of fit (RMS)**	1.71	2.65	3.12	4.23
**Events in sub G_1 [%]**	4.77	11.50	9.11	10.20
**Events in G_1 [%]**	32.40	36.00	18.80	3.89
**Events in S [%]**	46.30	50.90	62.40	22.50
**Events in G_2 [%]**	15.40	3.93	13.50	56.70
**Events in super G_2 [%]**	1.72	-0.26	-0.03	2.15
**Mean G_1 peak value**	369	391	372	372
**Mean G_2 peak value**	712	755	717	717
**G_1 peak CV**	8.19	9.68	7.77	10.00
**G_2 peak CV**	6.33	5.39	7.35	7.26

48 h incubation time

	0.1 % DMSO	resveratrol	IRA 5	10 µM noc.
**Model used to fit histogram**	Watson	Watson	Watson	Watson
**Goodness of fit (RMS)**	1.84	4.06	3.72	3.45
**Events in sub G_1 [%]**	4.58	17.80	14.00	12.20
**Events in G_1 [%]**	38.30	19.10	29.80	3.01
**Events in S [%]**	37.20	59.60	45.70	38.50
**Events in G_2 [%]**	18.80	6.81	14.50	50.40
**Events in super G_2 [%]**	1.98	0.14	1.49	-2.62
**Mean G_1 peak value**	383	383	377	389
**Mean G_2 peak value**	739	739	728	751
**G_1 peak CV**	7.00	8.95	7.81	18.70
**G_2 peak CV**	7.15	9.78	7.21	7.03

Experiment 4

24 h incubation time

	0.1 % DMSO	resveratrol	IRA 5	10 µM noc.
**Model used to fit histogram**	Watson	Watson	Watson	Watson
**Goodness of fit (RMS)**	1.96	2.42	3.20	4.72
**Events in sub G_1 [%]**	4.51	9.49	9.61	13.00
**Events in G_1 [%]**	37.10	37.70	20.30	3.57
**Events in S [%]**	36.80	50.90	50.60	22.20
**Events in G_2 [%]**	26.30	3.37	23.50	55.20
**Events in super G_2 [%]**	-0.90	-0.36	-0.14	1.02
**Mean G_1 peak value**	329	372	327	341
**Mean G_2 peak value**	635	717	632	658
**G_1 peak CV**	9.66	14.70	8.18	9.45
**G_2 peak CV**	11.30	7.54	10.10	9.75

48 h incubation time

	0.1 % DMSO	resveratrol	IRA 5	10 µM noc.
**Model used to fit histogram**	Watson	Watson	Watson	Watson
**Goodness of fit (RMS)**	2.21	3.99	3.56	5.30
**Events in sub G_1 [%]**	4.35	12.00	12.80	20.70
**Events in G_1 [%]**	49.10	17.00	34.10	5.44
**Events in S [%]**	29.80	64.40	42.30	30.30
**Events in G_2 [%]**	19.70	11.60	14.40	50.40
**Events in super G_2 [%]**	0.44	-1.31	-0.86	0.98
**Mean G_1 peak value**	334	351	354	351
**Mean G_2 peak value**	644	677	704	677
**G_1 peak CV**	10.40	9.47	9.24	16.70
**G_2 peak CV**	12.20	10.30	7.53	9.67

Caco-2 cells, 80 µM resveratrol and IRA 5

Experiment 1

24 h incubation time

	0.1 % DMSO	resveratrol	IRA 5	10 µM noc.
**Model used to fit histogram**	Watson	Watson	Watson	Watson
**Goodness of fit (RMS)**	1.77	4.84	3.20	4.62
**Events in sub G_1 [%]**	8.04	22.00	12.00	13.30
**Events in G_1 [%]**	35.30	39.80	48.60	2.83
**Events in S [%]**	37.20	33.80	39.10	20.10
**Events in G_2 [%]**	21.20	6.40	4.18	62.20
**Events in super G_2 [%]**	-0.59	-0.30	-0.24	1.19
**Mean G_1 peak value**	334	351	372	342
**Mean G_2 peak value**	645	682	690	660
**G_1 peak CV**	9.81	10.90	15.10	9.59
**G_2 peak CV**	9.57	6.93	4.95	8.67

48 h incubation time

	0.1 % DMSO	resveratrol	IRA 5	10 µM noc.
**Model used to fit histogram**	Watson	Watson	Watson	Watson
**Goodness of fit (RMS)**	1.89	5.07	7.83	5.45
**Events in sub G_1 [%]**	4.27	18.30	26.20	23.50
**Events in G_1 [%]**	53.20	47.50	16.90	7.27
**Events in S [%]**	28.20	31.80	51.70	23.00
**Events in G_2 [%]**	14.90	3.75	8.60	57.00
**Events in super G_2 [%]**	1.43	0.00	-0.31	-0.28
**Mean G_1 peak value**	335	361	344	339
**Mean G_2 peak value**	646	696	664	654
**G_1 peak CV**	9.41	10.80	9.11	16.30
**G_2 peak CV**	8.42	6.63	8.51	12.50

Experiment 2

24 h incubation time

	0.1 % DMSO	resveratrol	IRA 5	10 µM noc.
**Model used to fit histogram**	Watson	Watson	Watson	Watson
**Goodness of fit (RMS)**	3.16	4.05	3.44	3.24
**Events in sub G_1 [%]**	6.70	15.20	8.53	12.20
**Events in G_1 [%]**	38.70	35.90	49.40	3.72
**Events in S [%]**	37.80	39.40	38.20	33.80
**Events in G_2 [%]**	20.30	6.66	7.97	47.10
**Events in super G_2 [%]**	-0.33	0.37	-0.36	0.33
**Mean G_1 peak value**	344	344	360	344
**Mean G_2 peak value**	664	664	662	662
**G_1 peak CV**	7.37	9.39	10.30	7.37
**G_2 peak CV**	8.30	8.99	12.60	8.13

48 h incubation time

	0.1 % DMSO	resveratrol	IRA 5	10 µM noc.
**Model used to fit histogram**	Watson	Watson	Watson	Watson
**Goodness of fit (RMS)**	2.78	6.84	7.01	3.65
**Events in sub G_1 [%]**	5.45	24.80	25.20	14.40
**Events in G_1 [%]**	47.60	43.20	20.40	4.39
**Events in S [%]**	30.80	29.30	47.30	20.80
**Events in G_2 [%]**	19.50	4.59	6.64	58.70
**Events in super G_2 [%]**	0.16	-0.32	0.69	-1.33
**Mean G_1 peak value**	345	363	345	351
**Mean G_2 peak value**	665	712	666	677
**G_1 peak CV**	10.00	12.30	8.65	11.40
**G_2 peak CV**	9.70	6.97	9.54	8.83

Experiment 3

24 h incubation time

	0.1 % DMSO	resveratrol	IRA 5	10 µM noc.
**Model used to fit histogram**	Watson	Watson	Watson	Watson
**Goodness of fit (RMS)**	2.17	4.11	2.83	5.51
**Events in sub G_1 [%]**	6.44	16.50	9.91	13.30
**Events in G_1 [%]**	33.90	33.00	46.40	2.00
**Events in S [%]**	42.90	40.50	40.00	22.10
**Events in G_2 [%]**	20.20	9.02	4.92	57.60
**Events in super G_2 [%]**	-0.94	-0.66	-0.13	0.64
**Mean G_1 peak value**	377	377	388	369
**Mean G_2 peak value**	728	728	749	712
**G_1 peak CV**	7.66	7.66	9.23	7.84
**G_2 peak CV**	7.08	9.82	6.43	7.26

48 h incubation time

	0.1 % DMSO	resveratrol	IRA 5	10 µM noc.
**Model used to fit histogram**	Watson	Watson	Watson	Watson
**Goodness of fit (RMS)**	2.39	8.19	8.30	6.31
**Events in sub G_1 [%]**	5.07	23.90	27.80	27.40
**Events in G_1 [%]**	46.20	40.10	18.00	4.22
**Events in S [%]**	31.50	34.40	48.50	21.00
**Events in G_2 [%]**	18.40	4.36	7.13	50.60
**Events in super G_2 [%]**	0.57	-0.09	-0.05	-2.71
**Mean G_1 peak value**	363	381	357	384
**Mean G_2 peak value**	700	736	706	741
**G_1 peak CV**	8.26	11.30	11.60	11.30
**G_2 peak CV**	8.20	5.75	9.49	8.11

Experiment 4

24 h incubation time

	0.1 % DMSO	resveratrol	IRA 5	10 µM noc.
**Model used to fit histogram**	Watson	Watson	Watson	Watson
**Goodness of fit (RMS)**	1.66	3.01	1.78	4.67
**Events in sub G_1 [%]**	3.25	12.00	4.70	9.85
**Events in G_1 [%]**	39.90	39.00	50.50	4.63
**Events in S [%]**	39.40	39.10	37.20	42.90
**Events in G_2 [%]**	16.50	11.80	7.17	53.40
**Events in super G_2 [%]**	1.44	1.06	0.66	-2.33
**Mean G_1 peak value**	383	380	403	377
**Mean G_2 peak value**	739	733	777	728
**G_1 peak CV**	10.50	10.40	12.10	12.60
**G_2 peak CV**	9.06	10.20	8.90	10.20

48 h incubation time

	0.1 % DMSO	resveratrol	IRA 5	10 µM noc.
**Model used to fit histogram**	Watson	Watson	Watson	Watson
**Goodness of fit (RMS)**	1.90	5.73	6.05	5.04
**Events in sub G_1 [%]**	4.19	20.90	16.30	14.90
**Events in G_1 [%]**	42.20	40.10	28.50	3.94
**Events in S [%]**	36.40	36.20	46.70	22.10
**Events in G_2 [%]**	18.80	4.23	4.90	51.30
**Events in super G_2 [%]**	0.12	-0.47	1.10	0.59
**Mean G_1 peak value**	369	402	380	380
**Mean G_2 peak value**	712	776	734	734
**G_1 peak CV**	10.40	12.20	12.10	12.70
**G_2 peak CV**	10.30	8.69	7.63	8.28

HCA-7 cells, 10 µM resveratrol and IRA 5

Experiment 1

24 h incubation time

	**0.1 % DMSO**	**resveratrol**	**IRA 5**	**10 µM noc.**
**Model used to fit histogram**	Watson	Watson	Watson	Watson
**Goodness of fit (RMS)**	5.29	8.75	9.02	7.49
**Events in sub G_1 [%]**	15.60	11.80	11.30	14.80
**Events in G_1 [%]**	28.30	23.40	31.40	13.10
**Events in S [%]**	46.60	58.40	54.50	38.40
**Events in G_2 [%]**	14.10	10.40	7.91	36.00
**Events in super G_2 [%]**	-0.27	-1.44	-0.91	-0.58
**Mean G_1 peak value**	200	212	209	200
**Mean G_2 peak value**	389	414	408	377
**G_1 peak CV**	7.93	5.69	5.97	6.05
**G_2 peak CV**	4.51	3.43	3.88	5.35

48 h incubation time

	**0.1 % DMSO**	**resveratrol**	**IRA 5**	**10 µM noc.**
**Model used to fit histogram**	Watson	Watson	Watson	Watson
**Goodness of fit (RMS)**	4.74	6.31	5.06	23.60
**Events in sub G_1 [%]**	8.27	8.26	8.41	37.70
**Events in G_1 [%]**	35.20	42.40	30.70	13.20
**Events in S [%]**	38.60	41.00	50.30	14.70
**Events in G_2 [%]**	19.50	14.60	15.60	35.30
**Events in super G_2 [%]**	0.51	-2.53	-1.15	1.69
**Mean G_1 peak value**	200	212	206	192
**Mean G_2 peak value**	389	414	402	374
**G_1 peak CV**	6.11	6.75	5.91	17.10
**G_2 peak CV**	5.26	5.99	5.32	5.91

Experiment 2

24 h incubation time

	0.1 % DMSO	resveratrol	IRA 5	10 µM noc.
**Model used to fit histogram**	Watson	Watson	Watson	Watson
**Goodness of fit (RMS)**	7.56	4.68	5.24	8.42
**Events in sub G_1 [%]**	8.84	5.32	5.16	14.00
**Events in G_1 [%]**	18.00	18.60	45.00	15.20
**Events in S [%]**	57.90	62.40	41.70	31.60
**Events in G_2 [%]**	17.10	15.40	9.97	43.20
**Events in super G_2 [%]**	0.50	1.33	0.68	4.70
**Mean G_1 peak value**	194	188	195	192
**Mean G_2 peak value**	379	368	379	354
**G_1 peak CV**	4.11	4.35	6.87	6.41
**G_2 peak CV**	3.91	6.50	8.28	6.94

48 h incubation time

	0.1 % DMSO	resveratrol	IRA 5	10 µM noc.
**Model used to fit histogram**	Watson	Watson	Watson	Watson
**Goodness of fit (RMS)**	4.05	3.43	3.50	17.20
**Events in sub G_1 [%]**	6.52	5.48	5.75	31.60
**Events in G_1 [%]**	31.60	38.00	28.80	8.77
**Events in S [%]**	39.20	36.50	51.30	20.90
**Events in G_2 [%]**	21.30	17.60	13.30	39.30
**Events in super G_2 [%]**	2.71	0.87	1.55	2.09
**Mean G_1 peak value**	180	192	175	183
**Mean G_2 peak value**	350	375	348	356
**G_1 peak CV**	5.16	5.06	6.42	13.30
**G_2 peak CV**	5.58	4.79	5.61	6.01

Experiment 3

24 h incubation time

	0.1 % DMSO	resveratrol	IRA 5	10 µM noc.
**Model used to fit histogram**	Watson	Watson	Watson	Watson
**Goodness of fit (RMS)**	5.89	8.45	6.02	4.31
**Events in sub G_1 [%]**	7.31	7.98	6.54	10.40
**Events in G_1 [%]**	17.40	25.30	43.00	7.37
**Events in S [%]**	50.60	54.70	42.70	38.40
**Events in G_2 [%]**	24.70	16.50	9.30	40.70
**Events in super G_2 [%]**	1.34	0.87	0.16	4.59
**Mean G_1 peak value**	196	198	203	195
**Mean G_2 peak value**	381	373	397	373
**G_1 peak CV**	5.11	5.67	9.60	4.42
**G_2 peak CV**	4.57	10.40	7.89	5.66

48 h incubation time

	0.1 % DMSO	resveratrol	IRA 5	10 µM noc.
**Model used to fit histogram**	Watson	Watson	Watson	Watson
**Goodness of fit (RMS)**	9.35	8.29	4.64	12.50
**Events in sub G_1 [%]**	6.18	7.14	4.75	27.00
**Events in G_1 [%]**	38.40	42.90	25.40	7.20
**Events in S [%]**	31.40	36.90	52.30	18.60
**Events in G_2 [%]**	29.90	15.30	20.00	45.80
**Events in super G_2 [%]**	1.16	-0.09	0.27	4.03
**Mean G_1 peak value**	192	196	192	195
**Mean G_2 peak value**	369	381	374	360
**G_1 peak CV**	5.19	5.08	4.88	11.00
**G_2 peak CV**	4.90	4.67	4.71	5.79

Experiment 4

24 h incubation time

	0.1 % DMSO	resveratrol	IRA 5	10 µM noc.
**Model used to fit histogram**	Watson	Watson	Watson	Watson
**Goodness of fit (RMS)**	6.68	7.26	7.31	12.40
**Events in sub G_1 [%]**	10.90	11.60	9.56	21.00
**Events in G_1 [%]**	21.50	23.00	33.60	23.00
**Events in S [%]**	53.50	50.40	47.90	30.80
**Events in G_2 [%]**	17.10	14.80	11.20	30.70
**Events in super G_2 [%]**	1.29	0.89	3.45	0.88
**Mean G_1 peak value**	186	183	186	180
**Mean G_2 peak value**	362	369	362	345
**G_1 peak CV**	5.53	3.70	7.12	10.60
**G_2 peak CV**	4.54	8.46	6.17	7.33

48 h incubation time

	0.1 % DMSO	resveratrol	IRA 5	10 µM noc.
**Model used to fit histogram**	Watson	Watson	Watson	Watson
**Goodness of fit (RMS)**	4.38	7.63	4.15	15.70
**Events in sub G_1 [%]**	7.20	9.62	5.60	27.40
**Events in G_1 [%]**	31.50	36.10	32.60	11.40
**Events in S [%]**	41.40	41.70	44.90	19.50
**Events in G_2 [%]**	20.90	18.10	15.50	41.30
**Events in super G_2 [%]**	0.58	-1.71	2.44	3.57
**Mean G_1 peak value**	184	189	170	177
**Mean G_2 peak value**	359	368	335	333
**G_1 peak CV**	5.24	6.85	5.39	11.90
**G_2 peak CV**	5.78	4.96	6.21	7.11

HCA-7 cells, 40 µM resveratrol and IRA 5

Experiment 1

24 h incubation time

	0.1 % DMSO	resveratrol	IRA 5	10 µM noc.
**Model used to fit histogram**	Watson	Watson	Watson	Watson
**Goodness of fit (RMS)**	4.79	6.13	4.68	10.30
**Events in sub G_1 [%]**	13.00	14.50	11.80	15.30
**Events in G_1 [%]**	25.40	52.00	34.00	13.80
**Events in S [%]**	46.10	20.30	39.00	32.10
**Events in G_2 [%]**	16.40	16.20	13.50	40.80
**Events in super G_2 [%]**	2.16	2.23	3.68	5.22
**Mean G_1 peak value**	202	206	206	198
**Mean G_2 peak value**	395	402	402	365
**G_1 peak CV**	6.97	6.95	7.87	6.73
**G_2 peak CV**	5.03	7.07	6.88	5.70

48 h incubation time

	0.1 % DMSO	resveratrol	IRA 5	10 µM noc.
**Model used to fit histogram**	Watson	Watson	Watson	Watson
**Goodness of fit (RMS)**	4.49	5.72	6.78	24.40
**Events in sub G_1 [%]**	8.91	8.99	12.60	37.20
**Events in G_1 [%]**	29.70	15.40	44.40	8.99
**Events in S [%]**	37.80	69.30	35.20	18.90
**Events in G_2 [%]**	23.90	8.67	16.10	34.00
**Events in super G_2 [%]**	2.64	0.73	-1.85	4.46
**Mean G_1 peak value**	196	201	206	177
**Mean G_2 peak value**	377	391	402	351
**G_1 peak CV**	5.72	8.82	9.80	14.60
**G_2 peak CV**	5.35	10.50	8.15	6.60

Experiment 2

24 h incubation time

	0.1 % DMSO	resveratrol	IRA 5	10 µM noc.
**Model used to fit histogram**	Watson	Watson	Watson	Watson
**Goodness of fit (RMS)**	6.68	10.40	6.59	7.57
**Events in sub G_1 [%]**	7.06	9.34	6.21	12.40
**Events in G_1 [%]**	21.40	60.70	40.50	12.00
**Events in S [%]**	54.20	23.00	46.60	33.90
**Events in G_2 [%]**	21.30	10.30	11.80	42.40
**Events in super G_2 [%]**	-0.82	-0.83	-0.05	3.14
**Mean G_1 peak value**	192	197	201	180
**Mean G_2 peak value**	375	385	377	336
**G_1 peak CV**	4.06	4.90	7.18	6.15
**G_2 peak CV**	4.54	6.83	8.61	7.16

48 h incubation time

	0.1 % DMSO	resveratrol	IRA 5	10 µM noc.
**Model used to fit histogram**	Watson	Watson	Watson	Watson
**Goodness of fit (RMS)**	9.68	5.55	6.89	18.00
**Events in sub G_1 [%]**	9.20	4.78	12.70	32.20
**Events in G_1 [%]**	34.30	21.60	43.30	11.40
**Events in S [%]**	39.60	70.50	33.90	19.20
**Events in G_2 [%]**	19.20	6.65	11.30	39.30
**Events in super G_2 [%]**	0.26	0.02	1.32	0.45
**Mean G_1 peak value**	187	195	189	195
**Mean G_2 peak value**	365	379	368	360
**G_1 peak CV**	4.36	9.29	8.51	19.50
**G_2 peak CV**	4.73	11.00	6.33	7.45

Experiment 3

24 h incubation time

	0.1 % DMSO	resveratrol	IRA 5	10 µM noc.
**Model used to fit histogram**	Watson	Watson	Watson	Watson
**Goodness of fit (RMS)**	4.59	9.55	2.94	5.61
**Events in sub G_1 [%]**	7.24	10.90	4.75	12.50
**Events in G_1 [%]**	12.40	51.40	37.40	16.30
**Events in S [%]**	57.60	21.50	39.90	29.20
**Events in G_2 [%]**	26.20	18.50	18.10	43.10
**Events in super G_2 [%]**	-0.49	1.77	1.01	3.48
**Mean G_1 peak value**	210	205	214	203
**Mean G_2 peak value**	411	400	418	386
**G_1 peak CV**	3.51	6.16	6.69	8.78
**G_2 peak CV**	5.81	6.07	7.87	6.16

48 h incubation time

	0.1 % DMSO	resveratrol	IRA 5	10 µM noc.
**Model used to fit histogram**	Watson	Watson	Watson	Watson
**Goodness of fit (RMS)**	2.60	4.13	9.26	13.00
**Events in sub G_1 [%]**	3.77	3.78	11.80	26.80
**Events in G_1 [%]**	24.70	23.50	31.40	10.40
**Events in S [%]**	45.30	67.20	46.70	19.70
**Events in G_2 [%]**	22.60	9.21	11.60	41.00
**Events in super G_2 [%]**	3.10	-0.77	0.59	0.48
**Mean G_1 peak value**	203	218	209	201
**Mean G_2 peak value**	398	425	410	393
**G_1 peak CV**	4.74	7.81	4.97	16.80
**G_2 peak CV**	4.75	9.96	3.64	6.12

Experiment 4

24 h incubation time

	0.1 % DMSO	resveratrol	IRA 5	10 µM noc.
**Model used to fit histogram**	Watson	Watson	Watson	Watson
**Goodness of fit (RMS)**	7.40	12.50	5.93	11.30
**Events in sub G_1 [%]**	12.20	14.90	8.78	26.40
**Events in G_1 [%]**	28.30	59.30	39.60	13.30
**Events in S [%]**	45.40	16.70	34.90	28.20
**Events in G_2 [%]**	15.40	13.80	17.80	35.10
**Events in super G_2 [%]**	4.14	1.27	1.14	2.96
**Mean G_1 peak value**	175	176	175	175
**Mean G_2 peak value**	337	347	348	324
**G_1 peak CV**	7.50	6.05	6.90	6.90
**G_2 peak CV**	5.24	5.10	7.67	7.32

48 h incubation time

	0.1 % DMSO	resveratrol	IRA 5	10 µM noc.
**Model used to fit histogram**	Watson	Watson	Watson	Watson
**Goodness of fit (RMS)**	3.37	7.15	8.05	17.00
**Events in sub G_1 [%]**	6.89	6.76	12.90	28.70
**Events in G_1 [%]**	27.00	23.60	38.20	13.20
**Events in S [%]**	42.80	63.70	43.00	20.10
**Events in G_2 [%]**	24.40	9.82	12.80	41.90
**Events in super G_2 [%]**	0.25	1.63	-0.72	0.59
**Mean G_1 peak value**	174	177	180	168
**Mean G_2 peak value**	341	347	353	321
**G_1 peak CV**	5.37	6.79	8.35	18.60
**G_2 peak CV**	6.53	9.13	5.42	6.98

HCA-7 cells, 80 μM resveratrol and IRA 5

Experiment 1

24 h incubation time

	0.1 % DMSO	resveratrol	IRA 5	10 μM noc.
**Model used to fit histogram**	Watson	Watson	Watson	Watson
**Goodness of fit (RMS)**	5.18	7.60	5.37	10.00
**Events in sub G_1 [%]**	11.70	17.20	14.80	18.80
**Events in G_1 [%]**	18.90	60.70	53.40	15.30
**Events in S [%]**	46.50	16.00	20.30	28.20
**Events in G_2 [%]**	21.80	11.40	11.40	41.40
**Events in super G_2 [%]**	5.12	1.03	2.26	3.77
**Mean G_1 peak value**	195	206	209	201
**Mean G_2 peak value**	379	402	408	365
**G_1 peak CV**	6.62	8.90	8.62	8.57
**G_2 peak CV**	7.51	7.33	6.54	6.32

48 h incubation time

	0.1 % DMSO	resveratrol	IRA 5	10 μM noc.
**Model used to fit histogram**	Watson	Watson	Watson	Watson
**Goodness of fit (RMS)**	3.54	9.39	6.09	23.30
**Events in sub G_1 [%]**	6.55	17.50	14.00	36.40
**Events in G_1 [%]**	30.80	54.60	13.90	10.20
**Events in S [%]**	38.90	20.50	66.80	19.80
**Events in G_2 [%]**	24.90	8.83	10.60	31.60
**Events in super G_2 [%]**	0.04	0.66	-0.58	1.97
**Mean G_1 peak value**	193	195	209	162
**Mean G_2 peak value**	376	379	395	368
**G_1 peak CV**	5.59	9.63	8.28	18.80
**G_2 peak CV**	6.15	7.76	6.33	5.97

Experiment 2

24 h incubation time

	0.1 % DMSO	resveratrol	IRA 5	10 µM noc.
**Model used to fit histogram**	Watson	Watson	Watson	Watson
**Goodness of fit (RMS)**	8.73	12.20	10.50	7.03
**Events in sub G_1 [%]**	8.54	12.40	11.10	14.90
**Events in G_1 [%]**	23.60	65.70	58.10	11.50
**Events in S [%]**	48.70	13.80	23.90	34.30
**Events in G_2 [%]**	21.00	11.60	10.30	39.40
**Events in super G_2 [%]**	2.40	0.85	0.04	2.85
**Mean G_1 peak value**	184	187	193	189
**Mean G_2 peak value**	355	364	376	348
**G_1 peak CV**	4.17	4.75	6.17	5.39
**G_2 peak CV**	4.58	5.58	6.95	5.52

48 h incubation time

	0.1 % DMSO	resveratrol	IRA 5	10 µM noc.
**Model used to fit histogram**	Watson	Watson	Watson	Watson
**Goodness of fit (RMS)**	3.35	8.85	12.60	16.80
**Events in sub G_1 [%]**	4.12	13.90	18.80	31.00
**Events in G_1 [%]**	33.00	60.70	19.90	7.45
**Events in S [%]**	38.80	16.70	56.60	21.90
**Events in G_2 [%]**	25.10	10.00	5.12	39.90
**Events in super G_2 [%]**	0.19	-1.24	1.03	-0.77
**Mean G_1 peak value**	187	195	153	183
**Mean G_2 peak value**	364	379	380	348
**G_1 peak CV**	5.19	8.58	32.70	13.10
**G_2 peak CV**	6.34	10.70	4.94	6.90

Experiment 3

24 h incubation time

	**0.1 % DMSO**	**resveratrol**	**IRA 5**	**10 µM noc.**
**Model used to fit histogram**	Watson	Watson	Watson	Watson
**Goodness of fit (RMS)**	6.40	10.00	9.95	5.23
**Events in sub G_1 [%]**	7.45	10.80	12.70	13.80
**Events in G_1 [%]**	19.40	56.50	51.20	9.01
**Events in S [%]**	57.30	19.70	22.60	30.10
**Events in G_2 [%]**	15.40	14.10	15.40	48.40
**Events in super G_2 [%]**	7.45	10.80	12.70	13.80
**Mean G_1 peak value**	203	196	213	198
**Mean G_2 peak value**	397	381	415	372
**G_1 peak CV**	5.32	4.59	5.89	4.49
**G_2 peak CV**	3.19	4.87	6.02	5.80

48 h incubation time

	**0.1 % DMSO**	**resveratrol**	**IRA 5**	**10 µM noc.**
**Model used to fit histogram**	Watson	Watson	Watson	Watson
**Goodness of fit (RMS)**	4.88	13.40	8.18	17.00
**Events in sub G_1 [%]**	4.64	14.40	12.00	30.80
**Events in G_1 [%]**	28.70	61.50	17.80	6.44
**Events in S [%]**	40.80	19.60	67.40	18.20
**Events in G_2 [%]**	29.60	7.92	6.94	41.00
**Events in super G_2 [%]**	4.64	14.40	12.00	30.80
**Mean G_1 peak value**	191	198	215	189
**Mean G_2 peak value**	372	385	391	366
**G_1 peak CV**	4.44	7.57	8.28	9.60
**G_2 peak CV**	5.69	6.46	7.95	5.77

Experiment 4

24 h incubation time

	0.1 % DMSO	resveratrol	IRA 5	10 µM noc.
**Model used to fit histogram**	Watson	Watson	Watson	Watson
**Goodness of fit (RMS)**	8.45	12.00	7.73	10.30
**Events in sub G_1 [%]**	8.19	15.40	12.70	22.00
**Events in G_1 [%]**	29.80	59.40	53.00	14.60
**Events in S [%]**	52.20	12.80	17.30	33.60
**Events in G_2 [%]**	14.90	17.30	18.90	31.40
**Events in super G_2 [%]**	1.01	1.49	0.66	1.17
**Mean G_1 peak value**	168	165	168	166
**Mean G_2 peak value**	328	322	328	330
**G_1 peak CV**	7.14	5.10	5.64	9.81
**G_2 peak CV**	4.97	5.19	7.27	6.81

48 h incubation time

	0.1 % DMSO	resveratrol	IRA 5	10 µM noc.
**Model used to fit histogram**	Watson	Watson	Watson	Watson
**Goodness of fit (RMS)**	3.98	10.80	6.82	13.30
**Events in sub G_1 [%]**	6.49	13.60	12.50	25.10
**Events in G_1 [%]**	30.70	63.40	15.00	5.18
**Events in S [%]**	39.60	14.60	68.40	22.20
**Events in G_2 [%]**	25.30	10.20	8.53	45.20
**Events in super G_2 [%]**	0.52	1.74	-0.52	3.58
**Mean G_1 peak value**	164	164	171	171
**Mean G_2 peak value**	320	320	333	310
**G_1 peak CV**	5.57	6.19	6.52	7.76
**G_2 peak CV**	7.25	5.24	7.95	6.39

HCT-116$^{p53-/-}$ cells, 10 µM resveratrol and IRA 5

Experiment 1

24 h incubation time

	0.1 % DMSO	resveratrol	IRA 5	10 µM noc.
**Model used to fit histogram**	Watson	Watson	Watson	Watson
**Goodness of fit (RMS)**	3.24	3.07	4.07	12.00
**Events in sub G_1 [%]**	1.18	1.71	1.73	1.35
**Events in G_1 [%]**	29.30	23.60	32.40	3.15
**Events in S [%]**	46.40	51.70	42.40	11.80
**Events in G_2 [%]**	23.30	23.30	25.30	73.60
**Events in super G_2 [%]**	1.07	0.76	-0.51	4.66
**Mean G_1 peak value**	193	200	196	203
**Mean G_2 peak value**	376	390	383	370
**G_1 peak CV**	4.64	4.43	4.05	17.10
**G_2 peak CV**	4.90	4.49	4.38	3.88

48 h incubation time

	0.1 % DMSO	resveratrol	IRA 5	10 µM noc.
**Model used to fit histogram**	Watson	Watson	Watson	Watson
**Goodness of fit (RMS)**	3.50	5.27	6.26	3.78
**Events in sub G_1 [%]**	1.97	8.40	2.15	7.15
**Events in G_1 [%]**	46.30	37.70	54.60	2.08
**Events in S [%]**	33.70	40.50	31.40	11.30
**Events in G_2 [%]**	19.90	13.40	13.60	78.70
**Events in super G_2 [%]**	-0.02	0.42	1.25	0.94
**Mean G_1 peak value**	185	195	194	194
**Mean G_2 peak value**	360	380	378	370
**G_1 peak CV**	4.19	4.26	4.26	5.01
**G_2 peak CV**	4.23	3.92	3.64	4.76

Experiment 2

24 h incubation time

	0.1 % DMSO	resveratrol	IRA 5	10 µM noc.
**Model used to fit histogram**	Watson	Watson	Watson	Watson
**Goodness of fit (RMS)**	2.79	2.34	2.38	9.37
**Events in sub G_1 [%]**	2.08	2.29	2.75	1.95
**Events in G_1 [%]**	28.20	26.10	30.20	1.57
**Events in S [%]**	46.80	48.90	44.50	6.69
**Events in G_2 [%]**	21.10	19.90	20.30	87.90
**Events in super G_2 [%]**	1.09	1.86	1.21	-0.32
**Mean G_1 peak value**	248	243	243	234
**Mean G_2 peak value**	484	474	474	445
**G_1 peak CV**	3.92	4.49	3.85	10.80
**G_2 peak CV**	3.51	3.45	3.59	4.09

48 h incubation time

	0.1 % DMSO	resveratrol	IRA 5	10 µM noc.
**Model used to fit histogram**	Watson	Watson	Watson	Watson
**Goodness of fit (RMS)**	3.67	3.96	3.91	9.59
**Events in sub G_1 [%]**	2.27	4.83	2.74	7.44
**Events in G_1 [%]**	39.00	31.70	39.70	4.27
**Events in S [%]**	40.90	47.80	40.20	11.50
**Events in G_2 [%]**	14.10	10.70	14.50	77.30
**Events in super G_2 [%]**	0.74	2.07	0.06	-3.93
**Mean G_1 peak value**	228	235	228	219
**Mean G_2 peak value**	444	458	445	427
**G_1 peak CV**	4.20	4.83	4.68	17.90
**G_2 peak CV**	3.90	3.89	4.56	5.63

Experiment 3

24 h incubation time

	0.1 % DMSO	resveratrol	IRA 5	10 µM noc.
**Model used to fit histogram**	Watson	Watson	Watson	Watson
**Goodness of fit (RMS)**	5.58	2.64	5.98	8.29
**Events in sub G_1 [%]**	2.60	2.14	3.30	1.55
**Events in G_1 [%]**	32.80	23.80	31.10	2.71
**Events in S [%]**	41.10	50.10	41.50	15.60
**Events in G_2 [%]**	28.80	24.80	28.30	80.90
**Events in super G_2 [%]**	-3.06	-0.99	-2.11	-4.05
**Mean G_1 peak value**	202	207	205	201
**Mean G_2 peak value**	394	404	400	383
**G_1 peak CV**	6.61	6.90	6.14	14.30
**G_2 peak CV**	6.89	6.15	6.16	6.04

48 h incubation time

	0.1 % DMSO	resveratrol	IRA 5	10 µM noc.
**Model used to fit histogram**	Watson	Watson	Watson	Watson
**Goodness of fit (RMS)**	6.35	4.51	3.15	7.07
**Events in sub G_1 [%]**	4.16	7.58	3.65	7.26
**Events in G_1 [%]**	42.20	39.00	44.00	6.69
**Events in S [%]**	32.20	38.00	34.30	11.20
**Events in G_2 [%]**	25.70	19.30	17.20	77.00
**Events in super G_2 [%]**	-2.89	-2.44	-0.42	1.42
**Mean G_1 peak value**	203	212	216	209
**Mean G_2 peak value**	396	413	421	386
**G_1 peak CV**	5.66	8.72	6.22	13.00
**G_2 peak CV**	6.26	9.82	5.46	7.25

Experiment 4

24 h incubation time

	0.1 % DMSO	resveratrol	IRA 5	10 µM noc.
**Model used to fit histogram**	Watson	Watson	Watson	Watson
**Goodness of fit (RMS)**	4.74	2.32	3.88	8.10
**Events in sub G_1 [%]**	1.99	2.27	2.08	1.92
**Events in G_1 [%]**	28.40	24.70	29.80	1.68
**Events in S [%]**	45.10	52.20	43.40	11.70
**Events in G_2 [%]**	27.40	19.00	27.20	76.00
**Events in super G_2 [%]**	-2.34	1.14	-1.61	6.06
**Mean G_1 peak value**	219	223	220	214
**Mean G_2 peak value**	427	436	430	409
**G_1 peak CV**	3.97	4.70	4.52	7.54
**G_2 peak CV**	4.57	4.02	4.89	4.23

48 h incubation time

	0.1 % DMSO	resveratrol	IRA 5	10 µM noc.
**Model used to fit histogram**	Watson	Watson	Watson	Watson
**Goodness of fit (RMS)**	4.96	3.25	4.75	7.99
**Events in sub G_1 [%]**	2.75	3.99	3.26	9.09
**Events in G_1 [%]**	38.60	33.00	32.50	9.38
**Events in S [%]**	37.40	45.80	42.10	15.30
**Events in G_2 [%]**	24.00	16.50	21.10	67.10
**Events in super G_2 [%]**	-2.25	0.16	-1.32	-2.58
**Mean G_1 peak value**	219	221	219	218
**Mean G_2 peak value**	428	431	427	416
**G_1 peak CV**	4.01	4.37	4.27	5.29
**G_2 peak CV**	4.59	4.22	4.15	5.60

HCT-116$^{p53-/-}$ cells, 40 µM resveratrol and IRA 5

Experiment 1

24 h incubation time

	0.1 % DMSO	resveratrol	IRA 5	10 µM noc.
**Model used to fit histogram**	Watson	Watson	Watson	Watson
**Goodness of fit (RMS)**	3.43	6.33	3.44	8.20
**Events in sub G_1 [%]**	1.84	2.33	2.17	1.72
**Events in G_1 [%]**	29.90	22.70	18.60	1.46
**Events in S [%]**	46.40	73.10	56.30	9.28
**Events in G_2 [%]**	21.10	6.77	26.30	80.60
**Events in super G_2 [%]**	-0.38	-0.95	-1.5	2.76
**Mean G_1 peak value**	217	230	246	219
**Mean G_2 peak value**	424	448	475	413
**G_1 peak CV**	5.25	6.40	4.90	10.00
**G_2 peak CV**	4.68	4.58	4.60	4.88

48 h incubation time

	0.1 % DMSO	resveratrol	IRA 5	10 µM noc.
**Model used to fit histogram**	Watson	Watson	Watson	Watson
**Goodness of fit (RMS)**	3.81	9.00	3.62	11.70
**Events in sub G_1 [%]**	1.62	11.60	4.05	7.06
**Events in G_1 [%]**	46.80	39.00	40.40	2.44
**Events in S [%]**	34.70	39.90	38.80	10.50
**Events in G_2 [%]**	18.00	12.70	16.40	84.50
**Events in super G_2 [%]**	-1.56	-1.18	-0.19	-6.57
**Mean G_1 peak value**	220	230	222	219
**Mean G_2 peak value**	429	448	434	427
**G_1 peak CV**	4.02	6.09	4.39	10.00
**G_2 peak CV**	4.37	4.51	4.02	5.56

Experiment 2

24 h incubation time

	0.1 % DMSO	resveratrol	IRA 5	10 µM noc.
**Model used to fit histogram**	Watson	Watson	Watson	Watson
**Goodness of fit (RMS)**	4.72	6.91	1.76	7.96
**Events in sub G_1 [%]**	1.87	0.77	1.83	1.83
**Events in G_1 [%]**	33.80	16.10	20.00	1.20
**Events in S [%]**	42.20	72.40	57.70	14.20
**Events in G_2 [%]**	25.70	9.10	19.20	74.80
**Events in super G_2 [%]**	-1.77	5.57	1.02	3.70
**Mean G_1 peak value**	191	201	204	206
**Mean G_2 peak value**	372	372	398	377
**G_1 peak CV**	4.99	5.80	5.45	8.93
**G_2 peak CV**	5.84	5.35	4.58	4.47

48 h incubation time

	0.1 % DMSO	resveratrol	IRA 5	10 µM noc.
**Model used to fit histogram**	Watson	Watson	Watson	Watson
**Goodness of fit (RMS)**	4.20	8.32	4.10	6.18
**Events in sub G_1 [%]**	3.02	7.05	4.50	5.09
**Events in G_1 [%]**	43.60	39.70	32.60	3.44
**Events in S [%]**	33.80	36.50	40.40	10.80
**Events in G_2 [%]**	20.80	21.90	24.60	80.20
**Events in super G_2 [%]**	-1.15	-2.23	-0.86	0.43
**Mean G_1 peak value**	201	206	200	201
**Mean G_2 peak value**	392	402	391	372
**G_1 peak CV**	4.71	7.26	5.12	16.20
**G_2 peak CV**	5.78	5.98	5.84	5.35

Experiment 3

24 h incubation time

	0.1 % DMSO	resveratrol	IRA 5	10 µM noc.
**Model used to fit histogram**	Watson	Watson	Watson	Watson
**Goodness of fit (RMS)**	2.69	9.95	3.31	13.90
**Events in sub G_1 [%]**	1.94	5.99	1.81	1.98
**Events in G_1 [%]**	30.70	21.00	24.40	3.06
**Events in S [%]**	45.60	72.30	50.80	15.60
**Events in G_2 [%]**	21.70	5.11	24.90	73.70
**Events in super G_2 [%]**	0.37	-0.26	-1.21	-3.05
**Mean G_1 peak value**	179	195	192	187
**Mean G_2 peak value**	349	379	374	351
**G_1 peak CV**	5.08	6.55	5.61	7.49
**G_2 peak CV**	4.76	3.79	5.54	5.02

48 h incubation time

	0.1 % DMSO	resveratrol	IRA 5	10 µM noc.
**Model used to fit histogram**	Watson	Watson	Watson	Watson
**Goodness of fit (RMS)**	4.72	4.63	3.58	9.48
**Events in sub G_1 [%]**	1.96	6.34	3.11	3.47
**Events in G_1 [%]**	39.70	31.30	35.10	3.37
**Events in S [%]**	36.00	39.40	41.00	11.70
**Events in G_2 [%]**	25.10	18.80	20.90	84.80
**Events in super G_2 [%]**	-1.91	2.43	-0.62	-3.83
**Mean G_1 peak value**	184	195	194	189
**Mean G_2 peak value**	360	381	378	357
**G_1 peak CV**	4.96	7.49	5.60	10.90
**G_2 peak CV**	5.81	6.25	5.96	5.70

Experiment 4

24 h incubation time

	0.1 % DMSO	resveratrol	IRA 5	10 µM noc.
**Model used to fit histogram**	Watson	Watson	Watson	Watson
**Goodness of fit (RMS)**	3.15	6.83	2.36	22.60
**Events in sub G_1 [%]**	1.47	3.32	1.93	2.42
**Events in G_1 [%]**	32.60	18.20	20.40	2.13
**Events in S [%]**	44.70	76.40	49.90	12.60
**Events in G_2 [%]**	21.00	6.73	26.40	70.00
**Events in super G_2 [%]**	0.07	-0.64	0.28	-4.90
**Mean G_1 peak value**	200	221	216	209
**Mean G_2 peak value**	391	431	422	377
**G_1 peak CV**	4.57	6.25	4.90	11.30
**G_2 peak CV**	4.80	3.65	4.62	6.54

48 h incubation time

	0.1 % DMSO	resveratrol	IRA 5	10 µM noc.
**Model used to fit histogram**	Watson	Watson	Watson	Watson
**Goodness of fit (RMS)**	4.58	11.10	2.73	9.12
**Events in sub G_1 [%]**	2.29	15.40	4.13	5.86
**Events in G_1 [%]**	48.60	42.40	40.00	3.41
**Events in S [%]**	29.80	31.50	35.80	11.00
**Events in G_2 [%]**	17.80	16.40	18.20	82.90
**Events in super G_2 [%]**	-0.23	-1.49	0.67	-5.79
**Mean G_1 peak value**	196	221	211	215
**Mean G_2 peak value**	382	431	410	398
**G_1 peak CV**	4.60	7.79	6.56	11.00
**G_2 peak CV**	5.57	5.17	5.76	6.93

HCT-116$^{p53-/-}$ cells, 80 µM resveratrol and IRA 5

Experiment 1

24 h incubation time

	0.1 % DMSO	resveratrol	IRA 5	10 µM noc.
**Model used to fit histogram**	Watson	Watson	Watson	Watson
**Goodness of fit (RMS)**	3.10	4.52	3.51	9.23
**Events in sub G_1 [%]**	2.13	3.00	2.52	1.99
**Events in G_1 [%]**	28.60	42.80	16.40	2.47
**Events in S [%]**	45.10	45.00	70.80	10.10
**Events in G_2 [%]**	24.50	9.78	10.50	79.90
**Events in super G_2 [%]**	0.40	-0.20	1.08	8.01
**Mean G_1 peak value**	185	201	192	192
**Mean G_2 peak value**	360	378	379	354
**G_1 peak CV**	4.78	7.97	5.76	10.00
**G_2 peak CV**	5.18	6.55	3.75	5.48

48 h incubation time

	0.1 % DMSO	resveratrol	IRA 5	10 µM noc.
**Model used to fit histogram**	Watson	Watson	Watson	Watson
**Goodness of fit (RMS)**	8.62	6.86	5.81	6.38
**Events in sub G_1 [%]**	4.30	10.60	5.21	3.76
**Events in G_1 [%]**	41.40	11.40	37.20	3.14
**Events in S [%]**	38.60	67.60	30.40	11.60
**Events in G_2 [%]**	18.60	14.40	33.80	77.50
**Events in super G_2 [%]**	-0.04	0.65	-3.64	5.37
**Mean G_1 peak value**	190	207	198	192
**Mean G_2 peak value**	371	388	386	351
**G_1 peak CV**	6.13	8.64	5.58	15.40
**G_2 peak CV**	5.12	5.47	7.91	5.32

Experiment 2

24 h incubation time

	0.1 % DMSO	resveratrol	IRA 5	10 µM noc.
**Model used to fit histogram**	Watson	Watson	Watson	Watson
**Goodness of fit (RMS)**	2.59	4.97	5.76	9.37
**Events in sub G_1 [%]**	1.93	3.66	3.71	1.03
**Events in G_1 [%]**	29.90	43.90	15.70	3.18
**Events in S [%]**	44.10	42.10	73.50	13.00
**Events in G_2 [%]**	20.90	8.75	10.80	75.60
**Events in super G_2 [%]**	1.72	1.35	-0.15	1.21
**Mean G_1 peak value**	221	233	224	219
**Mean G_2 peak value**	431	439	437	398
**G_1 peak CV**	4.64	7.36	4.98	20.50
**G_2 peak CV**	3.89	6.30	4.95	5.72

48 h incubation time

	0.1 % DMSO	resveratrol	IRA 5	10 µM noc.
**Model used to fit histogram**	Watson	Watson	Watson	Watson
**Goodness of fit (RMS)**	3.08	12.40	6.97	4.54
**Events in sub G_1 [%]**	1.96	17.70	11.70	5.44
**Events in G_1 [%]**	48.20	19.10	44.40	1.61
**Events in S [%]**	30.90	56.00	25.70	8.68
**Events in G_2 [%]**	20.70	12.50	21.40	82.90
**Events in super G_2 [%]**	-1.25	0.83	-1.25	1.99
**Mean G_1 peak value**	213	221	223	219
**Mean G_2 peak value**	414	432	435	408
**G_1 peak CV**	4.83	10.10	4.82	8.44
**G_2 peak CV**	5.03	5.11	7.03	5.58

Experiment 3

24 h incubation time

	0.1 % DMSO	resveratrol	IRA 5	10 µM noc.
**Model used to fit histogram**	Watson	Watson	Watson	Watson
**Goodness of fit (RMS)**	2.99	6.49	5.35	12.40
**Events in sub G_1 [%]**	2.53	4.07	1.59	2.07
**Events in G_1 [%]**	29.80	39.20	13.80	1.94
**Events in S [%]**	46.90	49.20	71.70	11.00
**Events in G_2 [%]**	20.70	11.50	15.10	74.30
**Events in super G_2 [%]**	-0.14	-0.03	1.11	10.60
**Mean G_1 peak value**	212	224	221	198
**Mean G_2 peak value**	414	408	431	363
**G_1 peak CV**	4.42	9.65	7.29	14.60
**G_2 peak CV**	4.54	8.67	3.72	6.90

48 h incubation time

	0.1 % DMSO	resveratrol	IRA 5	10 µM noc.
**Model used to fit histogram**	Watson	Watson	Watson	Watson
**Goodness of fit (RMS)**	3.80	9.60	5.02	8.76
**Events in sub G_1 [%]**	2.34	14.80	9.04	4.35
**Events in G_1 [%]**	46.90	14.40	31.30	3.49
**Events in S [%]**	33.10	56.90	26.20	12.00
**Events in G_2 [%]**	16.40	20.40	32.80	72.00
**Events in super G_2 [%]**	-0.26	-1.53	-0.04	4.94
**Mean G_1 peak value**	201	215	219	219
**Mean G_2 peak value**	392	420	428	392
**G_1 peak CV**	4.62	6.69	6.24	20.60
**G_2 peak CV**	4.77	6.50	4.66	5.05

Experiment 4

24 h incubation time

	0.1 % DMSO	resveratrol	IRA 5	10 µM noc.
**Model used to fit histogram**	Watson	Watson	Watson	Watson
**Goodness of fit (RMS)**	3.57	4.94	6.82	8.12
**Events in sub G_1 [%]**	1.36	1.69	0.34	0.76
**Events in G_1 [%]**	31.40	38.90	20.30	2.98
**Events in S [%]**	43.50	51.30	73.30	17.30
**Events in G_2 [%]**	25.00	8.45	12.00	75.50
**Events in super G_2 [%]**	-1.63	0.48	-1.11	-1.43
**Mean G_1 peak value**	214	219	218	219
**Mean G_2 peak value**	418	417	425	383
**G_1 peak CV**	4.91	6.83	7.05	32.50
**G_2 peak CV**	5.08	5.46	4.86	6.02

48 h incubation time

	0.1 % DMSO	resveratrol	IRA 5	10 µM noc.
**Model used to fit histogram**	Watson	Watson	Watson	Watson
**Goodness of fit (RMS)**	7.90	9.04	5.47	6.22
**Events in sub G_1 [%]**	6.46	16.60	6.69	3.04
**Events in G_1 [%]**	50.70	13.60	29.60	3.73
**Events in S [%]**	29.20	59.70	25.30	11.90
**Events in G_2 [%]**	15.50	15.80	33.20	75.40
**Events in super G_2 [%]**	-0.23	-1.45	3.27	2.24
**Mean G_1 peak value**	214	221	217	212
**Mean G_2 peak value**	416	431	423	399
**G_1 peak CV**	5.11	7.86	6.16	15.20
**G_2 peak CV**	3.87	4.96	5.04	5.24

LNCaP cells, 10 µM resveratrol and IRA 5

Experiment 1

24 h incubation time

	0.1 % DMSO	resveratrol	IRA 5	10 µM noc.
**Model used to fit histogram**	Watson	Watson	Watson	Watson
**Goodness of fit (RMS)**	2.34	4.54	3.11	3.28
**Events in sub G_1 [%]**	1.64	-2.58	3.05	6.36
**Events in G_1 [%]**	71.90	87.80	79.60	59.30
**Events in S [%]**	21.50	11.90	8.44	12.10
**Events in G_2 [%]**	6.94	5.44	8.90	22.40
**Events in super G_2 [%]**	-0.92	-0.44	-1.27	0.23
**Mean G_1 peak value**	402	390	389	389
**Mean G_2 peak value**	775	752	750	699
**G_1 peak CV**	7.39	8.63	6.94	6.95
**G_2 peak CV**	6.51	7.74	9.49	8.69

48 h incubation time

	0.1 % DMSO	resveratrol	IRA 5	10 µM noc.
**Model used to fit histogram**	Watson	Watson	Watson	Watson
**Goodness of fit (RMS)**	3.45	5.15	2.16	7.39
**Events in sub G_1 [%]**	2.52	2.94	1.94	16.10
**Events in G_1 [%]**	55.40	74.40	71.70	51.60
**Events in S [%]**	32.90	15.80	19.20	17.90
**Events in G_2 [%]**	8.62	7.85	7.46	17.80
**Events in super G_2 [%]**	0.59	0.25	-0.55	-0.69
**Mean G_1 peak value**	375	384	391	398
**Mean G_2 peak value**	724	724	755	740
**G_1 peak CV**	6.08	6.17	7.33	8.01
**G_2 peak CV**	4.04	7.71	5.31	5.89

Experiment 2

24 h incubation time

	0.1 % DMSO	resveratrol	IRA 5	10 µM noc.
**Model used to fit histogram**	Watson	Watson	Watson	Watson
**Goodness of fit (RMS)**	5.22	5.19	5.23	4.95
**Events in sub G_1 [%]**	2.33	2.69	3.73	6.85
**Events in G_1 [%]**	79.50	83.80	80.10	64.00
**Events in S [%]**	13.40	6.79	7.01	9.50
**Events in G_2 [%]**	6.59	6.85	10.10	19.10
**Events in super G_2 [%]**	0.62	1.56	1.03	1.09
**Mean G_1 peak value**	359	361	362	361
**Mean G_2 peak value**	692	692	692	678
**G_1 peak CV**	6.20	5.57	5.53	6.04
**G_2 peak CV**	5.33	6.18	5.21	6.30

48 h incubation time

	0.1 % DMSO	resveratrol	IRA 5	10 µM noc.
**Model used to fit histogram**	Watson	Watson	Watson	Watson
**Goodness of fit (RMS)**	3.01	4.19	2.34	10.10
**Events in sub G_1 [%]**	1.68	2.12	1.85	19.00
**Events in G_1 [%]**	57.40	78.20	76.60	53.90
**Events in S [%]**	29.90	12.90	10.90	12.40
**Events in G_2 [%]**	10.90	9.91	10.80	18.50
**Events in super G_2 [%]**	-0.14	-1.39	-0.90	0.00
**Mean G_1 peak value**	339	354	354	366
**Mean G_2 peak value**	654	683	683	678
**G_1 peak CV**	6.71	6.52	7.93	7.65
**G_2 peak CV**	6.14	8.40	9.43	7.88

Experiment 3

24 h incubation time

	0.1 % DMSO	resveratrol	IRA 5	10 µM noc.
**Model used to fit histogram**	Watson	Watson	Watson	Watson
**Goodness of fit (RMS)**	5.41	3.73	3.76	5.23
**Events in sub G_1 [%]**	1.70	2.83	3.35	10.80
**Events in G_1 [%]**	82.30	84.50	78.40	56.00
**Events in S [%]**	12.60	7.97	10.20	15.30
**Events in G_2 [%]**	6.12	5.53	7.62	17.90
**Events in super G_2 [%]**	0.19	0.66	0.38	1.36
**Mean G_1 peak value**	324	333	329	327
**Mean G_2 peak value**	624	642	640	607
**G_1 peak CV**	8.10	8.74	7.98	8.19
**G_2 peak CV**	6.19	6.02	6.04	8.31

48 h incubation time

	0.1 % DMSO	resveratrol	IRA 5	10 µM noc.
**Model used to fit histogram**	Watson	Watson	Watson	Watson
**Goodness of fit (RMS)**	3.56	2.66	4.19	12.10
**Events in sub G_1 [%]**	2.39	3.16	3.63	29.10
**Events in G_1 [%]**	61.60	71.70	76.30	43.60
**Events in S [%]**	26.80	16.60	12.90	16.00
**Events in G_2 [%]**	10.90	9.70	7.30	17.00
**Events in super G_2 [%]**	0.51	0.11	1.27	0.26
**Mean G_1 peak value**	331	326	329	330
**Mean G_2 peak value**	638	629	635	613
**G_1 peak CV**	8.33	8.19	8.96	9.75
**G_2 peak CV**	5.34	8.01	5.87	12.60

Experiment 4

24 h incubation time

	0.1 % DMSO	resveratrol	IRA 5	10 µM noc.
**Model used to fit histogram**	Watson	Watson	Watson	Watson
**Goodness of fit (RMS)**	6.46	6.27	6.77	6.39
**Events in sub G_1 [%]**	0.96	1.61	2.20	14.20
**Events in G_1 [%]**	79.60	88.80	85.90	49.00
**Events in S [%]**	16.00	6.97	5.75	15.60
**Events in G_2 [%]**	5.66	4.80	9.85	19.80
**Events in super G_2 [%]**	0.89	1.25	0.90	1.30
**Mean G_1 peak value**	309	310	313	310
**Mean G_2 peak value**	597	597	599	587
**G_1 peak CV**	8.88	8.39	7.97	8.04
**G_2 peak CV**	7.22	6.55	6.46	7.35

48 h incubation time

	0.1 % DMSO	resveratrol	IRA 5	10 µM noc.
**Model used to fit histogram**	Watson	Watson	Watson	Watson
**Goodness of fit (RMS)**	1.79	2.67	2.68	9.05
**Events in sub G_1 [%]**	2.55	3.31	2.45	18.30
**Events in G_1 [%]**	55.80	73.90	71.00	40.10
**Events in S [%]**	28.30	14.70	16.50	21.00
**Events in G_2 [%]**	12.80	9.95	11.10	22.30
**Events in super G_2 [%]**	0.68	-0.26	-0.24	-1.48
**Mean G_1 peak value**	311	315	311	313
**Mean G_2 peak value**	599	608	600	603
**G_1 peak CV**	8.36	8.39	9.47	9.71
**G_2 peak CV**	7.54	8.97	8.67	9.90

LNCaP cells, 40 µM resveratrol and IRA 5

Experiment 1

24 h incubation time

	0.1 % DMSO	resveratrol	IRA 5	10 µM noc.
**Model used to fit histogram**	Watson	Watson	Watson	Watson
**Goodness of fit (RMS)**	3.08	5.30	2.86	4.34
**Events in sub G_1 [%]**	3.40	-2.51	5.75	5.02
**Events in G_1 [%]**	69.50	82.80	68.20	57.80
**Events in S [%]**	21.60	10.20	16.80	14.10
**Events in G_2 [%]**	8.93	10.40	8.87	22.40
**Events in super G_2 [%]**	-0.79	-1.18	0.17	0.61
**Mean G_1 peak value**	406	380	385	377
**Mean G_2 peak value**	784	734	743	696
**G_1 peak CV**	9.28	8.18	7.42	7.36
**G_2 peak CV**	7.33	9.38	6.46	8.48

48 h incubation time

	0.1 % DMSO	resveratrol	IRA 5	10 µM noc.
**Model used to fit histogram**	Watson	Watson	Watson	Watson
**Goodness of fit (RMS)**	2.81	2.31	3.21	6.71
**Events in sub G_1 [%]**	2.24	1.41	4.08	17.30
**Events in G_1 [%]**	53.20	75.30	71.10	45.20
**Events in S [%]**	29.80	16.00	14.80	17.40
**Events in G_2 [%]**	15.70	7.14	8.51	19.90
**Events in super G_2 [%]**	-0.79	0.44	0.02	0.29
**Mean G_1 peak value**	368	380	387	398
**Mean G_2 peak value**	711	733	747	743
**G_1 peak CV**	6.52	7.81	7.64	8.40
**G_2 peak CV**	7.10	7.99	6.52	8.29

Experiment 2

24 h incubation time

	0.1 % DMSO	resveratrol	IRA 5	10 µM noc.
**Model used to fit histogram**	Watson	Watson	Watson	Watson
**Goodness of fit (RMS)**	4.29	6.05	3.47	4.13
**Events in sub G_1 [%]**	2.56	2.52	3.65	8.40
**Events in G_1 [%]**	77.80	81.40	73.10	57.80
**Events in S [%]**	14.00	12.00	15.50	11.40
**Events in G_2 [%]**	7.10	7.06	8.46	19.60
**Events in super G_2 [%]**	0.68	0.74	1.28	0.33
**Mean G_1 peak value**	356	358	361	360
**Mean G_2 peak value**	688	691	696	681
**G_1 peak CV**	6.80	5.99	7.60	6.78
**G_2 peak CV**	5.80	4.97	6.27	6.83

48 h incubation time

	0.1 % DMSO	resveratrol	IRA 5	10 µM noc.
**Model used to fit histogram**	Watson	Watson	Watson	Watson
**Goodness of fit (RMS)**	3.74	3.59	4.08	8.03
**Events in sub G_1 [%]**	1.22	2.14	5.80	13.60
**Events in G_1 [%]**	57.50	80.30	74.10	58.20
**Events in S [%]**	29.20	10.70	12.00	12.40
**Events in G_2 [%]**	12.20	6.16	8.78	22.40
**Events in super G_2 [%]**	-0.23	1.32	0.50	-0.21
**Mean G_1 peak value**	333	346	346	357
**Mean G_2 peak value**	643	668	668	666
**G_1 peak CV**	7.38	7.88	7.44	7.69
**G_2 peak CV**	7.07	6.45	7.09	7.98

Experiment 3

24 h incubation time

	0.1 % DMSO	resveratrol	IRA 5	10 µM noc.
**Model used to fit histogram**	Watson	Watson	Watson	Watson
**Goodness of fit (RMS)**	6.35	5.05	6.85	5.32
**Events in sub G_1 [%]**	2.55	4.04	2.79	11.90
**Events in G_1 [%]**	79.30	80.30	78.60	54.00
**Events in S [%]**	14.80	11.80	14.60	16.40
**Events in G_2 [%]**	7.17	6.12	9.20	19.20
**Events in super G_2 [%]**	0.29	0.58	1.18	0.15
**Mean G_1 peak value**	306	303	306	295
**Mean G_2 peak value**	591	591	591	560
**G_1 peak CV**	9.30	8.30	9.30	9.37
**G_2 peak CV**	8.11	6.11	6.61	9.61

48 h incubation time

	0.1 % DMSO	resveratrol	IRA 5	10 µM noc.
**Model used to fit histogram**	Watson	Watson	Watson	Watson
**Goodness of fit (RMS)**	2.24	2.08	5.98	9.58
**Events in sub G_1 [%]**	2.20	1.53	6.98	22.50
**Events in G_1 [%]**	54.60	75.20	75.80	45.60
**Events in S [%]**	28.50	15.90	12.90	17.10
**Events in G_2 [%]**	14.60	7.30	9.43	22.00
**Events in super G_2 [%]**	0.94	0.40	0.29	0.41
**Mean G_1 peak value**	301	314	324	327
**Mean G_2 peak value**	580	606	626	604
**G_1 peak CV**	8.98	9.40	9.51	12.00
**G_2 peak CV**	8.02	11.10	8.45	11.50

Experiment 4

24 h incubation time

	0.1 % DMSO	resveratrol	IRA 5	10 µM noc.
**Model used to fit histogram**	Watson	Watson	Watson	Watson
**Goodness of fit (RMS)**	6.36	3.76	3.07	4.74
**Events in sub G_1 [%]**	6.16	-2.11	1.30	10.40
**Events in G_1 [%]**	71.80	82.60	77.20	60.60
**Events in S [%]**	10.40	11.70	13.20	11.20
**Events in G_2 [%]**	5.11	7.55	9.74	16.80
**Events in super G_2 [%]**	1.47	0.69	0.09	1.00
**Mean G_1 peak value**	371	371	371	371
**Mean G_2 peak value**	716	716	716	687
**G_1 peak CV**	7.50	8.10	8.10	8.10
**G_2 peak CV**	5.88	6.56	7.88	9.08

48 h incubation time

	0.1 % DMSO	resveratrol	IRA 5	10 µM noc.
**Model used to fit histogram**	Watson	Watson	Watson	Watson
**Goodness of fit (RMS)**	7.38	4.15	6.53	6.80
**Events in sub G_1 [%]**	1.33	3.14	4.97	12.40
**Events in G_1 [%]**	71.00	82.00	79.50	62.20
**Events in S [%]**	24.40	9.71	10.10	12.40
**Events in G_2 [%]**	10.90	6.63	7.07	18.70
**Events in super G_2 [%]**	-0.73	0.83	0.81	-0.35
**Mean G_1 peak value**	366	375	372	380
**Mean G_2 peak value**	706	724	717	714
**G_1 peak CV**	8.50	7.01	8.03	7.92
**G_2 peak CV**	6.08	5.52	6.06	8.46

LNCaP cells, 80 µM resveratrol and IRA 5

Experiment 1

24 h incubation time

	**0.1 % DMSO**	**resveratrol**	**IRA 5**	**10 µM noc.**
**Model used to fit histogram**	Watson	Watson	Watson	Watson
**Goodness of fit (RMS)**	3.84	3.02	3.32	6.53
**Events in sub G_1 [%]**	4.38	4.15	6.61	11.90
**Events in G_1 [%]**	75.60	73.10	68.30	63.60
**Events in S [%]**	10.90	13.80	15.20	12.70
**Events in G_2 [%]**	7.16	6.63	7.71	19.50
**Events in super G_2 [%]**	0.65	0.96	1.20	-0.88
**Mean G_1 peak value**	372	370	375	380
**Mean G_2 peak value**	717	714	717	696
**G_1 peak CV**	7.60	7.30	8.52	8.75
**G_2 peak CV**	7.57	6.34	7.95	12.50

48 h incubation time

	**0.1 % DMSO**	**resveratrol**	**IRA 5**	**10 µM noc.**
**Model used to fit histogram**	Watson	Watson	Watson	Watson
**Goodness of fit (RMS)**	2.93	5.25	4.66	8.73
**Events in sub G_1 [%]**	1.48	3.29	7.82	13.00
**Events in G_1 [%]**	61.40	83.70	75.70	52.90
**Events in S [%]**	25.10	9.86	10.60	14.40
**Events in G_2 [%]**	13.90	6.58	5.91	21.80
**Events in super G_2 [%]**	-0.96	-0.49	0.49	-3.40
**Mean G_1 peak value**	363	373	372	372
**Mean G_2 peak value**	700	720	718	717
**G_1 peak CV**	9.01	7.68	7.67	8.55
**G_2 peak CV**	8.60	8.64	8.39	10.20

Experiment 2

24 h incubation time

	0.1 % DMSO	resveratrol	IRA 5	10 µM noc.
**Model used to fit histogram**	Watson	Watson	Watson	Watson
**Goodness of fit (RMS)**	3.36	2.70	5.56	5.77
**Events in sub G_1 [%]**	2.42	5.96	13.50	12.50
**Events in G_1 [%]**	76.50	67.60	64.50	50.00
**Events in S [%]**	13.20	21.10	18.40	13.90
**Events in G_2 [%]**	10.40	8.50	9.25	26.60
**Events in super G_2 [%]**	-0.42	0.22	-0.35	-0.04
**Mean G_1 peak value**	400	403	398	386
**Mean G_2 peak value**	773	773	773	719
**G_1 peak CV**	7.71	7.00	7.83	7.88
**G_2 peak CV**	7.31	8.08	7.79	8.68

48 h incubation time

	0.1 % DMSO	resveratrol	IRA 5	10 µM noc.
**Model used to fit histogram**	Watson	Watson	Watson	Watson
**Goodness of fit (RMS)**	2.14	2.47	2.09	7.64
**Events in sub G_1 [%]**	2.00	1.48	3.30	15.10
**Events in G_1 [%]**	63.80	76.00	75.10	49.90
**Events in S [%]**	23.30	16.60	16.90	13.00
**Events in G_2 [%]**	11.10	8.00	6.52	21.40
**Events in super G_2 [%]**	-0.71	-0.84	-0.93	0.45
**Mean G_1 peak value**	382	383	396	389
**Mean G_2 peak value**	737	739	765	716
**G_1 peak CV**	8.16	8.11	8.85	8.05
**G_2 peak CV**	6.68	9.36	7.86	8.84

Experiment 3

24 h incubation time

	0.1 % DMSO	resveratrol	IRA 5	10 µM noc.
**Model used to fit histogram**	Watson	Watson	Watson	Watson
**Goodness of fit (RMS)**	5.86	7.48	6.39	6.16
**Events in sub G_1 [%]**	2.00	5.24	4.33	9.15
**Events in G_1 [%]**	82.40	81.10	78.80	61.80
**Events in S [%]**	13.10	13.30	14.20	11.80
**Events in G_2 [%]**	6.05	5.90	9.38	23.10
**Events in super G_2 [%]**	-1.59	0.28	-0.87	-1.69
**Mean G_1 peak value**	352	354	354	348
**Mean G_2 peak value**	714	687	684	655
**G_1 peak CV**	8.31	7.76	8.10	8.26
**G_2 peak CV**	8.25	5.57	8.61	10.40

48 h incubation time

	0.1 % DMSO	resveratrol	IRA 5	10 µM noc.
**Model used to fit histogram**	Watson	Watson	Watson	Watson
**Goodness of fit (RMS)**	5.15	6.56	3.26	5.74
**Events in sub G_1 [%]**	1.33	5.91	3.60	12.80
**Events in G_1 [%]**	71.60	75.70	76.30	51.70
**Events in S [%]**	17.00	13.80	13.80	14.80
**Events in G_2 [%]**	13.10	5.17	7.65	15.80
**Events in super G_2 [%]**	-0.77	0.27	0.15	1.50
**Mean G_1 peak value**	354	366	366	369
**Mean G_2 peak value**	678	720	705	705
**G_1 peak CV**	8.88	8.69	9.15	9.72
**G_2 peak CV**	7.95	6.86	7.64	8.48

Experiment 4

24 h incubation time

	0.1 % DMSO	resveratrol	IRA 5	10 µM noc.
**Model used to fit histogram**	Watson	Watson	Watson	Watson
**Goodness of fit (RMS)**	2.57	4.41	6.08	5.86
**Events in sub G_1 [%]**	3.32	13.10	13.20	14.40
**Events in G_1 [%]**	73.20	61.50	61.50	46.20
**Events in S [%]**	14.90	24.70	21.60	15.80
**Events in G_2 [%]**	9.87	4.76	7.50	24.20
**Events in super G_2 [%]**	0.59	0.24	0.09	1.50
**Mean G_1 peak value**	370	384	380	369
**Mean G_2 peak value**	708	746	734	690
**G_1 peak CV**	9.05	7.30	9.64	9.81
**G_2 peak CV**	6.98	4.46	6.06	8.44

48 h incubation time

	0.1 % DMSO	resveratrol	IRA 5	10 µM noc.
**Model used to fit histogram**	Watson	Watson	Watson	Watson
**Goodness of fit (RMS)**	3.16	5.74	2.60	8.69
**Events in sub G_1 [%]**	3.37	-3.14	2.76	16.70
**Events in G_1 [%]**	59.80	84.30	74.10	53.90
**Events in S [%]**	23.90	16.00	17.10	12.70
**Events in G_2 [%]**	12.60	5.67	6.67	25.80
**Events in super G_2 [%]**	0.39	0.19	0.69	-1.10
**Mean G_1 peak value**	366	366	361	383
**Mean G_2 peak value**	696	705	683	699
**G_1 peak CV**	7.79	8.69	8.71	10.30
**G_2 peak CV**	7.50	7.47	7.65	11.30

Raw data of the densitometric evaluation of the COX-2 expression analysis (western blots)

Abbreviations: ctrl.: solvent control (0.1% DMSO); DMSO: dimethyl sulfoxide; I5: IRA 5 (1 = 1 µM, 50 = 50 µM, 100 = 100 µM); R: resveratrol (1 = 1 µM, 50 = 50 µM, 100 = 100 µM)

HCA-7 cells

Experiment 1

24 h incubation time

COX-2	ctrl.*	R 1	R 50	R 100	I5 1	I5 50	I5 100
**Area of density peaks**	13686.1	12976.9	11777.1	8801.1	11181.8	14371.6	19520.7
**Percent of density peaks**	14.8	14.1	12.8	9.5	12.1	15.6	21.1
**Relative density**	1.0	0.9	0.9	0.6	0.8	1.1	1.4
**GAPDH**							
**Area of density peaks**	21380.1	23915.3	27752.1	26935.2	26691.4	25307.2	25734.5
**Percent of density peaks**	12.0	13.5	15.6	15.2	15.0	14.2	14.5
**Relative density**	1.0	1.1	1.3	1.3	1.2	1.2	1.2
**Adjusted relative density**	**1.00**	**0.85**	**0.66**	**0.51**	**0.65**	**0.89**	**1.18**

* 0.1 % DMSO

Experiment 2

24 h incubation time

COX-2	ctrl.*	R 1	R 50	R 100	I5 1	I5 50	I5 100
**Area of density peaks**	7995.4	8707.7	7486.4	6396.0	9345.0	14549.6	21636.5
**Percent of density peaks**	10.5	11.4	9.8	8.4	12.3	19.1	28.4
**Relative density**	1.0	1.1	0.9	0.8	1.2	1.8	2.7
**GAPDH**							
**Area of density peaks**	30352.4	18726.4	18203.9	22237.9	27530.8	21208.9	23294.0
**Percent of density peaks**	18.8	11.6	11.3	13.8	17.0	13.1	14.4
**Relative density**	1.0	0.6	0.6	0.7	0.9	0.7	0.8
**Adjusted relative density**	**1.00**	**1.77**	**1.56**	**1.09**	**1.29**	**2.60**	**3.53**

* 0.1 % DMSO

Experiment 3

24 h incubation time

COX-2	ctrl.*	R 1	R 50	R 100	I5 1	I5 50	I5 100
**Area of density peaks**	9039.3	10291.1	8773.1	5990.9	11916.3	18463.9	21534.9
**Percent of density peaks**	10.5	12.0	10.2	7.0	13.9	21.5	25.0
**Relative density**	1.0	1.1	1.0	0.7	1.3	2.0	2.4
**GAPDH**							
**Area of density peaks**	36197.7	31838.9	27162.7	29574.4	30867.1	24460.7	22854.1
**Percent of density peaks**	17.8	15.7	13.4	14.6	15.2	12.1	11.3
**Relative density**	1.0	0.9	0.8	0.8	0.9	0.7	0.6
**Adjusted relative density**	**1.00**	**1.29**	**1.29**	**0.81**	**1.55**	**3.02**	**3.77**

* 0.1 % DMSO

Raw data of the COX-2 activity analysis

Abbreviations: ctrl.: solvent control (0.1 % DMSO); DMSO: dimethyl sulfoxide; SD: standard deviation

HCA-7 cells

IRA 5

Experiment 1

24 h incubation time

	ctrl.*	0.001 µM	0.01 µM	0.1 µM	1 µM	10 µM	50 µM
**PGE_2 well 1 [nM]**	45.49	54.97	50.33	40.73	24.46	18.92	15.03
**PGE_2 well 2 [nM]**	49.35	63.43	51.75	49.63	24.72	19.47	16.38
**PGE_2 well 3 [nM]**	46.19	49.89	49.39	45.69	26.13	19.18	16.77
**MEAN**	**47.01**	**56.10**	**50.49**	**45.35**	**25.10**	**19.19**	**16.06**
**SD**	2.05	6.84	1.19	4.46	0.90	0.28	0.91

* 0.1 % DMSO

Experiment 2

24 h incubation time

	ctrl.*	0.001 µM	0.01 µM	0.1 µM	1 µM	10 µM	50 µM
**PGE_2 well 1 [nM]**	46.18	47.73	43.57	37.71	26.86	21.79	16.32
**PGE_2 well 2 [nM]**	48.89	45.24	48.25	36.93	27.81	21.77	17.41
**PGE_2 well 3 [nM]**	49.52	42.77	47.67	42.53	27.48	22.06	17.76
**MEAN**	**48.20**	**45.24**	**46.49**	**39.05**	**27.39**	**21.87**	**17.16**
**SD**	1.77	2.48	2.55	3.03	0.48	0.16	0.75

* 0.1 % DMSO

Raw data of the glucuronidation assay in rat liver microsomes

Abbreviations: SD: standard deviation

	0 min incubation time	40 min incubation time	% glucuronidation
**IRA 5 sample 1 [µM]**	22.12	8.31	62.45
**IRA 5 sample 2 [µM]**	21.67	5.88	72.86
**IRA 5 sample 3 [µM]**	19.40	7.65	60.54
**MEAN**	**21.1**	**7.28**	**65.28**
**SD**	1.46	1.25	6.63
